# Transition‐Metal‐Free Coupling of 1,3‐Dipoles and Boronic Acids as a Sustainable Approach to C−C Bond Formation

**DOI:** 10.1002/chem.202001590

**Published:** 2020-07-21

**Authors:** Keith Livingstone, Sophie Bertrand, Alan R. Kennedy, Craig Jamieson

**Affiliations:** ^1^ Department of Pure and Applied Chemistry University of Strathclyde Thomas Graham Building, 295 Cathedral St Glasgow G1 1XL UK; ^2^ GlaxoSmithKline Medicines Research Centre Gunnels Wood Road Stevenage Hertfordshire SG1 2NY UK

**Keywords:** 1,3-dipoles, C−C coupling, nitrile imines, nitrile oxides, reactive intermediates

## Abstract

The need for alternative, complementary approaches to enable C−C bond formation within organic chemistry is an on‐going challenge in the area. Of particular relevance are transformations that proceed in the absence of transition‐metal reagents. In the current study, we report a comprehensive investigation of the coupling of nitrile imines and aryl boronic acids as an approach towards sustainable C−C bond formation. In situ generation of the highly reactive 1,3‐dipole facilitates a Petasis–Mannich‐type coupling via a nucleophilic boronate complex. The introduction of hydrazonyl chlorides as a complementary nitrile imine source to the 2,5‐tetrazoles previously reported by our laboratory further broadens the scope of the approach. Additionally, we exemplify for the first time the extension of this protocol into another 1,3‐dipole, through the synthesis of aryl ketone oximes from aryl boronic acids and nitrile *N*‐oxides.

## Graphical abstract

**A scalable and metal‐free approach** to carbon–carbon bond formation using 1,3‐dipole systems generated from hydrazonyl chloride or hydroxamoyl chloride precursors and aryl boronic acid derivatives is reported. Generation of the 1,3‐dipole results in activation of the boronic acid, with migration of the pendant aryl group to forge a new C−C bond in yields up to 80 % (see scheme).

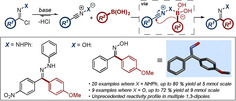

## Introduction

The formation of the C−C bond is of unique importance within synthetic organic chemistry.^1^ While a vast number of research groups have reported a raft of different protocols to facilitate this transformation, the overwhelming majority of these require harsh reaction conditions, or the application of transition metals.^2^ As research within the synthetic chemistry community gravitates towards more sustainable and economical solutions to such challenges,2b the development of complementary methodology towards C−C bond formation that obviates the requirement for expensive or toxic reagents is becoming increasingly important.

Recently, nitrile imines (NIs) have come to our attention as an attractive reagent in this regard.^3^, ^4^ The NI is a highly versatile 1,3‐dipole, first reported by Huisgen in 1959.4a Due to its substantial reactivity, the species normally exists as a transient intermediate, generated in situ from a suitable precursor prior to trapping with an appropriate reaction partner. As with most 1,3‐dipolar moieties, NI chemistry is dominated by 1,3‐dipolar cycloaddition, a versatile transformation with diverse applications in synthesis, bioorthogonal chemistry and materials science.^5^, ^6^ However, an additional aspect of this dipole is its significant reactivity with soft nucleophiles, such as thiols and carboxylic acids.4j, 7

**Scheme 1:**
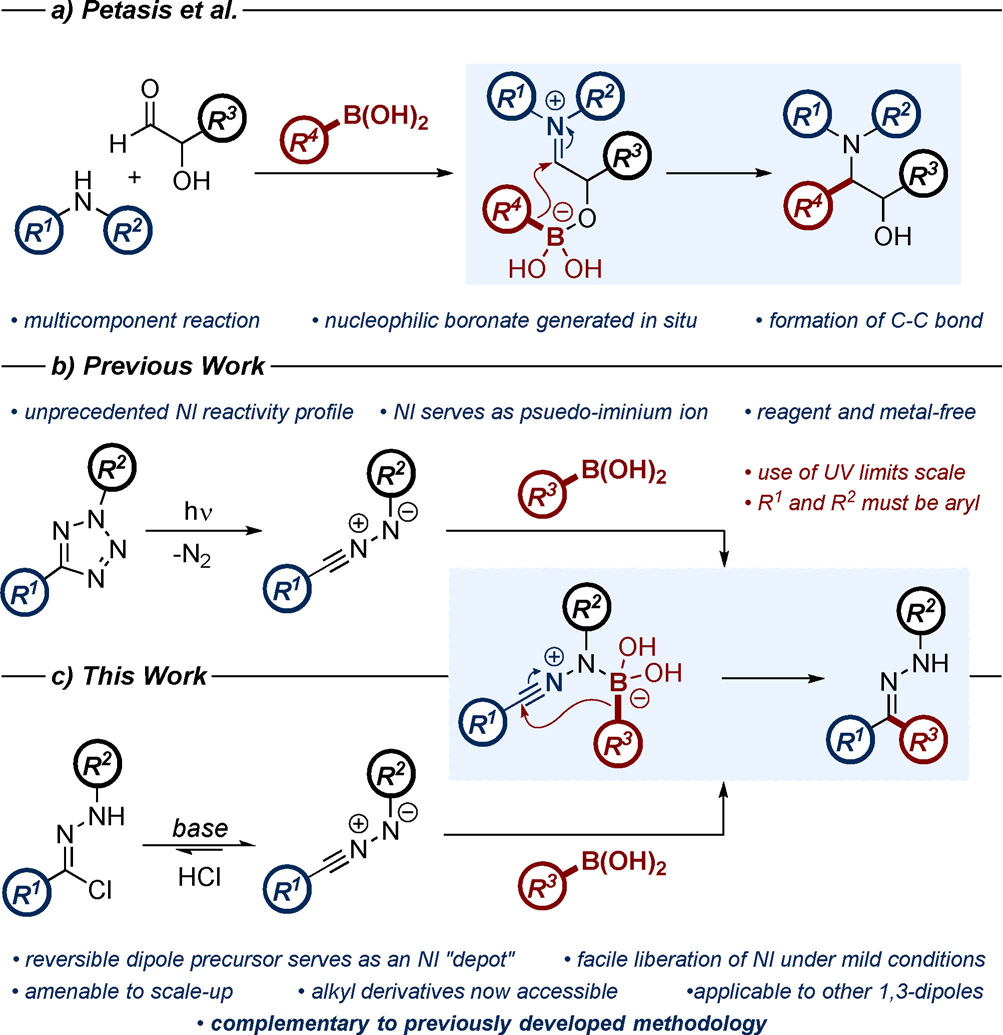
a) An overview of the Petasis–Mannich reaction, which features the generation of the nucleophilic boronate complex in situ. b) Previous efforts within our research group furnished a metal‐free route towards aryl ketone hydrazones employing the photolysis of 2,5‐tetrazole derivatives. c) This work: we introduce hydrazonyl chlorides as an alternative source of the NI 1,3‐dipole, which affords certain practical advantages over the previous methodology, including extension to other 1,3‐dipoles.

In a previous report, we disclosed that by exploiting the pleiotropic reactivity profile of the NI, combination with aryl boronic acids could facilitate C−C bond formation in the absence of any exogenous reagents.^8^ This approach drew on mechanistic inspiration from the work of Petasis, who demonstrated the formation of a C−C bond between α‐alkoxyiminium species and a variety of boronic acids and esters (Scheme 1 a).^9^, ^10^ In both transformations, the key mechanistic step is the formation of a nucleophilic boronate complex via coordination of the boronic acid to a nucleophilic heteroatom of the substrate. This electron‐rich boronate facilitates migration of the pendant aryl group to the electrophilic iminium moiety (or pseudo‐iminium moiety, in the case of the NI).

Our previous efforts in this field largely employed the 2,5‐diaryl tetrazole moiety as an NI precursor (Scheme 1 b).4i, 8, 11 This functionality has the advantage of direct and irreversible NI generation through exposure to 300 nm ultraviolet (UV) light, enabling reagent‐free access to the dipole with only nitrogen gas as a by‐product. However, despite the utility of the method, some limitations with this approach remain. From a practical perspective, the scale upon which photochemistry may be performed is often limited by the size of the light source available. Furthermore, the use of high‐energy UV‐B light carries with it associated health risks, which are exacerbated at a larger scale. Substitution of the NI is also restricted to aryl groups, to ensure that the precursor possesses an appropriate HOMO energy to enable photochemical activation.^12^

Based on all of the above, we sought to harness a complementary approach to NI generation to enhance the applicability of this methodology. Preliminary efforts at the time of our previous report highlighted the potential of hydrazonyl chlorides to serve as such an alternative (Scheme 1 c).4a, 13, 14 NI production from this precursor differs to the 2,5‐tetrazole in that it is a reversible process,11a meaning that the introduction of this method could modulate the extraneous decomposition of the highly reactive dipole. Additionally, it is likely that such a procedure would be more amenable to scale‐up in comparison to our initial protocol, owing to the absence of UV light. Employing similar reasoning, NIs bearing alkyl substituents should also now be compatible within such a manifold, thus expanding the substrate scope. One final advantage of the hydrazonyl chloride as an NI source is that it may hypothetically facilitate extension of the protocol into other 1,3‐dipolar species, owing to the similarity of the precursors. For example, the closely related hydroxamoyl chlorides, a common source of the nitrile oxide (NO) 1,3‐dipole.^15^

## Results and Discussion

**Figure 1:**
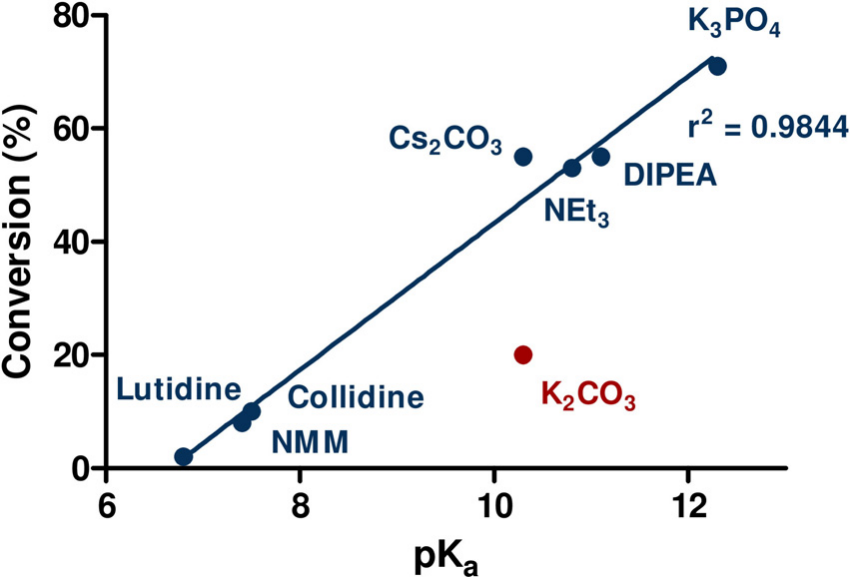
The correlation between the p*K*_a_ of bases employed and reaction conversion is evident within the range of 6.5 to 12.5. The notable outlier, K_2_CO_3_, is thought to possess limited solubility in the reaction solvent (c.f. Cs_2_CO_3_).

Our optimization campaign commenced with the application of hydrazonyl chloride **1a** as an NI precursor, with the electron‐rich 4‐(methoxy)phenylboronic acid (**2a**) selected as an appropriate reaction partner. Initial efforts proved to be encouraging, affording 32 % conversion to the desired reaction product (Table 1, entry 1). The reaction was found to be highly sensitive to both solvent and base selection (see Supporting Information for further details), with DCM and K_3_PO_4_ identified as a favourable combination (Table 1, entry 15). The p*K*_a_ of the base employed was found to be of particular importance,^16^ with a direct correlation evident in the region of 6 to 13 (Figure 1). Interestingly, negligible by‐product formation was observed throughout this optimization, with starting material **1a** comprising the majority of the remaining mass balance.

**Table 1:** Preliminary investigations into solvent and base selection.^[a]^

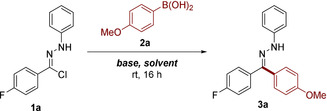				
Entry	Solvent	Base	ArB(OH)_2_ stoichiometry	Conv. [%]^[b]^
1	MeCN	NEt_3_	3	32
2	THF	NEt_3_	3	22
3	EtOAc	NEt_3_	3	48
4	1,4‐dioxane	NEt_3_	3	58
5	PhMe	NEt_3_	3	50
6	DCM	NEt_3_	3	75
7	DCM	NEt_3_	1.1	54
8	DCE	NEt_3_	1.1	40
9	CHCl_3_	NEt_3_	1.1	51
10	DCM	Pyridine	1.1	<1
11	DCM	Collidine	1.1	10
12	DCM	K_2_CO_3_	1.1	20
13	DCM	Cs_2_CO_3_	1.1	55
14	DCM	DIPEA	1.1	55
**15**	**DCM**	**K_3_PO_4_**	**1.1**	**71**
16	DCM	KO^t^Bu	1.1	15

[a] Reactions performed on a 0.1 mmol scale using 3 equiv base at a concentration of 0.1 m. [b] Conversions were determined by ^19^F NMR with reference to an internal standard.

**Scheme 2:**
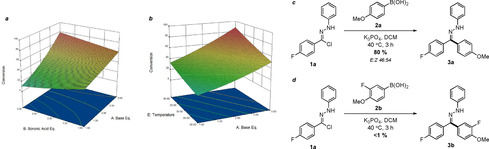
a) A 3D response surface outlining the dependence of boronic and base stoichiometry on reaction conversion, showing that an excess of base relative to the reaction partner is favoured. b) A 3D response surface highlighting the impact of temperature on the yield of procedure, with raised temperatures resulting in a higher conversion. c) The successful isolation of an 80 % yield of **3a** when employing the optimized reaction conditions. d) The introduction of electron‐deficient boronic acid substrates highlighted the drawbacks of the initially developed conditions.

A two‐level, five‐factor, half‐fractional Design of Experiments study^17^, ^18^ further implicated the importance of the role of the base. Increased stoichiometry of the base was found to significantly improve reaction conversion, particularly when present in greater proportions than the boronic acid (Scheme 2 a). Increased temperature was also found to be a major contributor to the overall conversion (Scheme 2 b). The reaction conditions developed through the application of this data afforded an 80 % yield of aryl hydrazone **3a** in 3 hours, requiring only 1.1 equivalents of boronic acid **2a** (Scheme 2 c).

Unfortunately, introduction of the more electron‐deficient boronic acid **2b** highlighted certain limitations of the protocol, with minimal conversion to the desired reaction product (Scheme 2 d). As a consequence, we sought to further modify the reaction conditions to increase compatibility with a broad scope of boronic acids. Due to the proven impact of temperature on reaction conversion (Scheme 2 b), alternative solvents with higher boiling points that had previously demonstrated adequate conversion were considered (Table 1, entries 4 and 5). The introduction of toluene as a direct replacement of DCM within the optimized conditions was found to be relatively straightforward, furnishing comparable conversions to hydrazone **3a** in only 1 hour (Table 2, entry 2). Furthermore, an immediate improvement was observed in the conversion of electron‐deficient substrate **3b**, with a 34 % conversion after heating at reflux for 2 hours (Table 2, entry 4). In parallel with our previous report,^8^ the introduction of dry conditions and increased boronic acid stoichiometry facilitated an improved 42 % turnover to the desired reaction product (Table 2, entry 7). These conditions were found to be directly comparable to those previously developed in the case of boronic acid **2a**, with a 75 % isolated yield of hydrazone **3a**.

**Table 2:** The introduction of toluene as a solvent and further optimization of electron‐deficient boronic acid substrates.^[a]^

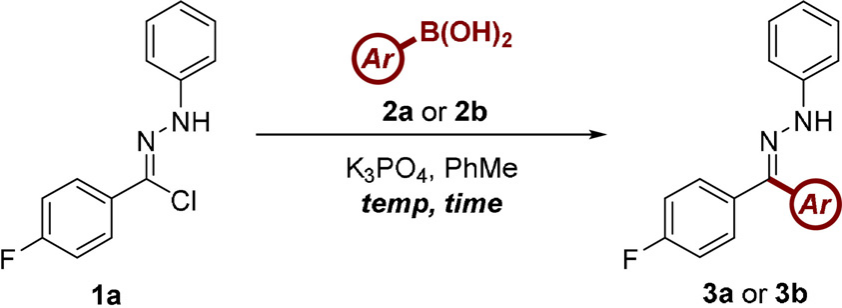				
Entry	ArB(OH)_2_ species	*t* [h]	*T* (°C)	Conv. [%]^[b]^
1	**2a**	3	50	75
2	**2a**	1	80	81
3	**2b**	2	80	17
4	**2b**	2	110	34
5^[c]^	**2b**	16	110	35
6^[c,d]^	**2b**	16	110	41
**7^[c,d,e]^**	**2b**	**16**	**110**	**42 (27^[f]^)**
8^[c,d,e]^	**2a**	16	110	75^[f]^

[a] Reactions performed on a 0.1 mmol scale using 1.1 equiv **2a/2b** and 3 equiv K_3_PO_4_ at a concentration of 0.1 m. [b] Conversions were determined by ^19^F NMR with reference to an internal standard. [c] N_2_ atmosphere. [d] 3Å mol. sieves (400 mg mmol^−1^) were added. [e] 2 equiv ArB(OH)_2_ were used. [f] Isolated yield.

In a concurrent approach towards exemplifying the scope of this procedure, and comparing the efficiency of the transformation to our previously reported conditions, the scope of the boronic acid component was explored by employing a similar palette of substrates. In most cases, this offered a direct comparison between the benefits of both methods of NI generation, with the results shown in Table 3.

**Table 3:** Investigation of the scope of aryl boronic acids compatible with the reaction conditions.^[a]^

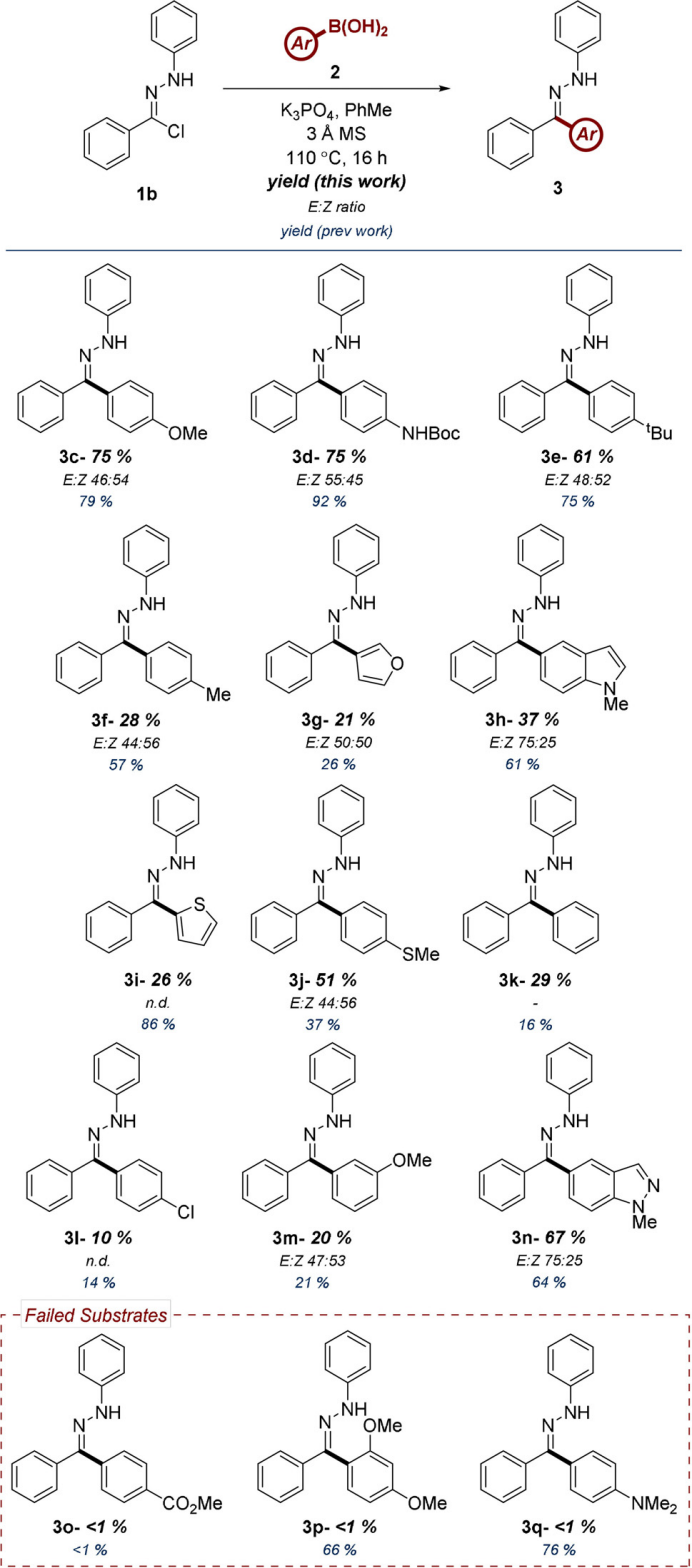

[a] Reactions performed on a 0.25 mmol scale using 2 equiv **2**, 3 equiv K_3_PO_4_, and 400 mg mmol^−1^ 3 Å mol. sieves at a concentration of 0.1 m.

The reaction conditions once again demonstrated an excellent compatibility with electron‐rich boronic acids, affording **3c**, **3d** and **3e** in good yield. In comparison to NI generation via tetrazole photolysis, the conversion levels observed are slightly lower. This correlation is exacerbated in the case of the moderately electron‐donating **3f** and heterocyclic analogues **3g**, **3h** and **3i**. This is understandable in the latter cases, when considering the limited stability of the corresponding boronic acids and the more forcing conditions necessitated by the modified approach. Having stated this, one advantage afforded by the hydrazonyl chloride NI source is the improved yields of electron‐neutral boronic acids (c.f. **3j** and **3k**). Electron‐deficient boronic acids **3l** and **3m** retained almost identical conversion values, despite the change in reaction conditions. The yield of indazole substrate **3n** was also largely unaffected by the alternative source of NI. Unfortunately, as was identified in our previous report, moderate to strongly electron‐deficient boronic acids failed to furnish the desired C−C bond (c.f. **3o**). The modified protocol was also shown to be more sensitive to steric occlusion, with the unanticipated negative result of *ortho*‐substituted substrate **3p**. The failure of substrate **3q** remains unexplained.

**Scheme 3:**
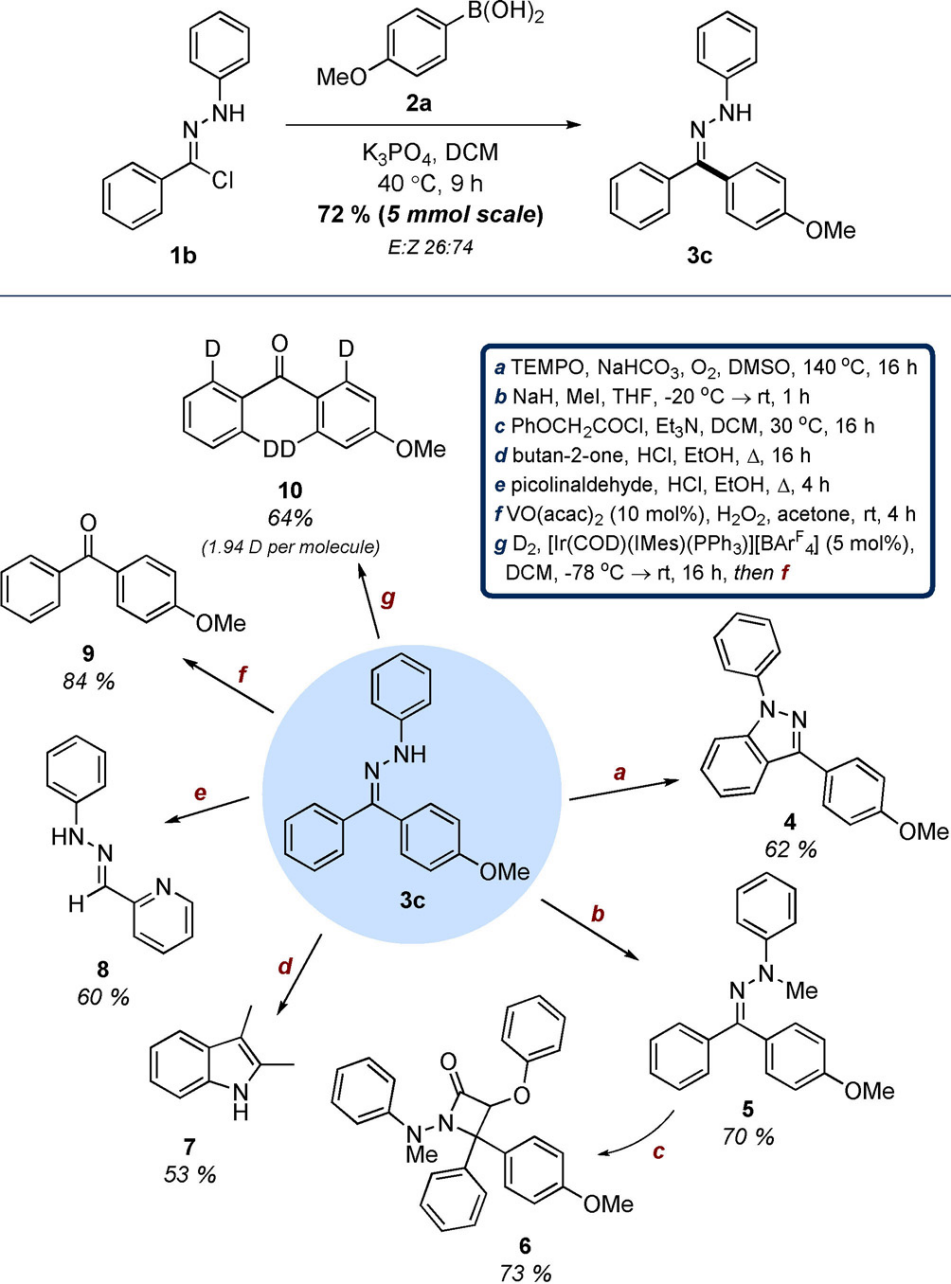
Applications of aryl hydrazone product **3c**.

While the reaction itself is believed to be stereospecific,^8^ the isolation of *E*:*Z* mixtures of the hydrazone was a consequence of thermal isomerization during the reaction and the presence of mildly acidic conditions during purification (column chromatography). The inability of the hydrazonyl chloride to improve these yields relative to the 2,5‐tetrazole as an NI source was an unexpected result. While the reversibility of dipole formation from this precursor should inhibit NI decomposition, it is possible that the reaction conditions may induce degradation of **1b** via an alternative pathway, such as through formation of the analogous carbonium ion.14b However, while it was established that the yields from different boronic acids afforded by the hydrazonyl chloride were largely unchanged in comparison to NI generation via a 2,5‐tetrazole, other advantages of the new procedure were evident. Repeating the synthesis of **3c** on a 5 mmol scale furnished the desired product in a comparable yield of 72 %. This scale would represent a significant challenge if employing the pre‐existing methodology, however the introduction of hydrazonyl chlorides as an NI source and negating the necessity of employing UV light greatly improves the practicality of the procedure. To further exemplify the benefits of the facile synthesis of **3c** on scale (5 mmol), the hydrazone was derivatized to afford a number of secondary and tertiary products (Scheme 3). This demonstrates the wide‐ranging applicability of this methodology in the efficient and transition‐metal‐free synthesis of diverse heterocyclic motifs (c.f. **4** and **6**), or in the introduction of masked aryl ketones and hydrazines (c. f. **8** and **9**). Of particular note is the synthesis of deuterated ketone **10**, where for the first time, an aryl hydrazone was employed as a directing group using a hydrogen isotope exchange (HIE) catalyst developed by Kerr.^19^ Site‐selective deuteration is achieved through iridium(III)‐promoted C−H activation.

To further exemplify the utility of NI generation from two complementary sources, we subsequently utilized hydrazonyl chloride precursors in the synthesis of a small library of substrates that would otherwise be inaccessible through tetrazole photolysis (Table 4). This was preceded by the efficient generation of hydrazone **3r**, which established the proof‐of‐concept that substitution of the NI precursor was well‐tolerated. Nitro‐substituted hydrazone **3s** and analogous derivatives **3t** and **3u** were all accessed in good to acceptable yields. These compounds represent examples of substrates incompatible with the route previously developed in our laboratory, due to the very limited photochemical reactivity of the relevant nitro‐substituted tetrazole precursors.4i, 20 Alkyl substitution of the NI, which was similarly unsuited to the initial UV‐promoted protocol, was also accomplished (c.f. **3v** and **3w**). While increased steric bulk of the *C*‐terminus was shown to be relatively inconsequential, the overall yields of these analogues were generally low. This is thought to be attributable to the truncated π‐system of the corresponding NI increasing the reactivity of the intermediate, leading to unwanted side‐reactions. A similar phenomenon is likely responsible for the failure of substrates **3x** and **3y**. While diversification of the NI N‐terminus is an attractive prospect from a synthetic perspective, the significant role of the *N*‐aryl ring in stabilising the intermediate likely further accelerates decomposition in these substrates.^21^

**Table 4:** Synthesis of hydrazone systems containing aryl‐nitro and alkyl substituents.^[a]^

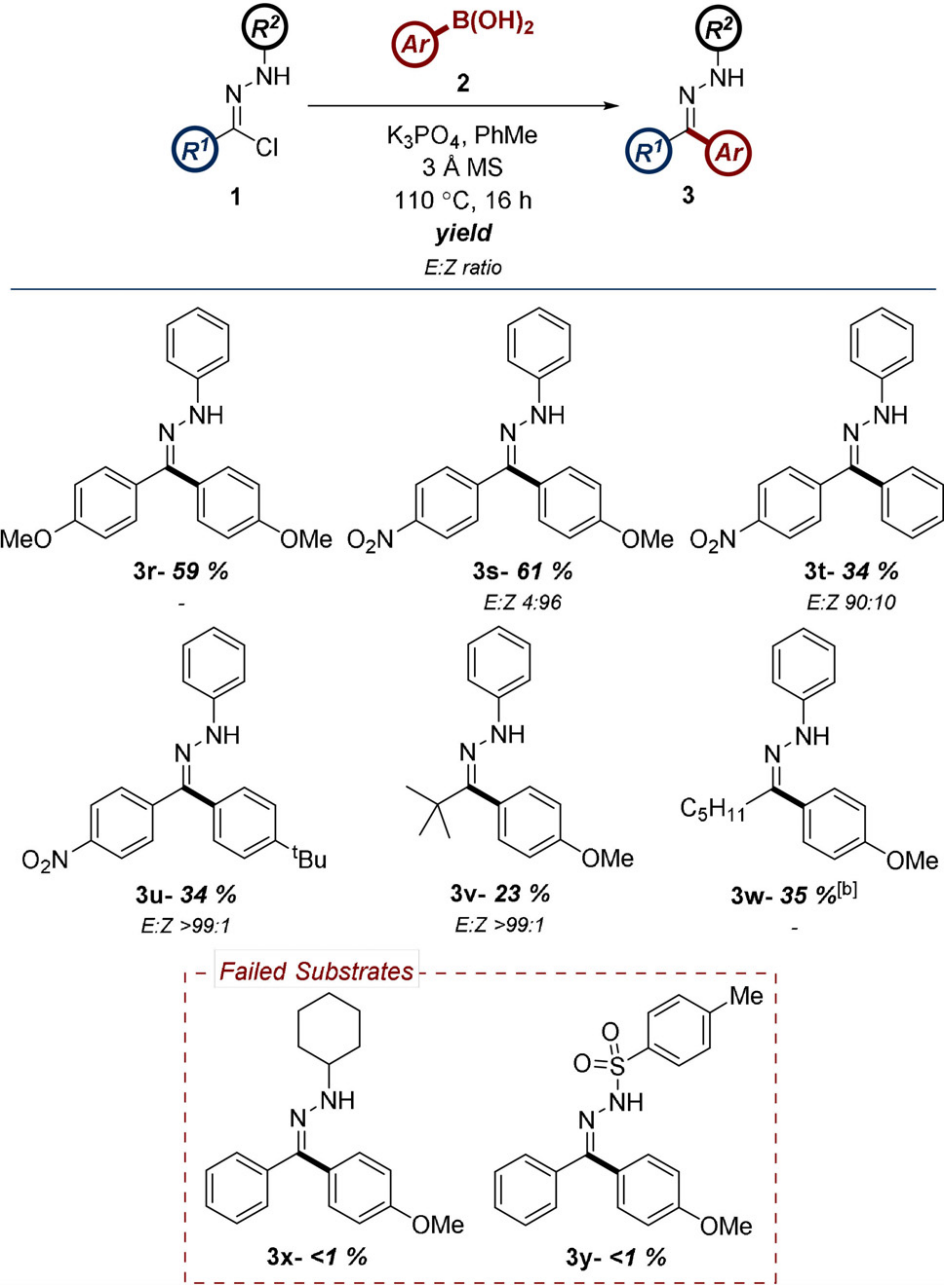

[a] Reactions performed on a 0.25 mmol scale using 2 equiv **2**, 3 equiv K_3_PO_4_, and 400 mg mmol^−1^ 3 Å mol. sieves at a concentration of 0.1 m. [b] Isolated as the corresponding ketone.

One of the principal benefits of the development of transition‐metal‐free C−C bond formation using hydrazonyl chlorides as an NI source is the potential applicability of the method towards other 1,3‐dipole systems. In particular, the nitrile oxide (NO) 1,3‐dipole can be readily accessed from the closely related hydroxamoyl chloride moiety.^22^ Accordingly, we reasoned that appropriate adaption of the reaction conditions developed in the current study could furnish an analogous protocol for C−C bond formation from boronic acids and NOs.

Initial efforts using a similar base and solvent combination as described above failed to furnish the desired ketone oxime from hydroxamoyl chloride **11a**, despite application of the more reactive boronic acid **2a** (Table 5, entry 1). Indeed, the NO 1,3‐dipole proved significantly more capricious in its reactivity in comparison to the analogous NI (see SI for further details), with only two bases found to afford the product **12a** in an appreciable yield (Table 5, entries 2 and 3). *N*,*N*’‐Dimethylaniline (DMA) was eventually identified as an appropriate choice of base. The notably lower p*K*_a_ of this compound in comparison to the bases evaluated as part of the NI screen further emphasizes the elevated reactivity of the NO species.

**Table 5:** The optimization of reaction conditions for the metal‐free C−C bond formation between nitrile oxides and aryl boronic acids.^[a]^

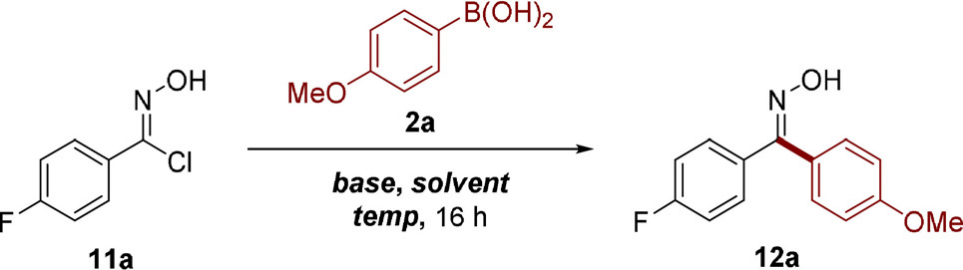				
Entry	Base	Solvent	*T* [°C]	Conv. [%]^[b]^
1^[c]^	K_3_PO_4_	DCM	25	<1
2^[c]^	Cs_2_CO_3_	DCM	25	17
3	DMA	DCM	25	43
4	DMA	CHCl_3_	25	37
5	DMA	CHCl_3_	60	63
**6^[d,e]^**	**DMA**	**CHCl_3_**	**60**	**70 (62^[f]^)**

[a] Reactions performed on a 0.1 mmol scale using 1.5 equiv **2a** and 3 equiv base at a concentration of 0.1 m. [b] Conversions were determined by ^19^F NMR with reference to an internal standard. [c] 3 equiv **2a** and 5 equiv base. [d] 2 equiv **2a** and 5 equiv base. [e] 3 hours reaction time. [f] Isolated yield.

While direct application of the reaction conditions identified for the NI dipole was not possible, the conditions developed for the corresponding NO species nevertheless exhibited similar characteristics, with a dependence on base stoichiometry and elevated temperatures. The identification of chloroform as an alternative solvent (Table 5, entry 4) enabled operation at elevated temperatures, ultimately furnishing a 62 % isolated yield of oxime **12a** with an unprotracted reaction time (Table 5, entries 5 and 6).

With optimized conditions in hand, a small selection of boronic acids were reacted with phenyl hydroxamoyl chloride **11b** in order to further delineate the scope of the reaction (Table 6). Particular attention was invested in the comparison of these results with those generated when employing NI precursor **1b**. In general, the results obtained from this part of the study were very similar to the isolated yields found in Table 3. Electron‐rich boronic acids were again shown to possess the most favourable properties within the protocol, with oximes **12b** and **12c** isolated in 68 and 71 % yields, respectively. The noted decrease in yield observed for **12d** is consistent with the reduced inductive influence of the *para*‐methyl group. Substrates **12e** to **12h** highlight the comparability of NO and NI reactivity, as all four oximes isolated under these conditions were furnished in very similar yield in comparison to the corresponding NI substrate. One interesting difference when employing the NO 1,3‐dipole is the compatibility of the *N*,*N’*‐dimethylaniline moiety. While hydrazone **3q** was not successfully isolated, oxime **12i** was obtained in moderate yield. The procedure was, however, found to be incompatible with *ortho*‐substitution of the boronic acid (c.f. **12j**), with the synthesis of electron‐deficient substrate **12k** also unsuccessful. The increased stereoselectivity of oximes **12** relative to hydrazones **3** is likely symptomatic of the increased hydrolytic stability of oximes under mildly acidic conditions, such as silica chromatography,^23^ in addition to the lower reaction temperature limiting thermal isomerization.

**Table 6:** Investigation of the scope of aryl ketone oximes accessible from hydroxamoyl chloride **11b**.^[a]^

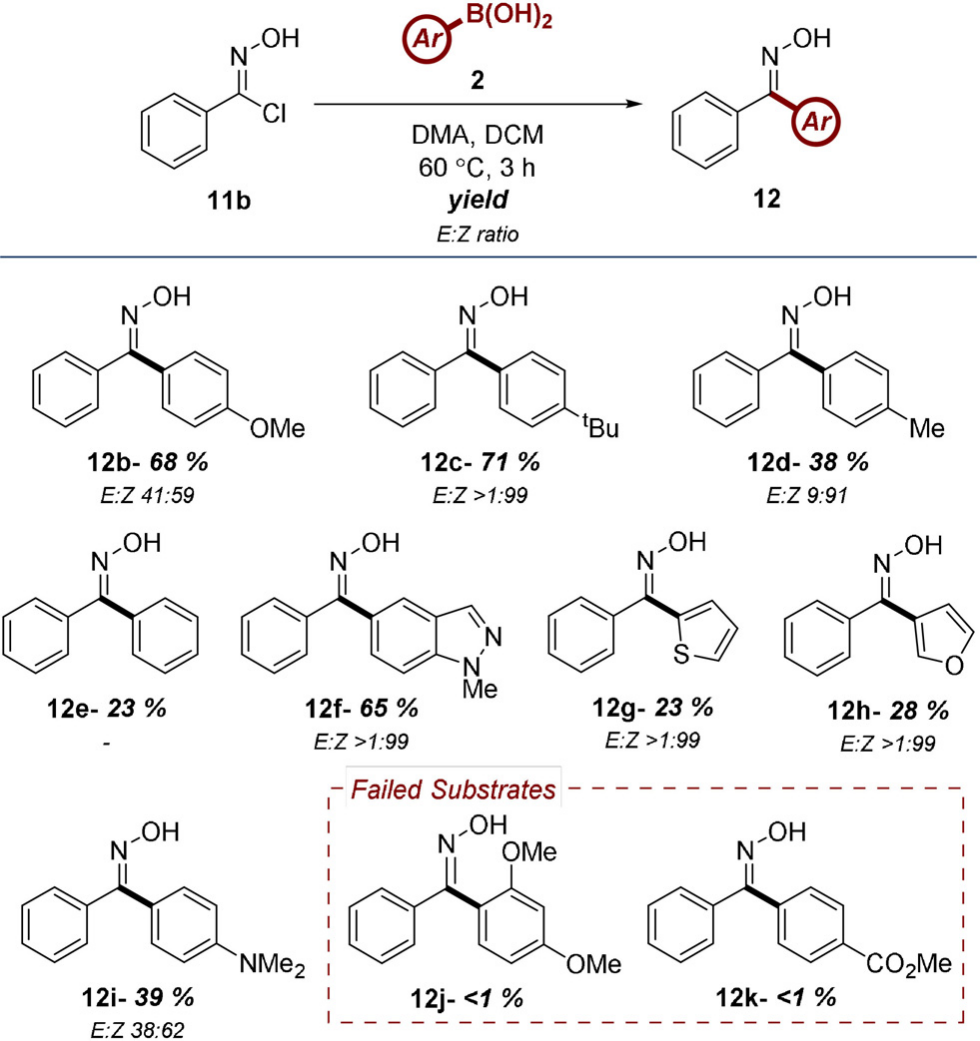

[a] Reactions performed on a 0.25 mmol scale using 2 equiv **2** and 3 equiv DMA at a concentration of 0.1 m.

**Scheme 4:**
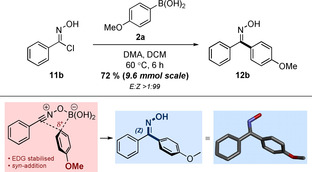
The summation of mechanistic evidence indicates an intramolecular, facially selective aryl group migration.

As a final demonstration of its utility, the nitrile oxide procedure was also shown to be compatible on large scale, with 1.5 g of **11b** smoothly converted to oxime **12b** in a 72 % yield (Scheme 4). Expedient purification of this sample afforded the desired compound with complete *Z*‐stereoselectivity, providing evidence that the isomerization of **12b** that was previously observed (Table 6) was a consequence of extended exposure to silica during column chromatography, rather than during the transformation itself. An X‐ray crystal structure of **12b** also added weight to our previous mechanistic proposal,^8^ indicating that aryl migration occurs via an intramolecular process, following prior coordination of the boronic acid moiety to the anionic terminus of the NO. This C−C bond formation appears to proceed with complete *syn*‐selectivity, relative to the oxime directing group. Given the similarity between the properties of the dipoles, it is probable that this facial selectivity is also implicated when employing the NI, although further studies are required to corroborate this hypothesis.

## Conclusions

In summary, we have comprehensively investigated the application of NIs and boronic acids in the metal‐free synthesis of C−C bonds. This expanded report greatly enhances the utility of the protocol following on from our initial communication. Introduction of the hydrazonyl chloride as an alternative and complementary source of the NI 1,3‐dipole can achieve comparable yields of aryl ketone hydrazones with respect to 2,5‐tetrazole photolysis, while negating some of the practical shortcomings associated with UV light. The protocol was shown to operate effectively on a larger scale, serving as a feedstock for a diverse array of further transformations. A number of hydrazones previously inaccessible through our earlier conditions were also furnished in moderate to good yields by adopting this methodology, providing a route towards alkyl hydrazones through metal‐free C−C bond formation. Furthermore, mechanistic translation of the procedure into the related NO 1,3‐dipole family was accomplished via a relatively straightforward optimization campaign, with exemplification of the protocol through a substrate scope highlighting the substantial similarities in reactivity between the NI and NO dipoles. We anticipate that this transformation may serve as a facile alternative towards traditional C−C bond formation, while simultaneously aiding in furthering the understanding of the complex reactivity profiles of NIs and related 1,3‐dipoles.

## Experimental Section

### General procedure for the synthesis of ketone hydrazones 3

To an oven‐dried 5 mL microwave vial was added 3 Å molecular sieves (400 mg mmol^−1^), hydrazonyl chloride (1 equiv), and boronic acid (2 equiv). The mixture was dissolved in toluene (0.1 m), and K_3_PO_4_ (3 equiv) was added to initiate the reaction. The solution was purged with N_2_, and heated at 110 °C for 16 h. The reaction mixture was diluted with ethyl acetate, filtered through Celite and rinsed with additional ethyl acetate. The crude solution was concentrated under vacuum and purified by column chromatography.

### General procedure for the synthesis of aryl ketone oximes 12

To an oven‐dried 5 mL microwave vial was added hydroxamoyl chloride (1 equiv) and boronic acid (2 equiv). The mixture was dissolved in chloroform (0.1 m), and *N*,*N*’‐dimethylaniline (5 equiv) was added to initiate the reaction. The solution was purged with N_2_, and heated at 60 °C for 3 h. The reaction mixture was diluted with DCM, and washed with 1 m HCl solution. The organic phase was separated, washed with brine, passed through a phase separator and concentrated under vacuum. The crude residue was purified by column chromatography.

## Conflict of interest

The authors declare no conflict of interest.

## Supporting information

As a service to our authors and readers, this journal provides supporting information supplied by the authors. Such materials are peer reviewed and may be re‐organized for online delivery, but are not copy‐edited or typeset. Technical support issues arising from supporting information (other than missing files) should be addressed to the authors.

Supplementary
