# Supplementary material for: Transition‐Metal‐Free Coupling of 1,3‐Dipoles and Boronic Acids as a Sustainable Approach to C−C Bond Formation
Source: Chemistry. 2020 Jul 21;26(46):10591–7. doi: 10.1002/chem.202001590 (PMC7496359; doi:10.1002/chem.202001590)

Chemistry–A European Journal

Supporting Information

Transition-Metal-Free Coupling of 1,3-Dipoles and Boronic Acids as a Sustainable Approach to C–C Bond Formation

Keith Livingstone,[a] Sophie Bertrand,[b] Alan R. Kennedy,[a] and Craig Jamieson*[a]

Table of Contents

1 General Methods 1

1.1 Purification of Solvents and Reagents 1

1.2 Experimental Details 1

1.3 Purification of Products 1

1.4 Analysis of Products 1

2 General Procedures 2

3 Optimisation of Reaction Conditions 4

3.1 Hydrazonyl Chloride ^{19}F NMR Assay 4

General Optimisation Conditions 4

Solvent Screen 4

Base Screen 5

Counterion Screen 6

Design of Experiments Study[5] 7

Introduction of Toluene as an Alternative Solvent 9

Electron-deficient Boronic Acid Substrates 9

3.2 Hydroxamoyl Chloride ^{19}F NMR Assay 10

General Optimisation Conditions for Hydroxamoyl Chloride ^{19}F NMR Assay 10

Base Screen 10

Screen of Base and Boronic Acid Stoichiometry 1 11

Solvent Screen 11

Screen of Base and Boronic Acid Stoichiometry 2 12

4 Characterisation of Compounds 13

4.1 Starting Materials 13

4.2 Ketone Hydrazones 20

4.3 Derivatization of 3c 30

4.4 Ketone Oximes 35

5 X-Ray Crystallography 40

6 References 41

7 NMR Spectra of Compounds 42

1 General Methods

1.1 Purification of Solvents and Reagents

Anhydrous DCM and toluene were obtained from a PureSolv SPS-400-5 solvent purification system. Solvents were transferred to and stored in septum-sealed oven-dried flasks over activated 4 Å molecular sieves and purged with and stored under nitrogen. Acetone, dichloromethane, ethyl acetate, methanol, petroleum ether 40–60 °C, and tetrahydrofuran were used as obtained from suppliers without further purification. K_3PO_4 was stored in a vacuum oven at 60 °C.

1.2 Experimental Details

All reactions were performed using round-bottom flasks, microwave vials or HPLC vials of appropriate volume. Reactions were carried out at elevated temperatures using a temperature-regulated hotplate/stirrer. Room temperature generally refers to ~ 20 °C. Reactions requiring a reduced temperature were performed using an ice bath (0 °C) or a dry ice/actone slurry (-78 °C) and a temperature probe unless otherwise stated. Phase separation was conducted using IST Isolute Phase Separator Cartridges.

1.3 Purification of Products

Thin layer chromatography was carried out using Merck silica plates coated with fluorescent indicator UV254. These were analysed under 254 nm UV light or developed using potassium permanganate solution. Flash chromatography was carried out using ZEOprep 60 HYD 40-63 µm silica gel.

1.4 Analysis of Products

Fourier Transformed Infra-Red (FTIR) spectra were obtained using an A2 Technologies ATR 32 machine. ^{1}H, ^{19}F and ^{13}C NMR spectra were obtained on a Bruker DRX 500 spectrometer at 500, 376 and 126 MHz, respectively or on a Bruker AV3 400 at 400, 471 and 101 MHz, respectively, or on a Bruker AVANCE 400 spectrometer at 400, 471 and 101 MHz, respectively. Chemical shifts are reported in ppm and coupling constants are reported in Hz with $CDCl_3$ referenced at 7.26 (^{1}H) and 77.16 ppm (^{13}C), DMSO-d_6 referenced at 2.50 (^{1}H) and 39.52 ppm (^{13}C), and acetone-d_6 referenced at 2.05 (^{1}H) and 29.84 ppm (^{13}C). High-resolution mass spectra were obtained on a Thermofisher LTQ Orbitrap XL instrument at the EPSRC National Mass Spectrometry Service Centre (NMSSC), Swansea.

2 General Procedures

General Procedure A: Synthesis of Aldehyde Hydrazones

Phenyl hydrazine hydrochloride (1 equiv.) was washed with 1M NaOH solution to generate the free phenyl hydrazine. This compound was dissolved and minimal EtOH and added dropwise to a solution of the relevant aryl aldehyde (1 equiv.) in EtOH (2.5 M). A few drops of H_2SO_4 were added to accelerate the reaction. The solution was stirred until consumption of the aldehyde starting material, before the mixture was filtered to isolate the product as a precipitate, which was used in the following steps without further purification. In the circumstances where the product would fail to precipitate, the reaction mixture was concentrated to dryness, and purified by column chromatography.

General Procedure B: Chlorination of Aldehyde Hydrazones

In accordance with previous literature precedent,[1] to an oven-dried flask under nitrogen was added *N*-chlorosuccinimde (1.7 equiv.) in DCM (1 M). Dimethylsulfide (3 equiv.) was added to the solution dropwise at 0 °C, maintaining a temperature of below 10 °C at all times. The reaction mixture was stirred for an additional 15 minutes, and then cooled to -78 °C. A solution of the relevant hydrazone (1 equiv.) was dissolved in minimal DCM and added dropwise to the reaction mixture. Stirring was maintained at -78 °C for 2 hours before being raised to room temperature. Following consumption of the hydrazone starting material, the mixture was further diluted by DCM and washed successively with H_2O and brine. The solution was passed through a phase separator, concentrated under vacuum and purified by column chromatography.

General Procedure C: Synthesis of Alkyl Hydrazides

To a solution of the relevant acid chloride (1 equiv.) and pyridine (10 equiv.) in DCM (1M) was added phenyl hydrazine (5 equiv.) at 0 °C. The reaction mixture was left to stir overnight at room temperature, before it was quenched using H_2O. The mixture was partitioned between DCM and H_2O, and organic layer was washed successively with a further portion of H_2O and brine. The solution was passed through a phase separator, concentrated under vacuum and purified by column chromatography.

General Procedure D: Chlorination of Alkyl Hydrazides

In accordance with previous literature precedent,[2] to a solution of triphenylphosphine (1.2 equiv.) in MeCN (0.7 M) was added the relevant alkyl hydrazide (1 equiv.) and carbon tetrachloride (1.3 equiv.) at room temperature. The solution was stirred at this temperature overnight, before the reaction mixture was concentrated under vacuum. The resulting crude material was purified by column chromatography.

General Procedure E: Synthesis of Hydroxamoyl Chlorides

In accordance with previous literature precedent,[3] a solution of the relevant aldehyde (1 equiv.), hydroxylamine hydrochloride (2 equiv.), and sodium hydroxide (2 equiv.) in ethanol (0.8 M) was refluxed for 2 hours. The suspension was filtered, and the solid was washed with a further portion of ethanol. The filtrate was concentrated under vacuum and the residue dissolved in ethyl acetate. The organic layer was washed with water and brine, passed through a hydrophobic frit and concentrated under vacuum. The crude hydrazone was dissolved in DMF (0.7 M) and *N*-chlorosuccinimide (1.1 equiv.) was added in 8-10 portions to maintain the temperature below 40 °C. The reaction mixture was stirred at room temperature for 6 hours, when it was diluted with ethyl acetate. The organic layer was sequentially washed with 5% lithium chloride solution, water, and brine, passed through a hydrophobic frit and concentrated under reduced pressure. The obtained residue was purified by column chromatography.

General Procedure F: Base-Mediated Coupling of Hydrazonyl Chlorides and Aryl Boronic Acids

To an oven-dried 2-5 mL microwave vial was added 3 Å molecular sieves (400 $mgmmol^{-1}$), hydrazonyl chloride (1 equiv.), and boronic acid (2 equiv.). The mixture was dissolved in toluene (0.1 M), and K_3PO_4 (3 equiv.) was added to initiate the reaction. The solution was purged with N_2, and heated at 110 °C for 16 h. The reaction mixture was diluted with ethyl acetate, filtered through Celite and rinsed with additional ethyl acetate. The crude solution was concentrated under vacuum and purified by column chromatography.

General Procedure G: Base-Mediated Coupling of Hydroxamoyl Chlorides and Aryl Boronic Acids

To an oven-dried 5 mL microwave vial was added hydroxamoyl chloride (1 equiv.) and boronic acid (2 equiv.). The mixture was dissolved in chloroform (0.1 M), and *N*,*N*'-dimethylaniline (5 equiv.) was added to initiate the reaction. The solution was purged with N_2, and heated at 60 °C for 3 h. The reaction mixture was diluted with DCM, and washed with 1 M HCl solution. The organic phase was separated, washed with brine, passed through a phase separator and concentrated under vacuum. The crude residue was purified by column chromatography.

3 Optimisation of Reaction Conditions

3.1 Hydrazonyl Chloride ^{19}F NMR Assay

General Optimisation Conditions

Hydrazonyl chloride **1a** (24.9 mg, 0.1 mmol) and 4-methoxyphenyl boronic acid (**2a**, 15.2-45.6 mg, 0.1-0.3 mmol) were added to the appropriate solvent (1-10 mL). Base (0.1-0.3 mmol) was added and the mixture was stirred at a specified temperature for the indicated period of time. Following reaction completion, a solution of compound **S1**[4] (21.6 mg, 0.1 mmol) in DCM (0.5 mL) was added to the reaction mixture. When using an inorganic base, the mixture was then filtered through celite to remove residual solids. The solution was spiked with $CDCl_3$ and analysed by ^{19}F NMR spectroscopy. Product peaks at -112.31 and -115.01 ppm were integrated against the peak of reaction standard compound **S1** at -132.21 ppm.

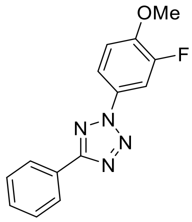

S1

*Figure S 1: The structure of tetrazole **S1***

Solvent Screen

Table S 1: Selection of the optimal reaction solvent in the generation of hydrazones from hydrazonyl chlorides[a]

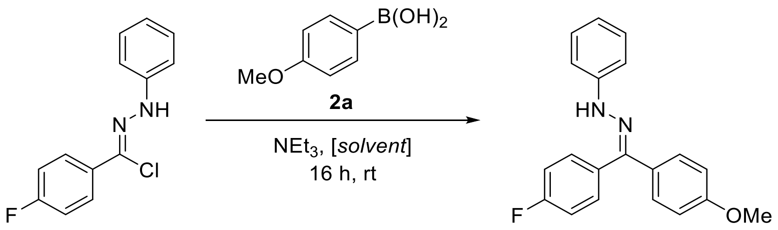

1a **3a**

Entry	Solvent	Conversion (%)[b,c]
1	MeCN	**32[d]**
2	THF	**22**
3	DCM	**75**
4	Et_2O	**15**
5	MeOH	**<1[d]**
6	^{i}PrOH	**<1[d]**
7	1,4-dioxane	**58**

8	DMSO	**<1[d]**
9	EtOAc	**48**
10	DMF	**<1**
11	2-MeTHF	**26**
12	PhMe	**50**
13	$COMe_2$	**47[d]**
14	H_2O	**13**
15	DCE	**40**
16	$CHCl_3$	**51**
17	CPME	**20**

[a]Reactions performed using 3 equiv. **2a** and 3 equiv. Et_3N, at a concentration of 0.1 M. [b]Conversion determined by ^{19}F NMR with reference to an internal standard. [c]Average value of two experiments with a difference of <10 %. [d]Significant by-product formation observed.

Base Screen

Table S 2: Selection of the optimal base in the generation of hydrazones from hydrazonyl chlorides[a]

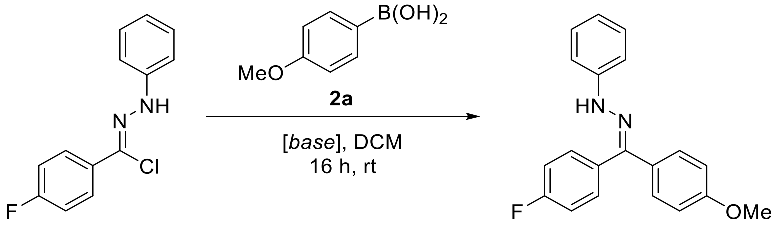

1a **3a**

Entry	Base	Conversion (%)[b,c]
1	KTFA	**<1**
2	KOAc	**<1**
3	Pyridine	**<1**
4	Lutidine	**2**
5	NMM	**8**
6	Collidine	**10**
7	DABCO	**<1[d]**
8	K_2CO_3	**20**
9	NEt_3	**53**
10	DIPEA	**55**
11	DBU	**9[d]**
12	K_3PO_4	**71**

13	KF	**37**
14	KOH	**7**[d]
15	KO^{t}Bu	**15**[d]
16	BEMP	**6**[d]

[a]Reactions performed using 1.1 equiv. **2a** and 3 equiv. base, at a concentration of 0.1 M. [b]Conversion determined by ^{19}F NMR with reference to an internal standard. [c]Average value of two experiments with a difference of <10 %. [d]Significant by-product formation observed.

Counterion Screen

Table S 3: Selection of the optimal base and counterion in the generation of hydrazones from hydrazonyl chlorides[a]

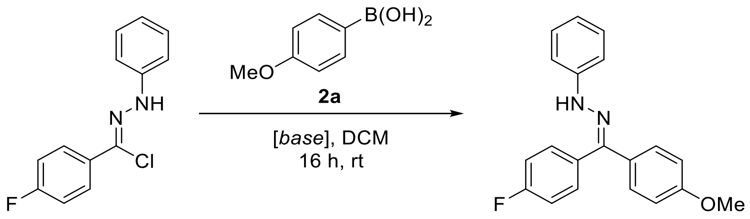

1a **3a**

Entry	Base	Conversion (%)[b,c]
1	$(NH_4)_2CO_3$	**<1**
2	Na_2CO_3	**<1**
3	$CaCO_3$	**<1**
4	K_2CO_3	**20**
5	Cs_2CO_3	**55**
6	KH_2PO_4	**<1**
7	K_2HPO_4	**<1**
8	K_3PO_4	**70**
9	Na_3PO_4	**3**
10	$Ca_3(PO_4)_2$	**<1**
11	$Mg_3(PO)_2$	**<1**
12[d]	K_3PO_4	**60**[e]

[a]Reactions performed using 1.1 equiv. **2a** and 3 equiv. base at a concentration of 0.1 M. [b]Conversion determined by ^{19}F NMR with reference to an internal standard. [c]Average value of two experiments with a difference of <10 %. [d]$CHCl_3$ was used as the reaction solvent. [e]Significant by-product formation observed.

Design of Experiments Study[5]

*Table S 4: The raw data obtained in the Design of Experiments study into the formation of hydrazone **3a***

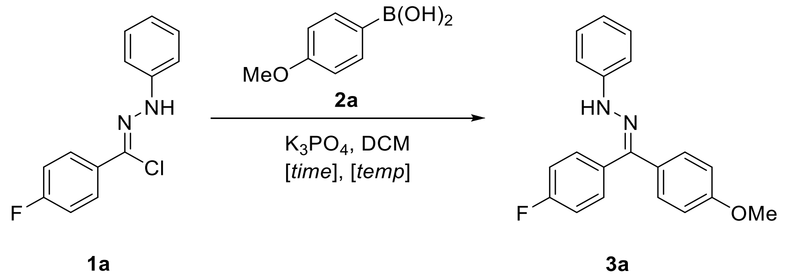

Entry	Base Eq	Boronic Acid Eq	Concentration (M)	Time (h)	Temperature (°C)	Conversion (%)a
1	2	2	0.06	9.5	30	**71**
2	2	2	0.06	9.5	30	**60**
3	2	2	0.06	9.5	30	**69**
4	2	2	0.06	9.5	30	**61**
5	1	1	0.01	16	20	**28**
6	1	1	0.01	16	20	**20**
7	1	1	0.01	16	20	**28**
8	1	1	0.1	16	40	**45**
9	1	1	0.1	16	40	**48**
10	1	1	0.1	16	40	**51**
11	1	1	0.1	3	20	**24**
12	1	1	0.1	3	20	**21**
13	1	1	0.1	3	20	**24**
14	1	1	0.01	3	40	**34**
15	1	1	0.01	3	40	**35**
16	1	1	0.01	3	40	**32**
17	1	3	0.1	16	20	**2**
18	1	3	0.1	16	20	**2**
19	1	3	0.1	16	20	**1**
20	1	3	0.01	16	40	**22**
21	1	3	0.01	16	40	**22**
22	1	3	0.01	16	40	**23**
23	1	3	0.01	3	20	**1**
24	1	3	0.01	3	20	**1**
25	1	3	0.01	3	20	**2**
26	1	3	0.1	3	40	**13**
27	1	3	0.1	3	40	**18**
28	1	3	0.1	3	40	**25**

27	3	1	0.1	16	20	**64**
28	3	1	0.1	16	20	**59**
29	3	1	0.1	16	20	**67**
30	3	1	0.01	16	40	**85**
31	3	1	0.01	16	40	**81**
32	3	1	0.01	16	40	**80**
33	3	1	0.01	3	20	**12**
34	3	1	0.01	3	20	**12**
35	3	1	0.01	3	20	**13**
36	3	1	0.1	3	40	**79**
37	3	1	0.1	3	40	**75**
38	3	1	0.1	3	40	**80**
39	3	3	0.01	16	20	**61**
40	3	3	0.01	16	20	**64**
41	3	3	0.01	16	20	**65**
42	3	3	0.1	16	40	**84**
43	3	3	0.1	16	40	**80**
44	3	3	0.1	16	40	**82**
45	3	3	0.1	3	20	**62**
46	3	3	0.1	3	20	**53**
47	3	3	0.10	3	20	**66**
48	3	3	0.01	3	40	**67**
49	3	3	0.01	3	40	**76**
50	3	3	0.01	3	40	**82**

[a] Conversion determined by ^{19}F NMR with reference to an internal standard

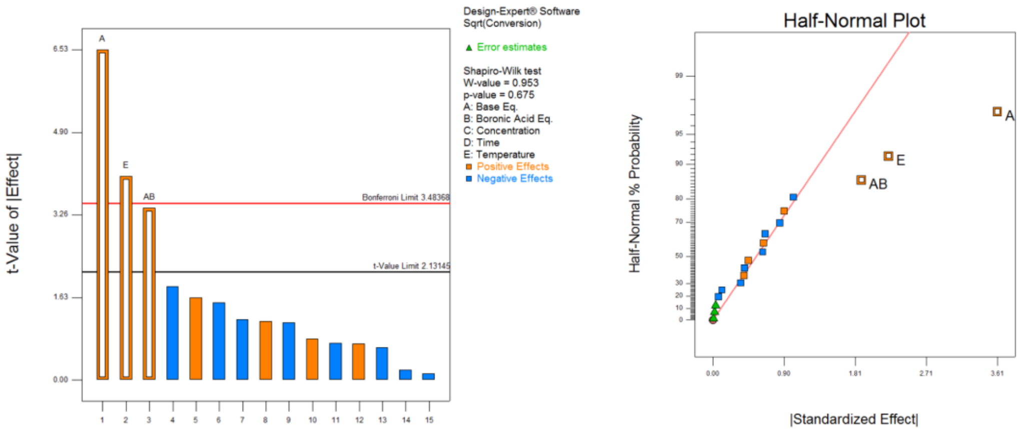

Figure S 2: The t-Values and Half-Normal Plot of the Design of Experiments study, highlighting the significant impact of base stoichiometry (A) and temperature (E)

Introduction of Toluene as an Alternative Solvent

Table S 5: The impact of introducing toluene and shortening the reaction time on the formation of ***3a****.*[a]

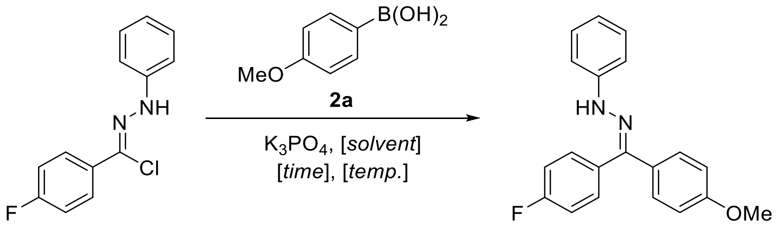

1a **3a**

Entry	Solvent	Temperature (°C)	Time (h)	Conversion (%)[b,c]	Isolated Yield (%)
1	DCM	40	3	**85**	**80**
2	DCM	40	16	**86**	**85**
3	PhMe	50	3	**75**	**69**
4	PhMe	80	1	**81**	**75**

[a]Reactions performed using 1.1 equiv. **2a** and 3 equiv. K_3PO_4, at a concentration of 0.1 M. [b]Conversion determined by ^{19}F NMR with reference to an internal standard. [c]Average value of two experiments with a difference of <10 %.

Electron-deficient Boronic Acid Substrates

Table S 6: Modification of the reaction conditions to accommodate electron-deficient substrate ***2b***[a]

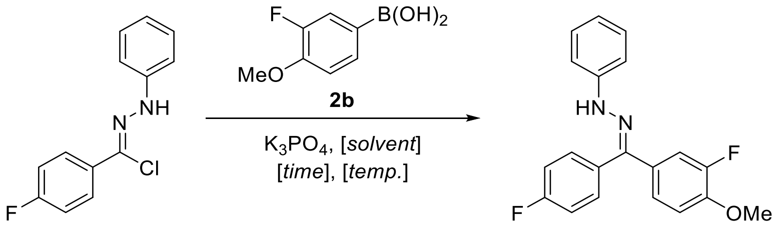

1a **3b**

Entry	Temperature (°C)	Time (h)	$B(OH)_2$ Stoichiometry (equivs)	Conversion (%)[b,c]
1	60	2	1.1	**2**
2	80	2	1.1	**17**
3	110	2	1.1	**34**
4	60	2	2	**3**
5	110	2	2	**24**
6[d,e]	80	2	1.1	**4**
7[d,e]	80	2	2	**2**
8[d,e]	110	2	2	**30**
9	110	16	1.1	**23**
10[d,e]	110	16	1.1	**41**

11[d,e]	110	16	2	**42 (27[f])**
12[d,e]	110	16	3	**48**
13[d]	110	16	1.1	**35**

[a]Reactions performed using 1.1 equiv. **2a** and 3 equiv. K_3PO_4, at a concentration of 0.1 M. [b]Conversion determined by ^{19}F NMR with reference to an internal standard. [c]Average value of two experiments with a difference of <10 %. [d]Performed under an N_2 atmosphere. [e]3 Å molecular sieves were added. [f]Isolated yield.

3.2 Hydroxamoyl Chloride ^{19}F NMR Assay

General Optimisation Conditions for Hydroxamoyl Chloride ^{19}F NMR Assay

Hydroxamoyl chloride **11a** (174. mg, 0.1 mmol) and 4-methoxyphenylboronic acid (**2a**, 22.8-45.6 mg, 0.15-0.3 mmol) were dissolved in a specified solvent (1 mL). Base (0.3-0.5 mmol) was added, and the reaction mixture was stirred at the specified temperature for the indicated period of time. Following reaction completion, a solution of 1,4-difluorobenzene (0.75 M in $CDCl_3$) was added to the reaction mixture. When using an inorganic base, the mixture was then filtered through celite to remove residual solids. The solution was analysed by ^{19}F NMR spectroscopy. Product peaks at -111.76 and -111.94 ppm were integrated against the peak of reaction standard 1,4-difluorobenzene at -119.63 ppm.

Base Screen

Table S 7: Selection of the optimal base in the generation of oximes from hydroxamoyl chlorides[a]

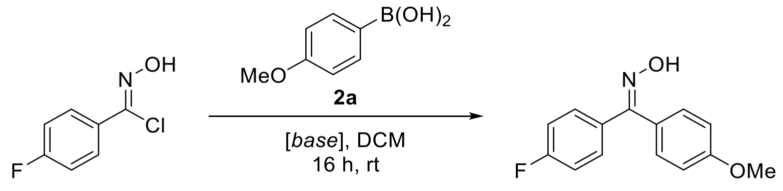

11a **12a**

Entry	Base	Conversion (%)[b]
1	KH_2PO_4	**<1**
2	K_2HPO_4	**<1**
3	K_3PO_4	**<1**
4	$NaHCO_3$	**<1**
5	Cs_2CO_3	**17**
6	NEt_3	**<1**
7	Pyridine	**<1[c]**
8	DBU	**<1[c]**
9	DMA (*N*,*N*'-Dimethylaniline)	**58**

[a]Reactions performed using 3 equiv. **2a** and 5 equiv. base, at a concentration of 0.1 M. [b]Conversion determined by ^{19}F NMR with reference to an internal standard. [c]Significant by-product formation observed.

Screen of Base and Boronic Acid Stoichiometry 1

Table S 8: Investigation of the impact of reagent stoichiometry in the generation of oximes from hydroxamoyl chlorides[a]

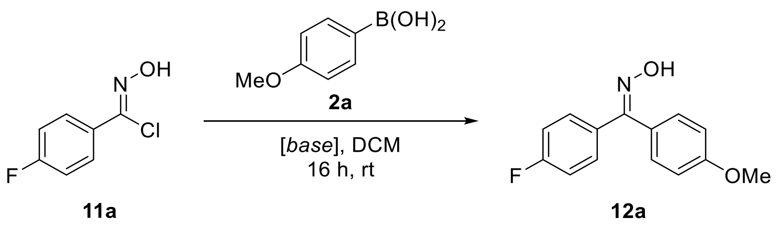

Entry	Base	Base Stoichiometry (equiv.)	Boronic Acid Stoichiometry (equiv.)	Conversion (%)[b]
1	Cs_2CO_3	5	3	**17**
2	Cs_2CO_3	3	3	**12**
3	Cs_2CO_3	3	1.5	**27**
4	Cs_2CO_3	1.5	1.5	**30**
5	DMA	5	3	**58**
6	DMA	3	3	**54**
7	DMA	3	1.5	**43**
8	DMA	1.5	1.5	**37**

[a]Reactions performed at a concentration of 0.1 M. [b]Conversion determined by ^{19}F NMR with reference to an internal standard.

Solvent Screen

Table S 9: Selection of the optimal solvent in the generation of oximes from hydroxamoyl chlorides[a]

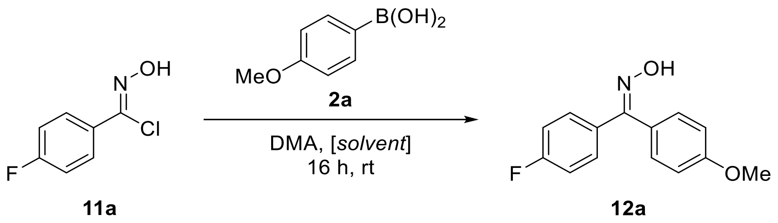

Entry	Solvent	Conversion (%)[b]
1	DCM	**43**
2	Toluene	**<1**
3	1,4-Dioxane	**<1**
4	THF	**<1**
5	MeCN	**37**
6	Acetone	**12**
7	$CHCl_3$	**37**

8	2-MeTHF	**<1**
9	EtOAc	**3**
10	MeOH	**7**
11	DCM[c]	**52**
12	$CHCl_3$[d]	**63**
13	MeCN[e]	**57**

[a]Reactions performed using 1.5 equiv. **2a** and 3 equiv. base, at a concentration of 0.1 M. [b]Conversion determined by ^{19}F NMR with reference to an internal standard. [c]Heated at 40 °C. [d]Heated at 60 °C. [e]Heated at 80 °C.

Screen of Base and Boronic Acid Stoichiometry 2

Table S 10: Investigation of the impact of reagent stoichiometry in the generation of oximes from hydroxamoyl chlorides[a]

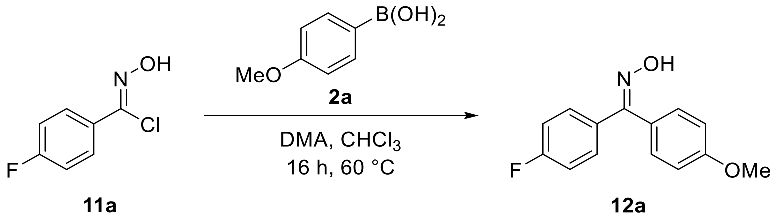

Entry	Base Stoichiometry (equiv.)	Boronic Acid Stoichiometry (equiv.)	Conversion (%)[b]
1	3	1.5	**64**
2	3	2	**69 (54[c,d])**
3	3	2.5	**71**
4	3	3	**77**
5	5	1.5	**65**
6	5	2	**70 (62[c,d])**
7	5	2.5	**78**

[a]Reactions performed at a concentration of 0.1 M. [b]Conversion determined by ^{19}F NMR with reference to an internal standard. [c]Isolated yield. [d]3 hours reaction time.

4 Characterisation of Compounds

4.1 Starting Materials

Compound S2a, 1-(4-fluorobenzylidene)-2-phenylhydrazine

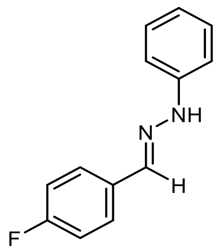

Synthesised according to General Procedure A using 4-fluorobenzaldehyde (5.27 mL, 50 mmol) and phenylhydrazine hydrochloride (7.59 g, 52.5 mmol), to yield the product (9.04 g, 84 %) as a pink solid.

ν_{max} (neat): 3310, 3053, 1595, 1574, 1524, 1502, 1487, 1445, 1229 cm^{-1}

^{1}H NMR (400 MHz, $CDCl_3$) δ 7.66 – 7.61 (m, 3H, 2 x ArH, 1 x ArCNH), 7.32 – 7.25 (m, 2H, 2 x ArH), 7.15 – 7.02 (m, 4H, 4 x ArH), 6.89 (t, *J* = 7.3 Hz, 1H, 1 x ArH), exchangeable proton not observed

^{13}C NMR (101 MHz, $CDCl_3$) δ 163.0 (d, $^{1}J_{C\text{-}F}$ = 248.2 Hz), 144.8, 136.2, 131.7, 129.5, 127.9 (d, $^{3}J_{C\text{-}F}$ = 8.0 Hz), 120.4, 115.8 (d, $^{2}J_{C\text{-}F}$ = 22.0 Hz), 112.9

^{19}F NMR (376 MHz, $CDCl_3$) δ -112.72 (tt, $J_{H\text{-}F}$ = 8.6, 5.5 Hz)

HRMS (ESI) *m/z*: $[M+H]^+$ calculated for $C_{13}H_{12}FN_2$ 215.0979, found 215.0980

Consistent with previously reported data.[6]

Compound 1a, 4-fluoro-*N*-phenylbenzohydrazonoyl chloride

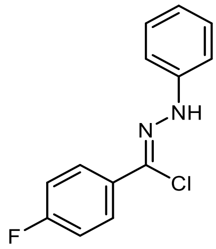

Synthesised according to General Procedure B using 4-fluorobenzaldehyde phenylhydrazone **S2a** (6.4 g, 30 mmol), *N*-chlorosuccinimide (6.8 g, 51 mmol), and dimethyl sulfide (6.7 mL, 90 mmol) to yield the product (3.1 g, 42 %) as a light yellow solid.

ν_{max} (neat): 3308, 3051, 1595, 1572, 1499, 1435, 1406, 1227 cm^{-1}

^{1}H NMR (400 MHz, $CDCl_3$) δ 7.99 (br. s, 1H, NH), 7.94 – 7.87 (m, 2H, 2 x ArH), 7.35 – 7.28 (m, 2H, 2 x ArH), 7.20 – 7.14 (m, 2H, 2 x ArH), 7.14 – 7.06 (m, 2H, 2 x ArH), 6.95 (tt, *J* = 7.4, 1.1 Hz, 1H, ArH)

^{13}C NMR (101 MHz, $CDCl_3$) δ 163.5 (d, $^{1}J_{C\text{-}F}$ = 249.8 Hz), 143.4, 130.8 (d, $^{4}J_{C\text{-}F}$ = 3.6 Hz), 129.6, 128.4 (d, $^{3}J_{C\text{-}F}$ = 8.1 Hz), 123.8, 121.4, 115.6 (d, $^{2}J_{C\text{-}F}$ = 21.9 Hz), 113.6

^{19}F NMR (471 MHz, $CDCl_3$) δ -111.77 - -111.86 (m)

HRMS (ESI) *m/z*: $[M-H]^+$ calculated for $C_{13}H_{11}ClFN_2$ 247.0433, found 247.0430

Consistent with previously reported data.[7]

Compound S2b, 1-benzylidene-2-phenylhydrazine

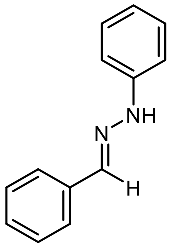

Synthesised according to General Procedure A using benzaldehyde (2.65 mL, 25 mmol) and phenylhydrazine hydrochloride (2.87 g, 25 mmol), to yield the product (4.11 g, 84 %) as an off-white solid.

ν_{max} (neat): 3310, 3055, 3024, 1591, 1564, 1522, 1493, 1483, 1440 cm^{-1}

^1H NMR (400 MHz, $CDCl_3$) δ 7.71 – 7.64 (m, 2H, 2 x ArH), 7.63 (s, 1H, 1 x ArCNH), 7.43 – 7.35 (m, 2H, 2 x ArH), 7.35 – 7.27 (m, 3H, 3 x ArH), 7.17 – 7.10 (m, 2H, 2 x ArH), 6.90 (tt, *J* = 7.3, 1.1 Hz, 1H, 1 x ArH), exchangeable proton not observed

^{13}C NMR (101 MHz, $CDCl_3$) δ 144.8, 137.5, 135.5, 129.4, 128.7, 128.5, 126.3, 120.2, 112.9

HRMS (ESI) *m/z*: $[M+H]^+$ calculated for $C_{13}H_{13}N_2$ 197.1073, found 197.1073

Consistent with previously reported data.[6]

Compound 1b, *N*-phenylbenzohydrazonoyl chloride

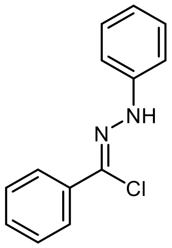

Synthesised according to General Procedure B using benzaldehyde phenylhydrazone **S2b** (4.9 g, 25 mmol), *N*-chlorosuccinimide (5.7 g, 43 mmol), and dimethyl sulfide (5.5 mL, 75 mmol) to yield the product (5.8 g, 64 %) as a light pink solid.

ν_{max} (neat): 3302, 3049, 2955, 2918, 1595, 1581, 1570, 1501, 1487, 1447 cm^{-1}

^1H NMR (400 MHz, $CDCl_3$) δ 8.05 (br. s, 1H, NH), 7.98 – 7.90 (m, 2H, 2 x ArH), 7.45 – 7.36 (m, 3H, 3 x ArH), 7.35 – 7.29 (m, 2H, 2 x ArH), 7.22 – 7.17 (m, 2H, 2 x ArH), 6.95 (tt, *J* = 7.4, 1.1 Hz, 1H, 1 x ArH)

^{13}C NMR (101 MHz, $CDCl_3$) δ 143.5, 134.6, 129.5, 129.4, 128.6, 126.6, 124.8, 121.3, 113.6

HRMS (ESI) *m/z*: $[M-H]^+$ calculated for $C_{13}H_{10}ClN_2$ 229.0527, found 229.0525

Consistent with previously reported data.[1]

Compound S2c, 1-(4-methoxybenzylidene)-2-phenylhydrazine

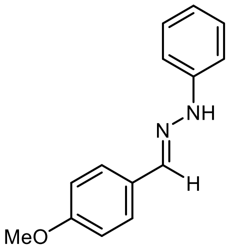

Synthesised according to General Procedure A using 4-methoxybenzaldehyde (0.68 mL, 5 mmol) and phenylhydrazine hydrochloride (795 mg, 5.5 mmol), to yield the product (756 mg, 67 %) as a pale yellow solid.

ν_{max} (neat): 3314, 3024, 2955, 1593, 1528, 1497, 1464 cm^{-1}

^{1}H NMR (400 MHz, $CDCl_3$) δ 7.65 (s, 1H, ArCHN), 7.61 (d, *J* = 8.7 Hz, 2H, 2 x ArH), 7.33 – 7.23 (m, 2H, 2 x ArH), 7.14 – 7.07 (m, 2H, 2 x ArH), 6.92 (d, *J* = 8.7 Hz, 2H, 2 x ArH), 6.90 – 6.82 (m, 1H, ArH), 3.85 (s, 3H, OCH_3), exchangeable proton not observed

^{13}C NMR (101 MHz, $CDCl_3$) δ 160.2, 145.1, 137.5, 129.4, 128.3, 127.7, 120.0, 114.3, 112.8, 55.5

HRMS (ESI) *m/z*: $[M+H]^+$ calculated for $C_{14}H_{15}N_2O$ 227.1183, found 227.1179

Consistent with previously reported data.[8]

Compound 1c, 4-methoxy-*N*-phenylbenzohydrazonoyl chloride

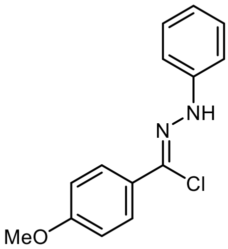

Synthesised according to General Procedure B using 4-methoxybenzaldehyde phenylhydrazone **S2c** (617 mg, 4.4 mmol), *N*-chlorosuccinimide (620 mg, 7.5 mmol), and dimethyl sulfide (0.62 mL, 13.3 mmol) to yield the product (165 mg, 23 %) as a white solid.

ν_{max} (neat): 3314, 3055, 3007, 2957, 2930, 2837, 1599, 1584, 1499, 1462, 1433 cm^{-1}

^{1}H NMR (400 MHz, $CDCl_3$) δ 7.95 (br. s, 1H, NH), 7.91 – 7.84 (m, 2H, 2 x ArH), 7.35 – 7.28 (m, 2H, 2 x ArH), 7.20 – 7.15 (m, 2H, 2 x ArH), 6.99 – 6.89 (m, 3H, 3 x ArH), 3.86 (s, 3H, OCH_3)

^{13}C NMR (101 MHz, $CDCl_3$) δ 160.7, 143.7, 129.5, 128.0, 127.3, 124.9, 121.0, 113.9, 113.4, 55.5

HRMS (ESI) *m/z*: $[M+H]^+$ calculated for $C_{14}H_{14}ClN_2O$ 225.1023, found 225.1022

Consistent with previously reported data.[7]

Compound S2d, 1-(4-nitrobenzylidene)-2-phenylhydrazine

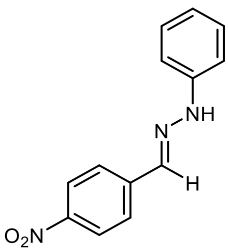

Synthesised according to General Procedure A using 4-nitrobenzaldehyde (755.6 mg, 5 mmol) and phenylhydrazine hydrochloride (795 mg, 5.5 mmol), to yield the product (953.4 mg, 79 %) as a red solid.

ν_{max} (neat): 3300, 3059, 3034, 1593, 1557, 1537, 1493, 1449, 1410, 1315, 1271 cm^{-1}

^{1}H NMR (500 MHz, $CDCl_3$) δ 8.22 (d, *J* = 8.9 Hz, 2H, 2 x ArH), 8.00 (br. s, 1H, NH), 7.76 (d, *J* = 8.9 Hz, 2H, 2 x ArH), 7.68 (s, 1H, ArCHN), 7.36 – 7.29 (m, 2H, 2 x ArH), 7.15 (d, *J* = 7.6 Hz, 2H, 2 x ArH), 6.98 - 6.92 (m, 1H, ArH)

^{13}C NMR (126 MHz, $CDCl_3$) δ 147.2, 143.7, 141.9, 133.9, 129.6, 126.4, 124.2, 121.4, 113.3

HRMS (ESI) *m/z*: $[M+H]^+$ calculated for $C_{13}H_{12}N_3O_2$ 242.0924, found 242.0927

Consistent with previously reported data.[6]

Compound 1d, 4-nitro-*N*-phenylbenzohydrazonoyl chloride

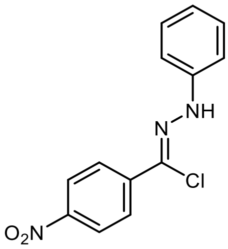

Synthesised according to General Procedure B using 4-nitrobenzaldehyde phenylhydrazone **S2d** (1000 mg , 4.1 mmol), *N*-chlorosuccinimide (940 mg, 7.1 mmol), and dimethyl sulfide (0.92 mL, 12.5 mmol) to yield the product (512 mg, 45 %) as a red solid.

ν_{max} (neat): 3318, 3057, 2924, 1603, 1593, 1547, 1504, 1441, 1406, 1379, 1331 cm^{-1}

^{1}H NMR (400 MHz, $CDCl_3$) δ 8.31 – 8.22 (m, 3H, 2 x ArH, NH), 8.10 – 8.04 (m, 2H, 2 x ArH), 7.38 – 7.32 (m, 2H, 2 x ArH), 7.24 – 7.19 (m, 2H, 2 x ArH), 7.06 – 6.98 (m, 1H, ArH)

^{13}C NMR (101 MHz, $CDCl_3$) δ 147.8, 142.6, 140.3, 129.7, 126.8, 123.9, 122.4, 122.3, 114.0

HRMS (ESI) *m/z*: $[M+H]^+$ calculated for $C_{13}H_{11}ClN_3O_2$ 276.0540, found 276.0546

Consistent with previously reported data.[9]

Compound S3a, *N*'-phenylpivalohydrazide

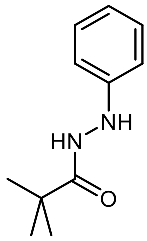

Synthesised according to General Procedure C using pivaloyl chloride (245 µL, 2.0 mmol), phenyl hydrazine (985 µL, 10 mmol), and pyridine (177.2 µL, 2.2 mmol) to yield the product (367 mg, 95 %) as a yellow solid.

ν_{max} (neat): 3260, 3252, 2968, 1657, 1597, 1537, 1493, 1481, 1435, 1400, 1358 cm^{-1}

^{1}H NMR (500 MHz, DMSO-d_6, 80 °C) δ 10.09 (s, 0.2H, NH), 9.26 (s, 0.8H, NH), 7.38 – 7.09 (m, 3H, 3 x ArH), 6.74 – 6.69 (m, 2H, 2 x ArH), 1.21 (s, *J* = 4.6 Hz, 7.2H, ^{t}Bu), 1.14 (s, 1.8H, ^{t}Bu) (84:16 mixture of rotamers)

^{13}C NMR (126 MHz, DMSO-d_6, 80 °C) δ 176.6, 149.4, 128.1, 118.1, 112.1, 37.0, 26.9

HRMS (ESI) *m/z*: $[M+H]^+$ calculated for $C_{11}H_{17}N_2O$ 193.1341, found 193.1337

Consistent with previously reported data.[10]

Compound 1e, *N*-phenylpivalohydrazonoyl chloride

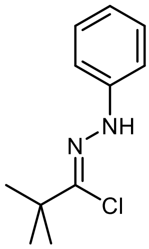

Synthesised according to General Procedure D using hydrazide **S3a** (367 mg, 1.9 mmol), triphenylphosphine (600 mg, 2.3 mmol), and carbon tetrachloride (250 µL, 2.5 mmol) to yield the product (203 mg, 51 %) as an orange liquid.

ν_{max} (neat): 3333, 2972, 2930, 2903, 2868, 1601, 1502, 1477, 1458, 1435, 1395, 1364, 1337, 1310 cm^{-1}

^{1}H NMR (500 MHz, $CDCl_3$) δ 7.63 (s, 1H, NH), 7.26 (t, *J* = 7.9 Hz, 2H, 2 x ArH), 7.04 (d, *J* = 7.7 Hz, 2H, 2 x ArH), 6.88 (t, *J* = 7.3 Hz, 1H, ArH), 1.31 (s, 9H, ^{t}Bu)

^{13}C NMR (126 MHz, $CDCl_3$) δ 144.3, 135.9, 129.4, 120.6, 113.2, 41.1, 28.5

HRMS (ESI) *m/z*: $[M]^+$ calculated for $C_{11}H_{15}ClN_2$ 210.0924, found 210.0925

Compound S3b, *N'*-phenylhexanehydrazide

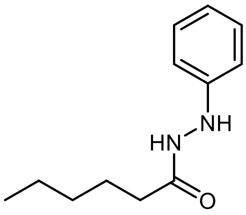

Synthesised according to General Procedure C using hexanoyl chloride (280 μL, 2.0 mmol), phenyl hydrazine (985 μL, 10 mmol), and pyridine (177.2 μL, 2.2 mmol) to yield the product (116 mg, 28 %) as a yellow solid.

ν_{max} (neat): 3291, 3215, 3084, 3026, 2953, 2926, 2070, 2853, 1661, 1636, 1601, 1570, 1495, 1460, 1437, 1377, 1335 cm^{-1}

^{1}H NMR (500 MHz, DMSO-d_6, 80 °C) δ 9.37 (s, 1H, NH), 7.13 (t, *J* = 7.4 Hz, 2H, 2 x ArH), 6.75 – 6.67 (m, 3H, 3 x ArH), 2.17 (t, *J* = 7.2 Hz, 2H, CH_2), 1.63 – 1.55 (m, 2H, CH_2), 1.32 (app. s, 4H, 2 x CH_2), 0.93 – 0.82 (m, 3H, CH_3) 1H not observed (exchangeable)

^{13}C NMR (126 MHz, DMSO-d_6, 80 °C) δ 171.5, 149.1, 128.1, 118.1, 112.0, 33.0, 30.5, 24.3, 21.3, 13.2

HRMS (ESI) *m/z*: $[M+H]^+$ calculated for $C_{12}H_{19}N_2O$ 207.1497, found 207.1502

Compound 1f, *N*-phenylhexanehydrazonoyl chloride

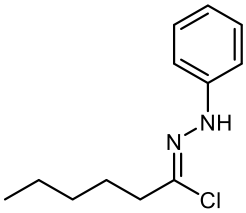

Synthesised according to General Procedure D using hydrazide **S3b** (272 mg, 1.32 mmol), triphenylphosphine (416 mg, 1.58 mmol), and carbon tetrachloride (166 μL, 1.72 mmol) to yield the product (75 mg, 25 %) as a yellow liquid.

ν_{max} (neat): 3150, 3053, 3015, 2955, 2928, 2859, 1665, 1601, 1576, 1558, 1495, 1458, 1379, 1294 cm^{-1}

^{1}H NMR (500 MHz, $CDCl_3$) δ 7.62 (s, 1H, NH), 7.29 (d, *J* = 7.9 Hz, 2H, 2 x ArH), 7.06 (d, *J* = 7.6 Hz, 2H, 2 x ArH), 6.91 (t, *J* = 7.3 Hz, 1H, ArH), 2.67 – 2.61 (m, 2H, CH_2), 1.79 – 1.70 (m, 2H, CH_2), 1.43 – 1.35 (m, 4H, 2 x CH_2), 0.95 (t, *J* = 7.0 Hz, 3H, CH_3)

^{13}C NMR (126 MHz, $CDCl_3$) δ 144.1, 129.4, 128.0, 120.6, 113.2, 38.9, 30.9, 26.5, 22.5, 14.1

HRMS (ESI) *m/z*: [M-HCl] calculated for $C_{12}H_{17}N_2$ 189.1392, found 189.1389

Compound 11a, 4-fluoro-*N*-hydroxybenzimidoyl chloride

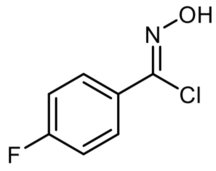

Synthesised according to General Procedure E using 4-fluorobenzaldehyde (2700 µL, 25 mmol), hydroxylamine hydrochloride (3475 mg, 50 mmol), sodium hydroxide (2000 mg, 50 mmol), and *N*-chlorosuccinimide (1795 mg, 13.4 mmol) to yield the product (1966 mg, 46 %) as a white solid.

ν_{max} (neat): 3366, 3204, 3183, 3073, 3055, 1599, 1504, 1435, 1410, 1298, 1234 cm^{-1}

^{1}H NMR (400 MHz, $CDCl_3$) δ 8.08 (s, 1H, OH), 7.84 (dd, J = 8.7, 5.3 Hz, 2H, 2 x ArH), 7.10 (app. t, J = 8.7 Hz, 2H, 2 x ArH)

^{13}C NMR (126 MHz, $CDCl_3$) δ 164.4 (d, $^{1}J_{C\text{-}F}$ = 251.9 Hz), 139.7, 129.4 (d, $^{3}J_{C\text{-}F}$ = 8.6 Hz), 128.6 (d, $^{4}J_{C\text{-}F}$ = 3.3 Hz), 115.8 (d, $^{2}J_{C\text{-}F}$ = 22.2 Hz)

^{19}F NMR (471 MHz, $CDCl_3$) δ -109.22 – -109.30 (m)

HRMS (ESI) *m/z*: $[M+H]^+$ calculated for C_7H_6ClFNO 174.0122, found 174.0117

Consistent with previously reported data.[11]

Compound 11b, *N*-hydroxybenzimidoyl chloride

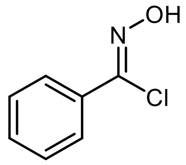

Synthesised according to General Procedure E using benzaldehyde (2551 µL, 25 mmol), hydroxylamine hydrochloride (3475 mg, 50 mmol), sodium hydroxide (2000 mg, 50 mmol), and *N*-chlorosuccinimide (3672 mg, 27.5 mmol) to yield the product (3701 mg, 95%) as a yellow solid.

ν_{max} (neat): 3269, 3125, 3092, 3061, 3036, 2899, 1624, 1578, 1491, 1449, 1314, 1233 cm^{-1}

^{1}H NMR (500 MHz, $CDCl_3$) δ 8.10 (s, 1H, OH), 7.85 (d, J = 8.0 Hz, 2H, 2 x ArH), 7.49 – 7.39 (m, 3H, 3 x ArH)

^{13}C NMR (126 MHz, $CDCl_3$) δ 140.5, 132.5, 130.9, 128.7, 127.4

HRMS (ESI) *m/z*: $[M+H]^+$ calculated for C_7H_7ClNO 156.0216, found 156.0217

Consistent with previously reported data.[12]

4.2 Ketone Hydrazones

Compound 3a, 1-((4-fluorophenyl)(4-methoxyphenyl)methylene)-2-phenylhydrazine

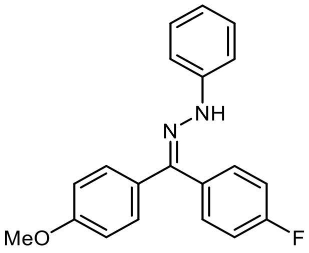

1. Hydrazonyl chloride **1a** (57.7 mg, 0.25 mmol) and 4-methoxybenzeneboronic acid (41.8 mg, 0.28 mmol) were dissolved in DCM (2.5 mL). K_3PO_4 (159 mg, 0.75 mmol) was added and the reaction mixture was stirred in a 5 mL microwave vial at 40 °C for 3 hours. Following reaction completion, the mixture was diluted with ethyl acetate, passed through celite and concentrated under reduced pressure. The crude residue was purified by column chromatography to yield the product (60.2 mg, 80 %, 46:54 *E*:*Z* mixture) as a yellow oil.

2. Synthesised according to General Procedure F using hydrazonyl chloride **1a** (62.0 mg, 0.25 mmol), 4-methoxyphenylboronic acid (61.8 mg, 0.50 mmol) and K_3PO_4 (159 mg, 0.75 mmol) to yield the product (61.8 mg, 75 %, 46:54 *E*:*Z* mixture) as a yellow oil.

ν_{max} (neat): 3302, 3053, 3003, 2961, 2934, 2835, 1597, 1574, 1562, 1464, 1433, 1406, 1331, 1317, 1300, 1288, 1242 cm^{-1}

^{1}H NMR (400 MHz, $CDCl_3$) δ 7.61 – 7.55 (m, 2H, 2 x ArH), 7.54 (s, 1H, NH), 7.29 – 7.23 (m, 4H, 2 x ArH), 7.13 – 7.07 (m, 4H, 4 x ArH), 7.02 (t, *J* = 8.8 Hz, 2H, 2 x ArH), 6.86 (tt, *J* = 7.5, 1.1 Hz, 1H, ArH), 3.91 (s, 3H, OCH_3)

^{13}C NMR (101 MHz, $CDCl_3$) δ 162.9 (d, $^1J_{C-F}$ = 247.5 Hz), 160.3, 144.8, 143.3, 135.1, 130.6, 129.4, 128.3 (d, $^3J_{C-F}$ = 7.9 Hz), 124.5, 120.1, 115.3, 115.2 (d, $^2J_{C-F}$ = 19.4 Hz), 113.0, 55.5

^{19}F NMR (376 MHz, $CDCl_3$) δ -114.08 (tt, *J* = 8.5, 5.5 Hz)

HRMS (ESI) *m/z*: $[M+H]^+$ calculated for $C_{20}H_{18}FN_2O$ 321.1403, found 321.1402

Compound 3b, 1-((3-fluoro-4-methoxyphenyl)(4-fluorophenyl)methylene)-2-phenylhydrazine

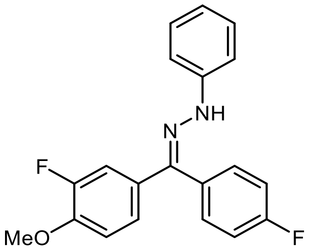

Synthesised according to General Procedure F using hydrazonyl chloride **1a** (62.0 mg, 0.25 mmol), (3-fluoro-4-methoxyphenyl)boronic acid (85.0 mg, 0.50 mmol) and K_3PO_4 (159 mg, 0.75 mmol) to yield the product (22.4 mg, 27 %, 88:12 *E*:*Z* mixture) as a yellow oil.

ν_{max} (neat): 3335, 3057, 2961, 2924, 2853, 1645, 1599, 1574, 1557, 1501, 1466, 1439, 1420, 1406 cm^{-1}

^{1}H NMR (400 MHz, $CDCl_3$) δ 7.57 – 7.52 (m, 2H, 2 x ArH), 7.49 (s, 1H, NH), 7.29 – 7.23 (m, 2H, 2 x ArH), 7.17 (t, J = 8.4 Hz, 1H, 1 x ArH), 7.10 – 7.05 (m, 4H, 4 x ArH), 7.04 – 6.98 (m, 2H, 2 x ArH), 6.86 (tt, J = 7.5, 1.1 Hz, 1H, ArH), 4.00 (s, 3H, OCH_3)

^{13}C NMR (101 MHz, $CDCl_3$) δ 163.0 (d, $^{1}J_{C,F}$ = 248.1 Hz), 153.2 (d, $^{1}J_{C-F}$ = 249.4 Hz), 148.6 (d, $^{2}J_{C-F}$ = 10.5 Hz), 144.6, 141.9, 134.6, 129.4, 128.3 (d, $^{3}J_{C-F}$ = 7.9 Hz), 125.6 (d, $^{3}J_{C-F}$ = 4.1 Hz), 125.0 (d, $^{3}J_{C-F}$ = 5.7 Hz), 120.4, 117.2 (d, $^{2}J_{C,F}$ = 18.5 Hz), 115.3 (d, $^{2}J_{C-F}$ = 21.6 Hz), 114.5, 113.1, 56.5

^{19}F NMR (376 MHz, $CDCl_3$) δ -113.77 (tt, J = 8.5, 5.5 Hz), -132.29 – -132.40 (m)

HRMS (ESI) *m/z*: $[M+H]^+$ calculated for $C_{20}H_{17}F_2N_2O$ 339.1303, found 339.1306

Compound 3c, 1-((4-methoxyphenyl)(phenyl)methylene)-2-phenylhydrazine

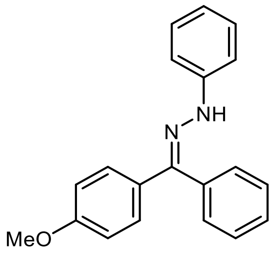

Synthesised according to General Procedure F using hydrazonyl chloride **1b** (57.7 mg, 0.25 mmol), 4-methoxyphenylboronic acid (61.8 mg, 0.50 mmol) and K_3PO_4 (159 mg, 0.75 mmol) to yield the product (60.2 mg, 75 %, 46:54 *E*:*Z* mixture) as a yellow oil.

To an oven-dried 100 mL round-bottom flask was added hydrazonyl chloride **8** (1153.5 mg, 5 mmol, 1 equiv.), and 4-methoxyphenylboronic acid (836 mg, 5.5 mmol, 1.1 equiv.). The mixture was dissolved in DCM (50 mL), and K_3PO_4 (3184 mg, 15 mmol, 3 equiv.) was added. The solution was heated at 40 °C for 9 h. The reaction mixture was diluted with ethyl acetate, filtered through Celite and rinsed with additional ethyl acetate. The crude solution was concentrated under vacuum and purified by column chromatography to furnish hydrazone **3c** (1087 mg, 72 %, 26:74 *E*:*Z* mixture) as a yellow oil.

ν_{max} (neat): 3306, 3055, 3024, 2963, 2932, 1649, 1599, 1578, 1503, 1491, 1458, 1443 cm^{-1}

^{1}H NMR (400 MHz, $CDCl_3$) δ 7.67 – 7.50 (m, 4H, 4 x ArH), 7.46 – 7.40 (br. s, 0.5 H, NH), 7.40 – 7.24 (m, 5.5 H, 5 x ArH, 0.5 x NH), 7.16 – 7.07 (m, 3H, 3 x ArH), 6.92 – 6.83 (m, 2H, 2 x ArH), 3.92 (s, 1.5 H, OCH_3), 3.84 (s, 1.5 H, OCH_3) (46:54 mixture of stereoisomers using general procedure F)

^{13}C NMR (101 MHz, $CDCl_3$) δ 160.2, 159.9, 145.0, 144.8, 144.4, 144.2, 138.9, 133.1, 131.4, 130.7, 129.7, 129.3, 129.2, 128.3, 128.1, 128.0, 126.7, 124.7, 120.1, 119.9, 115.2, 113.8, 113.0, 112.9, 55.5, 55.4 (mixture of stereoisomers)

HRMS (ESI) *m/z*: $[M+H]^+$ calculated for $C_{20}H_{19}N_2O$ 303.1493, found 303.1492

Consistent with previously reported data.[13]

Compound 3d, *tert*-butyl (4-(phenyl(2-phenylhydrazono)methyl)phenyl)carbamate

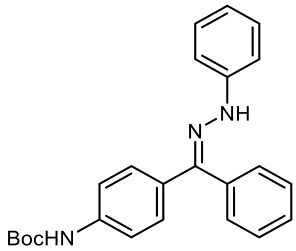

Synthesised according to General Procedure F using hydrazonyl chloride **1b** (57.7 mg, 0.25 mmol), (4-((*tert*-butoxycarbonyl)amino)phenyl)boronic acid (118.5 mg, 0.5 mmol), and K_3PO_4 (159.2 mg, 0.75 mmol) to afford the product as a yellow oil (72.6 mg, 75 %, 55:45 *E*:*Z* mixture).

ν_{max} (neat): 3327, 3055, 2976, 2930, 1725, 1701, 1641, 1599, 1518, 1501, 1445, 1406 cm^{-1}

^{1}H NMR (500 MHz, $CDCl_3$) δ 7.64 – 7.49 (m, 5H, 5 x ArH), 7.47 – 7.43 (m, 0.6H, NH), 7.36 – 7.23 (m, 6.4H, 6 x ArH, 0.4 x NH), 7.13 – 7.06 (m, 2H, 2 x ArH), 6.86 (m, 1H, 1 x ArH), 6.76 (br. s, 0.6H, NH), 6.60 (br. s, 0.4H, NH), 1.60 – 1.57 (m, 5.1H, ^{t}Bu), 1.56 – 1.53 (m, 3.9H, ^{t}Bu) (55:45 mixture of stereoisomers)

^{13}C NMR (101 MHz, $CDCl_3$) δ 152.8, 152.7, 144.9, 144.8, 144.2, 144.0, 139.4, 138.7, 138.4, 133.4, 132.9, 130.2, 129.8, 129.34, 129.25, 128.3, 128.1, 127.4, 127.1, 126.7, 120.1, 120.0, 119.5, 118.2, 113.1, 113.0, 81.2, 80.8, 28.5 (mixture of stereoisomers)

HRMS (ESI) *m/z*: $[M+H]^+$ calculated for $C_{24}H_{26}N_3O_2$ 388.2020, found 388.2011

Compound 3e, 1-((4-(*tert*-butyl)phenyl)(phenyl)methylene)-2-phenylhydrazine

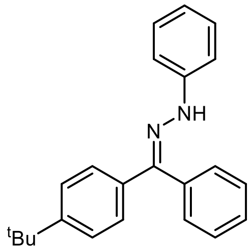

Synthesised according to General Procedure F using hydrazonyl chloride **1b** (57.7 mg, 0.25 mmol), 4-(*tert*-butyl)phenylboronic acid (89.0 mg, 0.50 mmol), and K_3PO_4 (159.2 mg, 0.75 mmol) to afford the product (49.9 mg, 61 %, 48:52 *E*:*Z* mixture) as a yellow oil.

ν_{max} (neat): 3325, 3057, 2961, 2866, 1655, 1601, 1578, 1501, 1491, 1445 cm^{-1}

^{1}H NMR (500 MHz, $CDCl_3$) δ 7.62 – 7.45 (m, 5H, 5 x ArH), 7.43 (br. s, 0.5H, 0.5 x NH), 7.35 – 7.27 (m, 3H, 3 x ArH), 7.25 – 7.19 (m, 3.5H, 3 x ArH, 0.5 x NH), 7.09 – 7.02 (m, 2H, 2 x ArH), 6.84 – 6.78 (m, 1H, ArH), 1.39 (s, 4.8H, ^{t}Bu), 1.31 (s, 4.2H, ^{t}Bu) (48:52 mixture of stereoisomers)

^{13}C NMR (126 MHz, $CDCl_3$) δ 152.4, 151.3, 144.89, 144.87, 144.5, 138.8, 135.8, 133.1, 129.8, 129.7, 129.32, 129.28, 129.0, 128.3, 128.0, 126.71, 126.66, 126.4, 125.3, 120.1, 120.0, 113.08, 113.05, 113.0, 31.5, 31.4 (mixture of stereoisomers)

HRMS (ESI) *m/z*: $[M+H]^+$ calculated for $C_{23}H_{25}N_2$ 329.2012, found 329.2014

Compound 3f, 1-phenyl-2-(phenyl(*p*-tolyl)methylene)hydrazine

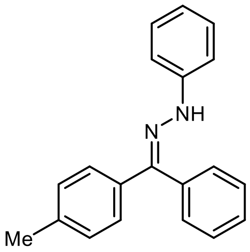

Synthesised according to General Procedure F using hydrazonyl chloride **1b** (57.7 mg, 0.25 mmol), *p*-tolylboronic acid (68.0 mg, 0.50 mmol), and K_3PO_4 (159.2 mg, 0.75 mmol) to afford the product (20.4 mg, 28 %, 44:56 *E*:*Z* mixture) as a yellow oil.

ν_{max} (neat): 3318, 3053, 3024, 2980, 2920, 1736, 1710, 1655, 1601, 1578, 1555, 1487, 1441 cm^{-1}

^1H NMR (400 MHz, $CDCl_3$) δ 7.67 – 7.50 (m, 4H, 4 x ArH), 7.48 (s, 0.5H, NH), 7.41 (d, *J* = 7.7 Hz, 1H, ArH), 7.38 - 7.29 (m, 3H, 3 x ArH), 7.29 – 7.27 (m, 1.5H, 1.5 x ArH, 0.5 x NH), 7.26 – 7.24 (m, 1H, ArH), 7.19 – 7.14 (m, 1H, ArH), 7.14 – 7.09 (m, 2H, 2 x ArH), 6.91 – 6.84 (m, 1H, ArH), 2.50 (s, 1.7H, CH_3), 2.39 (s, 1.3H, CH_3) (44:56 mixture of stereoisomers)

^{13}C NMR (101 MHz, $CDCl_3$) δ 144.9, 144.8, 144.52, 144.48, 139.3, 138.7, 138.1, 135.8, 133.1, 130.5, 129.8, 129.32, 129.26, 129.2, 129.0, 128.3, 128.1, 126.7, 126.6, 120.1, 120.0, 113.03, 112.99, 112.96, 21.6, 21.4 (mixture of stereoisomers)

HRMS (ESI) *m/z*: $[M+H]^+$ calculated for $C_{20}H_{19}N_2$ 287.1543, found 287.1544

Consistent with previously reported data.[14]

Compound 3g, 1-(furan-3-yl(phenyl)methylene)-2-phenylhydrazine

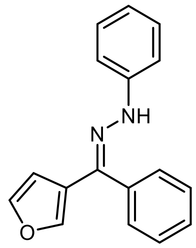

Synthesised according to General Procedure F using hydrazonyl chloride **1b** (57.7 mg, 0.25 mmol), furan-3-ylboronic acid (55.9 mg, 0.50 mmol), and K_3PO_4 (159.2 mg, 0.75 mmol) to afford the product (14.1 mg, 21 %, 50:50 *E*:*Z* mixture) as a yellow oil.

ν_{max} (neat): 3323, 3138, 3053, 3024, 2924, 2853, 1647, 1599, 1558, 1541, 1493, 1445 cm^{-1}

^1H NMR (500 MHz, $CDCl_3$) δ 7.82 (s, 0.5H, 0.5 x NH), 7.71 – 7.66 (m, 1.5H, 1.5 x ArH), 7.64 (s, 0.5H, 0.5 x ArH), 7.54 (t, *J* = 7.4 Hz, 1H, ArH), 7.51 – 7.43 (m, 1H, 0.5 x ArH, 0.5 x NH), 7.42 – 7.36 (m, 1.5H, 1.5 x ArH), 7.34 (t, *J* = 7.4 Hz, 1H, ArH), 7.31 – 7.19 (m, 2.5H, 2.5 x ArH), 7.12 (d, *J* = 8.1 Hz, 1H, ArH), 7.08 (s, 0.5H, 0.5 x ArH), 7.02 (d, *J* = 8.1 Hz, 1H, ArH), 6.94 (s, *J* = 0.7 Hz, 0.5H, 0.5 x ArH), 6.86 (t, *J* = 7.3 Hz, 0.5H, 0.5 x ArH), 6.82 (t, *J* = 7.3 Hz, 0.5H, 0.5 x ArH), 6.50 (d, *J* = 0.6 Hz, 0.5H, 0.5 ArH) (50:50 mixture of stereoisomers)

^{13}C NMR (101 MHz, $CDCl_3$) δ 144.8, 144.7, 144.6, 143.7, 142.4, 141.9, 138.6, 138.4, 136.7, 132.9, 129.7, 129.6, 129.4, 129.3, 128.6, 128.4, 128.3, 127.6, 126.6, 120.4, 120.0, 116.2, 113.2, 112.9, 111.1, 108.2 (mixture of stereoisomers)

HRMS (ESI) *m/z*: $[M+H]^+$ calculated for $C_{17}H_{15}N_2O$ 263.1179, found 263.1182

Compound 3h, 1-methyl-5-(phenyl(2-phenylhydrazono)methyl)-1*H*-indole

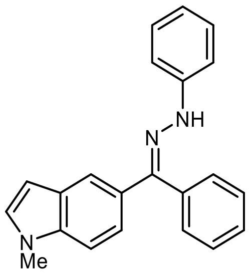

Synthesised according to General Procedure F using hydrazonyl chloride **1b** (57.7 mg, 0.25 mmol), (1-methyl-1*H*-indol-5-yl)boronic acid (87.3 mg, 0.50 mmol), and K_3PO_4 (159.2 mg, 0.75 mmol) to afford the product (30.0 mg, 37 %, 75:25 *E*:*Z* mixture) as a yellow oil.

ν_{max} (neat): 3319, 3053, 2959, 2926, 1715, 1645, 1599, 1499, 1491, 1443 cm^{-1}

^1H NMR (400 MHz, $CDCl_3$) δ 7.85 (dd, *J* = 8.7, 1.7 Hz, 0.25H, 0.25 x ArH), 7.69 – 7.58 (m, 3.5H, 2.5 x ArH, 1 x NH), 7.54 – 7.50 (m, 1.25H, 1.25 x ArH), 7.41 – 7.37 (m, 0.75H, 0.75 x ArH), 7.34 – 7.27 (m, 2.75H, 2.75 x ArH), 7.25 – 7.05 (m, 5.25H, 5.25 x ArH), 7.02 (d, *J* = 3.1 Hz, 0.25H, 0.25 x ArH), 6.86 – 6.80 (m, 1H, ArH), 6.58 (dd, *J* = 3.1, 0.8 Hz, 0.75H, 0.75 x ArH), 6.42 (dd, *J* = 3.1, 0.7 Hz, 0.25H, 0.25 x ArH), 3.90 (s, 2.25H, 2.25 x ArH), 3.80 (s, 0.75H, 0.75 x ArH) (75:25 mixture of stereoisomers)

^{13}C NMR (101 MHz, $CDCl_3$) δ 146.2, 145.8, 145.3, 145.0, 139.4, 136.9, 133.8, 130.4, 130.09, 130.06, 129.7, 129.4, 129.34, 129.30, 129.1, 128.21, 128.17, 127.9, 126.91, 126.87, 123.5, 122.4, 122.0, 120.5, 120.4, 119.8, 119.7, 113.0, 112.9, 110.7, 109.3, 101.9, 101.7, 33.2, 33.1 (mixture of stereoisomers)

HRMS (ESI) *m/z*: $[M+H]^+$ calculated for $C_{22}H_{20}N_3$ 326.1652, found 326.1654

Compound 3i, 1-phenyl-2-(phenyl(thiophen-2-yl)methylene)hydrazine

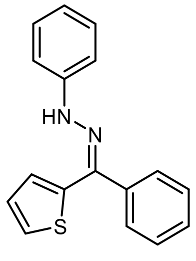

Synthesised according to General Procedure F using hydrazonyl chloride **1b** (57.7 mg, 0.25 mmol), thiophen-2-ylboronic acid (64.0 mg, 0.50 mmol), and K_3PO_4 (159.2 mg, 0.75 mmol) to afford the product (18.2 mg, 26 %, *E*:*Z* ratio not determined) as a yellow oil.

ν_{max} (neat): 3320, 3100, 3053, 3030, 2959, 2924, 1597, 1557, 1499, 1487, 1441, 1425 cm^{-1}

^{1}H NMR (400 MHz, $CDCl_3$) δ 7.95 (br. s, 0.5H, 0.3 x NH), 7.69 – 7.50 (m, 3H, 3 x ArH), 7.46 – 7.40 (m, 1.5H, 1 x ArH, 0.5 x NH), 7.38 – 7.26 (m, 3H, 3 x ArH), 7.26 – 7.19 (m, 2H, 2 x ArH), 7.13 (dd, *J* = 8.6, 1.1 Hz, 1H, ArH), 7.05 (dd, *J* = 8.6, 1.0 Hz, 1H, ArH), 6.94 – 6.82 (m, 1.5H, 1.5 x ArH), 6.66 (dd, *J* = 3.6, 1.1 Hz, 0.5H, 0.5 x ArH) (mixture of stereoisomers)

^{13}C NMR (101 MHz, $CDCl_3$) δ 144.8, 144.4, 140.9, 140.8, 138.9, 136.8, 132.2, 132.0, 129.8, 129.7, 129.5, 129.41, 129.36, 129.1, 128.33, 128.29, 128.0, 127.1, 126.7, 126.3, 126.1, 120.6, 120.3, 113.3, 113.1(mixture of stereoisomers)

HRMS (ESI) *m/z*: $[M+H]^+$ calculated for $C_{17}H_{15}N_2S$ 279.0950, found 279.0953

Consistent with previously reported data.[15]

Compound 3j, 1-((4-(methylthio)phenyl)(phenyl)methylene)-2-phenylhydrazine

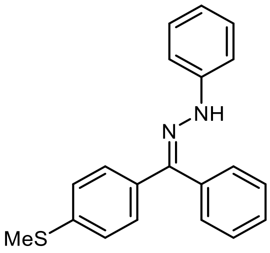

Synthesised according to General Procedure F using hydrazonyl chloride **1b** (57.7 mg, 0.25 mmol), 4-(methylthio)phenylboronic acid (84.0 mg, 0.50 mmol), and K_3PO_4 (159.2 mg, 0.75 mmol) to afford the product (40.9 mg, 51 %, 44:56 *E*:*Z* mixture) as a yellow oil.

ν_{max} (neat): 3324, 3051, 3022, 2920, 2851, 1653, 1599, 1545, 1503, 1489, 1443, 1396, cm^{-1}

^{1}H NMR (500 MHz, $CDCl_3$) δ 7.60- 7.57 (m, 2H, 2 x ArH), 7.55 – 7.49 (m, 2H, 2 x ArH), 7.46 (br. s, 1H, NH), 7.43 (d, *J* = 8.3 Hz, 1H, ArH), 7.32 (m, 2H, 2 x ArH), 7.29 – 7.22 (m, 3.5H, 3 x ArH, 0.5 x NH), 7.20 (d, *J* = 8.6 Hz, 1H, ArH), 7.12 – 7.05 (m, 2H, 2 x ArH), 6.85 (td, *J* = 7.3, 4.3 Hz, 1H, ArH), 2.58 (s, 1.5H, SCH_3), 2.49 (s, 1.5H, SCH_3) (44:56 mixture of stereoisomers)

^{13}C NMR (101 MHz, $CDCl_3$) δ 144.7, 144.0, 143.8, 140.5, 138.6, 138.5, 135.5, 132.8, 129.9, 129.8, 129.43, 129.37, 129.3, 129.1, 128.3, 128.2, 127.1, 127.0, 126.7, 126.3, 120.24, 120.19, 113.1, 113.0, 15.9, 15.4 (mixture of stereoisomers)

HRMS (ESI) *m/z*: $[M+H]^+$ calculated for $C_{20}H_{19}N_2S$ 319.1263, found 319.1260

Compound 3k, 1-(diphenylmethylene)-2-phenylhydrazine

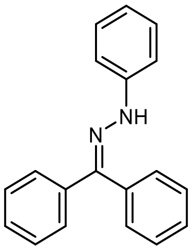

Synthesised according to General Procedure F using hydrazonyl chloride **1b** (57.7 mg, 0.25 mmol), phenylboronic acid (61.0 mg, 0.50 mmol), and K_3PO_4 (159.2 mg, 0.75 mmol) to afford the product (19.4 mg, 29 %) as a yellow oil.

ν_{max} (neat): 3321, 3055, 2924, 1599, 1578, 1560, 1501, 1489, 1443 cm^{-1}

^{1}H NMR (400 MHz, $CDCl_3$) δ 7.64 – 7.57 (m, 4H, 4 x ArH), 7.56-7.52 (m, 1H, ArH), 7.50 (br. s, 1H, NH), 7.38 – 7.22 (m, 7H, 7 x ArH), 7.12 – 7.07 (m, 2H, 2 x ArH), 6.86 (tt, *J* = 7.4, 1.1 Hz, 1H, ArH)

^{13}C NMR (101 MHz, $CDCl_3$) δ 144.8, 144.3, 138.5, 132.9, 129.8, 129.4, 129.3, 128.3, 128.1, 126.6, 120.2, 113.1, one C not observed

HRMS (ESI) *m/z*: $[M+H]^+$ calculated for $C_{19}H_{17}N_2$ 273.1386, found 273.1388

Consistent with previously reported data.[16]

Compound 3l, 1-((4-chlorophenyl)(phenyl)methylene)-2-phenylhydrazine

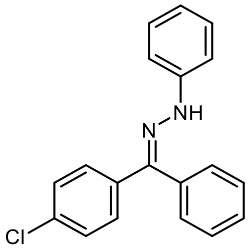

Synthesised according to General Procedure F using hydrazonyl chloride **1b** (57.7 mg, 0.25 mmol), 4-chlorophenylboronic acid (78.2 mg, 0.50 mmol), and K_3PO_4 (159.2 mg, 0.75 mmol) to afford the product (7.3 mg, 10 %, *E*:*Z* ratio not determined) as a yellow oil.

ν_{max} (neat): 3059, 3034, 2924, 2853, 1659, 1585, 1485, 1447, 1398 cm^{-1}

^{1}H NMR (400 MHz, $CDCl_3$) δ 7.63 – 7.41 (m, 6H, 5 x ArH, 1 x NH), 7.35 – 7.26 (m, 5H, 5 x ArH), 7.26 – 7.22 (m, 1H, ArH), 7.12 – 7.05 (m, 2H, 2 x ArH), 6.91 – 6.83 (m, 1H, ArH)

^{13}C NMR (101 MHz, $CDCl_3$) δ 144.6, 144.5, 143.1, 138.2, 137.1, 133.9, 132.5, 131.3, 130.9, 130.2, 130.0, 129.6, 129.4, 129.2, 128.8, 128.5, 128.4, 128.3, 127.8, 126.5, 123.3, 120.5, 120.4, 113.2, 113.1 (mixture of stereoisomers)

HRMS (ESI) *m/z*: $[M+H]^+$ calculated for $C_{19}H_{16}ClN_2$ 307.0997, found 307.1000

Consistent with previously reported data.[14]

Compound 3m, 1-((3-methoxyphenyl)(phenyl)methylene)-2-phenylhydrazine

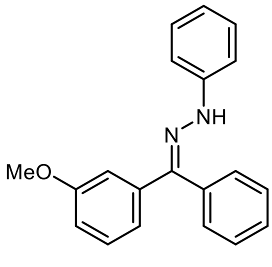

Synthesised according to General Procedure F using hydrazonyl chloride **1b** (57.7 mg, 0.25 mmol), 3-methoxyphenylboronic acid (85.0 mg, 0.50 mmol), and K_3PO_4 (159.2 mg, 0.75 mmol) to afford the product (15.4 mg, 20 %, 47:53 *E*:*Z* mixture) as a yellow oil.

ν_{max} (neat): 3325, 3298, 3055, 2955, 2926, 2853, 1655, 1597, 1578, 1504, 1485, 1447, 1431 cm^{-1}

^{1}H NMR (500 MHz, $CDCl_3$) δ 7.64 – 7.54 (m, 2H, 2 x ArH), 7.54 – 7.46 (m, 2H, 2 x ArH), 7.35 – 7.19 (m, 5.5H, 4.5 x ArH, 1 x NH), 7.11 – 7.02 (m, 3H, 3 x ArH), 6.91 (d, *J* = 7.4 Hz, 0.5H, 0.5 x

ArH), 6.88 – 6.77 (m, 2H, 2 x ArH), 3.83 (s, 1.5H, OCH_3), 3.82 (s, 1.5H, OCH_3) (47:53 mixture of stereoisomers)

^{13}C NMR (126 MHz, $CDCl_3$) δ 160.8, 159.7, 144.8, 144.7, 144.12, 144.08, 140.0, 138.3, 136.7, 134.2, 132.9, 131.0, 129.8, 129.40, 129.36, 129.3, 129.2, 128.3, 128.1, 126.6, 121.3, 120.3, 120.2, 119.6, 115.2, 114.3, 113.8, 113.1, 111.9, 55.5, 55.4 (mixture of stereoisomers)

HRMS (ESI) *m/z*: $[M+H]^+$ calculated for $C_{20}H_{19}N_2O$ 303.1492, found 303.1494

Consistent with previously reported data.[17]

Compound 3n, 1-methyl-5-(phenyl(2-phenylhydrazono)methyl)-1*H*-indazole

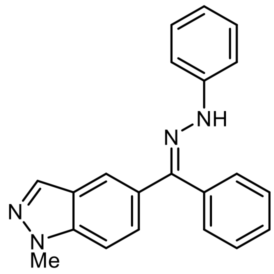

Synthesised according to General Procedure F using hydrazonyl chloride **1b** (57.7 mg, 0.25 mmol), (1-methyl-1*H*-indazol-5-yl)boronic acid (88.0 mg, 0.50 mmol), and K_3PO_4 (159.2 mg, 0.75 mmol) to afford the product (55.0 mg, 67 %, 75:25 *E*:*Z* mixture) as a yellow oil.

ν_{max} (neat): 3238, 3055, 2918, 2849, 1597, 1578, 1555, 1491, 1443 cm^{-1}

^{1}H NMR (400 MHz, $CDCl_3$) δ 8.15 (dd, *J* = 8.9, 1.6 Hz, 0.35H, 0.35 x ArH), 8.07 (d, *J* = 0.9 Hz, 0.65H, 0.65 x ArH), 7.88 (d, *J* = 0.7 Hz, 0.35H, 0.35 x ArH), 7.76 – 7.74 (m, 0.65H, 0.65 x ArH), 7.65 – 7.53 (m, 4H, 4 x ArH), 7.48 – 7.44 (m, 0.7H, 0.35 x ArH, 0.35 x NH), 7.42 – 7.35 (m, 1.3H, 0.65 x ArH, 0.65 x NH), 7.35 – 7.28 (m, 3H, 3 x ArH), 7.27 – 7.21 (m, 1H, ArH), 7.09 (td, *J* = 8.6, 1.0 Hz, 2H, 2 x ArH), 6.88 – 6.82 (m, 1H, ArH), 4.17 (s, 2H, 0.66 x NCH_3), 4.08 (s, 1H, 0.33 x NCH_3) (75:25 mixture of stereoisomers)

^{13}C NMR (101 MHz, $CDCl_3$) δ 144.91, 144.86, 144.8, 144.5, 139.9, 138.9, 133.6, 133.4, 133.1, 131.8, 129.9, 129.44, 129.36, 128.3, 128.1, 127.3, 126.7, 125.0, 124.9, 124.6, 122.4, 120.2, 120.1, 120.0, 113.1, 113.0, 110.7, 109.1, 35.9, 35.8 (mixture of stereoisomers)

HRMS (ESI) *m/z*: $[M+H]^+$ calculated for $C_{21}H_{19}N_4$ 327.1604, found 327.1604

Compound 3r, 1-(*bis*(4-methoxyphenyl)methylene)-2-phenylhydrazine

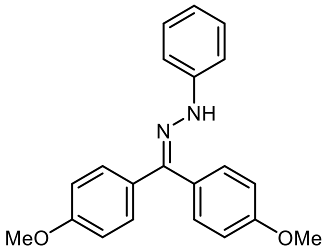

Synthesised according to General Procedure F using hydrazonyl chloride **1c** (65.2 mg, 0.25 mmol), 4-methoxyphenylboronic acid (76.0 mg, 0.50 mmol), and K_3PO_4 (159.2 mg, 0.75 mmol) to afford the product (49.3 mg, 59 %) as a yellow oil.

ν_{max} (neat): 3320, 3051, 3003, 2954, 2932, 2909, 1643, 1599, 1499, 1456, 1441, 1416 cm^{-1}

^{1}H NMR (500 MHz, $CDCl_3$) δ 7.52 (d, *J* = 8.9 Hz, 2H, 2 x ArH), 7.44 (s, 1H, NH), 7.27 – 7.19 (m, 4H, 4 x ArH), 7.10 – 7.02 (m, 4H, 4 x ArH), 6.84 (d, *J* = 8.9 Hz, 2H, 2 x ArH), 6.83 – 6.78 (m, 1H, ArH), 3.89 (s, 3H, OCH_3), 3.80 (s, 3H, OCH_3)

^{13}C NMR (101 MHz, $CDCl_3$) δ 160.2, 159.9, 145.1, 144.4, 131.8, 130.7, 129.3, 128.1, 125.0, 119.8, 115.1, 113.7, 112.9, 55.51, 55.47

HRMS (ESI) *m/z*: $[M+H]^+$ calculated for $C_{21}H_{21}N_2O_2$ 333.1598, found 333.1598

Consistent with previously reported data.[13]

Compound 3s, 1-((4-methoxyphenyl)(4-nitrophenyl)methylene)-2-phenylhydrazine

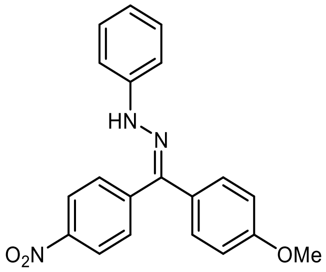

Synthesised according to General Procedure F using hydrazonyl chloride **1d** (68.9 mg, 0.25 mmol), 4-methoxyphenylboronic acid (85.0 mg, 0.50 mmol), and K_3PO_4 (159.2 mg, 0.75 mmol) to afford the product (53.2 mg, 61 %, 4:96 *E*:*Z* mixture) as a yellow oil.

ν_{max} (neat): 3306, 3291, 3057, 2997, 2926, 2841, 1641, 1591, 1549, 1504, 1487, 1466, 1439, 1404, 1339 cm^{-1}

^{1}H NMR (500 MHz, $CDCl_3$) δ 8.14 (d, *J* = 9.0 Hz, 2H, 2 x ArH), 7.78 (br. s, 1H, NH), 7.71 (d, *J* = 9.0 Hz, 2H, 2 x ArH), 7.30 – 7.22 (m, 4H, 4 x ArH), 7.13 (d, *J* = 8.7 Hz, 2H, 2 x ArH), 7.10 (d, *J* = 7.8 Hz, 2H, 2 x ArH), 6.93 – 6.88 (m, 1H, ArH), 3.92 (s, 3H, OCH_3)

^{13}C NMR (101 MHz, $CDCl_3$) δ ^{13}C NMR (126 MHz, $CDCl_3$) δ 160.7, 147.0, 145.0, 143.9, 141.6, 130.7, 129.5, 126.7, 123.7, 123.4, 121.2, 115.7, 113.5, 55.6

HRMS (ESI) *m/z*: $[M+H]^+$ calculated for $C_{20}H_{18}N_3O_3$ 348.1343, found 348.1344

Compound 3t, 1-((4-nitrophenyl)(phenyl)methylene)-2-phenylhydrazine

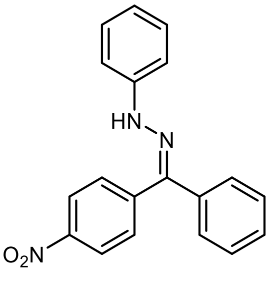

Synthesised according to General Procedure F using hydrazonyl chloride **1d** (68.9 mg, 0.25 mmol), phenylboronic acid (61.0 mg, 0.50 mmol), and K_3PO_4 (159.2 mg, 0.75 mmol) to afford the product (26.9 mg, 34 %, 90:10 *E*:*Z* mixture) as a yellow oil.

ν_{max} (neat): 3316, 3055, 2957, 2924, 2853, 1663, 1601, 1591, 1545, 1504, 1487, 1406, 1331 cm^{-1}

^{1}H NMR (400 MHz, $CDCl_3$) δ 8.49 – 8.44 (m, 0.2H, 0.2 x ArH), 8.18 – 8.13 (m, 1.8H, 1.8 x ArH), 7.74 – 7.69 (m, 2.8H, 1.8 x ArH, 1 x NH), 7.66 – 7.50 (m, 3.2H, 3.2 x ArH), 7.34 – 7.26 (m, 4H, 4 x ArH), 7.13 – 7.09 (m, 2H, 2 x ArH), 6.92 (tt, *J* = 7.5, 1.1 Hz, 1H, ArH) (90:10 mixture of stereoisomers)

^{13}C NMR (101 MHz, $CDCl_3$) δ 147.0, 144.6, 143.8, 141.6, 131.6, 130.3, 130.0, 129.5, 129.2, 126.7, 123.8, 121.3, 113.5

HRMS (ESI) *m/z*: $[M+H]^+$ calculated for $C_{19}H_{16}N_3O_2$ 318.1237, found 318.1239

Consistent with previously reported data.[18]

Compound 3u, 1-((4-(*tert*-butyl)phenyl)(4-nitrophenyl)methylene)-2-phenylhydrazine

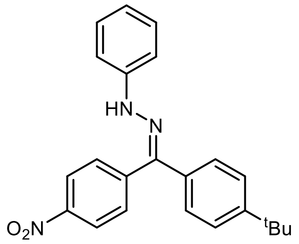

Synthesised according to General Procedure F using hydrazonyl chloride **1d** (68.9 mg, 0.25 mmol), (4-(*tert*-butyl)phenyl)boronic acid (89.0 mg, 0.50 mmol), and K_3PO_4 (159.2 mg, 0.75 mmol) to afford the product (31.3 mg, 34 %, >99:1 *E*:*Z* mixture) as a yellow oil.

ν_{max} (neat): 3321, 3053, 2959, 2926, 2864, 1599, 1543, 1504, 1487, 1404, 1364, 1302, 1246 cm^{-1}

^{1}H NMR (500 MHz, $CDCl_3$) δ 8.16 (d, *J* = 9.0 Hz, 2H, 2 x ArH), 7.81 (s, 1H, NH), 7.72 (d, *J* = 9.0 Hz, 2H, 2 x ArH), 7.63 (d, *J* = 8.3 Hz, 2H, 2 x ArH), 7.30 – 7.26 (m, 2H, 2 x ArH), 7.25 (d, *J* = 8.3 Hz, 2H, 2 x ArH), 7.12 (d, *J* = 7.7 Hz, 2H, 2 x ArH), 6.91 (t, *J* = 7.3 Hz, 1H, ArH), 1.43 (s, 9H, ^{t}Bu)

^{13}C NMR (101 MHz, $CDCl_3$) δ 153.2, 147.0, 144.9, 143.9, 141.8, 129.5, 128.9, 128.5, 127.2, 126.8, 123.7, 121.2, 113.5, 35.1, 31.4

HRMS (ESI) *m/z*: $[M+H]^+$ calculated for $C_{23}H_{24}N_3O_2$ 374.1869, found 374.1866

Compound 3v, 1-(1-(4-methoxyphenyl)-2,2-dimethylpropylidene)-2-phenylhydrazine

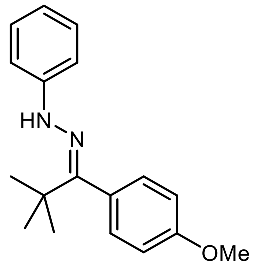

Synthesised according to General Procedure F using hydrazonyl chloride **1e** (52.7 mg, 0.25 mmol), 4-methoxyphenylboronic acid (76.0 mg, 0.50 mmol), and K_3PO_4 (159.2 mg, 0.75 mmol) to afford the product (16.2 mg, 23 %, >99:1 *E*:*Z* mixture) as a yellow solid.

ν_{max} (neat): 2957, 2928, 2870, 2857, 1724, 1665, 1601, 1574, 1508, 1476, 1460, 1443, 1416, 1395, 1366, 1304, 1275 cm^{-1}

^{1}H NMR (500 MHz, $CDCl_3$) δ 7.18 (app. t, *J* = 7.9 Hz, 2H, 2 x ArH), 7.03 (app. q, *J* = 8.8 Hz, 4H, 4 x ArH), 6.92 (d, *J* = 7.7 Hz, 2H, 2 x ArH), 6.78 – 6.73 (m, 2H, 1 x ArH, 1 x NH), 3.87 (s, 3H, OCH_3), 1.20 (s, 9H, ^{t}Bu)

^{13}C NMR (126 MHz, $CDCl_3$) δ 159.7, 154.9, 145.7, 130.2, 129.2, 125.7, 119.3, 114.7, 112.7, 55.4, 38.4, 28.8

HRMS (ESI) *m/z*: $[M-H]^-$ calculated for $C_{18}H_{21}N_2O$ 281.1654, found 281.1655

Compound 3w, 1-(4-methoxyphenyl)hexan-1-one

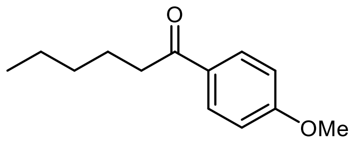

Synthesised according to General Procedure F using hydrazonyl chloride **1f** (56.2 mg, 0.25 mmol), 4-methoxyphenylboronic acid (76.0 mg, 0.50 mmol), and K_3PO_4 (159.2 mg, 0.75 mmol) to afford the product (18.0 mg, 35 %) as a yellow oil. Decomposition from the primary hydrazone product to the ketone occurred during column chromatography.

ν_{max} (neat): 3065, 3009, 2968, 2957, 2932, 2913, 2895, 2872, 2853, 2839, 1668, 1601, 1576, 1508, 1464, 1439, 1416, 1406, 1360, 1246, 1204, 1173 cm^{-1}

^{1}H NMR (500 MHz, $CDCl_3$) δ 7.94 (d, *J* = 8.9 Hz, 2H, 2 x ArH), 6.93 (d, *J* = 8.8 Hz, 2H, 2 x ArH), 3.87 (s, 3H, OCH_3), 2.94 – 2.87 (m, 2H, CH_2), 1.77 – 1.69 (m, 2H, CH_2), 1.39 – 1.34 (m, 4H, 2 x CH_2), 0.91 (t, *J* = 7.0 Hz, 3H, CH_3)

^{13}C NMR (126 MHz, $CDCl_3$) δ 199.4, 163.4, 130.4, 113.8, 55.6, 38.4, 31.7, 24.5, 22.7, 14.1 1C not observed

HRMS (ESI) *m/z*: $[M+H]^+$ calculated for $C_{13}H_{19}O_2$ 207.1385, found 207.1383

4.3 Derivatization of 3c

Compound 4, 6-methoxy-1,3-diphenyl-1*H*-indazole

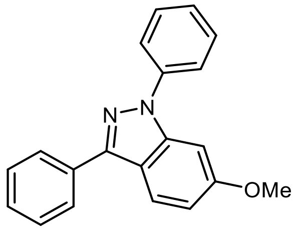

In accordance with previous literature precedent,[19] to a solution of hydrazone **3c** (75.6 mg, 0.25 mmol) in DMSO (1.5 mL) was added TEMPO (11.7 mg, 0.08 mmol) and $NaHCO_3$ (21 mg, 0.25 mmol). The reaction was stirred at 140 °C under an oxygen atmosphere for 16 hours. The mixture was quenched with H_2O and extracted twice using DCM. The combined organic phases were then washed with brine, passed through a phase separator and concentrated under vacuum. The crude product was purified using column chromatography to afford the target compound (46.7 mg, 62 %) as a white solid.

ν_{max} (neat): 3061, 3007, 2957, 2930, 2828, 1614, 1597, 1562, 1520, 1503, 1491, 1418, 1454, 1420, 1400 cm^{-1}

^{1}H NMR (500 MHz, $CDCl_3$) δ 8.04 – 8.00 (m, 2H, 2 x ArH), 7.94 (d, *J* = 8.9 Hz, 1H, ArH), 7.79 (d, *J* = 7.7 Hz, 2H, 2 x ArH), 7.57 (app. t, *J* = 7.7 Hz, 2H, 2 x ArH), 7.55 – 7.49 (m, 2H, 2 x ArH), 7.45 – 7.41 (m, 1H, ArH), 7.41 – 7.36 (m, 1H, ArH), 7.13 (d, *J* = 1.9 Hz, 1H, ArH), 6.95 (dd, *J* = 8.9, 1.9 Hz, 1H, ArH), 3.89 (s, 3H, OCH_3)

^{13}C NMR (126 MHz, $CDCl_3$) δ 160.1, 146.3, 141.8, 140.4, 133.4, 129.6, 128.9, 128.4, 127.8, 126.8, 123.3, 122.5, 117.9, 113.7, 92.1, 55.8

HRMS (ESI) *m/z*: $[M+H]^+$ calculated $C_{20}H_{17}N_2O$ 301.1335, found 301.1334

Consistent with previously reported data.[15]

Compound 5, 2-((4-methoxyphenyl)(phenyl)methylene)-1-methyl-1-phenylhydrazine

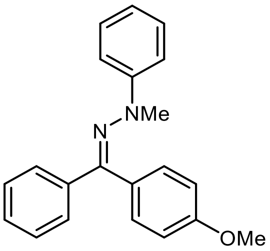

In accordance with previous literature precedent,[20] a 60 wt% suspension of sodium hydride in mineral oil (240 mg, 6 mmol) was added to a solution of hydrazone **3c** (75.6 mg, 0.25 mmol) in THF (1 mL) at -20 °C. The reaction mixture was stirred for 15 minutes, and methyl iodide (32 µL, 0.5 mmol) was added dropwise. The reaction mixture was allowed to warm to room temperature, stirred for an additional 3 hours, quenched with H_2O and extracted twice with DCM. The combined organic phases were then washed with brine, passed through a phase separator and concentrated under vacuum. The crude product was purified using column chromatography to afford the target compound (55.2 mg, 70 %) as a yellow oil.

ν_{max} (neat): 3057, 3024, 2953, 2924, 2852, 1597, 1493, 1460, 1443 cm^{-1}

^{1}H NMR (500 MHz, Acetone-d_6) δ 7.59 (dd, *J* = 8.1, 1.4 Hz, 2H, 2 x ArH), 7.38 (m, 3H, 3 x ArH), 7.34 – 7.29 (m, 2H, 2 x ArH), 7.27 – 7.22 (m, 2H, 2 x ArH), 7.18 – 7.13 (m, 2H, 2 x ArH), 7.07 – 7.03 (m, 2H, 2 x ArH), 6.87 – 6.81 (m, 1H, ArH), 3.87 (s, 3H, OCH_3), 2.91 (s, 3H, CH_3)

^{13}C NMR (101 MHz, Acetone-d_6) δ 161.0, 157.7, 151.6, 140.9, 131.4, 130.4, 130.0, 129.5, 129.0, 128.9, 120.2, 115.3, 114.8, 55.7, 41.5

HRMS (ESI) *m/z*: $[M+H]^+$ calculated $C_{21}H_{21}N_2O$ 317.1648, found 317.1654

Compound 6, 4-(4-methoxyphenyl)-1-(methyl(phenyl)amino)-3-phenoxy-4-phenylazetidin-2-one

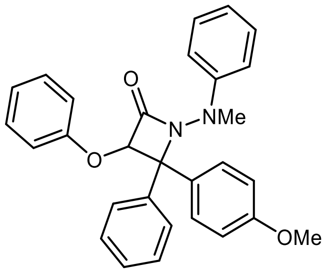

In accordance with previous literature precedent,[20] 2-phenoxyacetyl chloride (20.2 µL, 0.18 mmol), in DCM (1 mL) was added dropwise to a stirred solution of hydrazone **3c** (50.3 mg, 0.17 mmol) and triethylamine (30 µL, 0.22 mmol) in DCM (1 mL) at 0 °C. The reaction mixture was stirred at room temperature for 6 hours, quenched with $NaHCO_3$ solution and extracted twice with DCM. The combined organic phases were then washed with brine, passed through a phase separator and concentrated under vacuum. The crude product was purified using column chromatography to afford the target compound (54.7 mg, 73 %) as a white solid.

ν_{max} (neat): 3059, 3036, 2955, 2928, 2837, 1767, 1597, 1512, 1491, 1447, 1418 cm^{-1}

^1H NMR (500 MHz, $CDCl_3$) δ 7.41 – 7.37 (m, 1H, ArH), 7.33 – 7.13 (m, 10H, 10 x ArH), 7.01 – 6.94 (m, 2H, 2 x ArH), 6.91 (d, *J* = 7.8 Hz, 1.2H, 1.2 x ArH), 6.88 (d, *J* = 7.6 Hz, 0.8H, 0.8 x ArH), 6.86 - 6.82 (m, 2.4H, 2.4 x ArH), 6.81-6.77 (d, 1.6H, 1.6 x ArH), 5.79 (s, 0.6H, 0.6 x R_2CHO), 5.77 (s, 0.4H, 0.4 x R_2CHO), 3.78 (s, 1.8H, 1.8 x OCH_3), 3.77 (s, 1.2H, 1.2 x OCH_3), 3.17 (s, 1.2H, NCH_3), 3.15 (s, 1.8H, 1.8 x NCH_3) (58:42 mixture of stereoisomers)

^{13}C NMR (126 MHz, $CDCl_3$) δ 166.5, 166.4, 159.6, 159.5, 157.61, 157.56, 148.8, 148.7, 139.1, 138.1, 131.3, 130.7, 129.8, 129.7, 129.61, 129.58, 129.5, 129.1, 129.0, 128.6, 128.4, 128.14, 128.09, 127.7, 122.9, 122.8, 121.7, 121.6, 117.2, 117.1, 116.1, 115.8, 114.0, 113.1, 85.1, 84.9, 77.3, 77.2, 55.4, 55.3, 42.5, 42.3 (mixture of stereoisomers)

HRMS (ESI) *m/z*: $[M+H]^+$ calculated $C_{29}H_{27}N_2O_3$ 451.2016, found 451.2013

Compound 7, 2,3-dimethyl-1*H*-indole

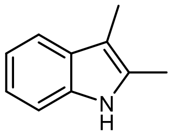

In accordance with previous literature precedent,[21] hydrazone **3c** (75.6 mg, 0.25 mmol) was added to a solution of butan-2-one (45 µL, 0.5 mmol) in ethanol (5 mL) and HCl (0.1 mL). The reaction mixture was refluxed for 16 h, before the solution was concentrated under vacuum and quenched with water. The organic phase was twice extracted using DCM, washed with brine, passed through a phase separator and concentrated under vacuum. The crude product was purified using column chromatography to afford the target compound (19.1 mg, 53 %) as a light brown solid.

ν_{max} (neat): 3393, 3051, 2914, 2857, 1649, 1609, 1584, 1522, 1483, 1462 cm^{-1}

^{1}H NMR (500 MHz, Acetone-d_6) δ 9.71 (br. s, 1H), 7.38 (d, *J* = 7.6 Hz, 1H, ArH), 7.23 (d, *J* = 7.7 Hz, 1H, ArH), 7.01 – 6.97 (m, 1H, ArH), 6.94 (td, *J* = 7.6, 0.9 Hz, 1H, ArH), 2.34 (s, 3H, CH_3), 2.18 (s, 3H, CH_3)

^{13}C NMR (126 MHz, Acetone-d_6) δ 136.7, 131.9, 130.4, 121.0, 119.1, 118.3, 111.0, 106.6, 11.3, 8.5

HRMS (ESI) *m/z*: $[M+H]^+$ calculated $C_{10}H_{12}N$ 145.0891, found 145.0879

Consistent with previously reported data.[22]

Compound 8, 2-((2-phenylhydrazono)methyl)pyridine

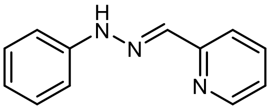

In accordance with previous literature precedent,[21] a mixture of hydrazone **3c** (75.6 mg, 0.25 mmol) and picolinaldehyde (35.7 µL, 0.38 mmol) in ethanol (1.25 mL) and HCl (0.125 mL) was stirred at room temperature for 24 hours. The reaction mixture was concentrated under vacuum and purified by column chromatography to afford the product (29.4 mg, 60 %) as a yellow solid.

ν_{max} (neat): 3223, 3183, 3125, 3057, 3022, 3003, 2945, 2891, 1597, 1566, 1493, 1466, 1450, 1435, 1356 cm^{-1}

^{1}H NMR (400 MHz, $CDCl_3$) δ 8.45 – 8.41 (m, 1H, ArH), 8.20 (s, 1H, NH), 7.90 (d, *J* = 8.1 Hz, 1H, ArH), 7.75 (s, 1H, ArCNH), 7.60 (td, *J* = 7.9, 1.5 Hz, 1H), 7.22 – 7.15 (m, 2H, 2 x ArH), 7.10 – 7.03 (m, 3H, 3 x ArH), 6.81 (tt, *J* = 7.4, 1.1 Hz, 1H, 1 x ArH)

^{13}C NMR (101 MHz, $CDCl_3$) δ 154.4, 148.5, 144.1, 136.9, 136.5, 129.5, 122.6, 121.0, 120.0, 113.2

HRMS (ESI) *m/z*: $[M+H]^+$ calculated $C_{12}H_{12}N_3$ 198.1031, found 198.1034

Consistent with previously reported data.[22]

Compound 9, (4-methoxyphenyl)(phenyl)methanone

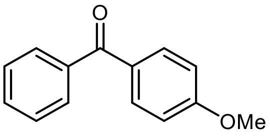

In accordance with previous literature precedent,[23] to a stirred mixture of hydrazone **3c** (75.6 mg, 0.25 mmol) and $VO(acac)_2$ (6.6 mg, 0.025 mmol) in acetone (0.5 mL) was added H_2O_2 (0.125 mL) dropwise at room temperature. The reaction mixture was stirred for two hours, and diluted with DCM. This solution was washed with 10% sodium metabisulfite and brine, passed through a phase separator and concentrated under vacuum. The crude product was purified by column chromatography to furnish ketone **9** (44.7 mg, 84 %) as an off-white solid.

ν_{max} (neat): 3063, 3003, 2965, 2841, 1639, 1595, 1578, 1504, 1468, 1439, 1414 cm^{-1}

^{1}H NMR (500 MHz, $CDCl_3$) δ 7.83 (d, *J* = 8.7 Hz, 2H, 2 x ArH), 7.75 (d, *J* = 7.6 Hz, 2H, 2 x ArH), 7.59 – 7.53 (m, 1H, ArH), 7.50 – 7.44 (m, 2H, 2 x ArH), 6.96 (d, *J* = 8.7 Hz, 2H, 2 x ArH), 3.88 (s, 3H, OCH_3)

^{13}C NMR (126 MHz, $CDCl_3$) δ 195.7, 163.4, 138.4, 132.7, 132.0, 130.3, 129.8, 128.3, 113.7, 55.6

HRMS (ESI) *m/z*: $[M+H]^+$ calculated for $C_{14}H_{13}O_2$ 213.0910, found 213.0909

Consistent with previously reported data.[24]

Compound 10, (4-methoxyphenyl-2,6-*d*2)(phenyl-2,6-*d*2)methanone

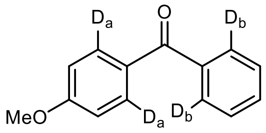

In accordance to previous literature precedent,[25] a flask bearing two stopcocks was flame dried and cooled under argon. Hydrazone **3c** (65.0 mg, 0.215 mmol), $[Ir(COD)(IMes)(PPh_3)][BAr^F_4]$ (18.6 mg, 0.0011 mmol), and DCM (2.5 mL) were then added and the flask was cooled to -78 °C with stirring. The flask was evacuated and refilled with D_2 gas from a balloon. This vacuum and refilling cycle was repeated one further time. The stopcocks were then closed and the flask was heated at 25 °C for 16 hours. The resulting mixture was concentrated under vacuum and the residue was passed through a silica plug using diethyl ether, to yield the corresponding hydrazone intermediate in excellent yield (63.5 mg, 98%).

After significant decomposition of this hydrazone was observed, a portion of hydrazone (30.0 mg, 0.10 mmol) was dissolved in acetone (0.5 mL). To this mixture was added $VO(acac)_2$ (2.7 mg, 0.01 mmol), and H_2O_2 (0.125 mL) dropwise at room temperature. The reaction mixture was stirred for two hours, and diluted with DCM. This solution was washed with 10% sodium metabisulfite and brine, passed through a phase separator and concentrated under vacuum. The crude product was purified by column chromatography to furnish ketone **10** (13.8 mg, 64%) as an off-white solid.

The extent of deuteration was assessed by ^1H NMR spectroscopy. The integrals were calibrated against a peak corresponding to a position not expected to be labelled. **Equation S1** was then used to calculate the extent of labelling:

$$\%D = 100 - \left[100 \times \left(\frac{Residual\ Integral}{Expected\ Inregral}\right)\right]$$

S1

ν_{max} (neat): 3053, 3005, 2965, 2943, 2914, 2839, 1639, 1582, 1504, 1466, 1435 cm^{-1}

^1H NMR (400 MHz, Acetone-d_6) δ 7.83 – 7.78 (m, 2H, 2 x ArH_a), 7.76 – 7.71 (m, 2H, 2 x ArH_b), 7.63 (dd, *J* = 7.8, 7.0 Hz, 1H, ArH), 7.56 – 7.51 (m, 2H, 2 x ArH), 7.10 – 7.05 (m, 2H, 2 x ArH), 3.91 (s, 3H, OCH_3)

Deuterium incorporation: D_a = 44 %, D_b = 77 %

Labelling expected against signal at 7.80 ppm and 7.74 ppm respectively, measured against signal at 3.91 ppm.

^{13}C NMR (101 MHz, Acetone-d_6) δ 185.4, 154.5, 129.4, 129.3, 123.2, 122.9, 121.1, 120.4, 120.1, 119.9, 119.3, 119.2, 104.7, 104.6, 46.1 (mixture of isotopes)

HRMS (ESI) *m/z*: $[M_{d1}+H]^+$ calculated for $C_{14}H_{12}D_1O_2$ 214.0972, found 214.0972

m/z: $[M_{d2}+H]^+$ calculated for $C_{14}H_{11}D_2O_2$ 215.1035, found 215.1030

m/z: $[M_{d3}+H]^+$ calculated for $C_{14}H_{10}D_3O_2$ 216.1098, found 216.1084
m/z: $[M_{d4}+H]^+$ calculated for $C_{14}H_9D_4O_2$ 217.1161, found 217.1146

4.4 Ketone Oximes

Compound 12a, (4-fluorophenyl)(4-methoxyphenyl)methanone oxime

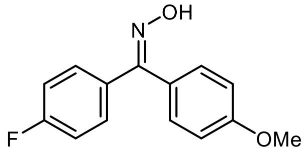

Synthesised according to General Procedure G using hydroxamoyl chloride **11a** (43.3 mg, 0.25 mmol), (4-methoxyphenyl)boronic acid (76.0 mg, 0.50 mmol), and DMA (158 µL, 1.25 mmol) to afford the product (38.0 mg, 62 %, >1:99 *E*:*Z* mixture) as a white solid.

ν_{max} (neat): 3277, 3237, 3194, 3144, 3065, 3022, 3011, 2963, 2924, 1607, 1599, 1574, 1508, 1456, 1443, 1412, 1323, 1300, 1256, 1236, 1177, 1153 cm^{-1}

^{1}H NMR (500 MHz, $CDCl_3$) δ 9.25 (br. s, 1H, OH), 7.52 – 7.42 (m, 4H, 4 x ArH), 7.10 – 6.99 (m, 4H, 4 x ArH), 3.90 (s, 3H, OCH_3)

^{19}F NMR (471 MHz, $CDCl_3$) δ -111.58 – -111.67 (m)

^{13}C NMR (126 MHz, $CDCl_3$) δ 163.7 (d, $^1J_{C\text{-}F}$ = 249.6 Hz), 160.4, 156.9, 133.0 (d, $^4J_{C\text{-}F}$ = 3.1 Hz), 131.3, 130.1 (d, $^3J_{C\text{-}F}$ = 8.3 Hz), 129.4, 124.6, 115.5 (d, $^2J_{C\text{-}F}$ = 21.6 Hz), 55.5

HRMS (ESI) *m/z*: $[M+H]^+$ calculated for $C_{14}H_{13}FNO_2$ 246.0930, found 246.0934

Consistent with previously reported data.[26]

Compound 12b, (4-methoxyphenyl)(phenyl)methanone oxime

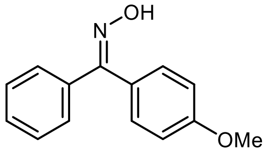

Synthesised according to General Procedure G using hydroxamoyl chloride **11b** (38.9 mg, 0.25 mmol), (4-methoxyphenyl)boronic acid (76.0 mg, 0.50 mmol), and DMA (158 µL, 1.25 mmol) to afford the product (38.5 mg, 68 %, 41:59 *E*:*Z* mixture) as a white solid.

Hydroxamoyl chloride **11b** (1500 mg, 9.64 mmol) and (4-methoxyphenyl)boronic acid (2932 mg, 19.28 mmol) were added to a 250 mL round-bottom flask and dissolved in chloroform (96 mL). The vessel was purged with N_2 and DMA (6100 µL, 48.2 mmol) was added to initiate the reaction. The mixture was heated at 60 °C for 6 hours, before being cooled to room temperature, diluted with DCM, and washed with 1M HCl solution and brine respectively. The resulting organic layers were passed through a phase separator, concentrated under vacuum and purified rapidly (~20 min from loading to elution) by column chromatography to afford the product (1585 mg, 72 %, >1:99 *E*:*Z* mixture) as a purple solid.

ν_{max} (neat): 3173, 3100, 3084, 3075, 3049, 3011, 2957, 2930, 2930, 2899, 2893, 1605, 1574, 1514, 1452, 1433, 1327, 1306, 1242, 1179, 1161 cm^{-1}

^{1}H NMR (400 MHz, $CDCl_3$) δ 9.04 (br. s, 1H, OH), 7.53 – 7.30 (m, 7H, 7 x ArH), 6.99 (d, *J* = 8.8 Hz, 1.2H, 1.2 x ArH), 6.86 (d, *J* = 8.9 Hz, 0.8H, 0.8 x ArH), 3.87 (s, 1.8H. 1.8 x OCH_3), 3.82 (s, 1.2H, 1.2 x OCH_3) (41:59 mixture of stereoisomers)

^{13}C NMR (101 MHz, $CDCl_3$) δ 160.9, 160.3, 157.7, 157.7, 136.9, 133.1, 131.3, 129.5, 129.4, 129.3, 129.1, 128.9, 128.43, 128.35, 128.3, 124.8, 113.9, 113.7, 55.4 (mixture of stereoisomers)

HRMS (ESI) *m/z*: $[M+H]^+$ calculated for $C_{14}H_{14}NO_2$ 228.1024, found 228.1029

Consistent with previously reported data.[26]

Compound 12c, (4-(*tert*-butyl)phenyl)(phenyl)methanone oxime

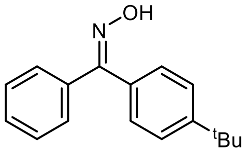

Synthesised according to General Procedure G using hydroxamoyl chloride **11b** (38.9 mg, 0.25 mmol), (4-(*tert*-butyl)phenyl)boronic acid (89.0 mg, 0.50 mmol), and DMA (158 µL, 1.25 mmol) to afford the product (44.8 mg, 71 %, >1:99 *E*:*Z* mixture) as a white solid.

ν_{max} (neat): 3279, 3225, 3208, 3194, 3132, 3086, 3080, 3055, 2961, 2932, 2905, 2866, 1609, 1508, 1495, 1449, 1439, 1395, 1364, 1327

^{1}H NMR (500 MHz, $CDCl_3$) δ 9.19 (br. s, 1H, OH), 7.51 – 7.47 (m, 4H, 4 x ArH), 7.41 (d, *J* = 8.3 Hz, 2H, 2 x ArH), 7.37 (d, *J* = 6.9 Hz, 1H, ArH), 7.35 – 7.32 (m, 2H, 2 x ArH), 1.38 (s, 9H, ^{t}Bu)

^{13}C NMR (126 MHz, $CDCl_3$) δ 158.1, 152.3, 136.7, 129.7, 129.5, 129.3, 128.4, 128.2, 125.3, 34.9, 31.4

HRMS (ESI) *m/z*: $[M+H]^+$ calculated for $C_{17}H_{20}NO$ 254.1545, found 254.1549

Compound 12d, phenyl(*p*-tolyl)methanone oxime

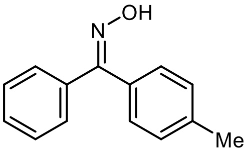

Synthesised according to General Procedure G using hydroxamoyl chloride **11b** (38.9 mg, 0.25 mmol), *p*-tolylboronic acid (68.0 mg, 0.50 mmol), and DMA (158 µL, 1.25 mmol) to afford the product (20.1 mg, 38 %, 9:91 *E*:*Z* mixture) as a white solid.

ν_{max} (neat): 3258, 3215, 3190, 3169, 3134, 3103, 3084, 3051, 3024, 2918, 1609, 1512, 1493, 1447, 1402, 1325, 1304 cm^{-1}

^{1}H NMR (500 MHz, $CDCl_3$) δ 8.66 (br. s, 1H, OH), 7.46 (d, *J* = 7.9 Hz, 2H, 2 x ArH), 7.38 – 7.24 (m, 7H, 7 x ArH), 2.41 (s, 2.7H, 2.7 x CH_3), 2.35 (s, 0.3H, 0.3 x CH_3) (9:91 mixture of stereoisomers)

^{13}C NMR (126 MHz, $CDCl_3$) δ 158.2, 139.3, 136.6, 129.8, 129.6, 129.4, 129.1, 128.4, 128.1, 21.6

HRMS (ESI) *m/z*: $[M+H]^+$ calculated for $C_{14}H_{14}NO$ 212.1075, found 212.1075

Consistent with previously reported data.[26]

Compound 12e, diphenylmethanone oxime

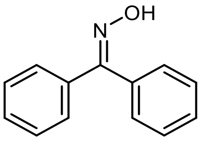

Synthesised according to General Procedure G using hydroxamoyl chloride **11b** (38.9 mg, 0.25 mmol), phenylboronic acid (61.0 mg, 0.50 mmol), and DMA (158 μL, 1.25 mmol) to afford the product (13.5 mg, 23 %) as a white solid.

ν_{max} (neat): 3211, 3188, 3177, 3132, 3057, 3026, 2953, 2922, 2887, 1491, 1445, 1429, 1329, 1300 cm^{-1}

^{1}H NMR (400 MHz, $CDCl_3$) δ 8.78 (br. s, 1H, OH), 7.53 – 7.40 (m, 7H, 7 x ArH), 7.40 – 7.30 (m, 3H, 3 x ArH)

^{13}C NMR (126 MHz, $CDCl_3$) δ 158.2, 136.4, 132.8, 129.7, 129.4, 129.3, 128.5, 128.4, 128.0

HRMS (ESI) *m/z*: $[M+H]^+$ calculated for $C_{13}H_{12}NO$ 198.0919, found 198.0917

Consistent with previously reported data.[26]

Compound 12f, (1-methyl-1*H*-indazol-5-yl)(phenyl)methanone oxime

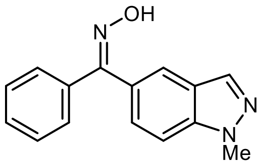

Synthesised according to General Procedure G using hydroxamoyl chloride **11b** (38.9 mg, 0.25 mmol), (1-methyl-1*H*-indazol-5-yl)boronic acid (88.0 mg, 0.50 mmol), and DMA (158 μL, 1.25 mmol) to afford the product (41.0 mg, 65 %, >1:99 *E*:*Z* mixture) as a white solid.

ν_{max} (neat): 3204, 3181, 3169, 3136, 3059, 3026, 2974, 2965, 2941, 2913, 2903, 2845, 1626, 1506, 1493, 1452, 1443, 1410, 1377, 1350, 1327, 1290 cm^{-1}

^{1}H NMR (500 MHz, $CDCl_3$) δ 9.36 (br. s, 1H, OH), 8.04 (s, 1H, ArH), 7.85 (s, 1H, ArH), 7.55 – 7.45 (m, 4H, 4 x ArH), 7.42 – 7.30 (m, 3H, 3 x ArH), 4.13 (s, 3H, CH_3)

^{13}C NMR (126 MHz, $CDCl_3$) δ 158.1, 139.9, 136.9, 133.6, 129.6, 128.5, 128.2, 127.9, 125.2, 123.8, 123.0, 108.8, 35.8

HRMS (ESI) *m/z*: $[M-H]^-$ calculated for $C_{15}H_{12}N_3O$ 250.0980, found 250.0988

Compound 12g, phenyl(thiophen-2-yl)methanone oxime

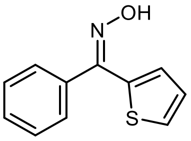

Synthesised according to General Procedure G using hydroxamoyl chloride **11b** (38.9 mg, 0.25 mmol), thiophen-2-ylboronic acid (64.0 mg, 0.50 mmol), and DMA (158 μL, 1.25 mmol) to afford the product (11.8 mg, 23 %, >1:99 *E*:*Z* mixture) as a white solid.

ν_{max} (neat): 3086, 3057, 3026, 2980, 2953, 2924, 2816, 2772, 2756, 1607, 1591, 1576, 1497, 1437, 1414, 1323, 1227 cm^{-1}

^{1}H NMR (500 MHz, $CDCl_3$) δ 9.76 (br. s, 1H, OH), 7.61 (d, *J* = 5.1 Hz, 1H, ArH), 7.55 (d, *J* = 7.7 Hz, 2H, 2 x ArH), 7.48 – 7.41 (m, 3H, 3 x ArH), 7.24 (d, *J* = 3.8 Hz, 1H, ArH), 7.06 (app. t, *J* = 4.4 Hz, 1H, ArH)

^{13}C NMR (126 MHz, $CDCl_3$) δ 152.0, 136.3, 133.0, 132.3, 131.3, 129.44, 129.42, 128.5, 125.8

HRMS (ESI) *m/z*: $[M+H]^+$ calculated for $C_{11}H_{10}NOS$ 204.0483, found 204.0479

Consistent with previously reported data.[27]

Compound 12h, furan-3-yl(phenyl)methanone oxime

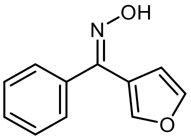

Synthesised according to General Procedure G using hydroxamoyl chloride **11b** (38.9 mg, 0.25 mmol), furan-3-ylboronic acid (55.9 mg, 0.50 mmol), and DMA (158 μL, 1.25 mmol) to afford the product (12.9 mg, 28 %, >1:99 *E*:*Z* mixture) as a white solid.

ν_{max} (neat): 3265, 3179, 3165, 3156, 3129, 3105, 3075, 3057, 3030, 2918, 1562, 1508, 1491, 1445, 1356, 1323, 1233 cm^{-1}

^{1}H NMR (500 MHz, $CDCl_3$) δ 9.66 (br. s, 1H, OH), 8.16 (s, 1H, ArH), 7.54 (d, *J* = 7.8 Hz, 2H, 2 x ArH), 7.48 – 7.39 (m, 4H, 4 x ArH), 6.60 (s, 1H, ArH)

^{13}C NMR (126 MHz, $CDCl_3$) δ 150.9, 147.5, 142.1, 136.2, 129.4, 128.8, 128.5, 117.3, 111.8

HRMS (ESI) *m/z*: $[M+H]^+$ calculated for $C_{11}H_{10}NO_2$ 188.0712, found 188.0708

Compound 12i, (4-(dimethylamino)phenyl)(phenyl)methanone oxime

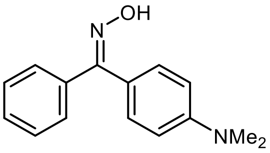

Synthesised according to General Procedure G using hydroxamoyl chloride **11b** (38.9 mg, 0.25 mmol), (4-(dimethylamino)phenyl)boronic acid (82.5 mg, 0.50 mmol), and DMA (158 μL, 1.25 mmol) to afford the product (23.1 mg, 39 %, 38:62 *E*:*Z* mixture) as a white solid.

ν_{max} (neat): 3211, 3186, 3167, 3156, 3140, 3130, 3113, 3073, 3024, 3003, 2992, 2893, 2812, 1607, 1526, 1485, 1445, 1425, 1412, 1362, 1331, 1202, 1231, 1202, 1155 cm^{-1}

^{1}H NMR (500 MHz, $CDCl_3$) δ 8.17 (s, 0.6H, 0.6 x OH), 7.94 (s, 0.4H, 0.4 x OH), 7.55 – 7.28 (m, 7H, 7 x ArH), 6.75 (d, *J* = 8.0 Hz, 1.2H, 1.2 x ArH), 6.64 (d, *J* = 8.0 Hz, 0.8H, 0.8 x ArH), 3.02 (s, 3.6H, 3.6 x NMe_2), 2.98 (s, 2.4H, 2.4 x NMe_2) (38:62 mixture of stereoisomers)

^{13}C NMR (101 MHz, $CDCl_3$) δ 158.5, 158.3, 151.4, 150.9, 137.5, 133.5, 131.3, 129.2, 128.9, 128.8, 128.6, 128.3, 123.9, 119.7, 111.8, 111.4, 40.4 (mixture of stereoisomers)

HRMS (ESI) *m/z*: $[M+H]^+$ calculated for $C_{15}H_{17}N_2O$ 241.1341, found 241.1341

5 X-Ray Crystallography

Crystallographic measurements were made at 150(2) K with an Oxford Diffraction Gemini S diffractometer and monochromated Cu radiation (λ = 1.54184 Å). Programs from the SHELX suite were used for structure solution and refinement.[28] Refinement was to convergence against F^2 using all unique reflections. Non-H atoms were refined anisotropically. All H atoms bound to C were observed in difference maps but were included in the final model as riding atoms. The H atom bound to O was refined freely and isotropically. The final model is shown in *Figure S 3* and selected crystallographic and refinement parameters are given in *Table S 11*. CCDC 1987300 contains the supplementary crystallographic data for this structure. These data can be obtained free of charge from the Cambridge Crystallographic Data Centre via www.ccdc.cam.ac.uk/data_request/cif.

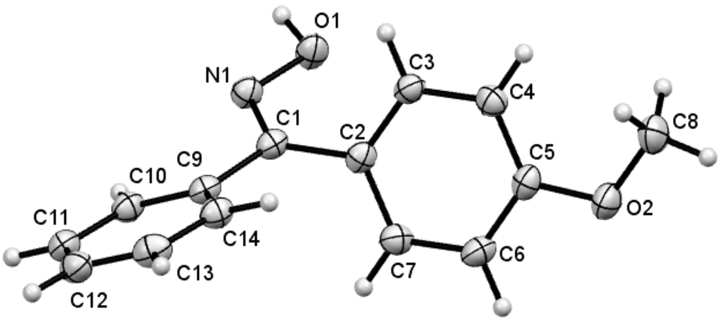

*Figure S 3: Molecular structure of compound **12b** with non-H atoms shown as 50% probability ellipsoids and H atoms as small spheres of arbitrary size*

Table S 11: Selected crystallographic and refinement parameters

**Compound**	12b	***Z***	4
**Formula**	$C_{14}H_{13}NO_2$	**Refls. Collected**	3911
**Molecular Weight**	227.25	**Refls. Independant**	2095
**Crystal system**	Monoclinic	**Refls. Observed**	1880
**Space Group**	$P2_1/c$	**Rint**	0.0191
**λ Å**	1.54184	**No. Parameters**	160
a **Å**	9.9101(3)	**Goodness of Fit**	1.043
b **Å**	9.4677(3)	**R[I>2σ(I)], *F***	0.0410
c **Å**	12.2514(4)	**Rw, F^2**	0.1166
β **°**	94.841(3)	**Residual *e* density Max. eÅ^{-3}**	0.217
**Volume Å^3**	1145.40(6)	**Residual *e* density Min. eÅ^{-3}**	-0.291
**Temp. *K***	150(2)	**Extinction coeff.**	0.0132(13)

6 References

[1] S. Stewart, R. Harris, C. Jamieson, *Synlett* **2014**, *25*, 2480–2484.

[2] Z. Guo, H. Jia, H. Liu, Q. Wang, J. Huang, H. Guo, *Org. Lett.* **2018**, *20*, 2939–2943.

[3] M. Tóth, S. Kun, É. Bokor, M. Benltifa, G. Tallec, S. Vidal, T. Docsa, P. Gergely, L. Somsák, J. P. Praly, *Bioorganic Med. Chem.* **2009**, *17*, 4773–4785.

[4] K. Livingstone, S. Bertrand, J. Mowat, C. Jamieson, *Chem. Sci.* **2019**, *10*, 10412–10416.

[5] B. Durakovic, *Period. Eng. Nat. Sci.* **2017**, *5*, 421–439.

[6] B. Török, A. Sood, S. Bag, R. Tulsan, S. Ghosh, D. Borkin, A. R. Kennedy, M. Melanson, R. Madden, W. Zhou, et al., *Biochemistry* **2013**, *52*, 1137–1148.

[7] G. Wang, X. Liu, T. Huang, Y. Kuang, L. Lin, X. Feng, *Org. Lett.* **2013**, *15*, 76–79.

[8] X. Deng, N. S. Mani, *J. Org. Chem.* **2008**, *73*, 2412–2415.

[9] V. V. Voronin, M. S. Ledovskaya, E. G. Gordeev, K. S. Rodygin, V. P. Ananikov, N. D. Zelinsky, *J. Org. Chem.* **2018**, *83*, 3819–3828.

[10] M. J. Kornet, T. T. Tita, A. P. Thio, *Synth. Commun.* **1986**, *16*, 1261–1274.

[11] C. Peifer, M. Abadleh, J. Bischof, D. Hauser, V. Schattel, H. Hirner, U. Knippschild, S. Laufer, *J. Med. Chem.* **2009**, *52*, 7618–7630.

[12] A. V. Dubrovskiy, R. C. Larock, *Org. Lett.* **2010**, *12*, 1180–1183.

[13] R. Fusco, F. Sannicolo, *J. Org. Chem.* **1981**, *46*, 83–89.

[14] W. Wei, Z. Wang, X. Yang, W. Yu, J. Chang, *Adv. Synth. Catal.* **2017**, *359*, 3378–3387.

[15] J. Yu, J. W. Lim, S. Y. Kim, J. Kim, J. N. Kim, *Tetrahedron Lett.* **2015**, *56*, 1432–1436.

[16] C. Mauger, G. Mignani, *Synth. Commun.* **2006**, *36*, 1123–1129.

[17] T. Zhang, W. Bao, *J. Org. Chem.* **2013**, *78*, 1317–1322.

[18] M. G. B. Drew, G. R. Willey, *J. Chem. Soc., Perkin Trans. 2* **1986**, 215–220.

[19] J. Hu, H. Xu, P. Nie, X. Xie, Z. Nie, Y. Rao, *Chem. - A Eur. J.* **2014**, *20*, 3932–3938.

[20] S. D. Sharma, S. B. Pandhi, *J. Org. Chem.* **1990**, *55*, 2196–2200.

[21] W. Wu, X. H. Fan, L. P. Zhang, L. M. Yang, *RSC Adv.* **2014**, *4*, 3364–3367.

[22] W. Wu, X.-H. Fan, L.-P. Zhang, L.-M. Yang, *RSC Adv.* **2014**, *4*, 3364–3367.

[23] S. Kanta De, *Synth. Commun.* **2004**, *34*, 4409–4415.

[24] K. O. Jeon, J. H. Jun, J. S. Yu, C. K. Lee, *J. Heterocycl. Chem.* **2003**, *40*, 763–771.

[25] W. J. Kerr, D. M. Lindsay, P. K. Owens, M. Reid, T. Tuttle, S. Campos, *ACS Catal.* **2017**, *7*, 7182–7186.

[26] W. Zhang, S. Yang, Q. Lin, H. Cheng, J. Liu, *J. Org. Chem.* **2019**, *84*, 851–859.

[27] A. Sasse, H. Stark, X. Ligneau, S. Elz, S. Reidemeister, C. R. Ganellin, J. C. Schwartz, W. Schunack, *Bioorganic Med. Chem.* **2000**, *8*, 1139–1149.

[28] G. M. Sheldrick, *Acta Crystallogr. Sect. A Found. Crystallogr.* **2015**, *71*, 3–8.

7 NMR Spectra of Compounds

^1H NMR (400 MHz, $CDCl_3$)

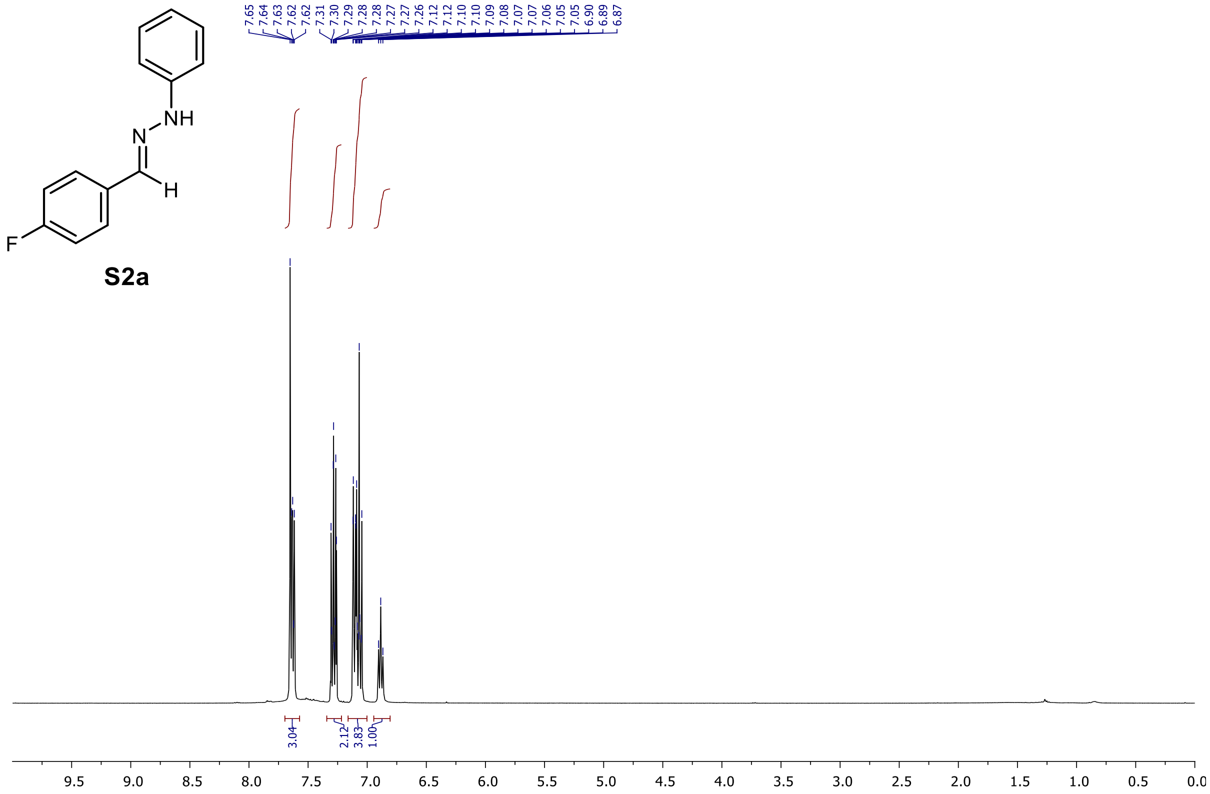

^{19}F NMR (376 MHz, $CDCl_3$)

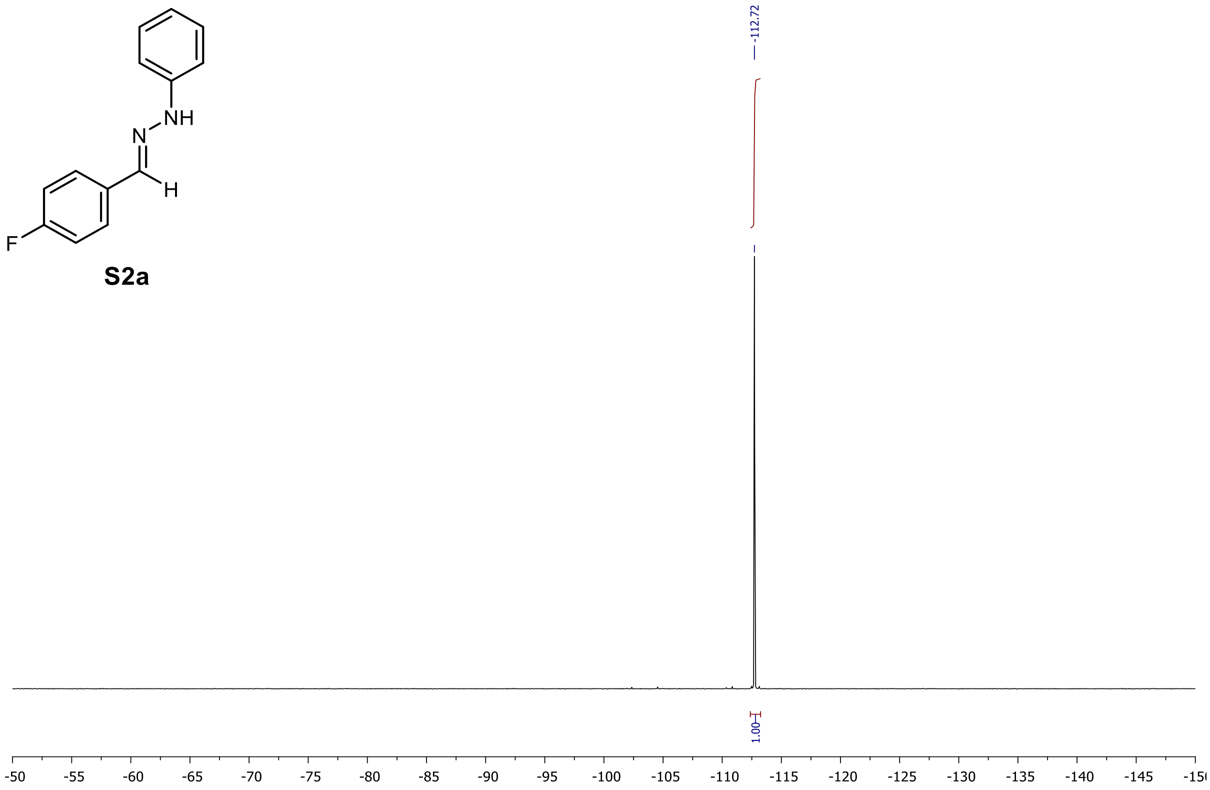

^{13}C NMR (101 MHz, $CDCl_3$)

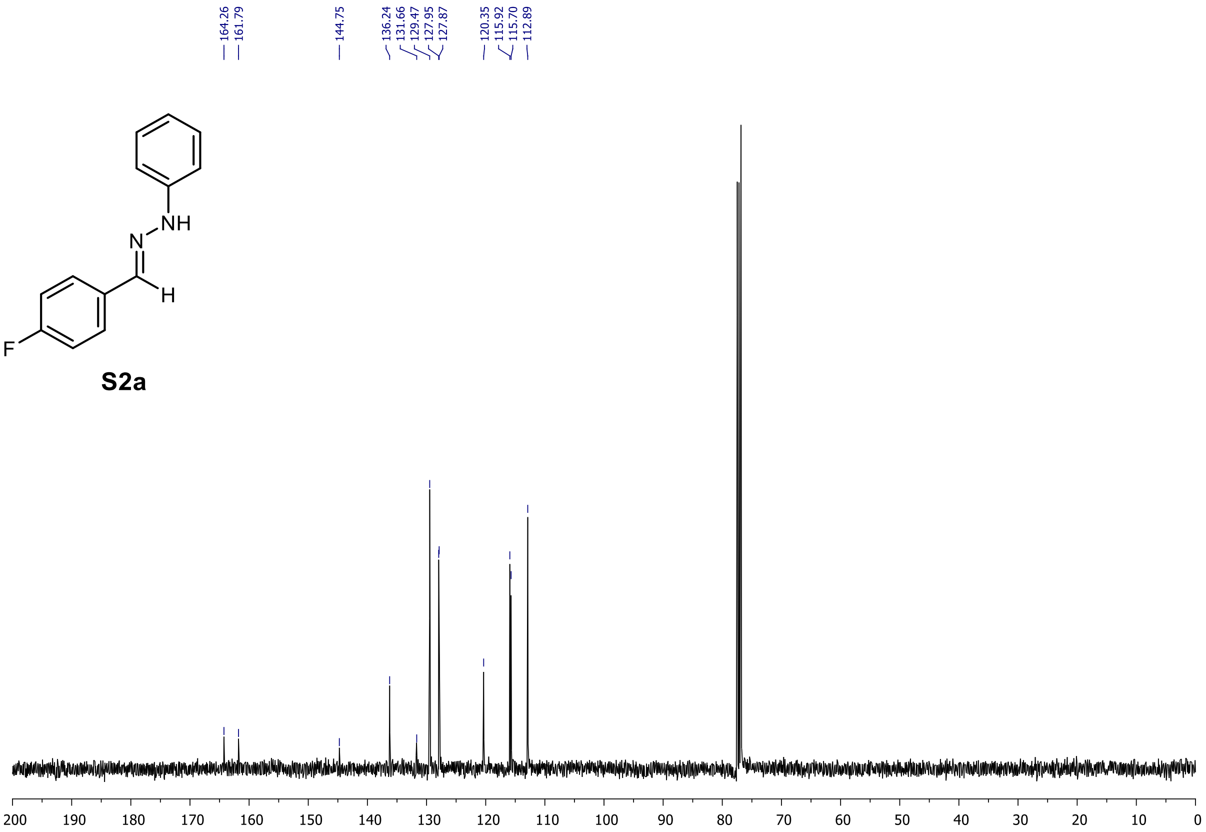

^{1}H NMR (400 MHz, $CDCl_3$)

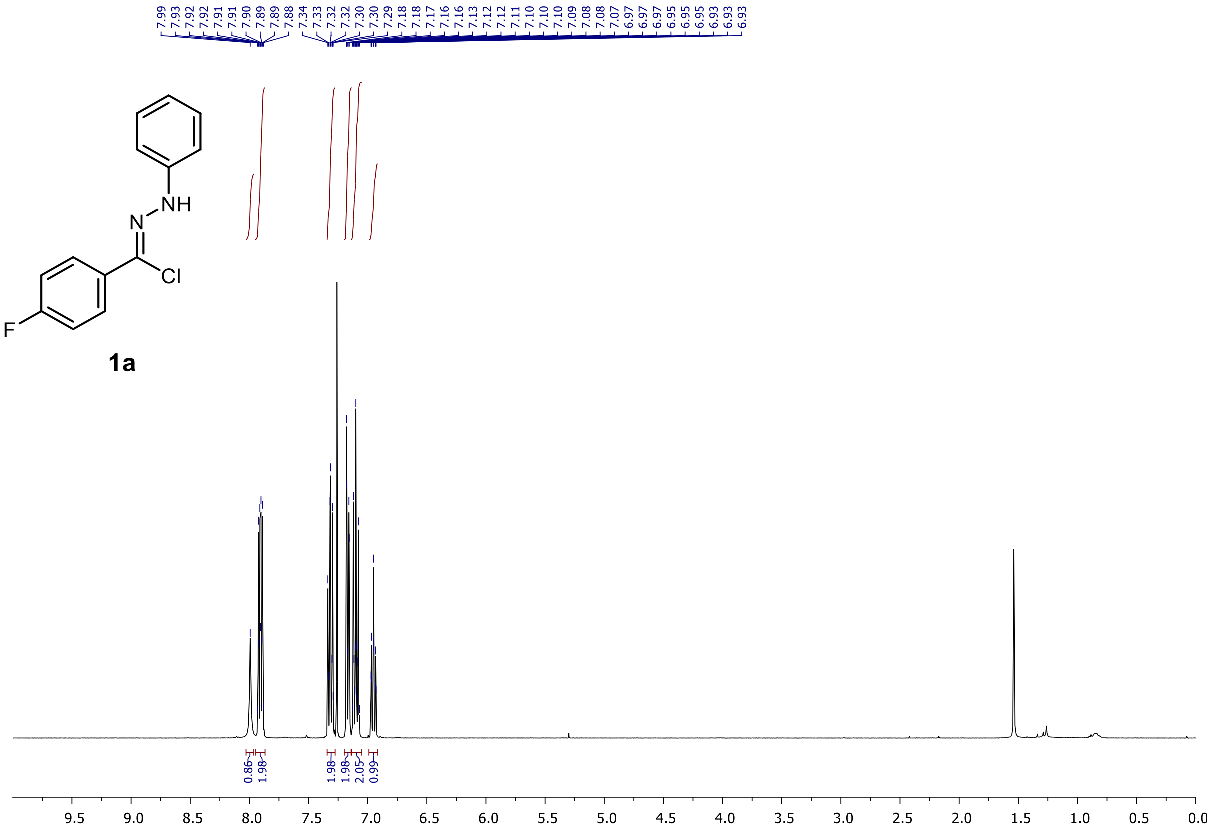

^{19}F NMR (471 MHz, $CDCl_3$)

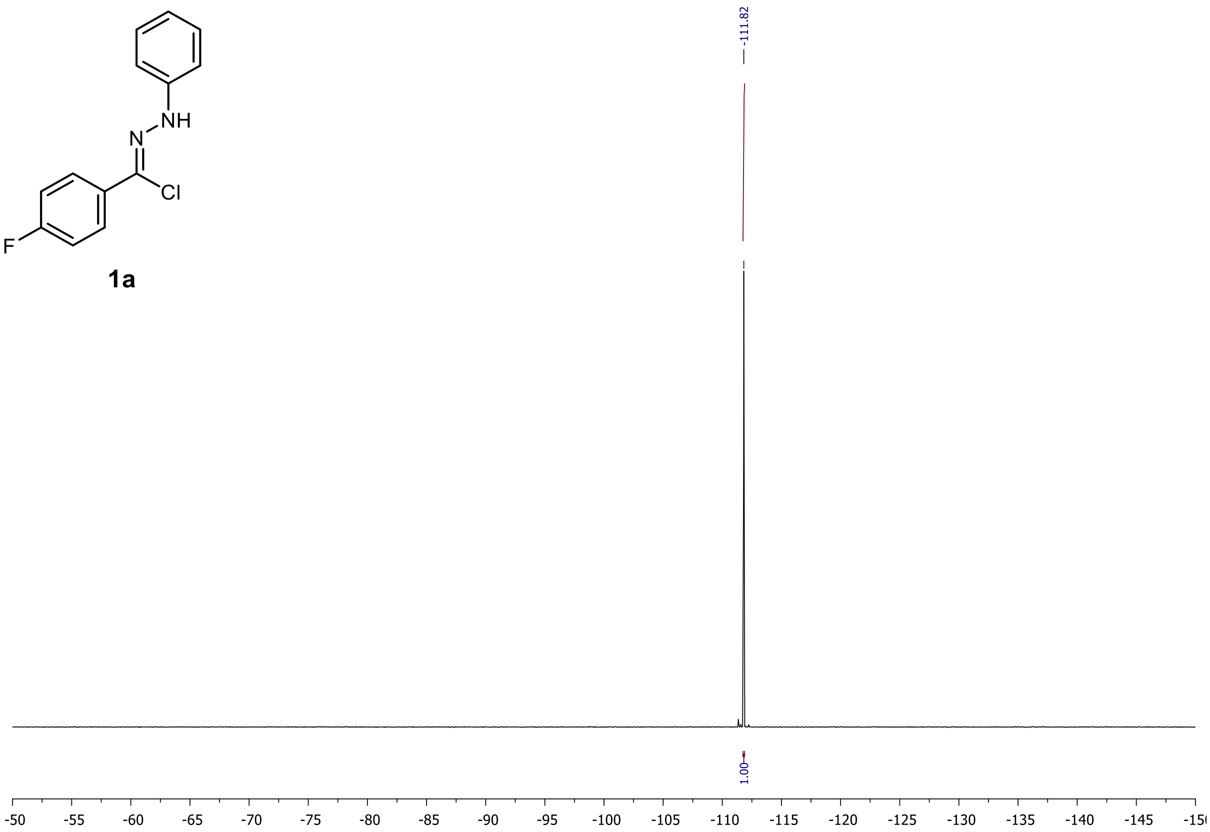

^{13}C NMR (101 MHz, $CDCl_3$)

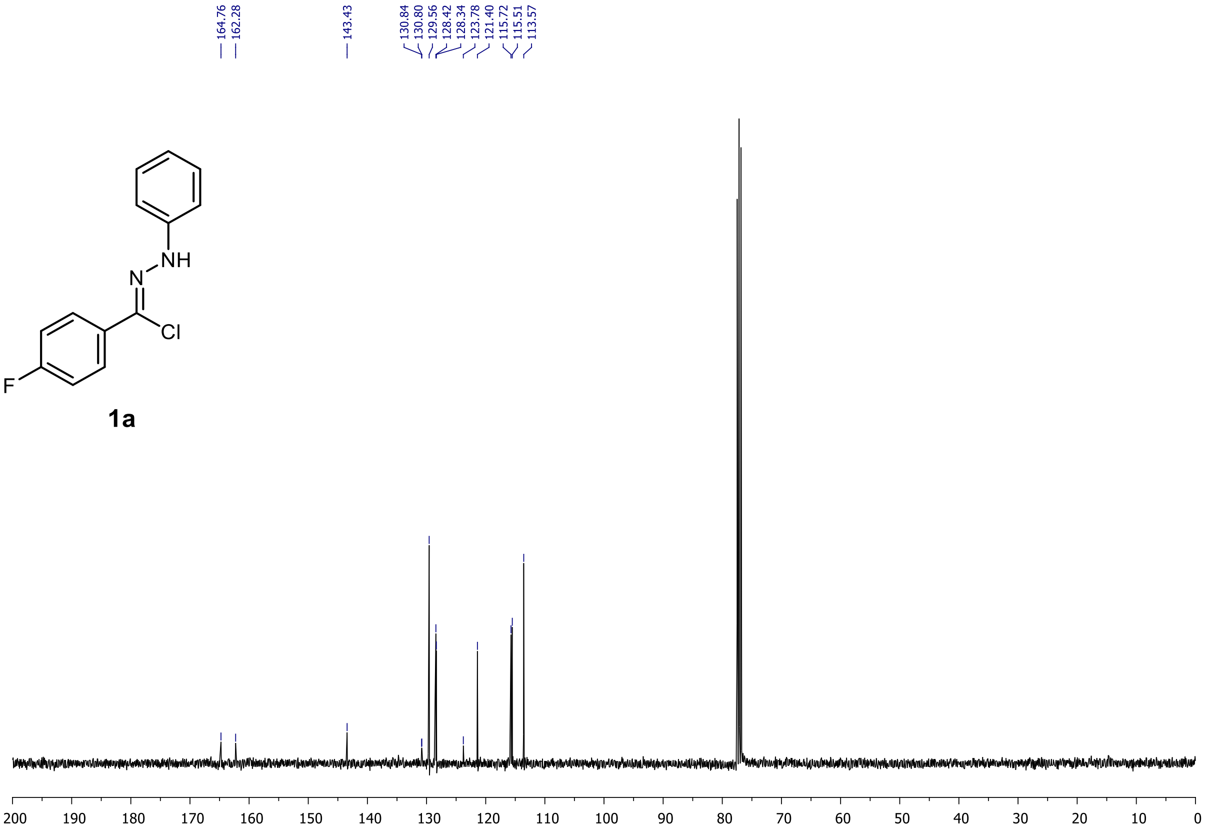

^{1}H NMR (400 MHz, $CDCl_3$)

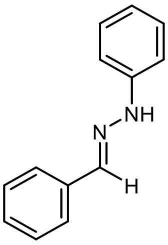

S2b

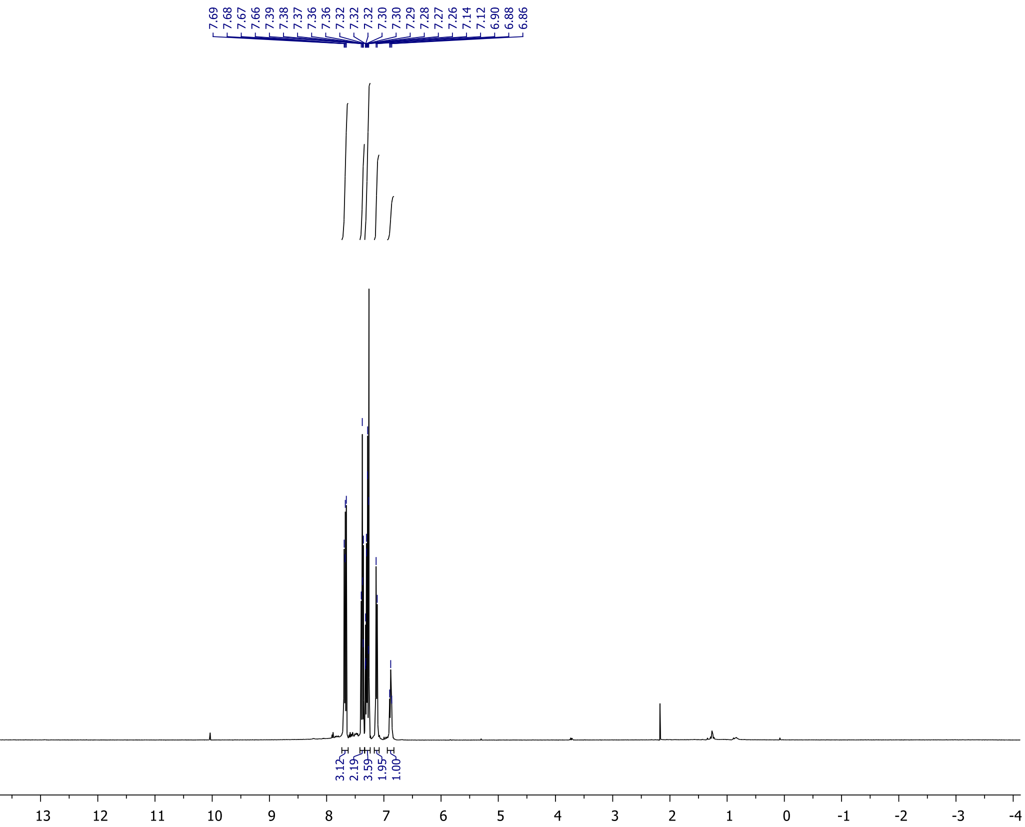

^{13}C NMR (101 MHz, $CDCl_3$)

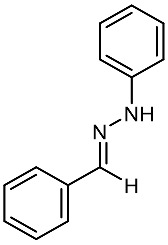

S2b

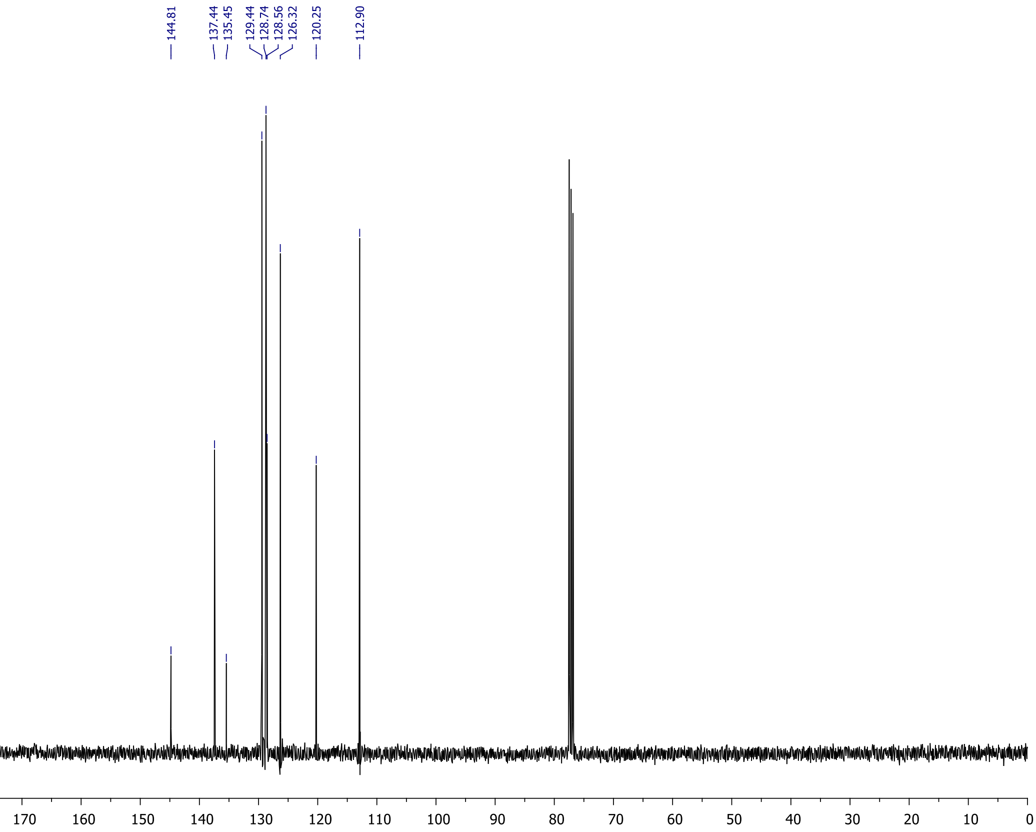

^{1}H NMR (400 MHz, $CDCl_3$)

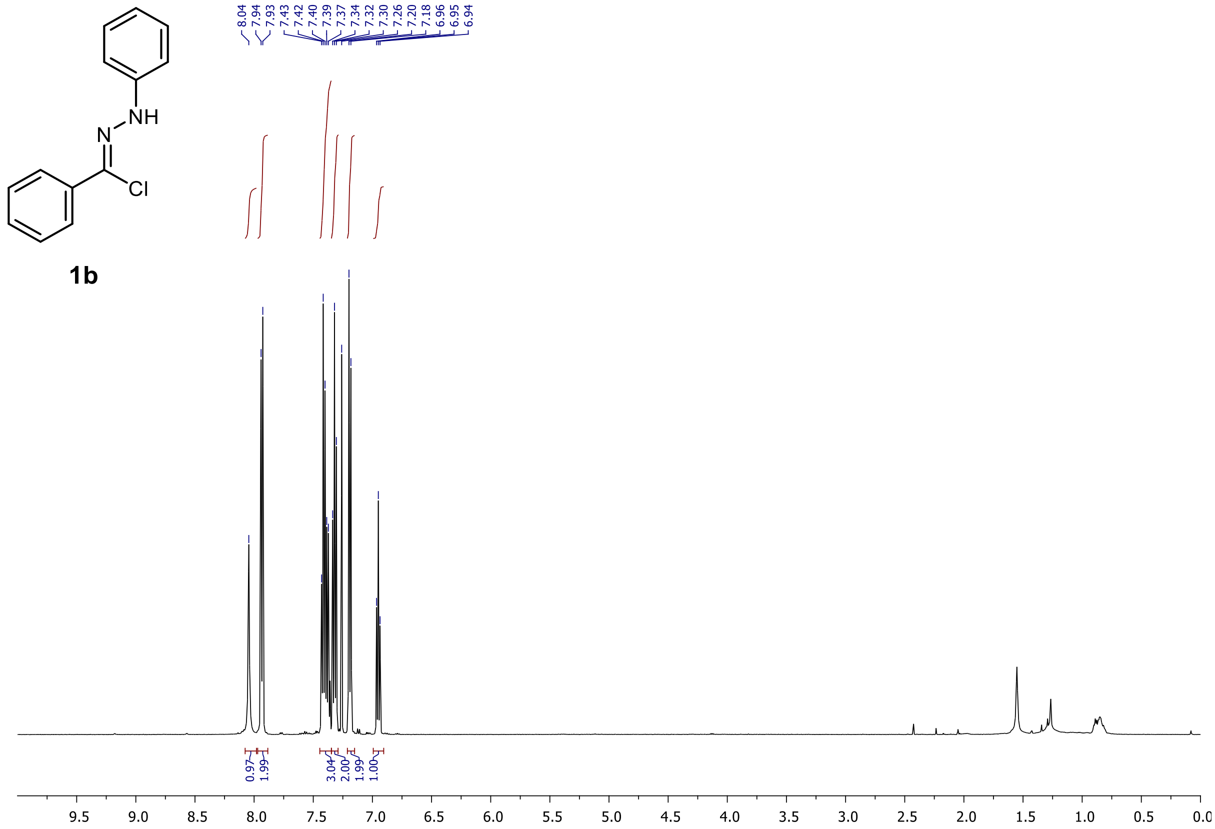

^{13}C NMR (101 MHz, $CDCl_3$)

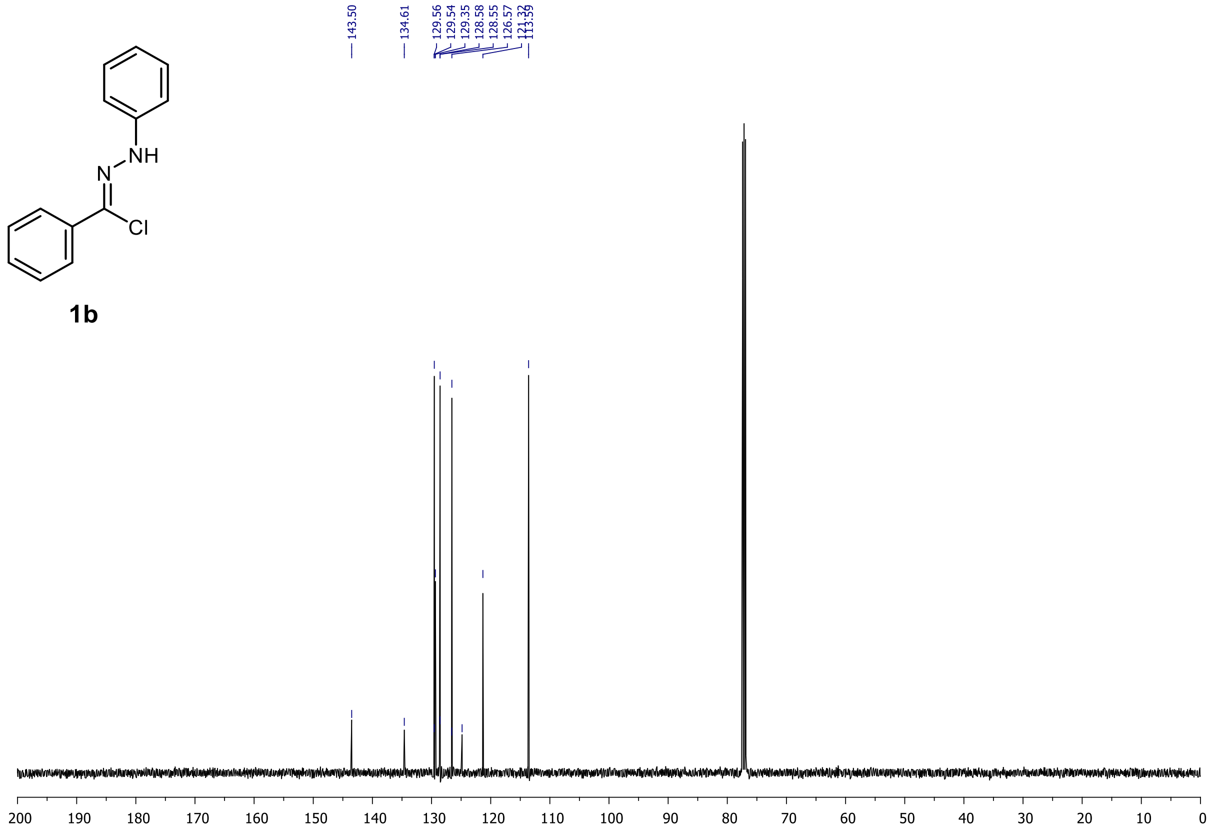

^{1}H NMR (400 MHz, $CDCl_3$)

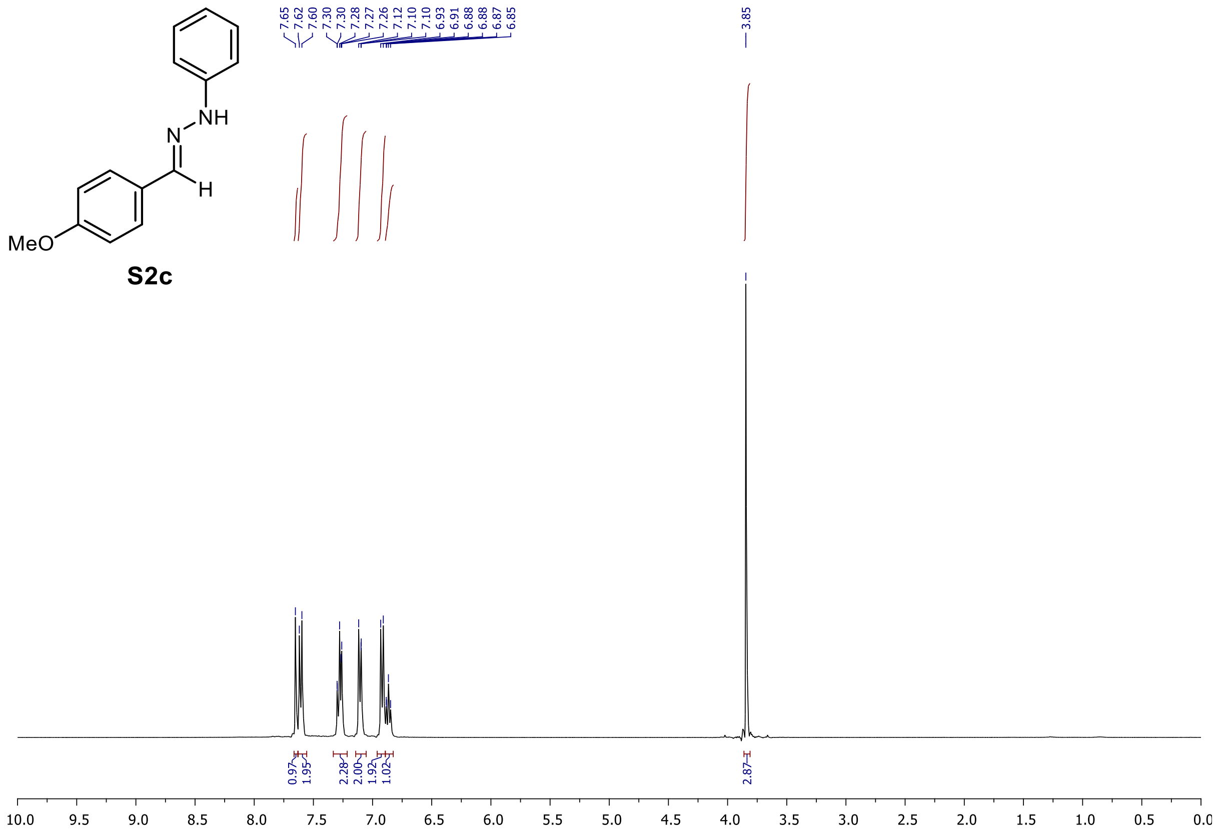

^{13}C NMR (101 MHz, $CDCl_3$)

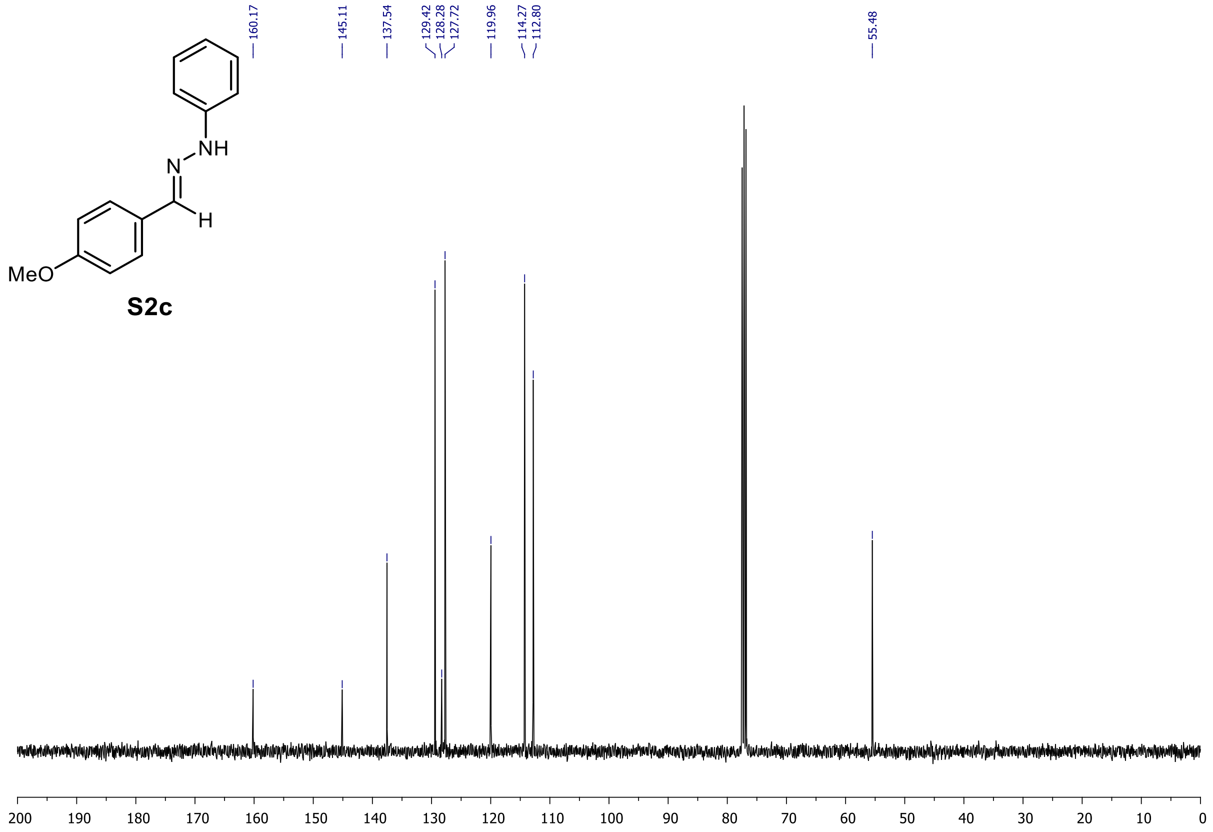

^{1}H NMR (400 MHz, $CDCl_3$)

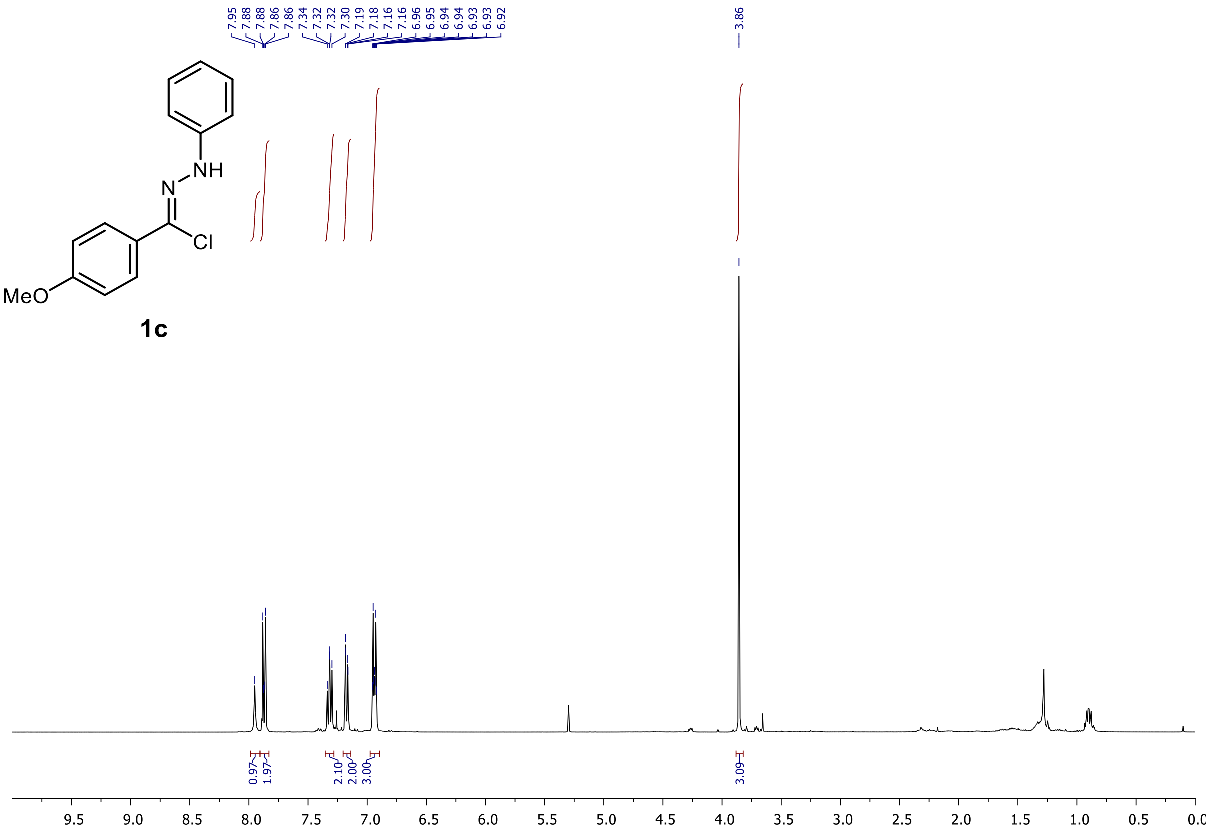

^{13}C NMR (101 MHz, $CDCl_3$)

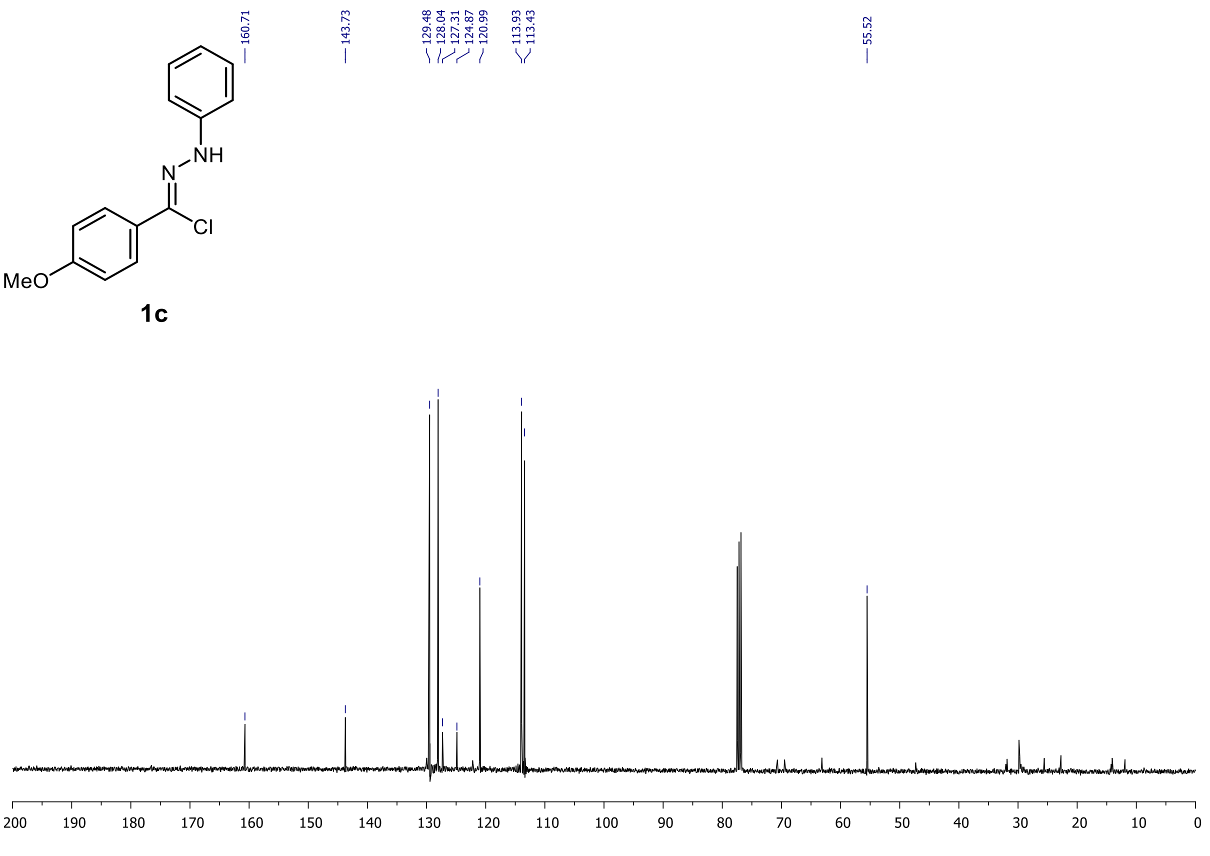

^{1}H NMR (500 MHz, $CDCl_3$)

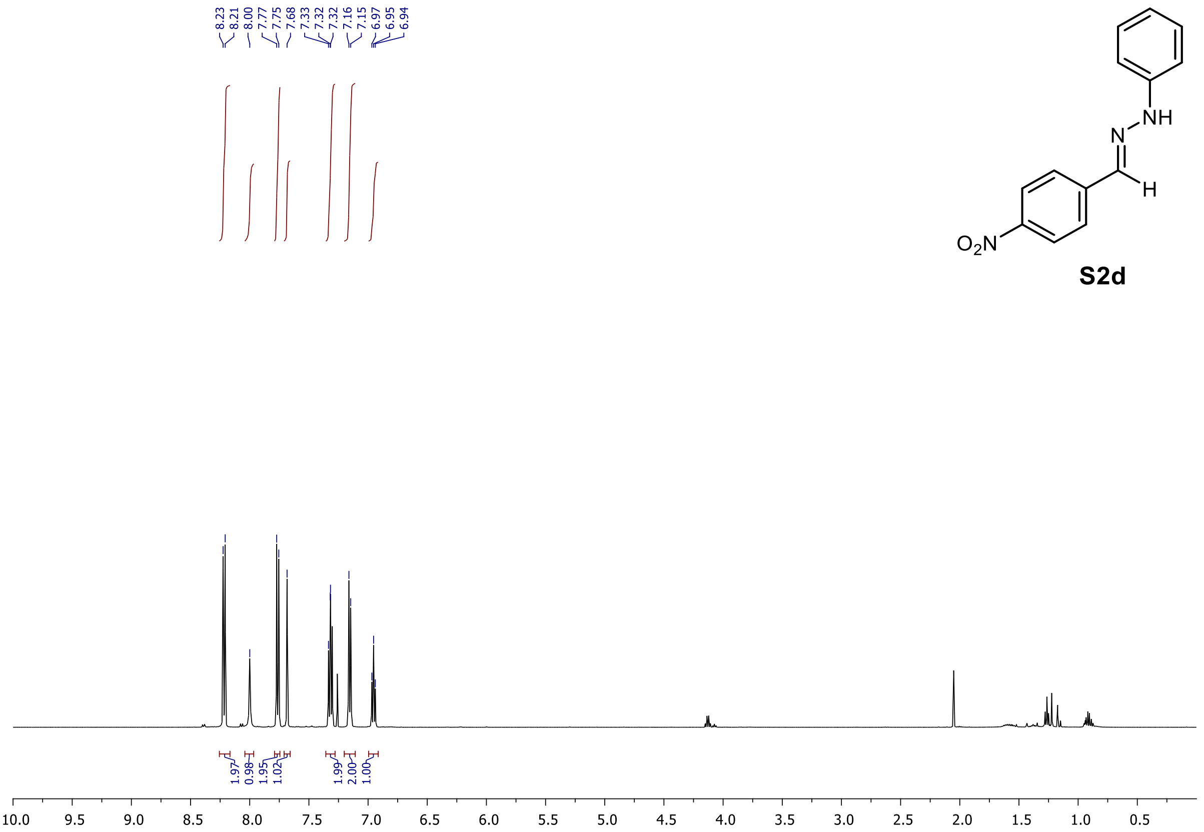

S2d

^{13}C NMR (101 MHz, $CDCl_3$)

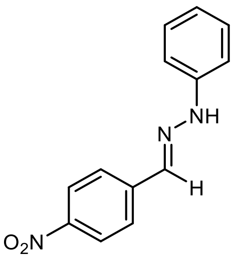

S2d

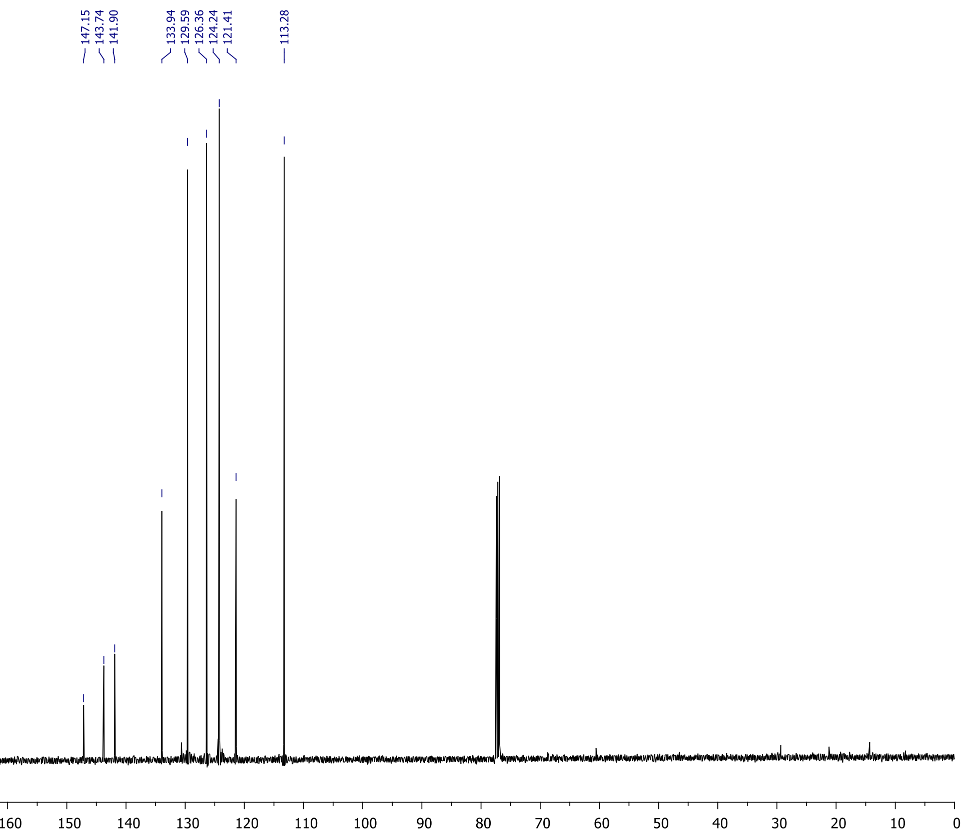

^{1}H NMR (400 MHz, $CDCl_3$)

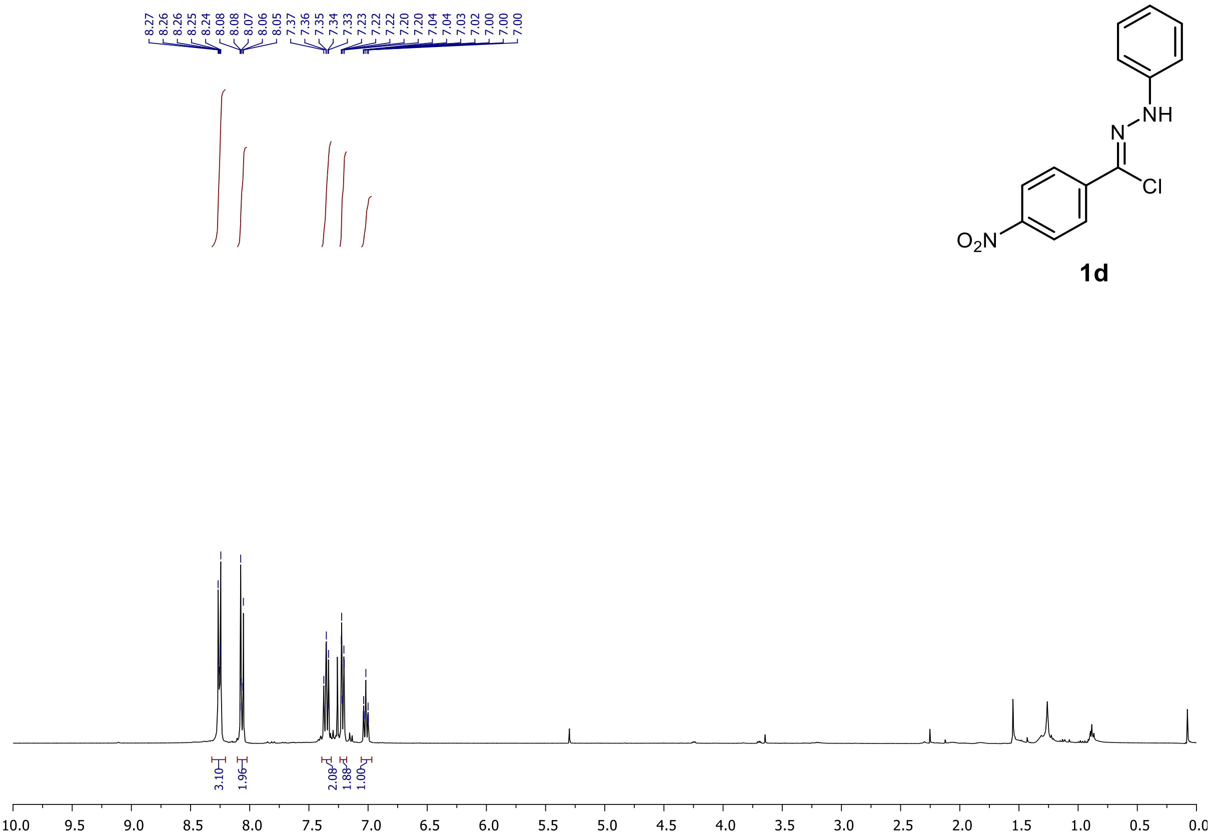

^{13}C NMR (101 MHz, $CDCl_3$)

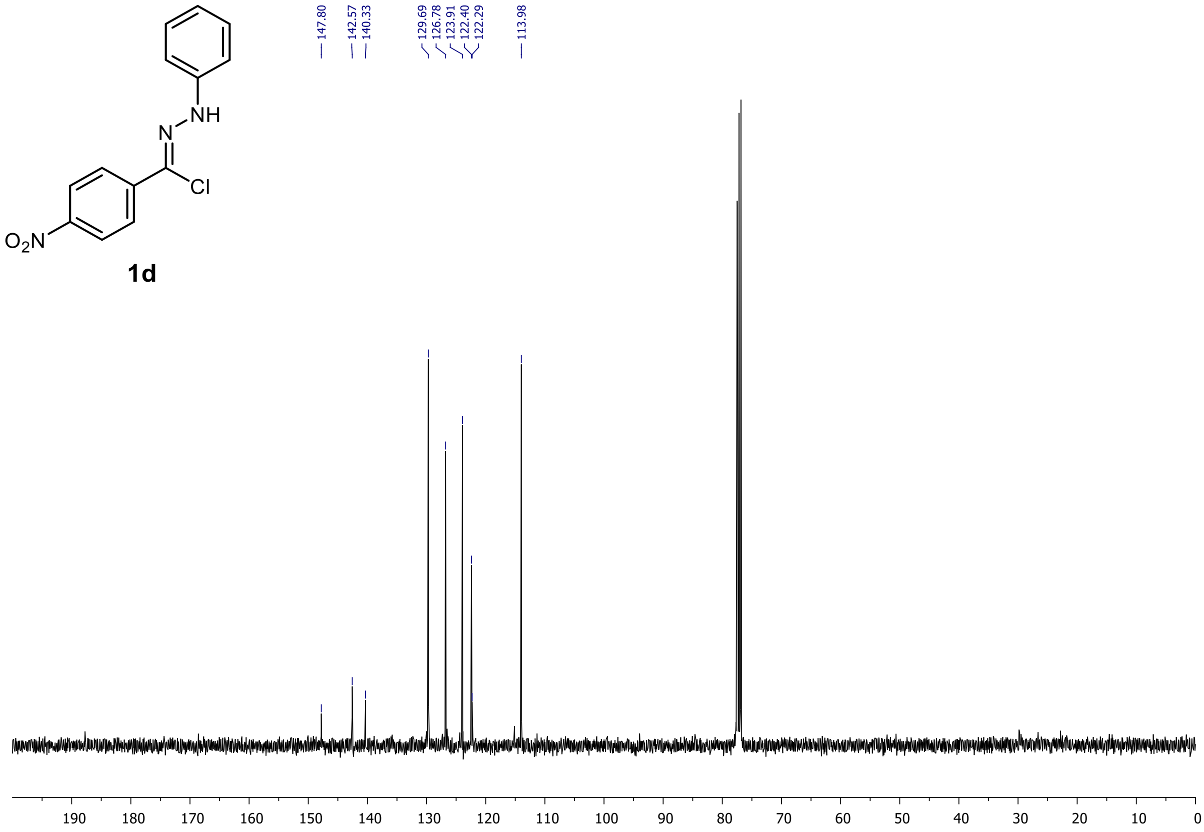

^{1}H NMR (400 MHz, DMSO-d_6), 80 °C

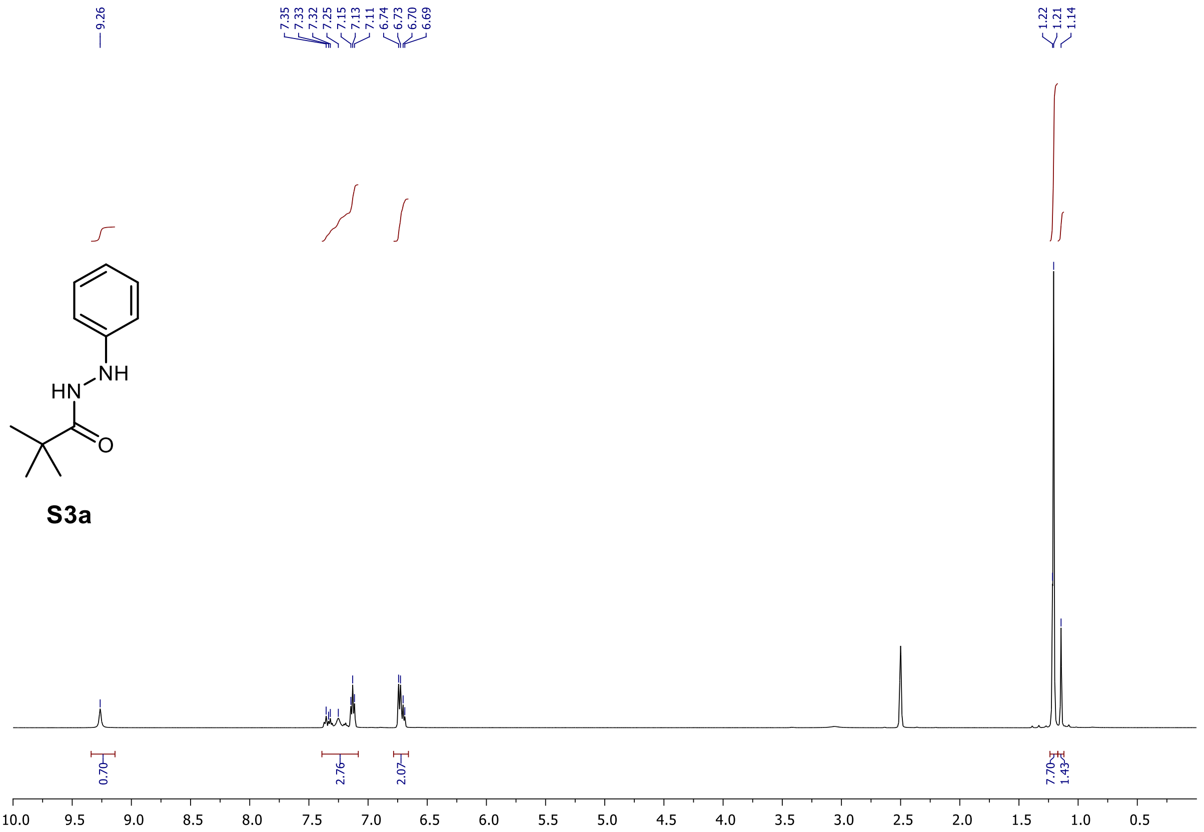

^{13}C NMR (101 MHz, DMSO-d_6), 80 °C

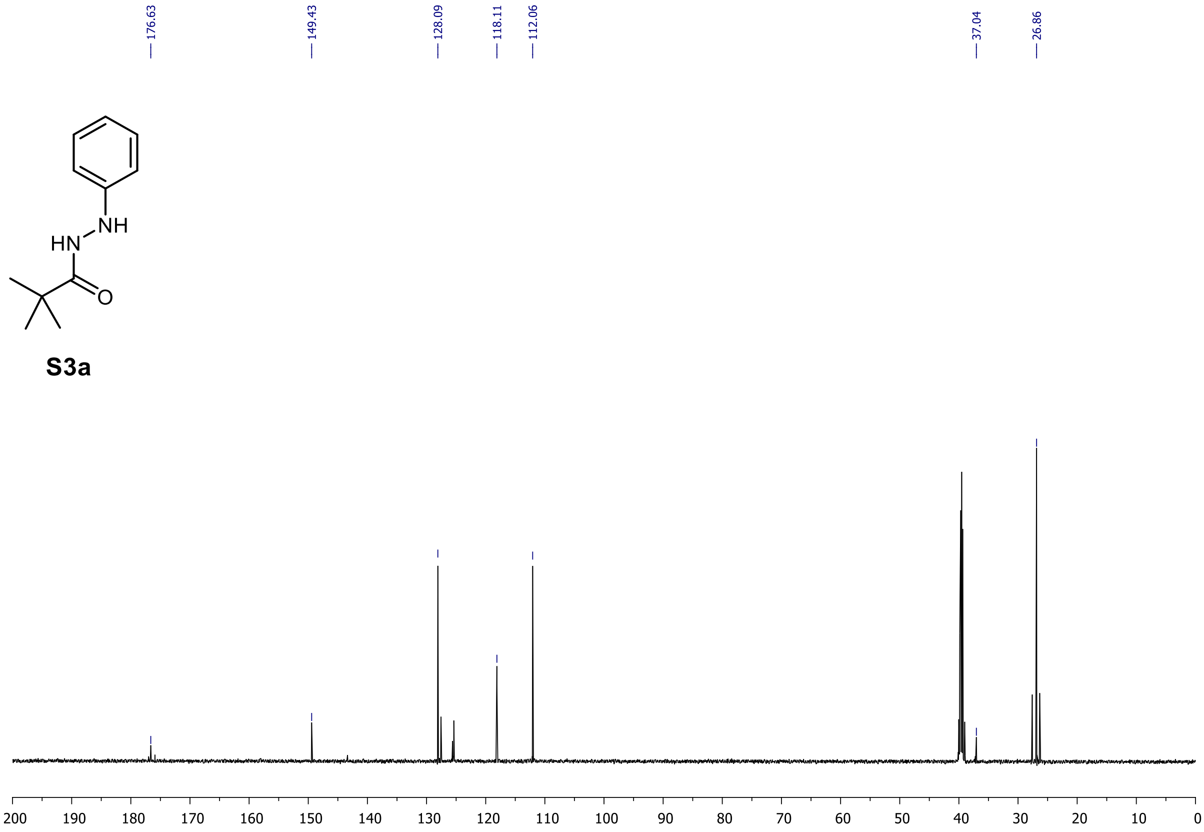

^{1}H NMR (500 MHz, $CDCl_3$)

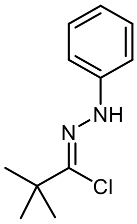

1e

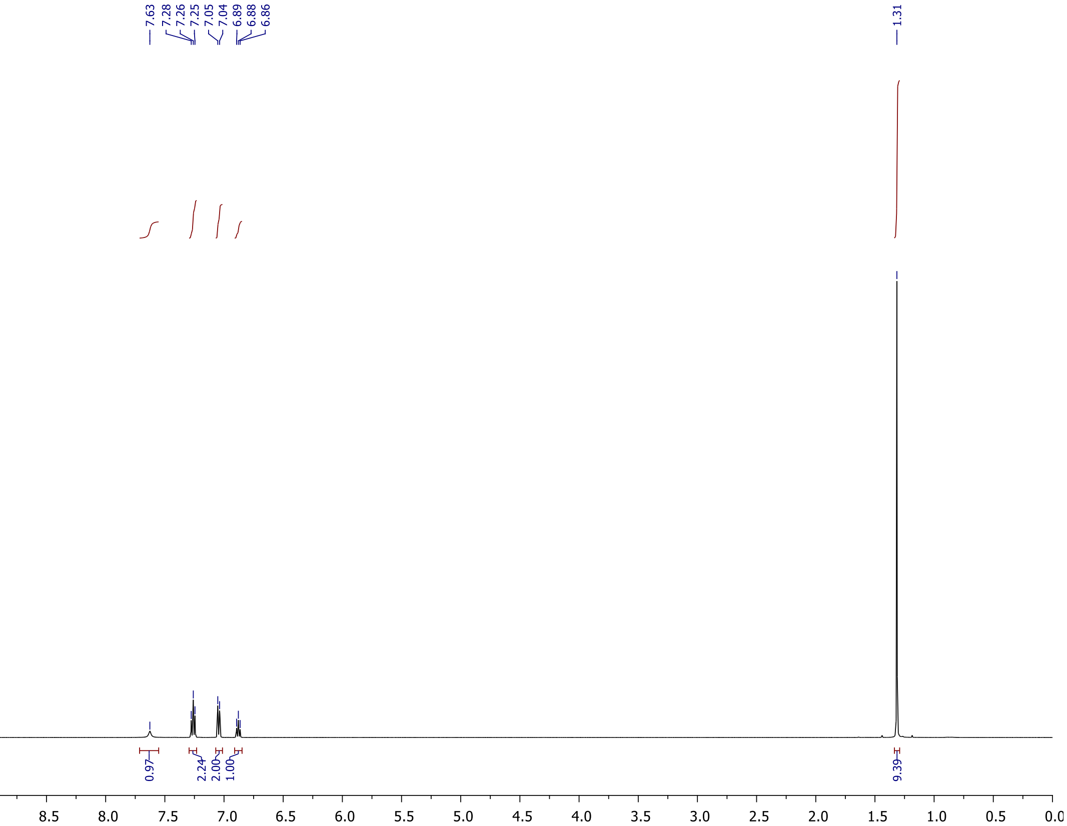

^{13}C NMR (126 MHz, $CDCl_3$)

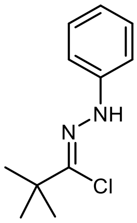

1e

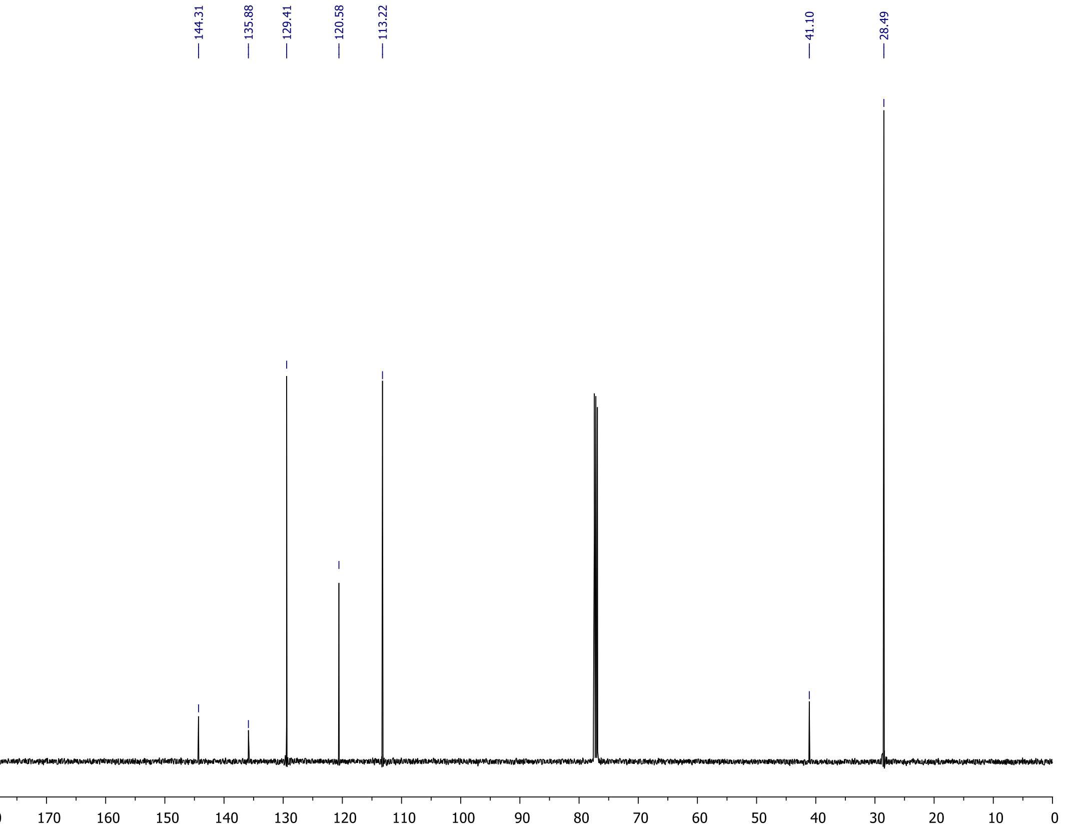

^{1}H NMR (400 MHz, DMSO-d_6), 80 °C

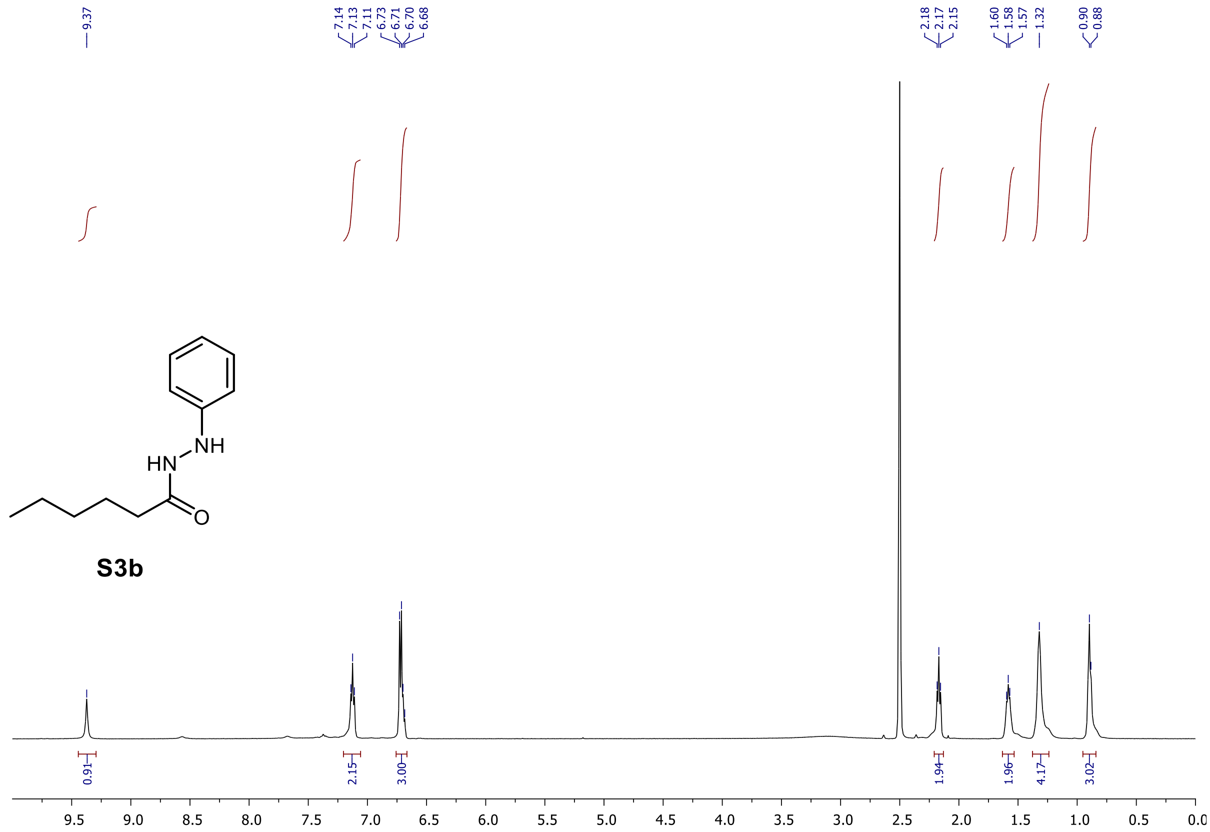

^{13}C NMR (101 MHz, DMSO-d_6), 80 °C

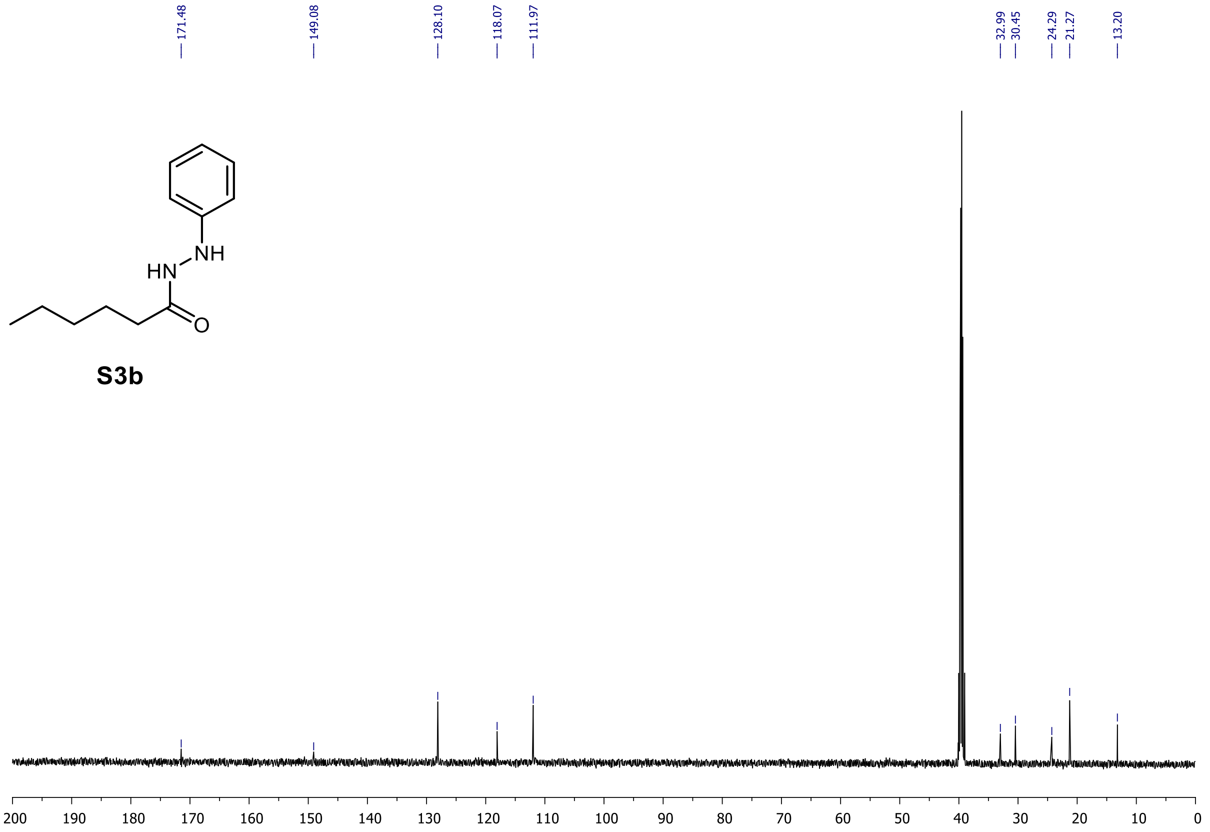

^{1}H NMR (500 MHz, $CDCl_3$)

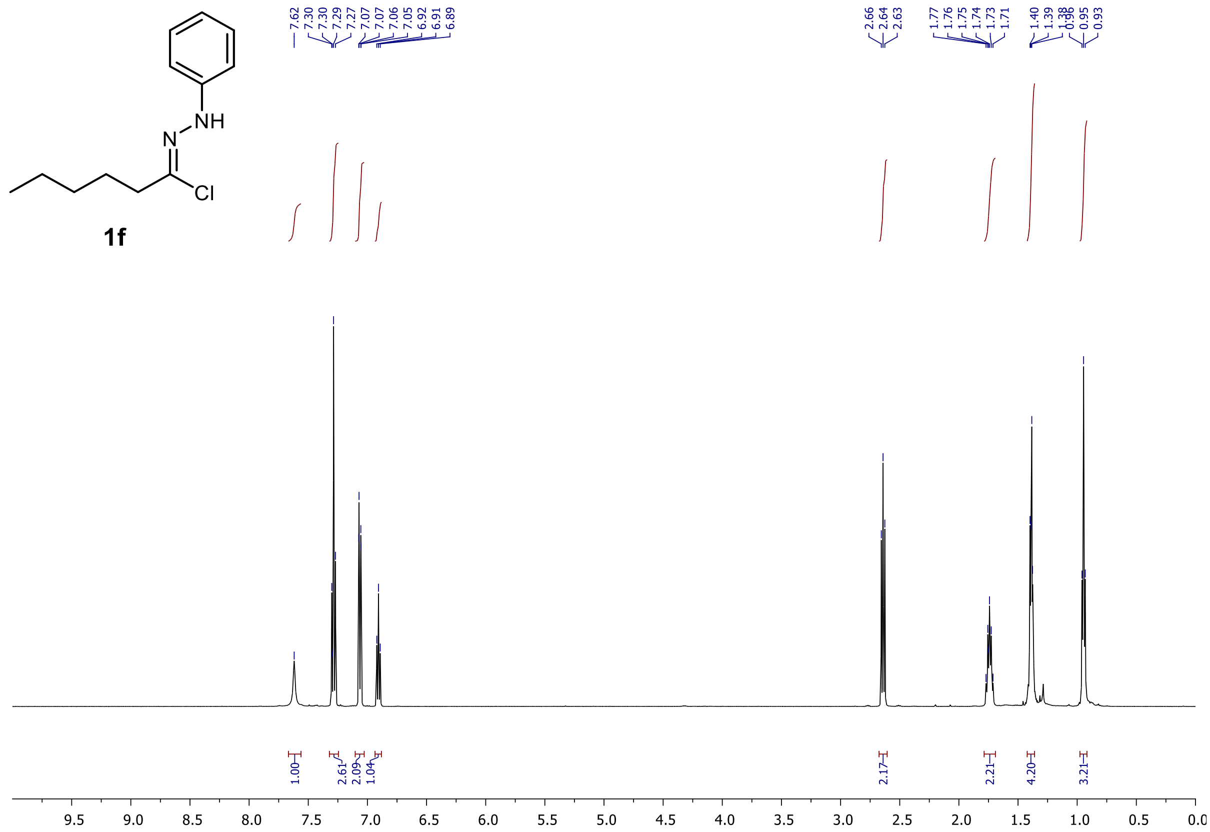

^{13}C NMR (126 MHz, $CDCl_3$)

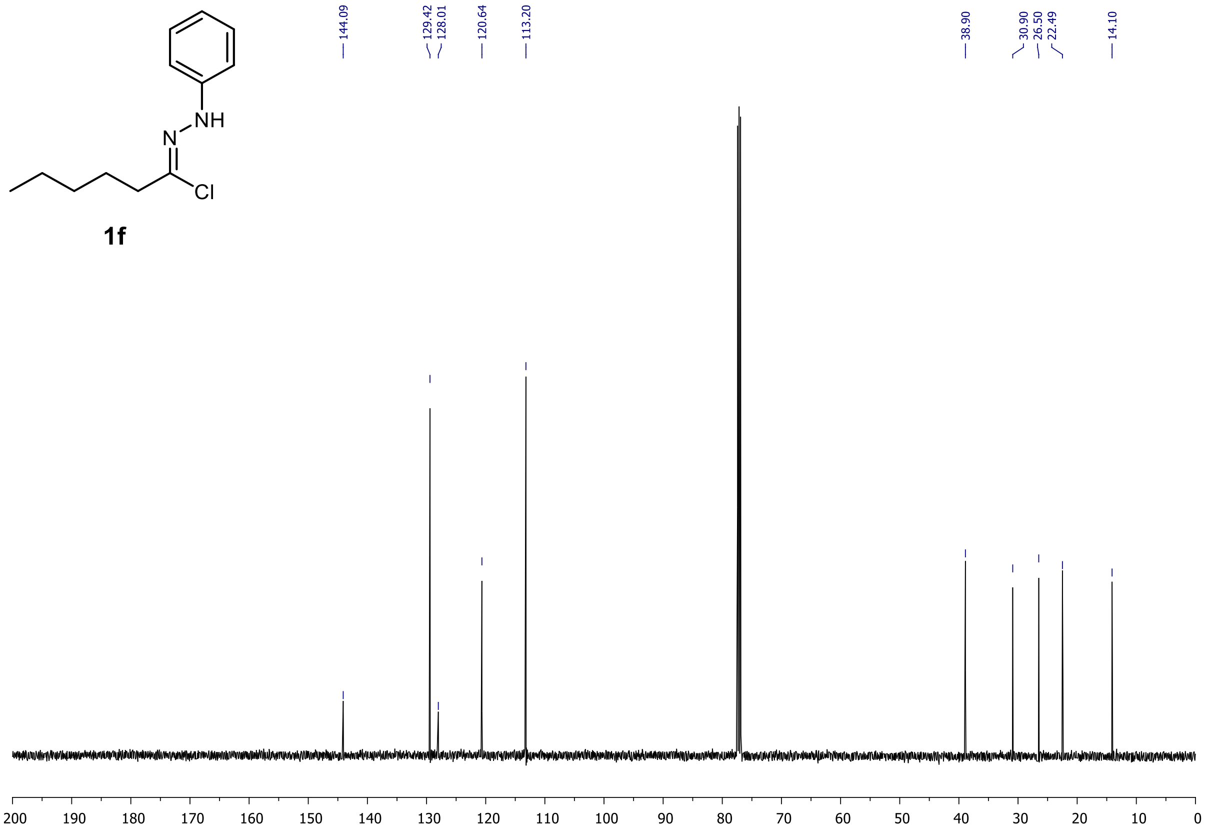

^{1}H NMR (400 MHz, $CDCl_3$)

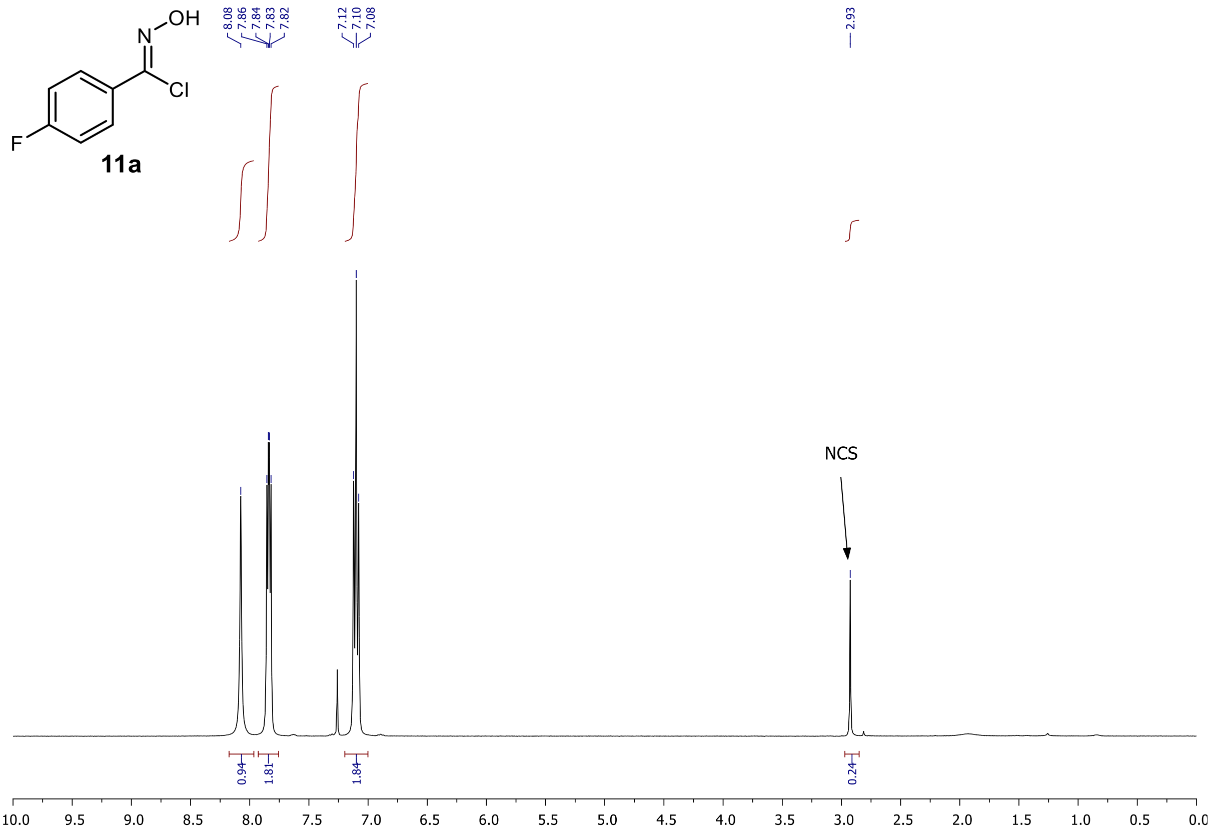

^{19}F NMR (471 MHz, $CDCl_3$)

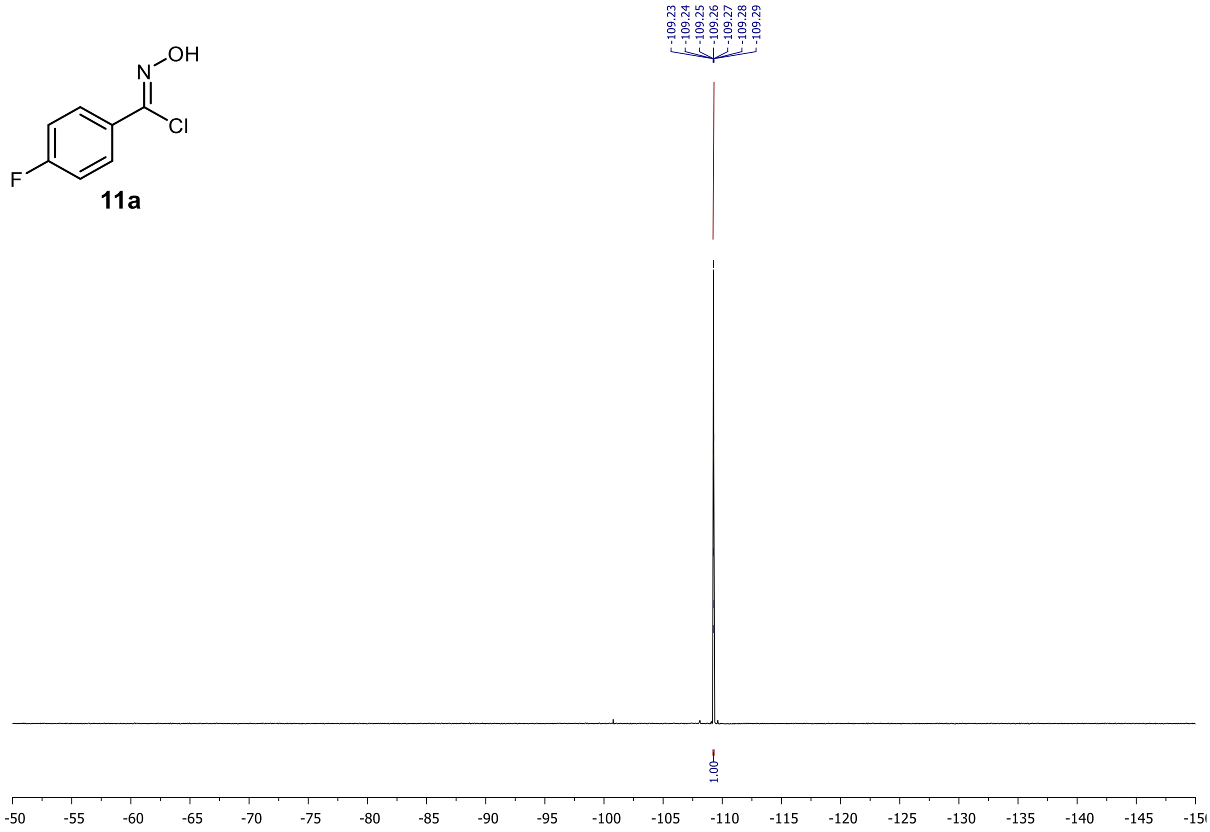

^{13}C NMR (126 MHz, $CDCl_3$)

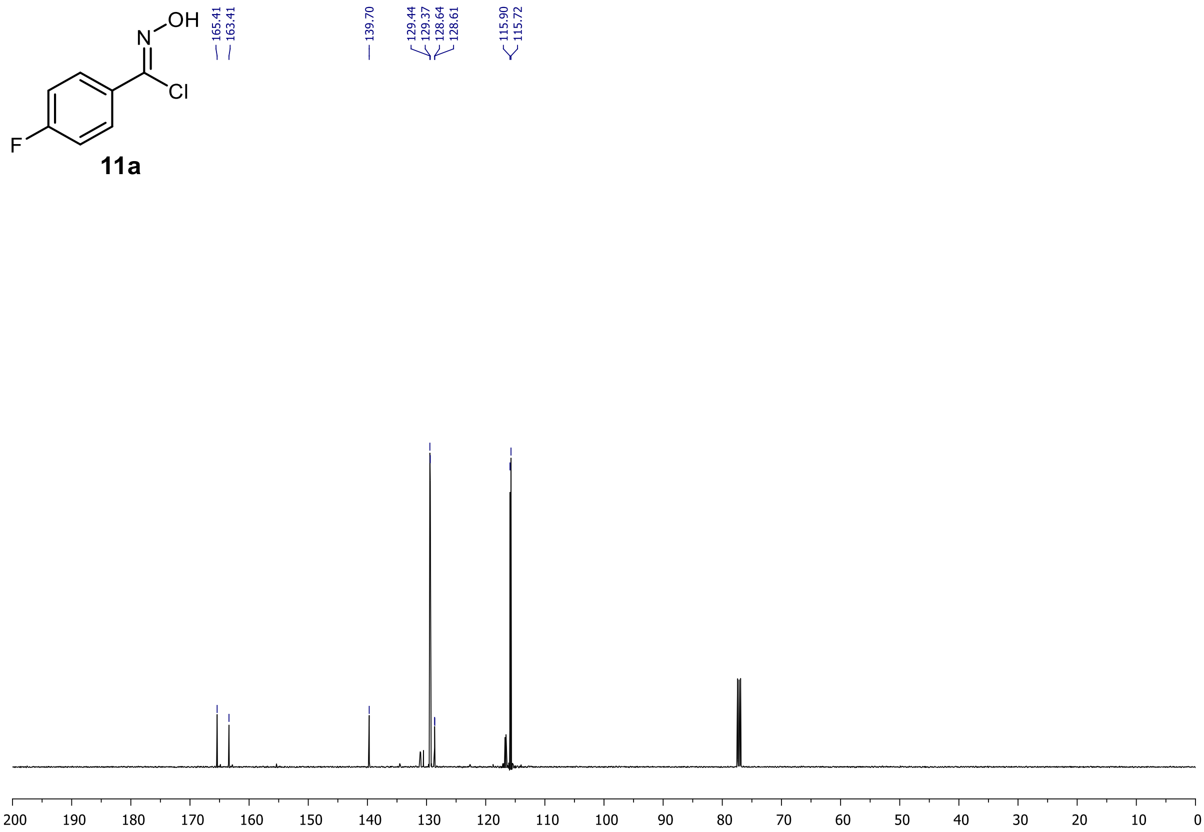

^{1}H NMR (500 MHz, $CDCl_3$)

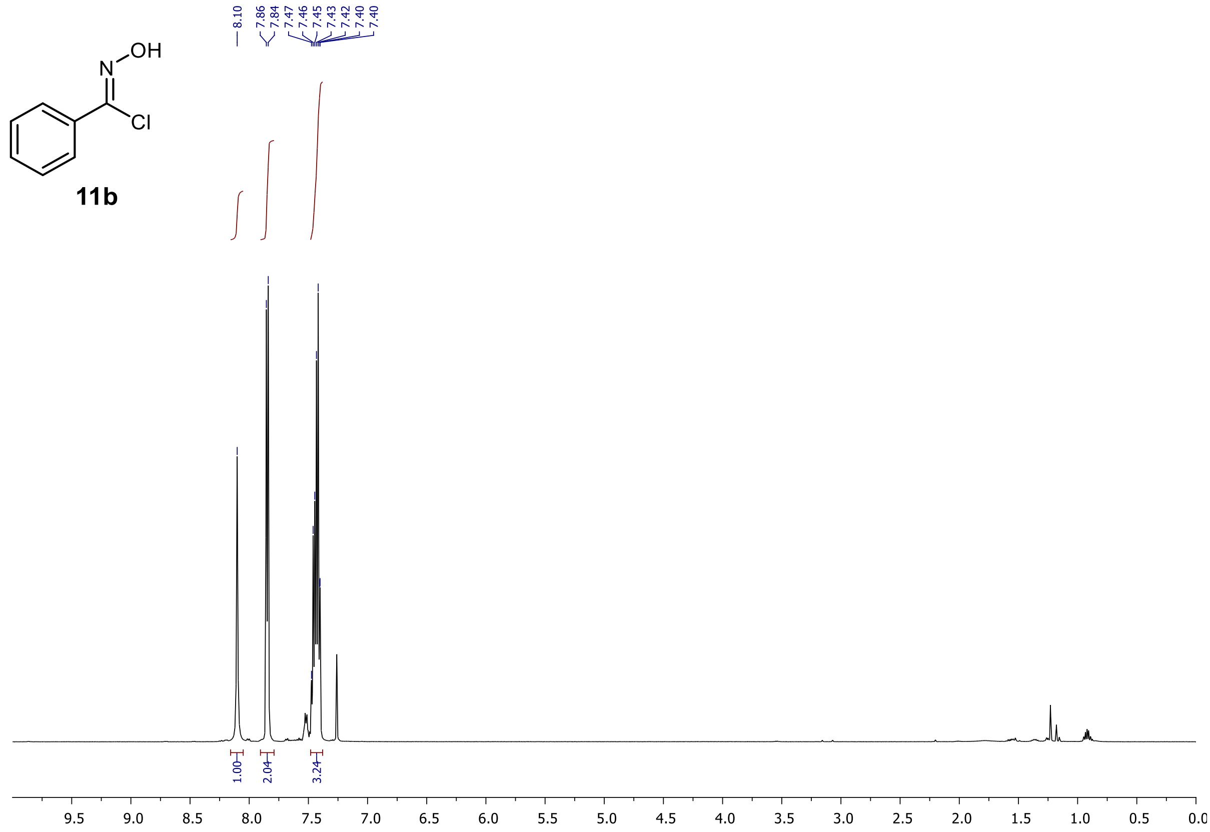

^{13}C NMR (126 MHz, $CDCl_3$)

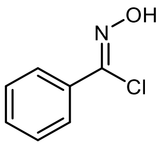

11b

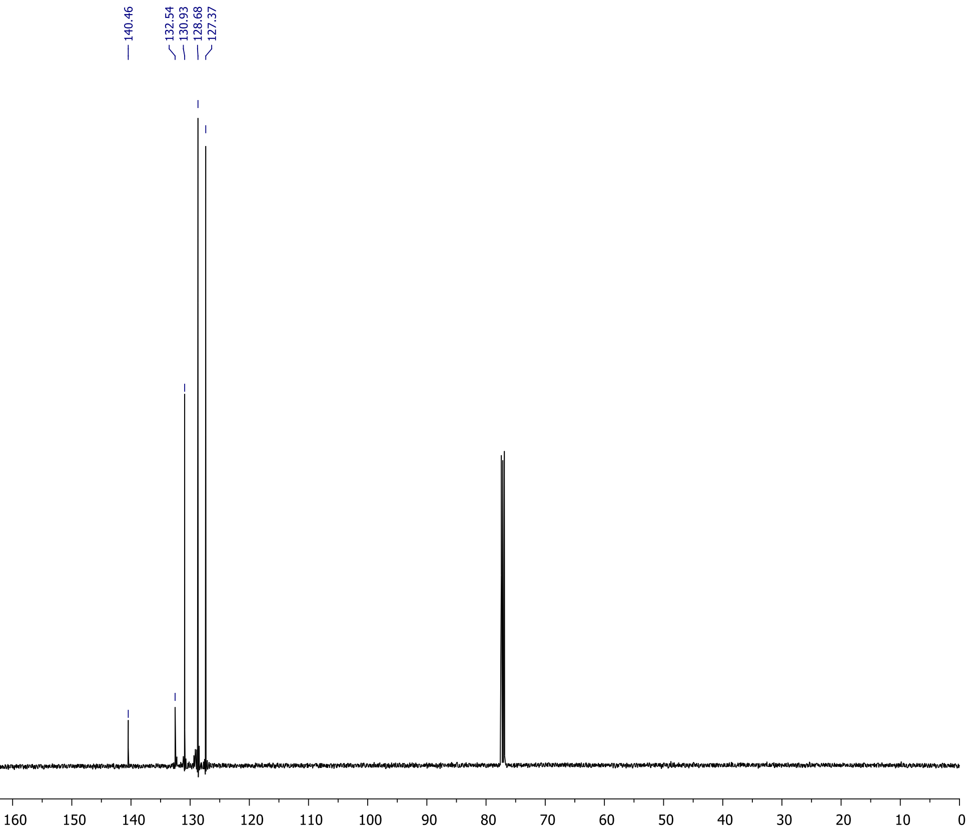

^{1}H NMR (500 MHz, $CDCl_3$)

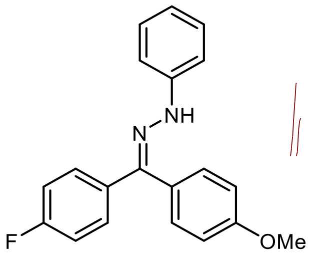

3a

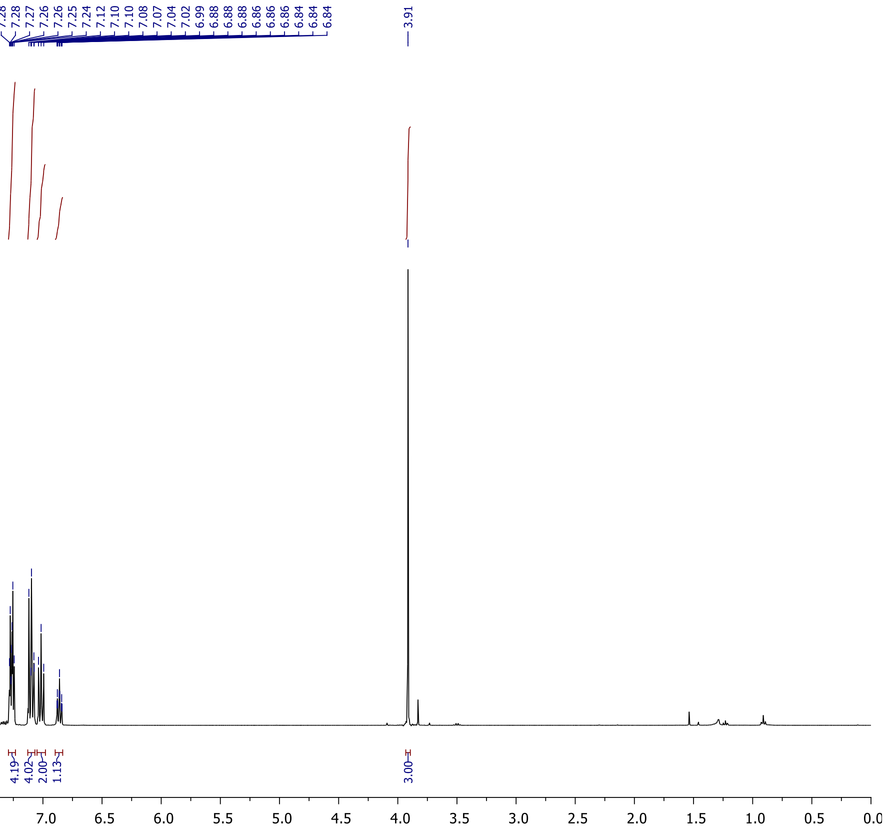

^{19}F NMR (471 MHz, $CDCl_3$)

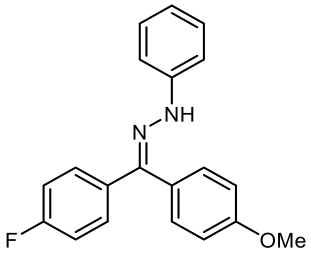

3a

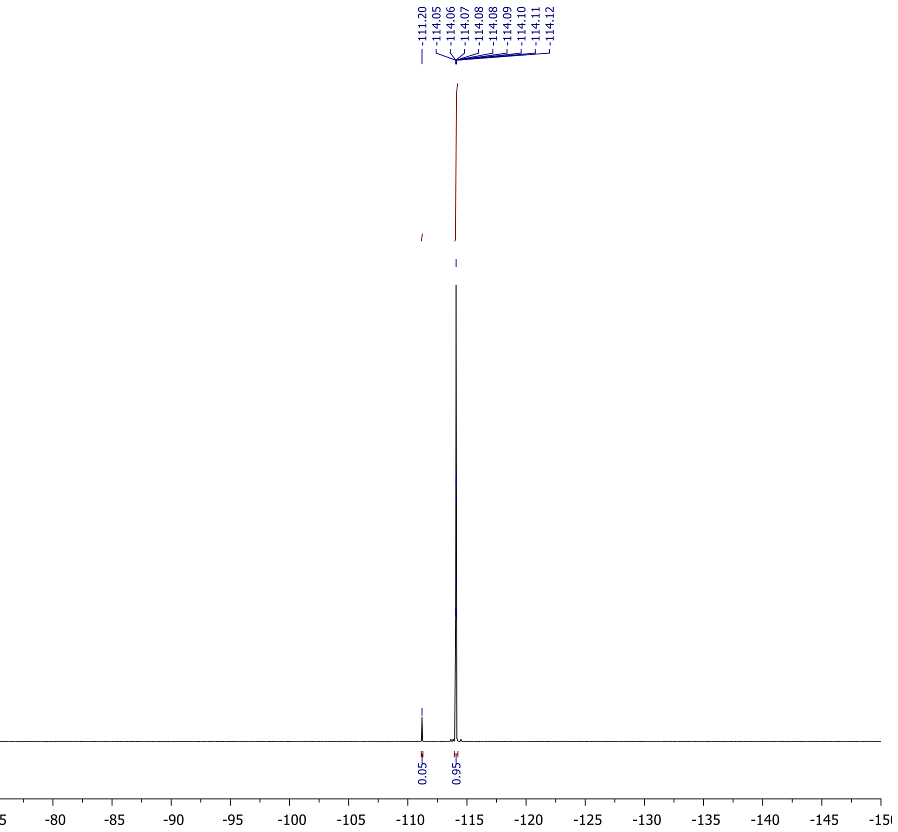

^{13}C NMR (126 MHz, $CDCl_3$)

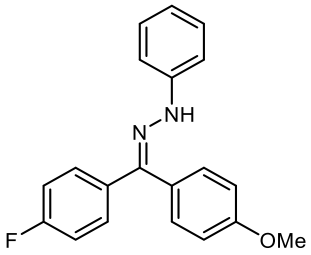

3a

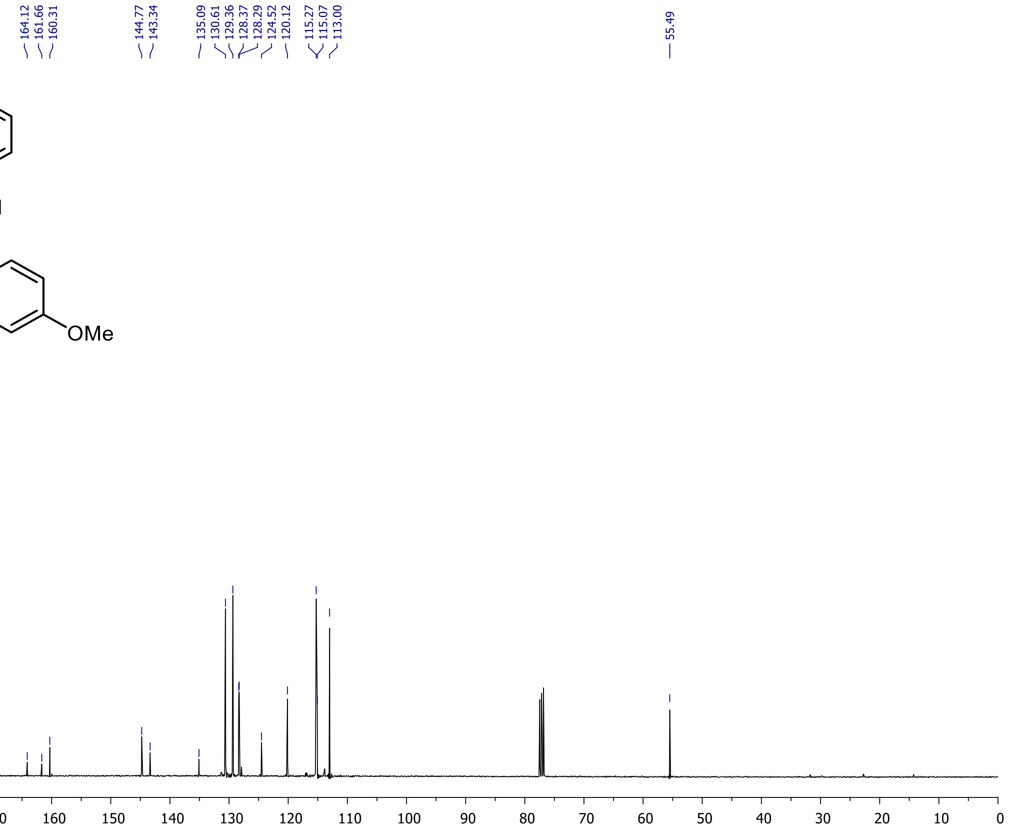

^{1}H NMR (500 MHz, $CDCl_3$)

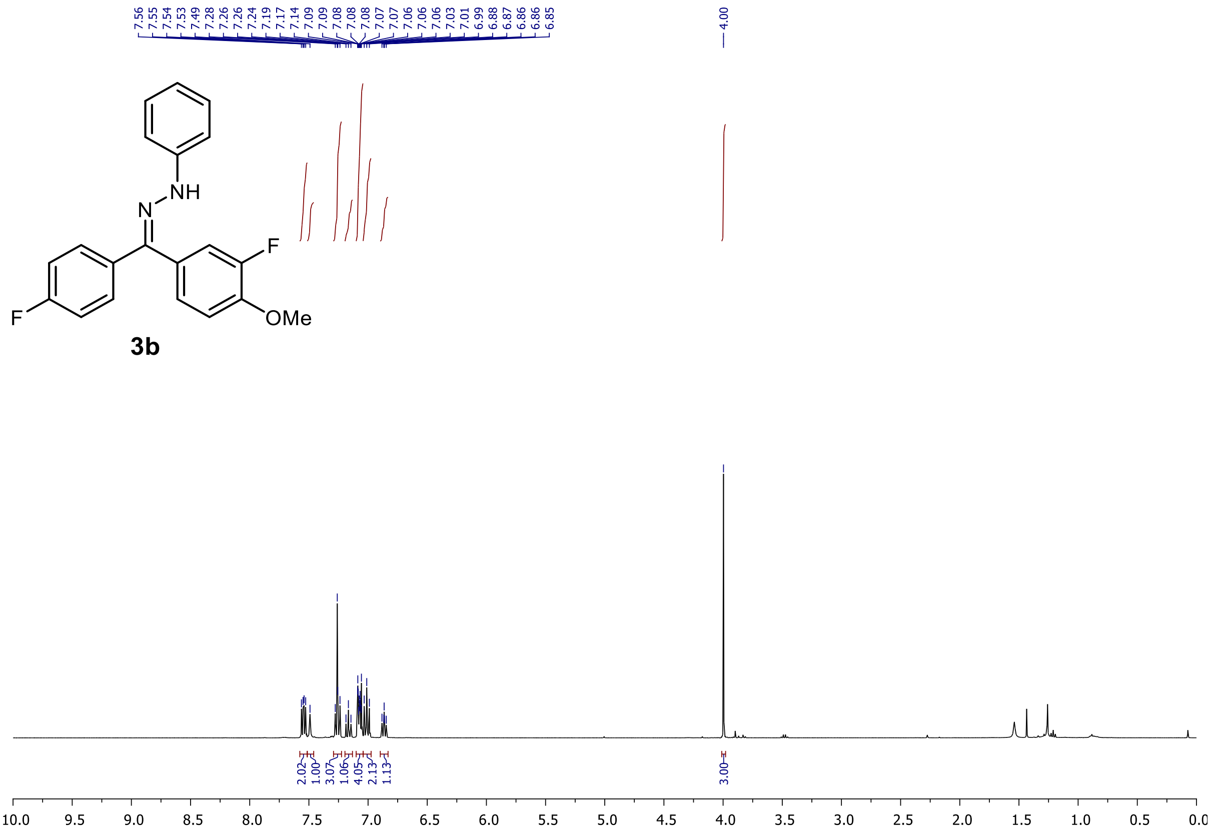

^{19}F NMR (471 MHz, $CDCl_3$)

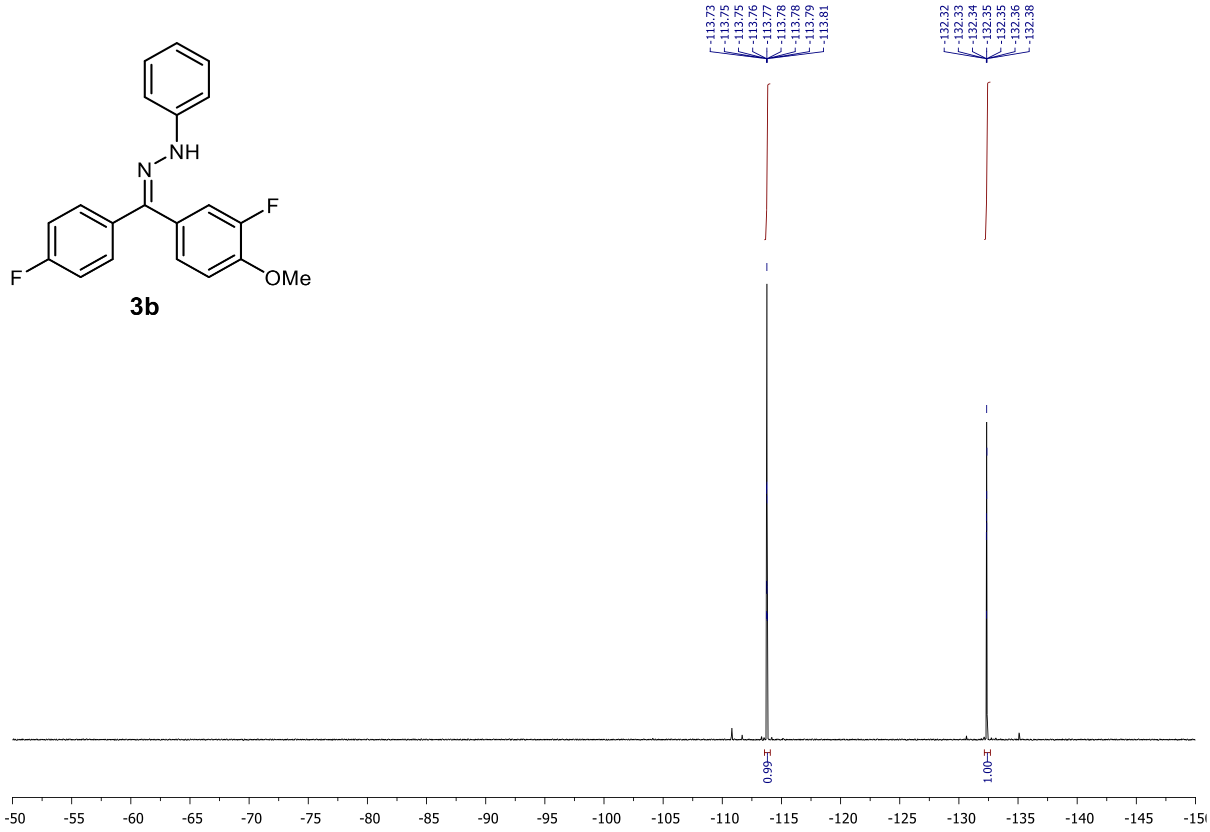

^{13}C NMR (126 MHz, $CDCl_3$)

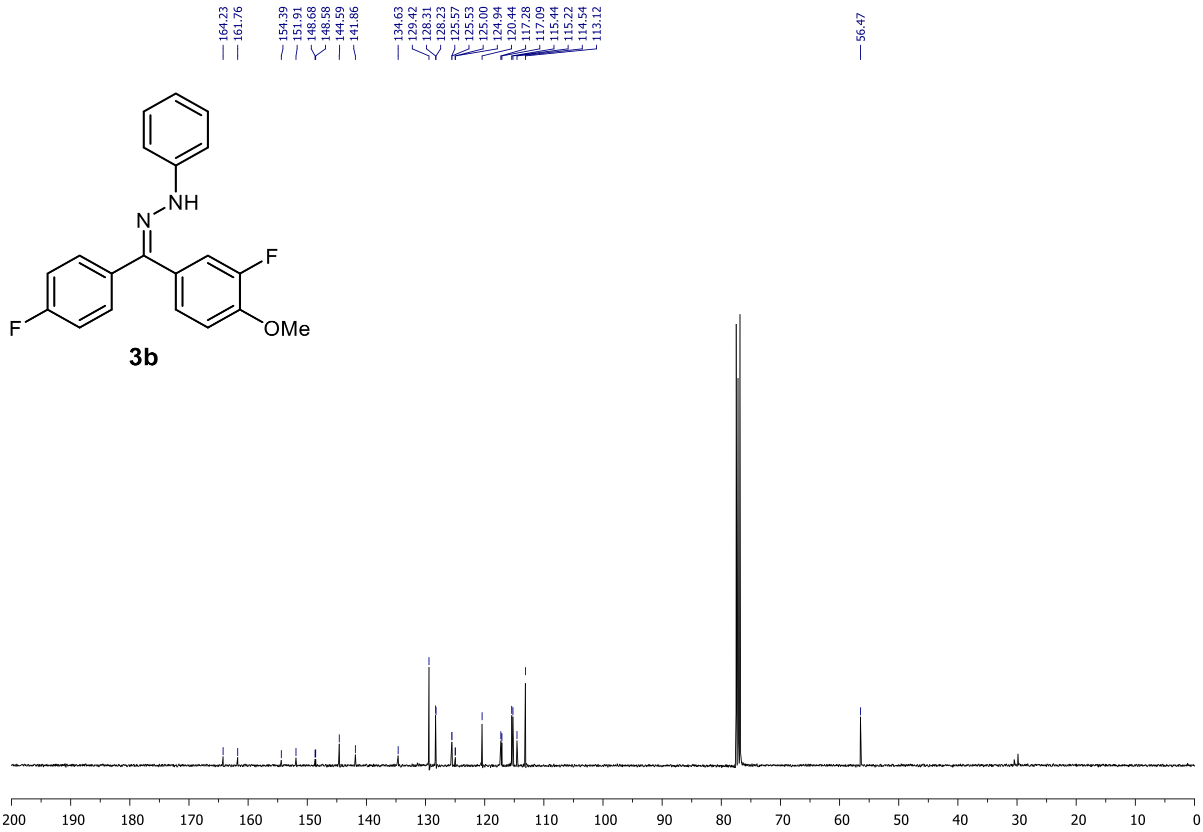

^{1}H NMR (400 MHz, $CDCl_3$)

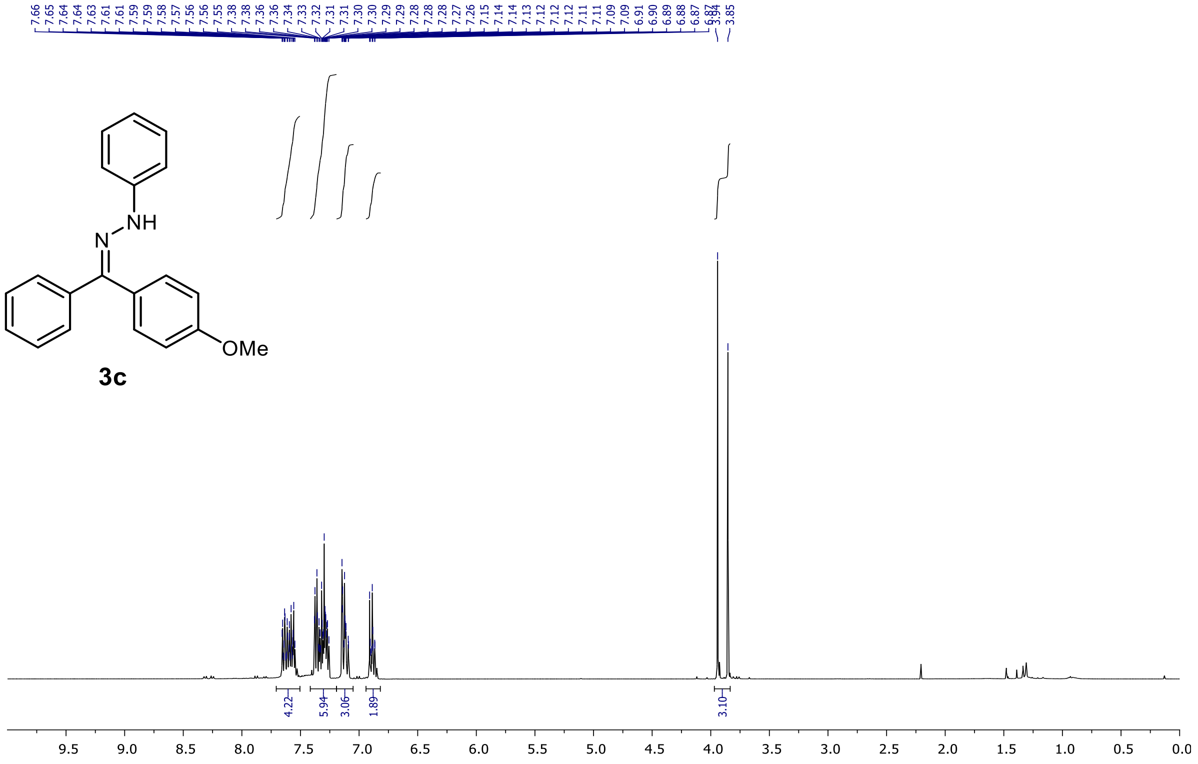

^{13}C NMR (101 MHz, $CDCl_3$)

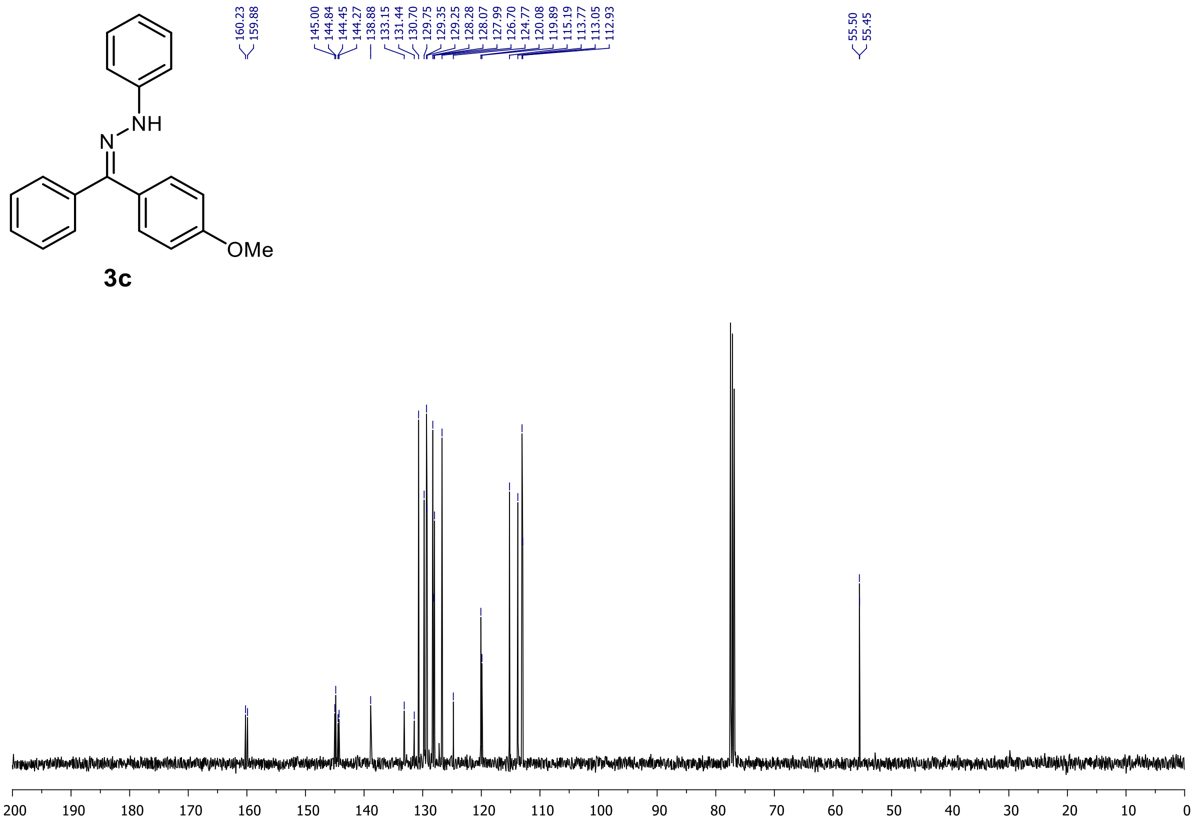

^{1}H NMR (500 MHz, $CDCl_3$)

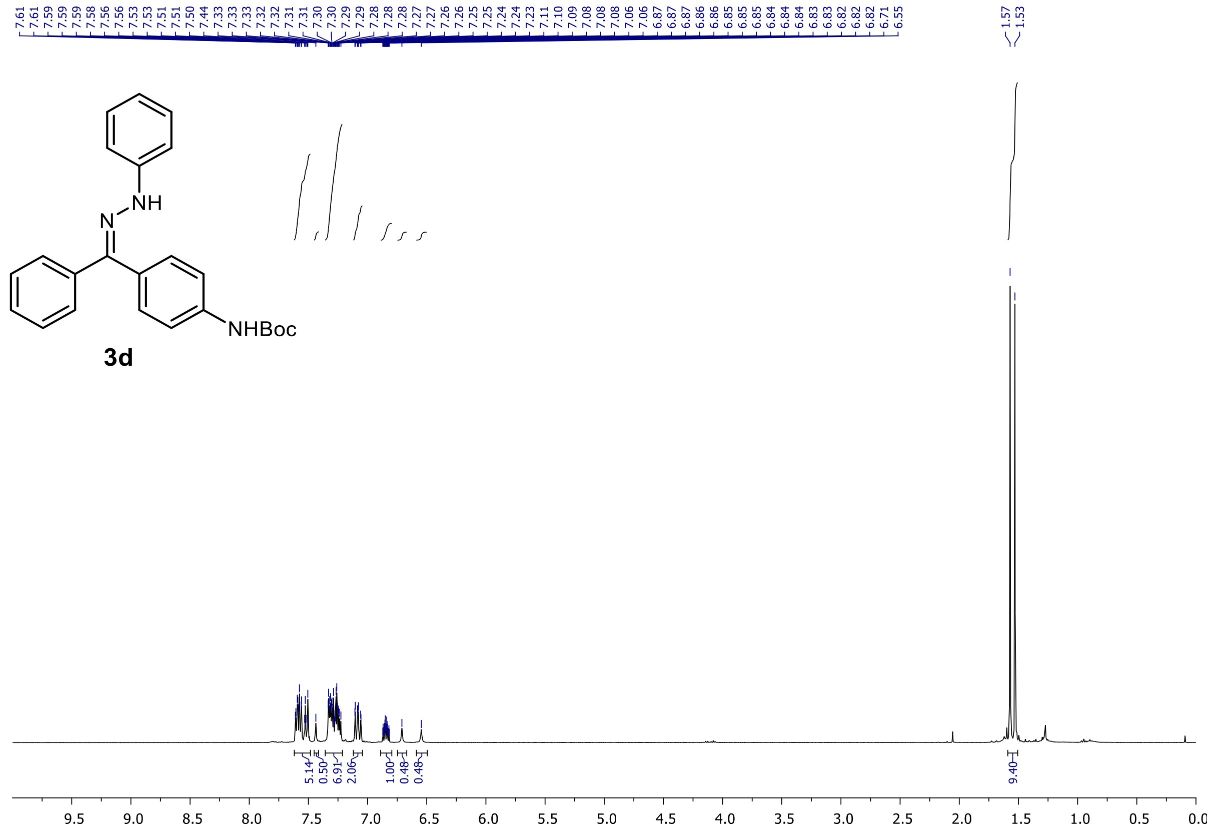

^{13}C NMR (101 MHz, $CDCl_3$)

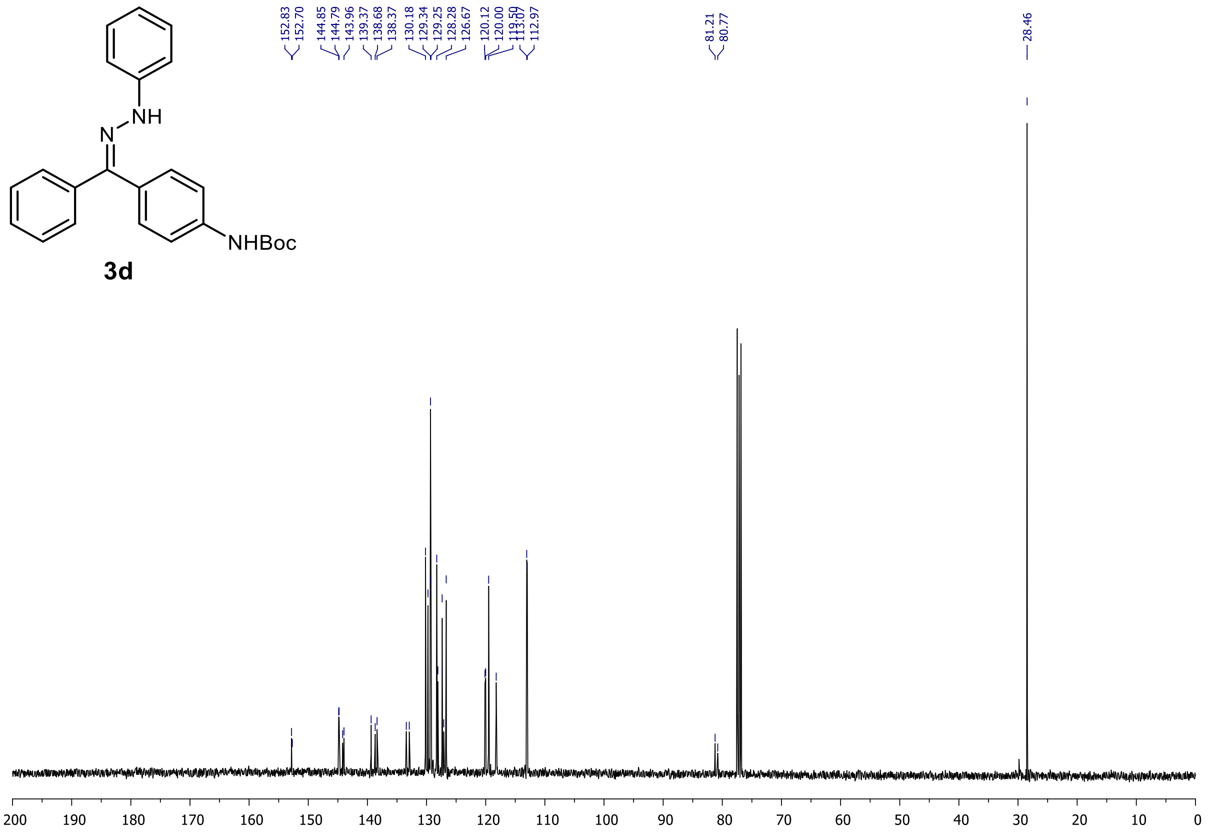

3d

^{1}H NMR (500 MHz, $CDCl_3$)

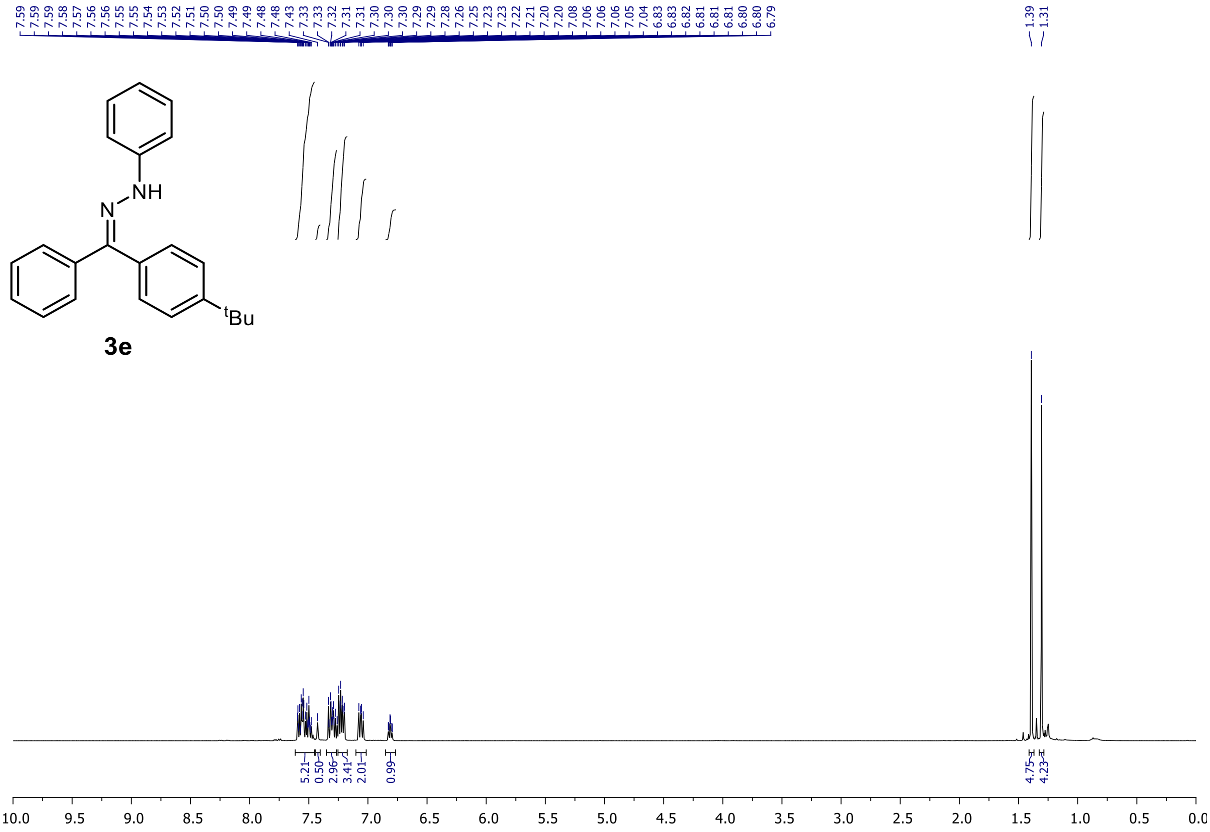

3e

^{13}C NMR (126 MHz, CDCl$_3$)

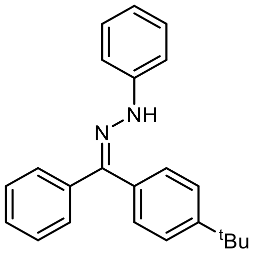

3e

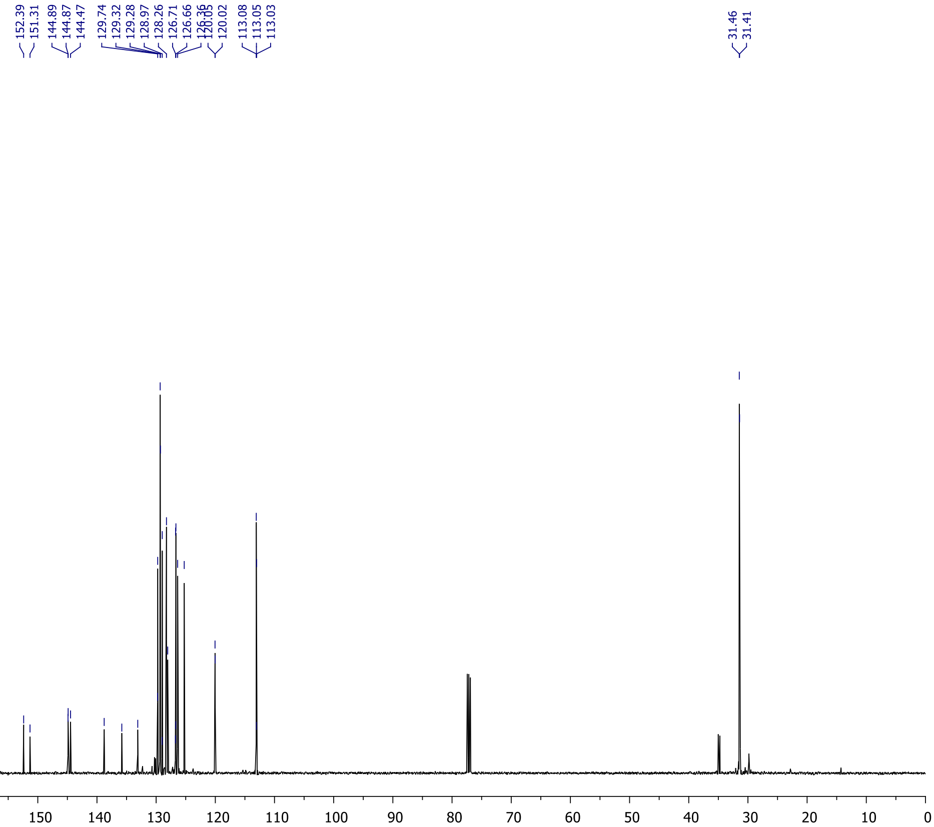

^{1}H NMR (400 MHz, CDCl$_3$)

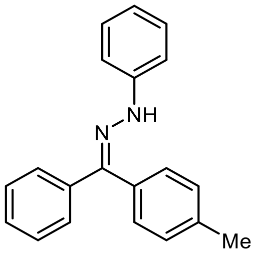

3f

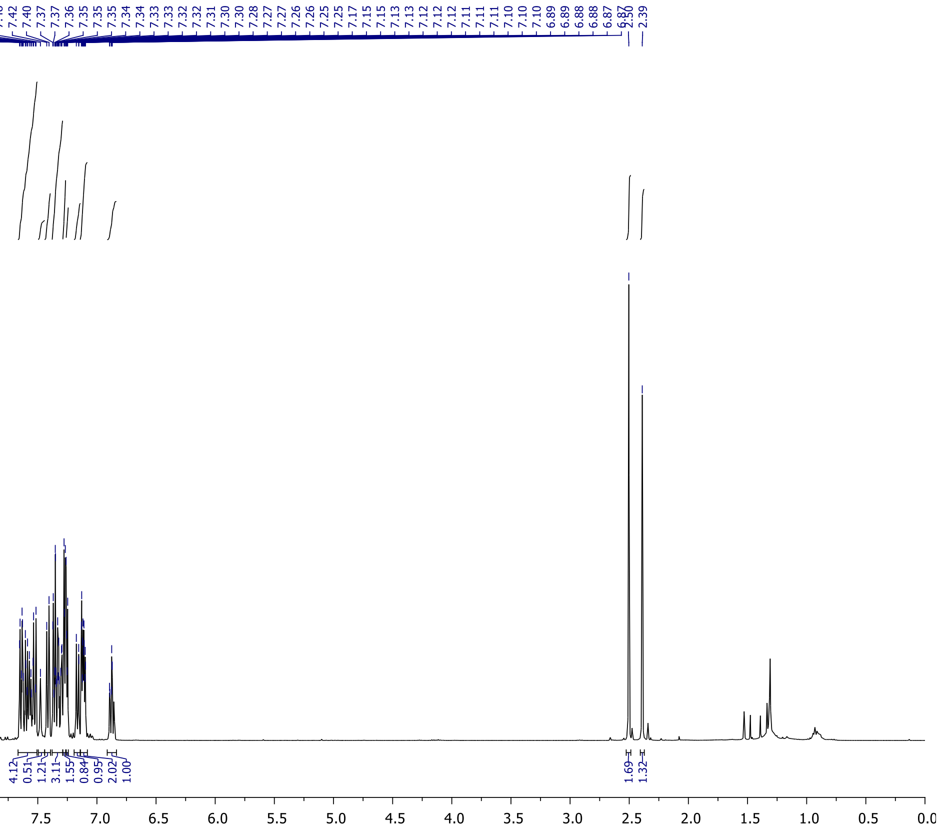

^{13}C NMR (101 MHz, $CDCl_3$)

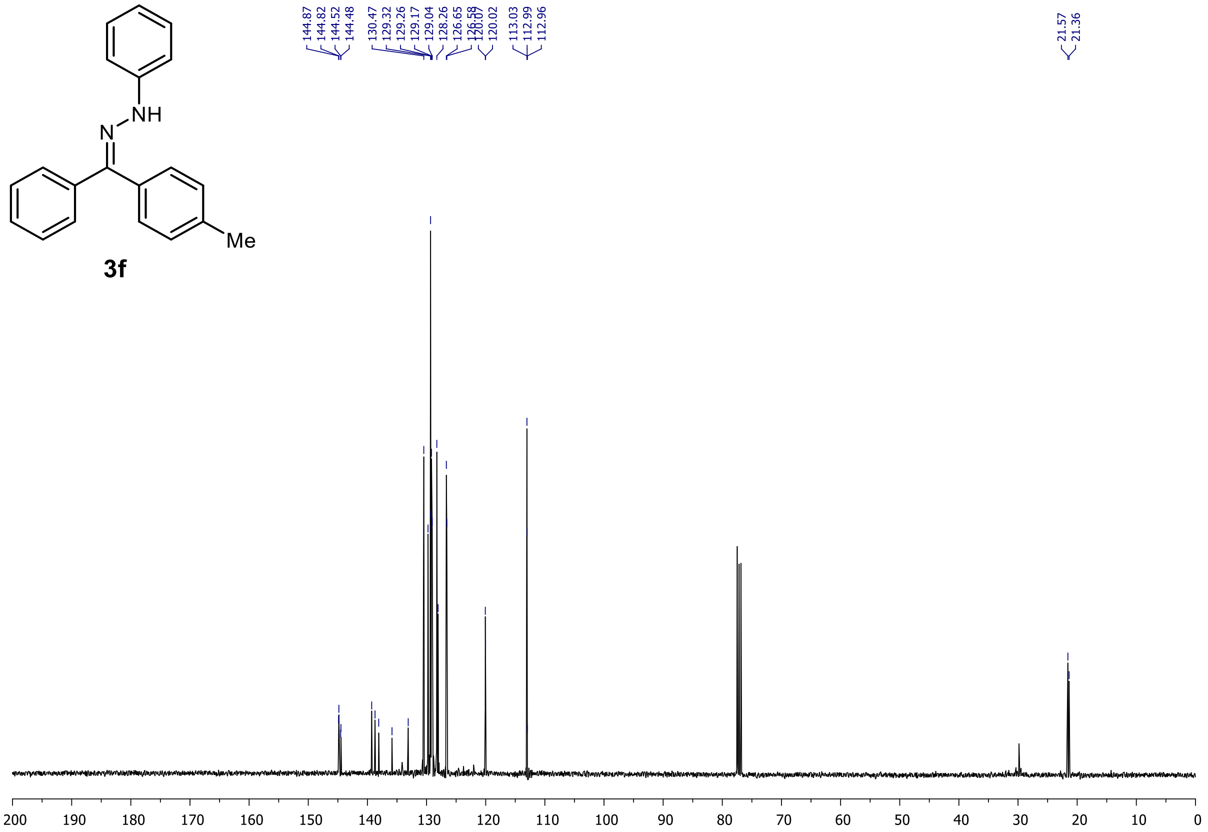

3f

^{1}H NMR (500 MHz, $CDCl_3$)

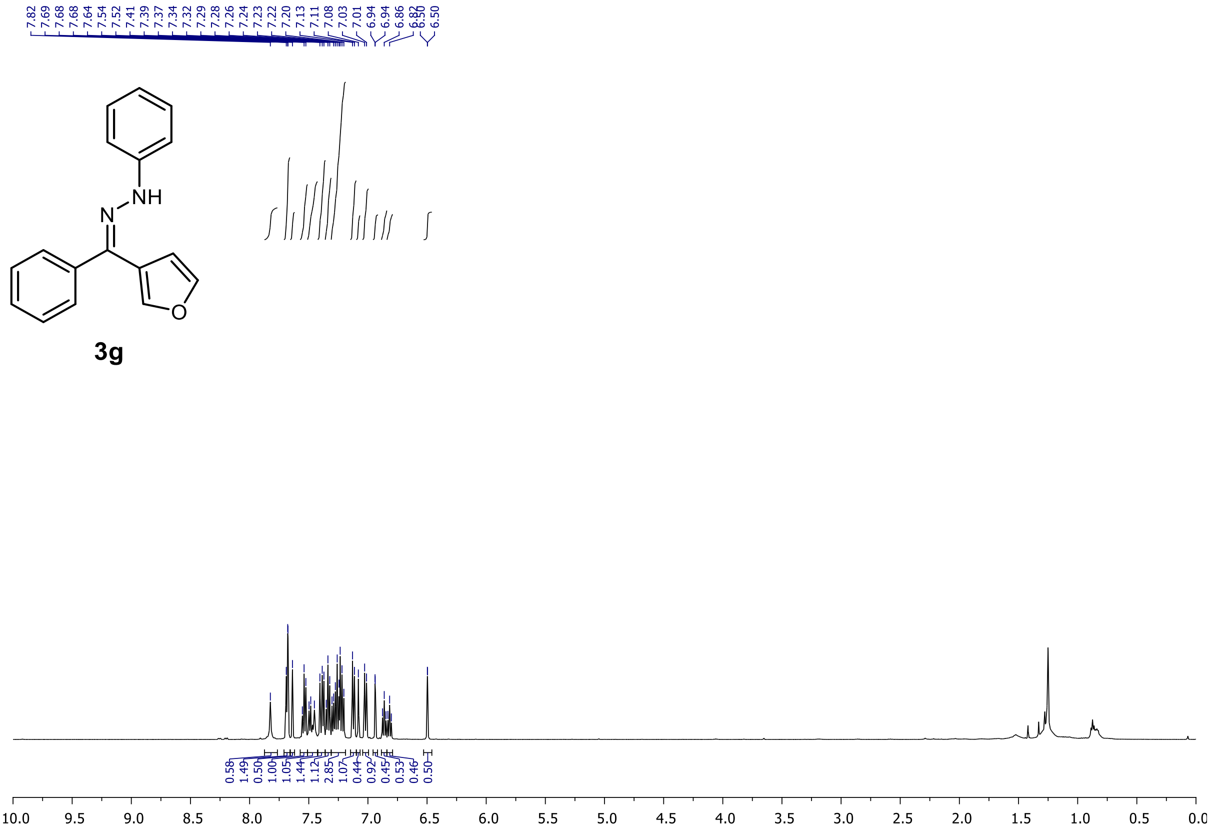

3g

^{13}C NMR (101 MHz, $CDCl_3$)

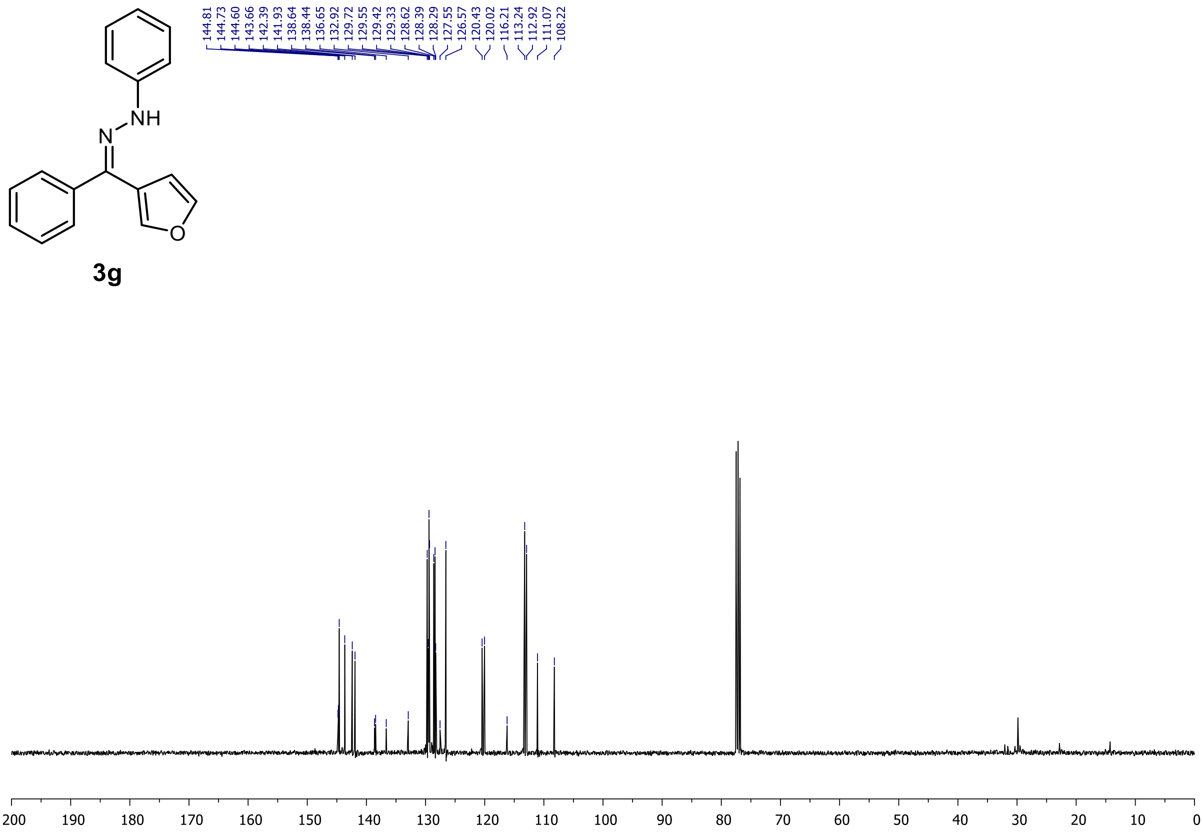

3g

^{1}H NMR (400 MHz, $CDCl_3$)

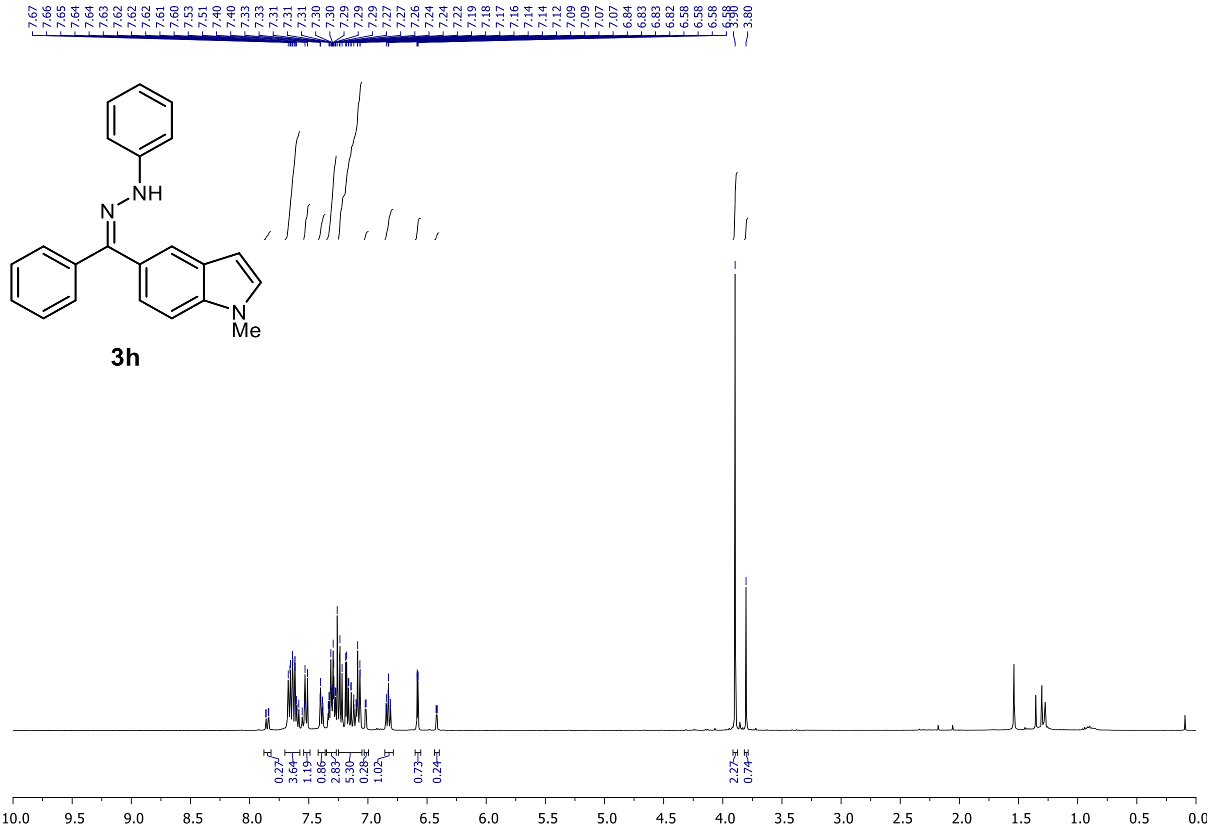

3h

^{13}C NMR (101 MHz, $CDCl_3$)

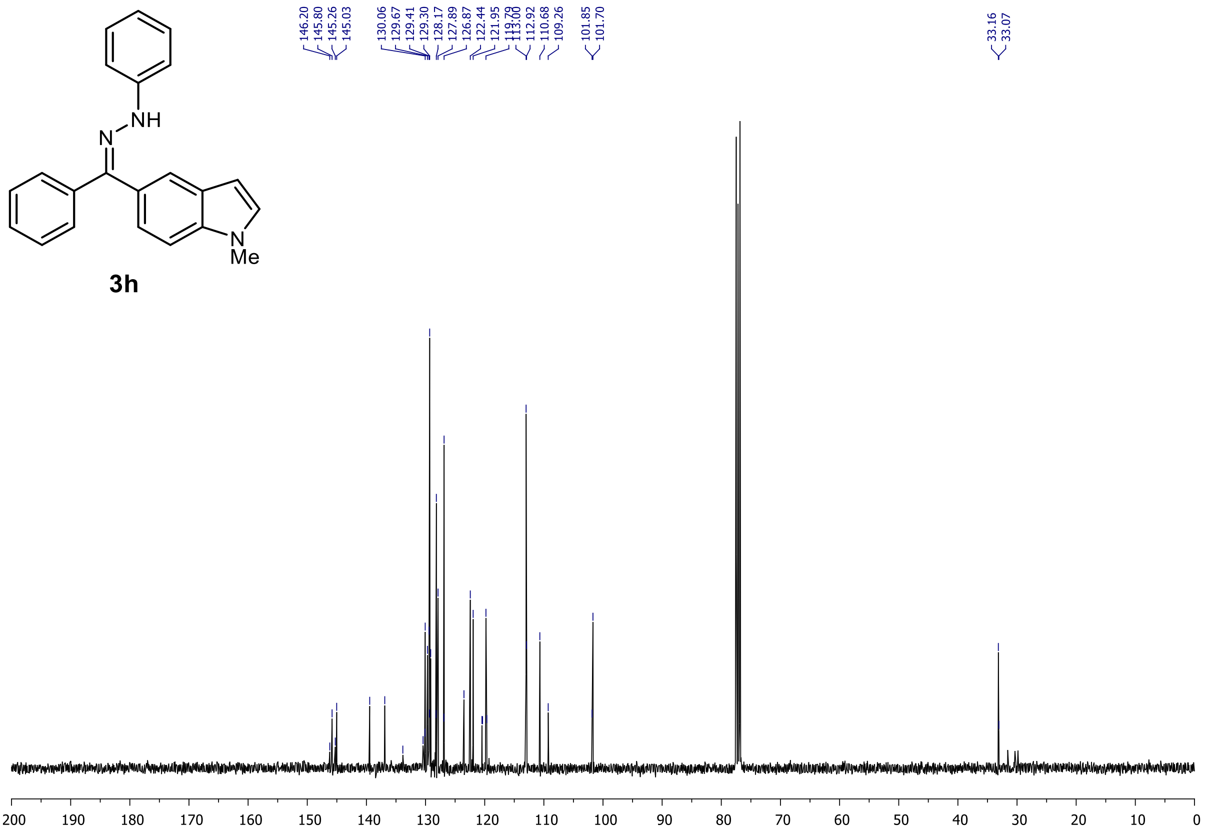

^{1}H NMR (400 MHz, $CDCl_3$)

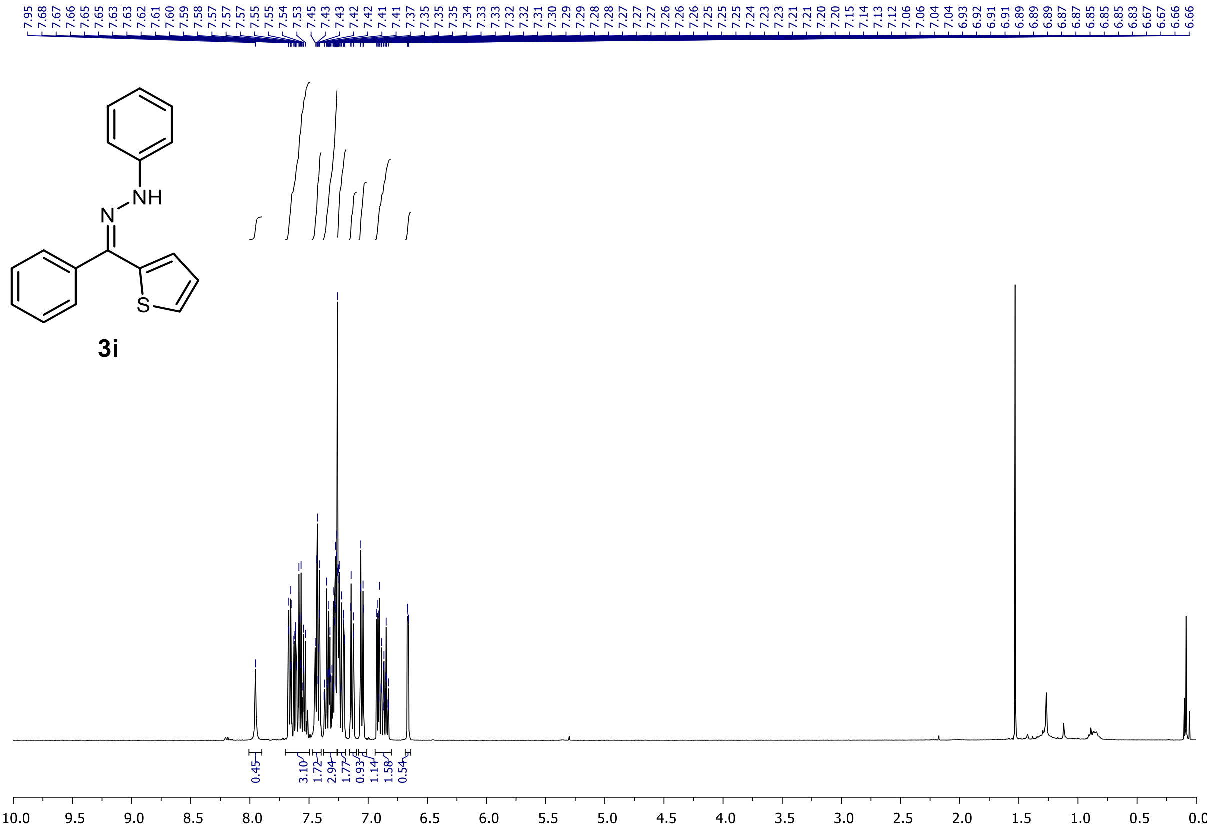

^{13}C NMR (101 MHz, CDCl$_3$)

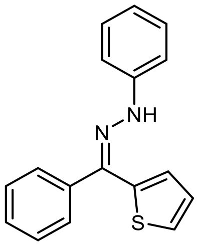

3i

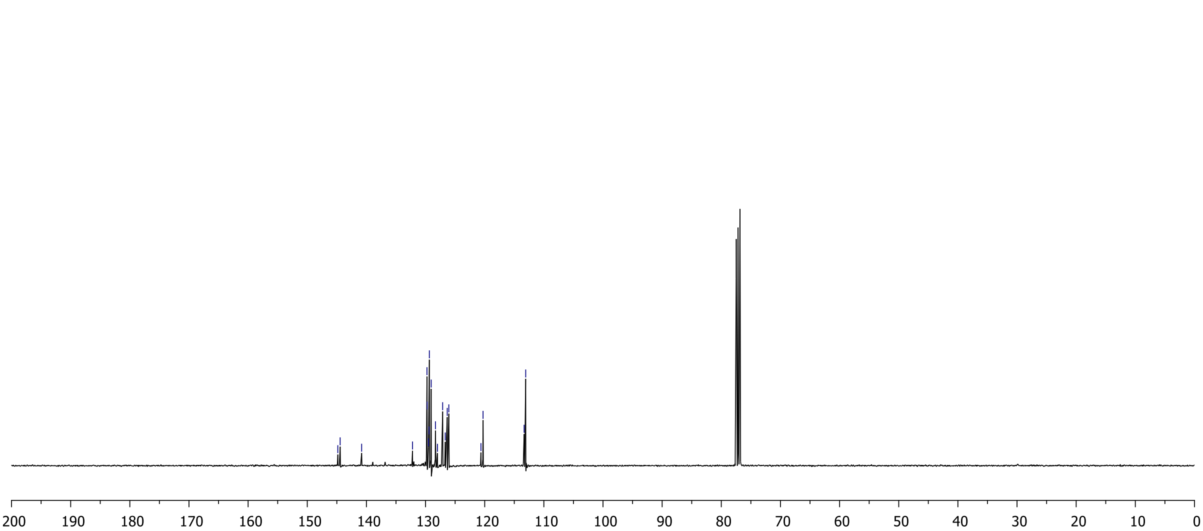

^{1}H NMR (500 MHz, CDCl$_3$)

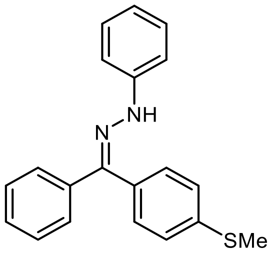

3j

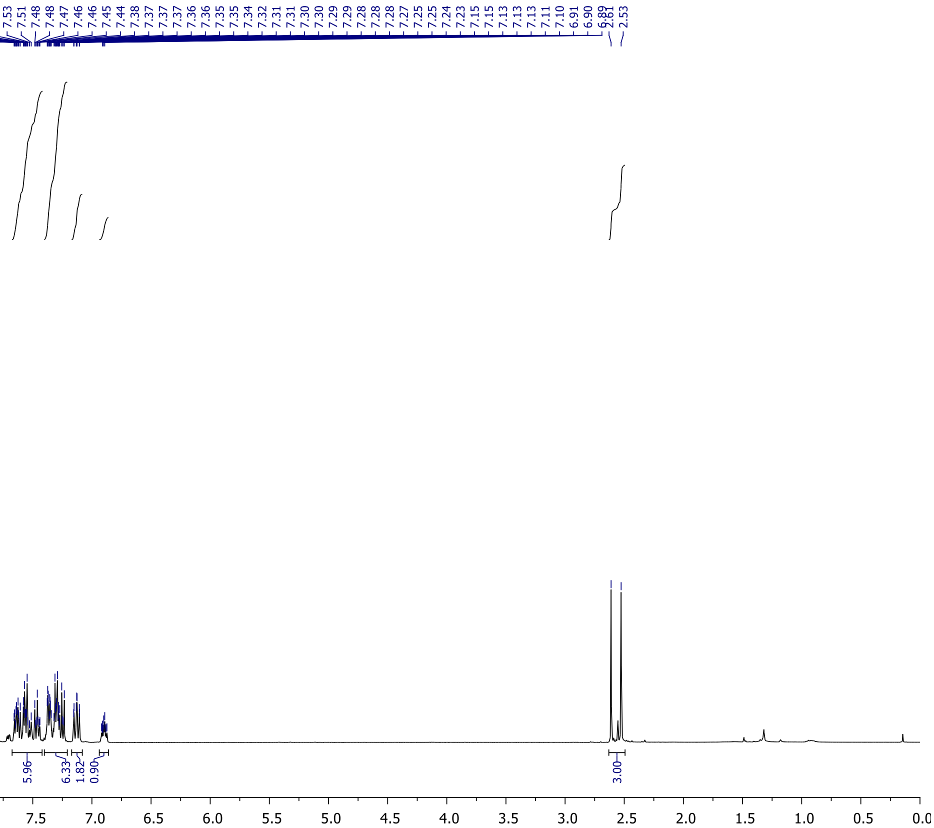

^{13}C NMR (101 MHz, $CDCl_3$)

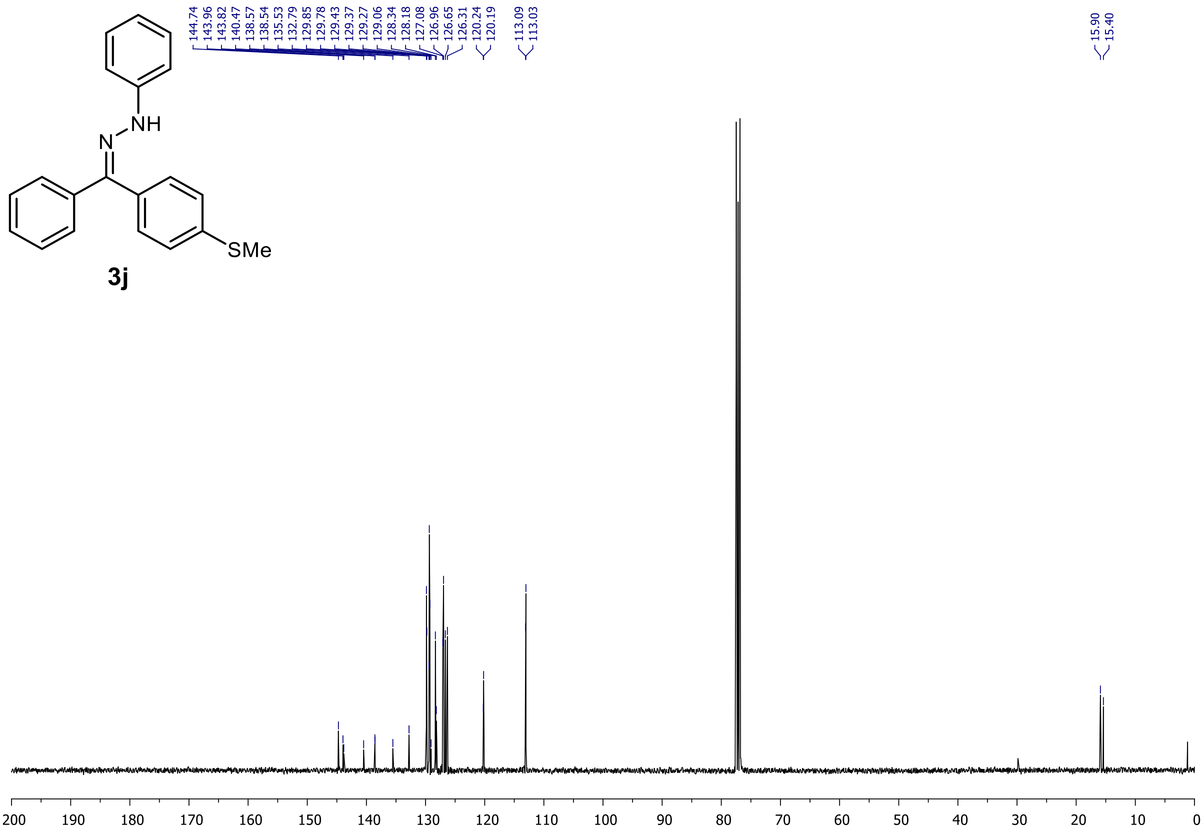

^{1}H NMR (400 MHz, $CDCl_3$)

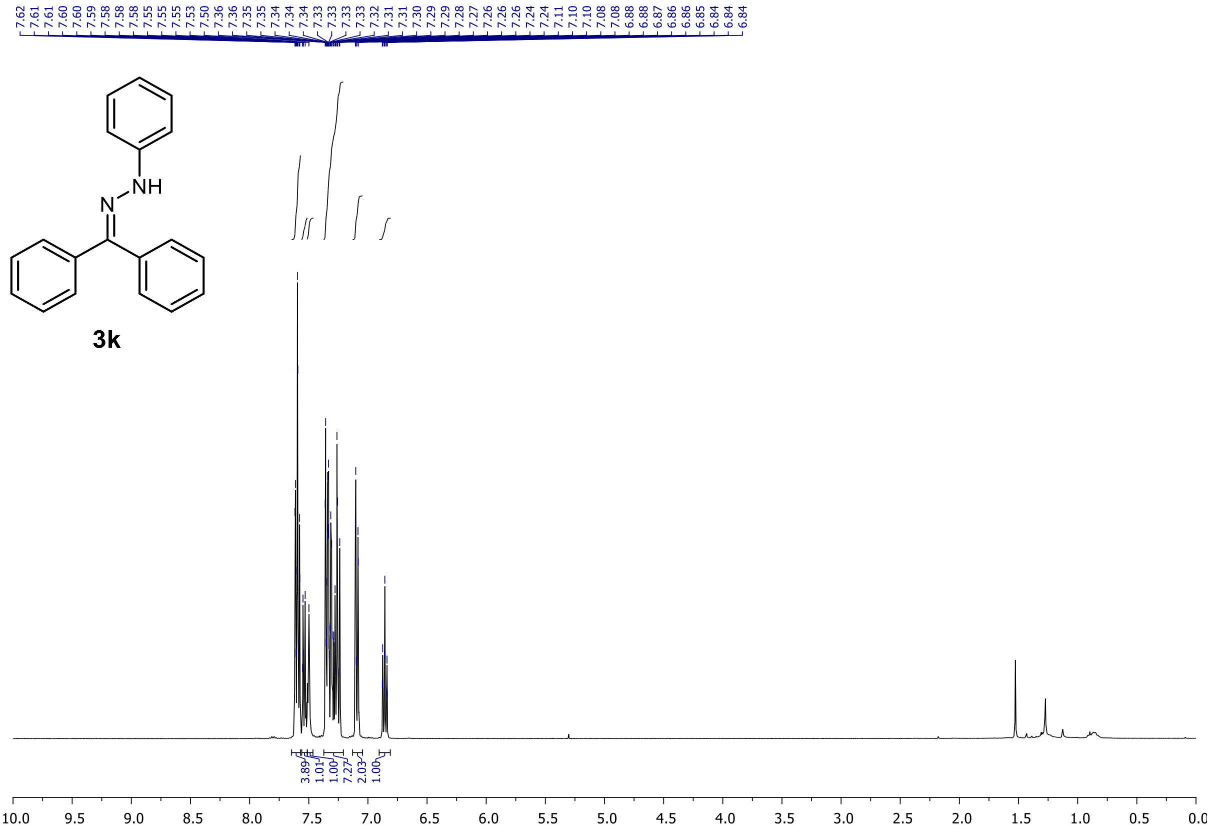

^{13}C NMR (101 MHz, $CDCl_3$)

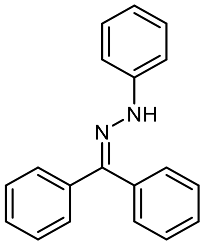

3k

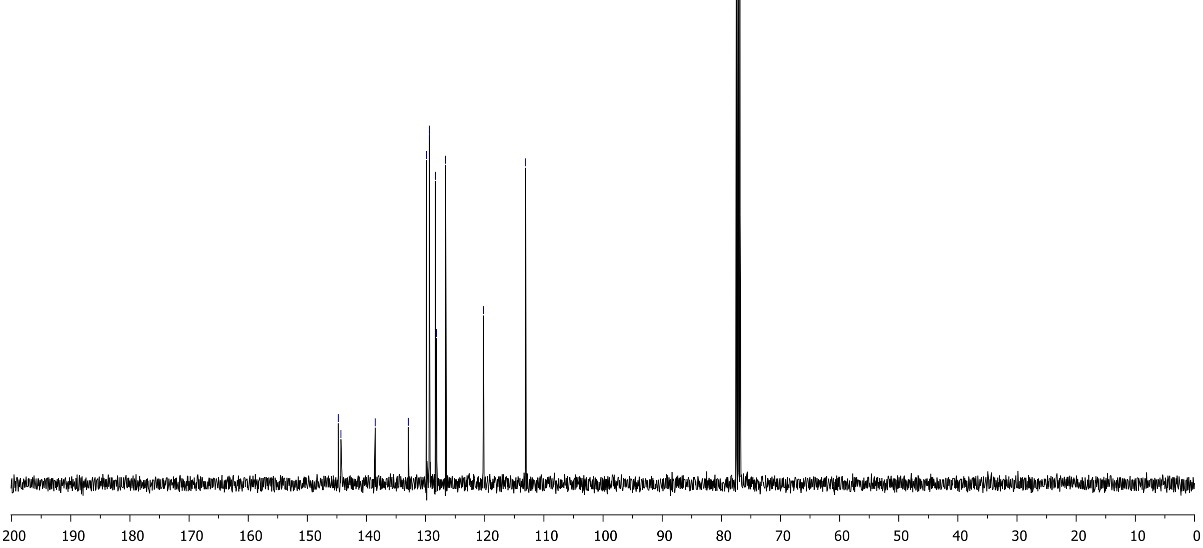

^{1}H NMR (400 MHz, $CDCl_3$)

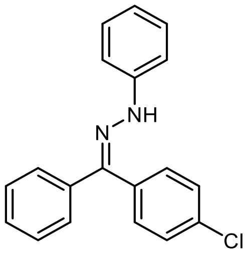

3l

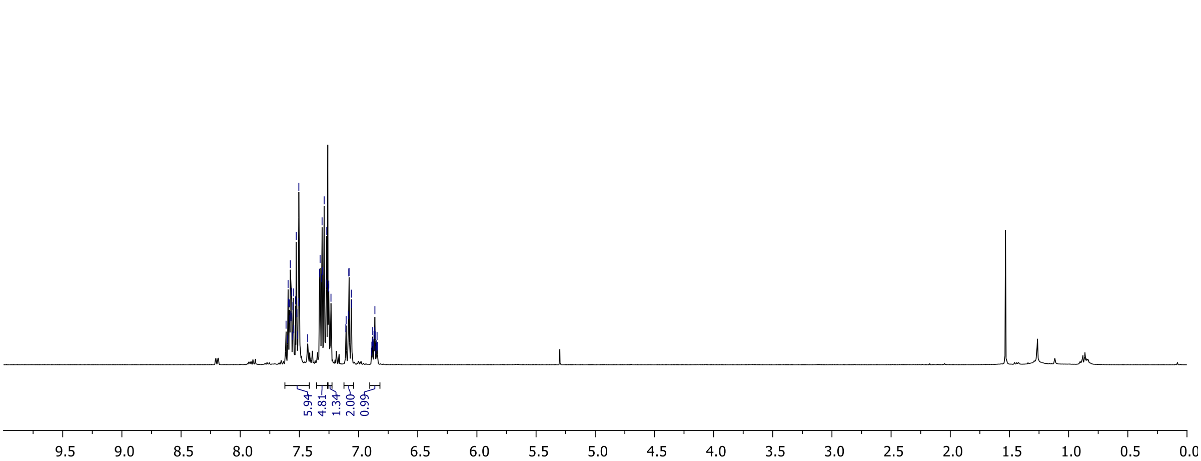

^{13}C NMR (101 MHz, $CDCl_3$)

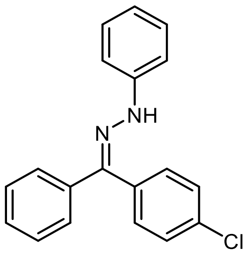

3l

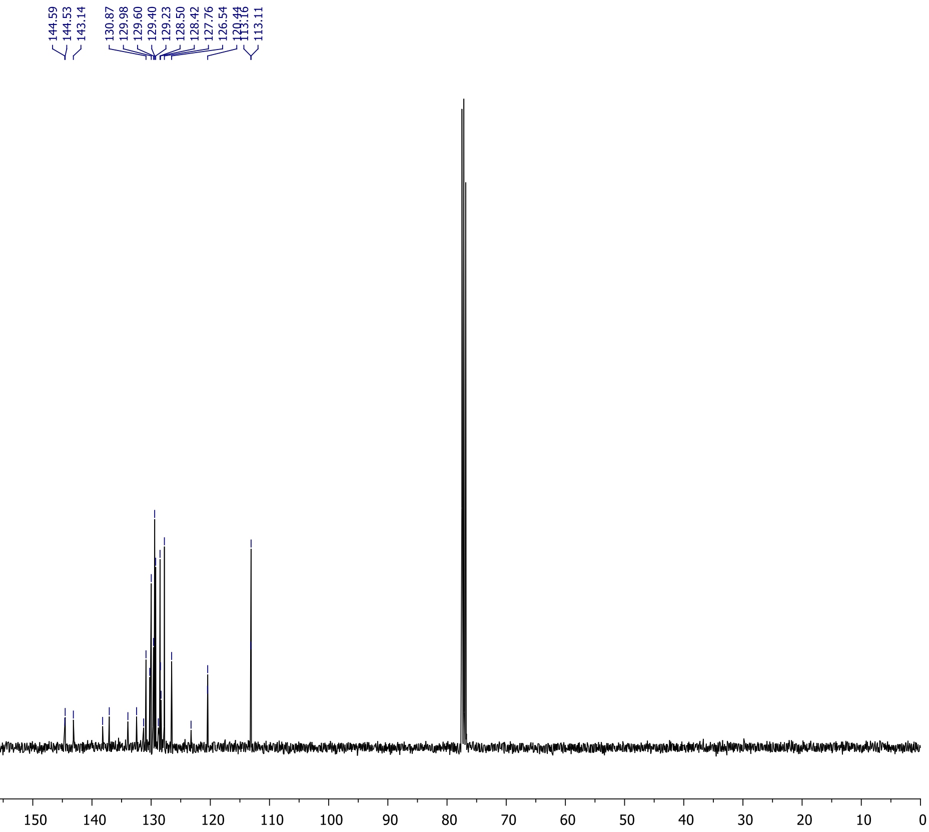

^{1}H NMR (500 MHz, $CDCl_3$)

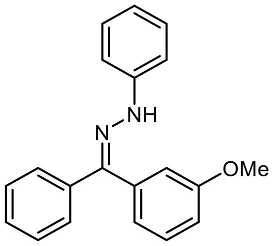

3m

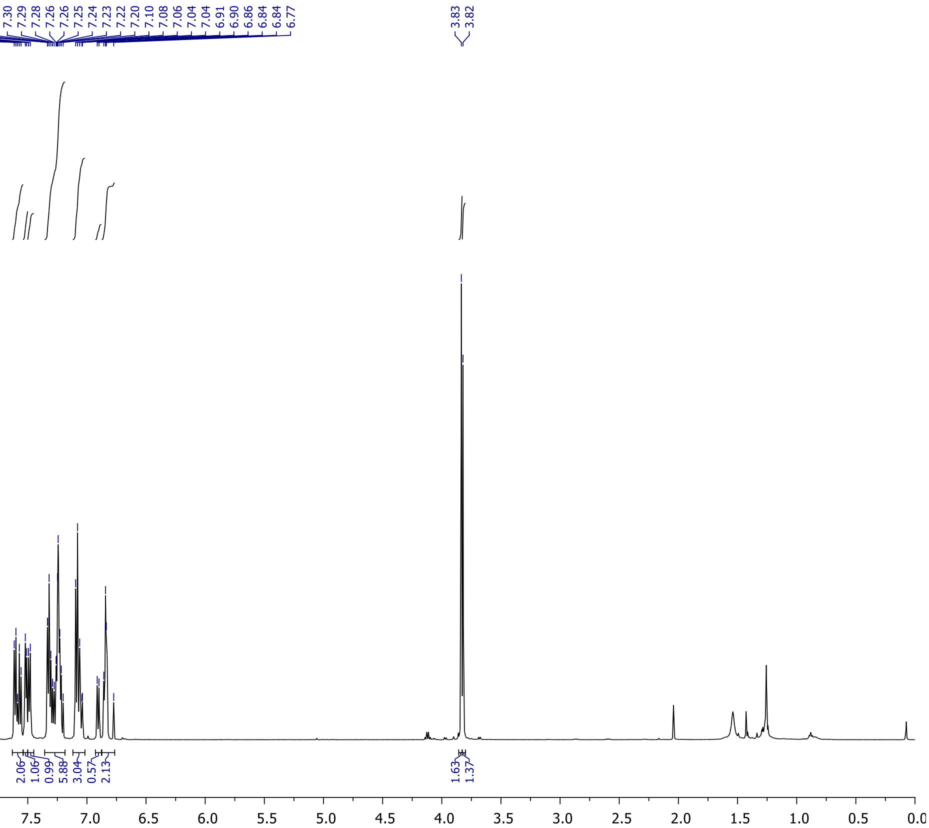

^{13}C NMR (126 MHz, $CDCl_3$)

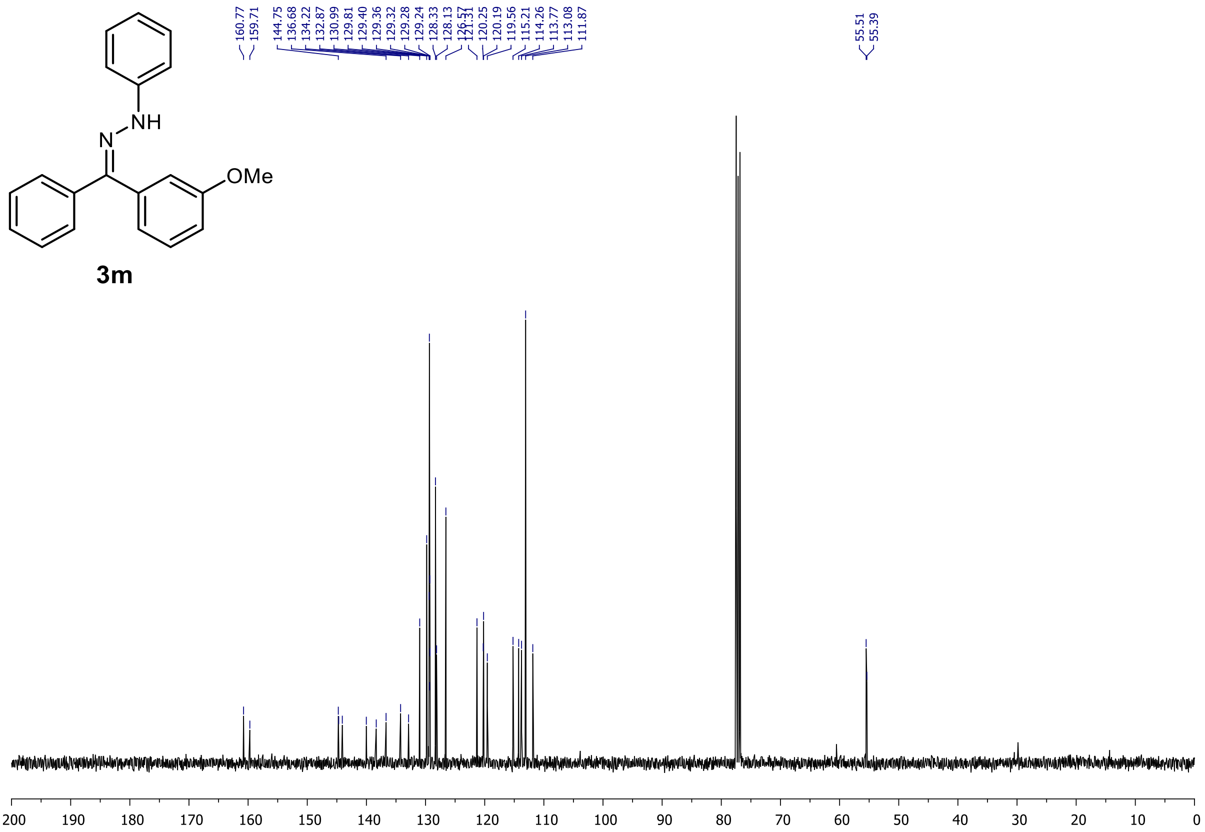

3m

^{1}H NMR (400 MHz, $CDCl_3$)

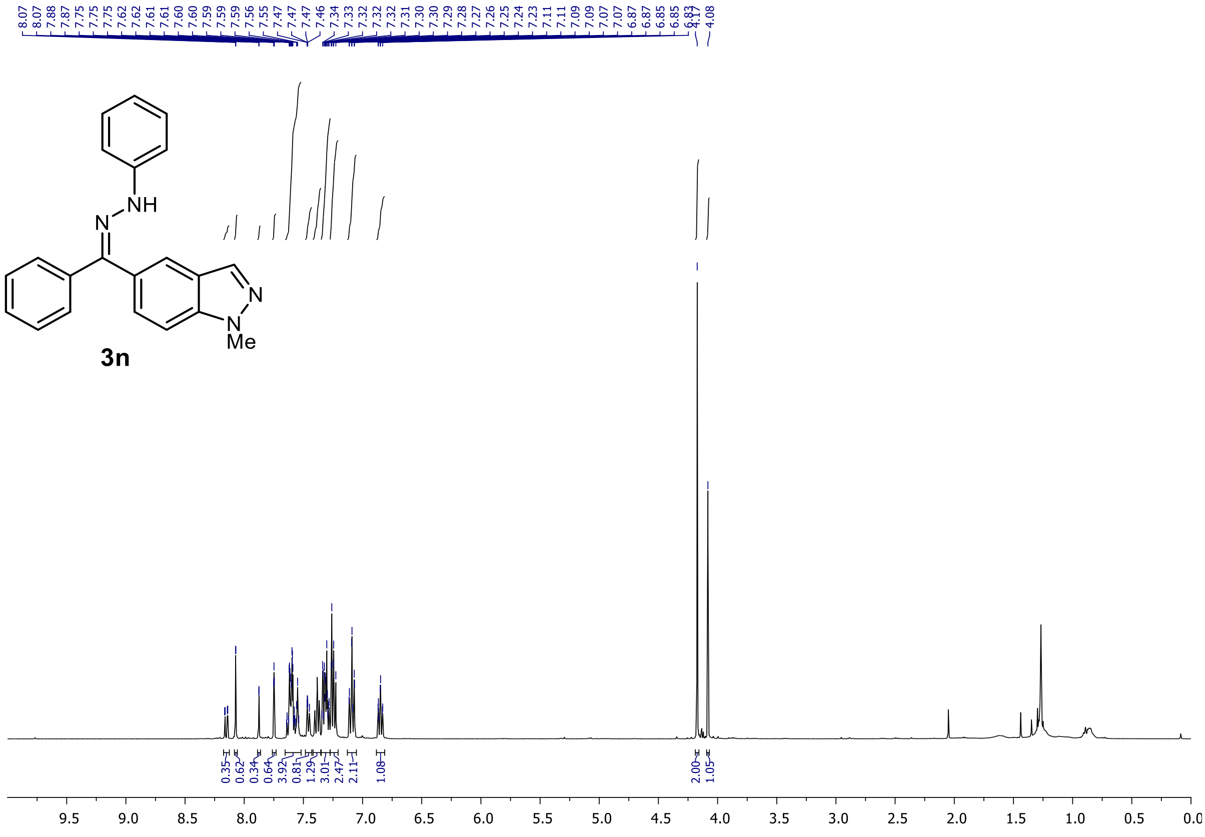

3n

^{13}C NMR (101 MHz, $CDCl_3$)

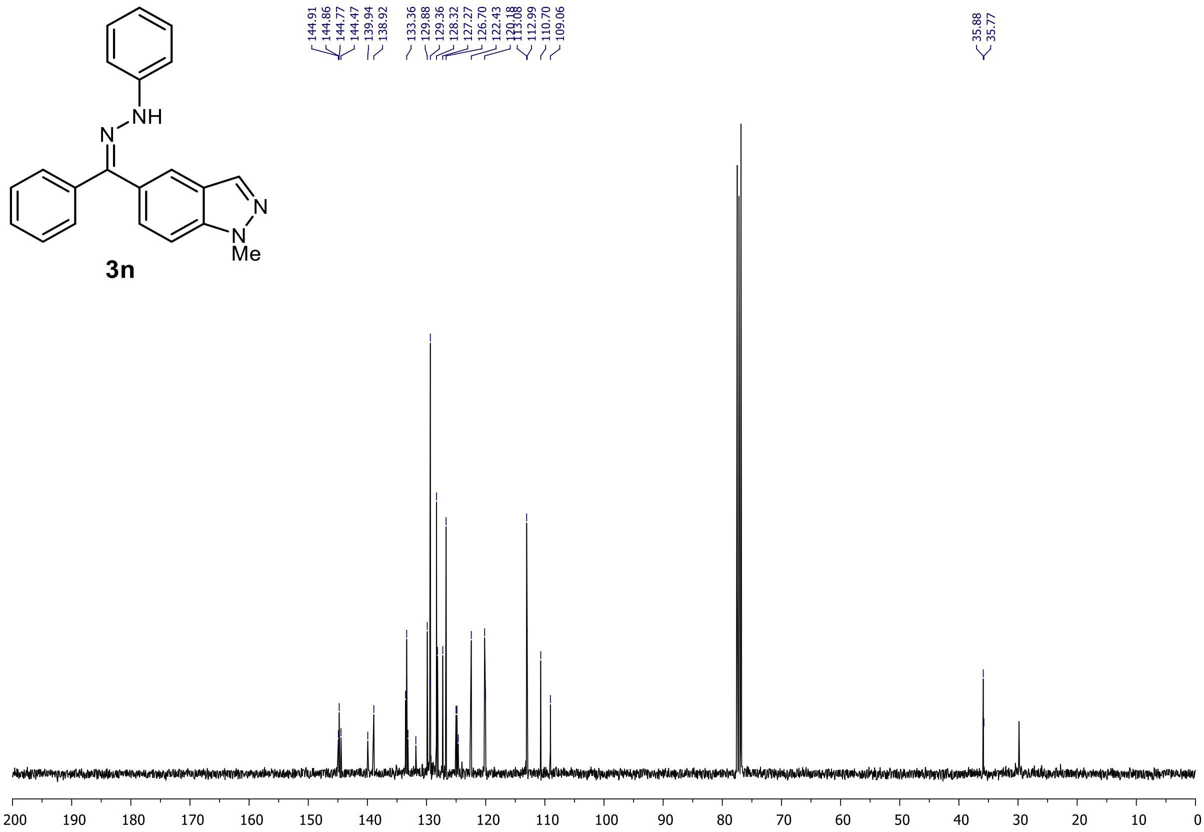

3n

^{1}H NMR (500 MHz, $CDCl_3$)

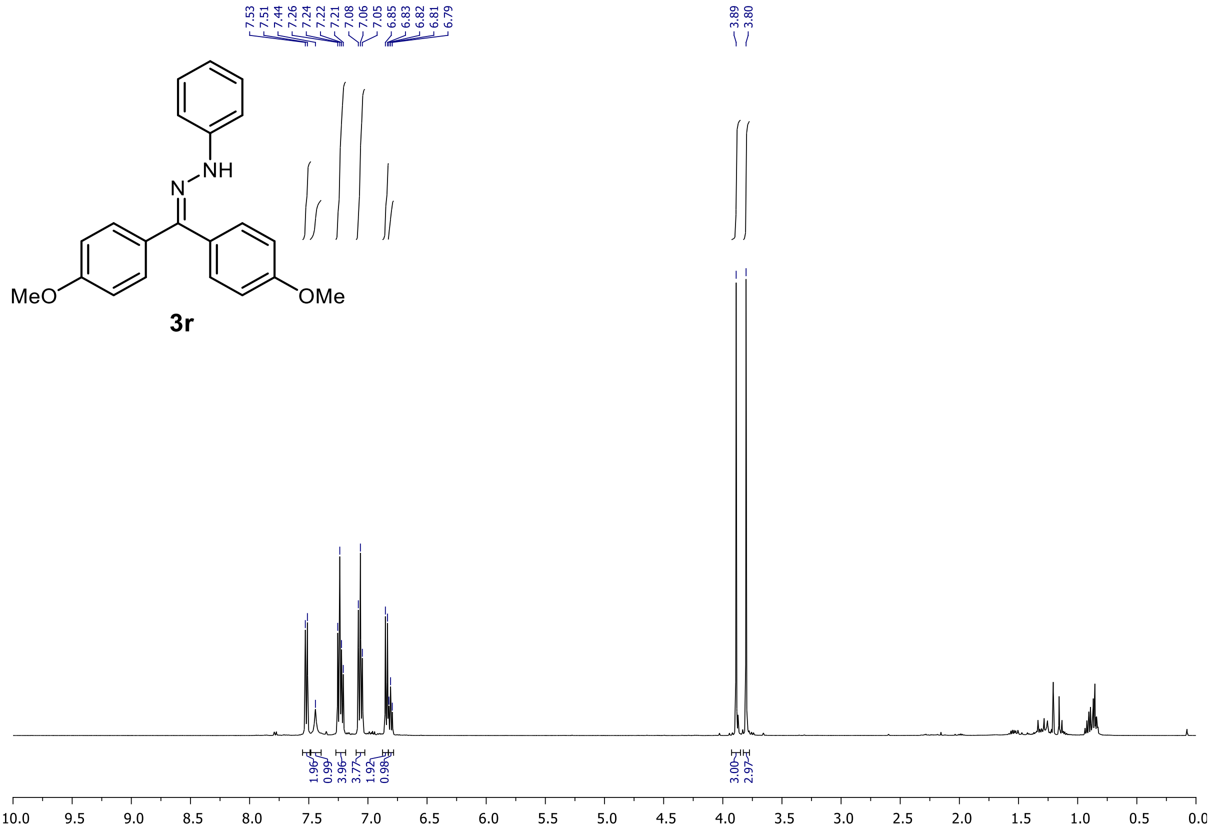

3r

^{13}C NMR (101 MHz, $CDCl_3$)

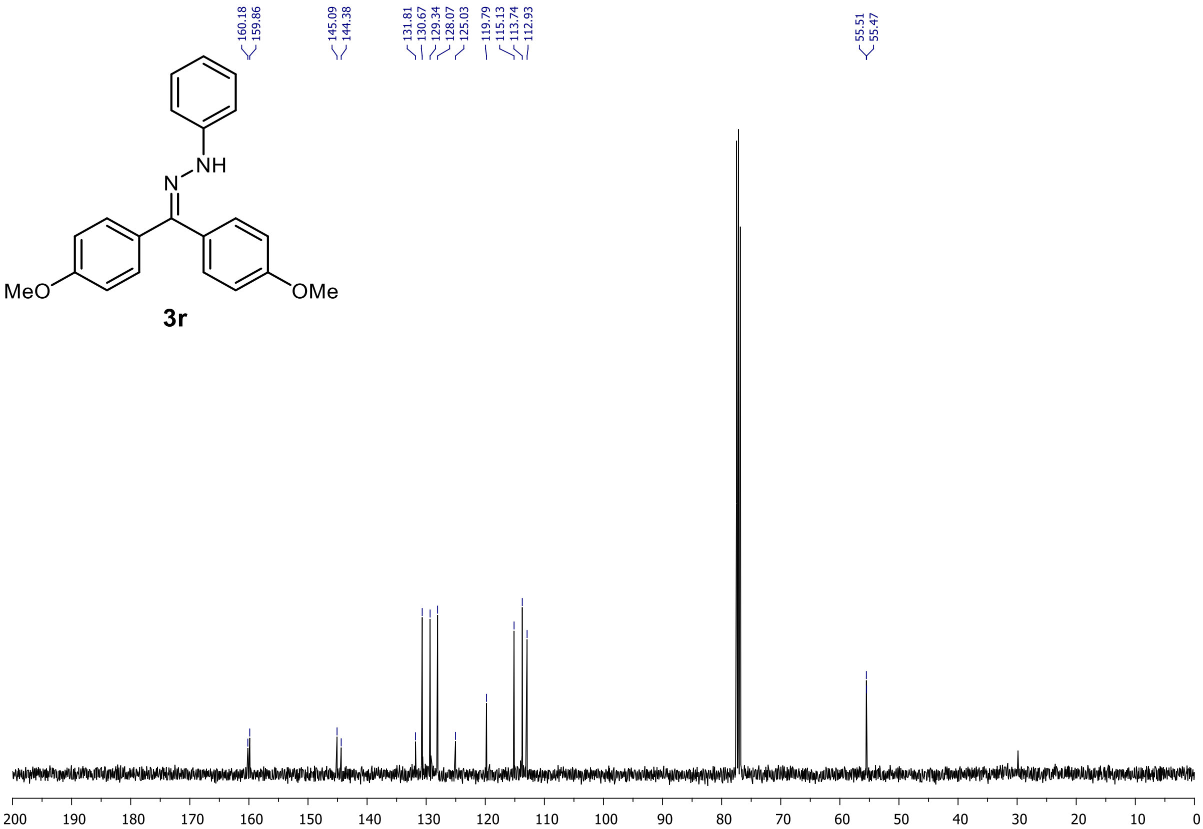

^{1}H NMR (500 MHz, $CDCl_3$)

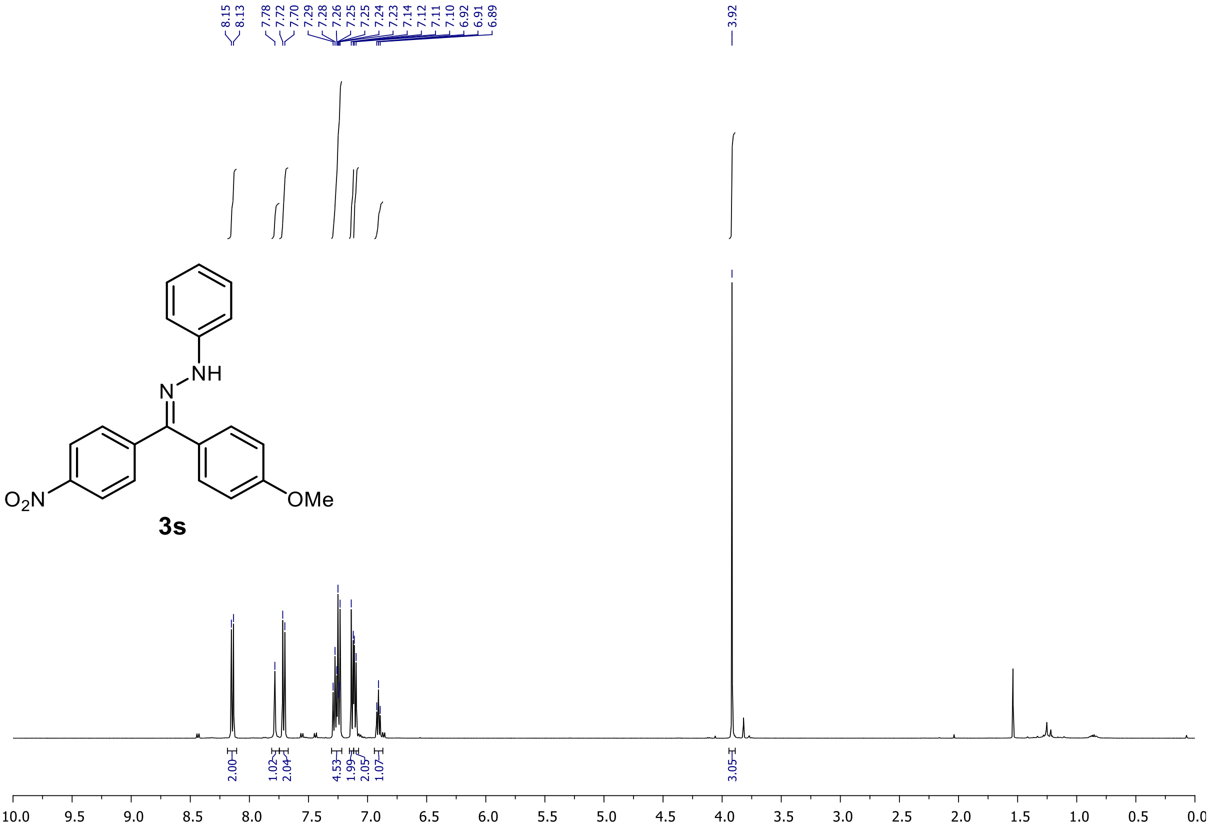

^{13}C NMR (101 MHz, $CDCl_3$)

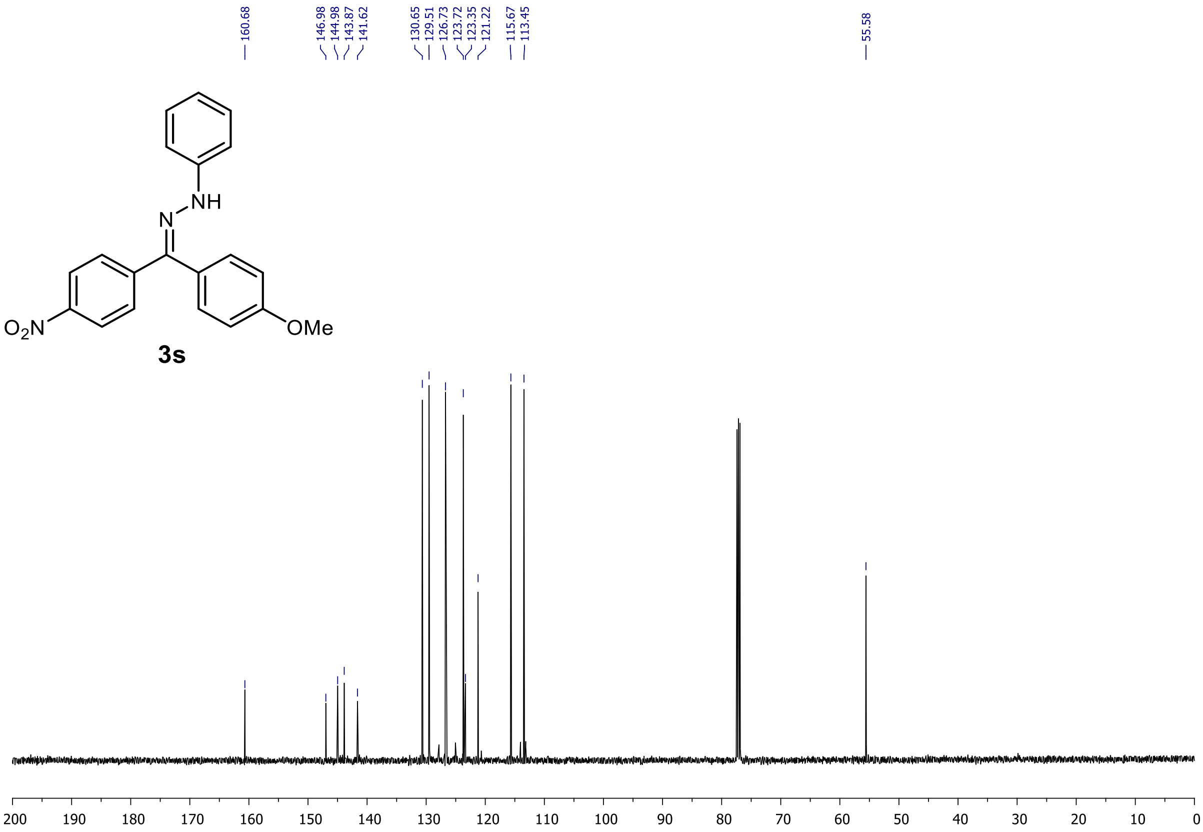

3s

^{1}H NMR (400 MHz, $CDCl_3$)

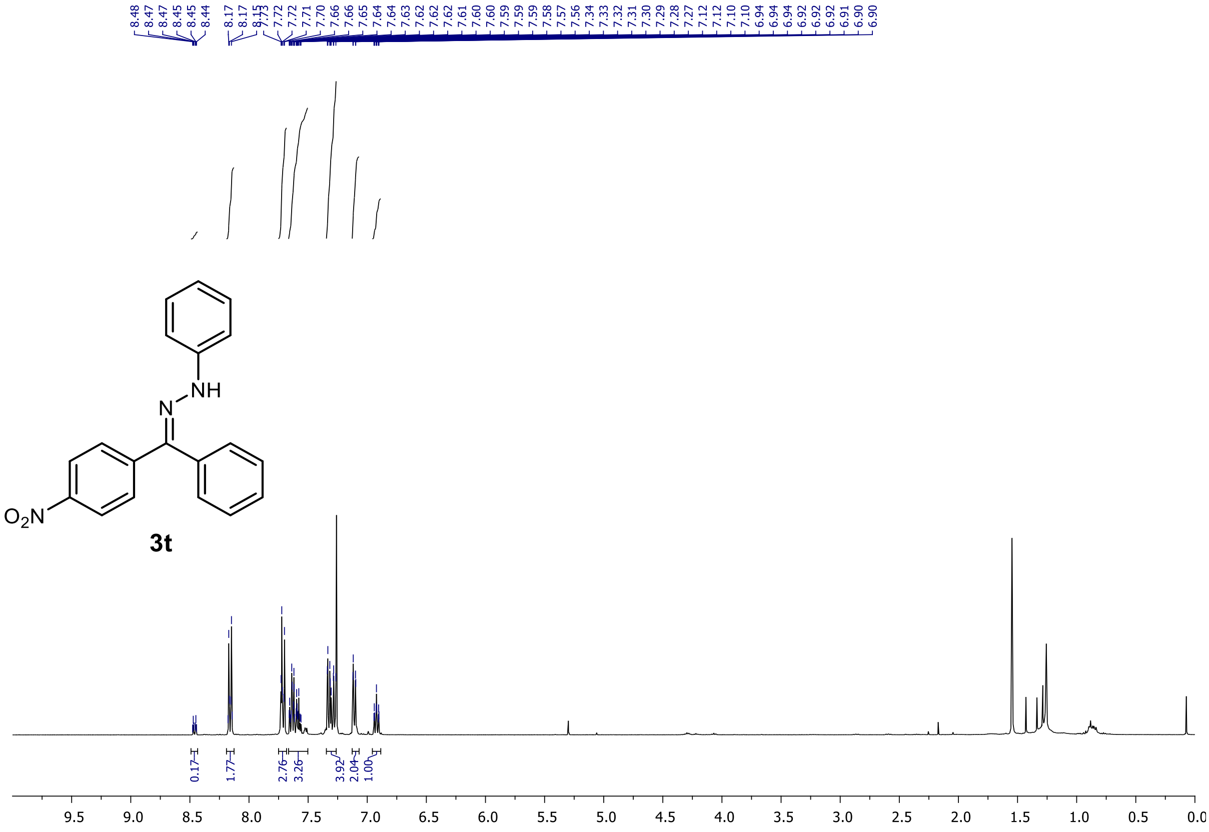

3t

^{13}C NMR (101 MHz, $CDCl_3$)

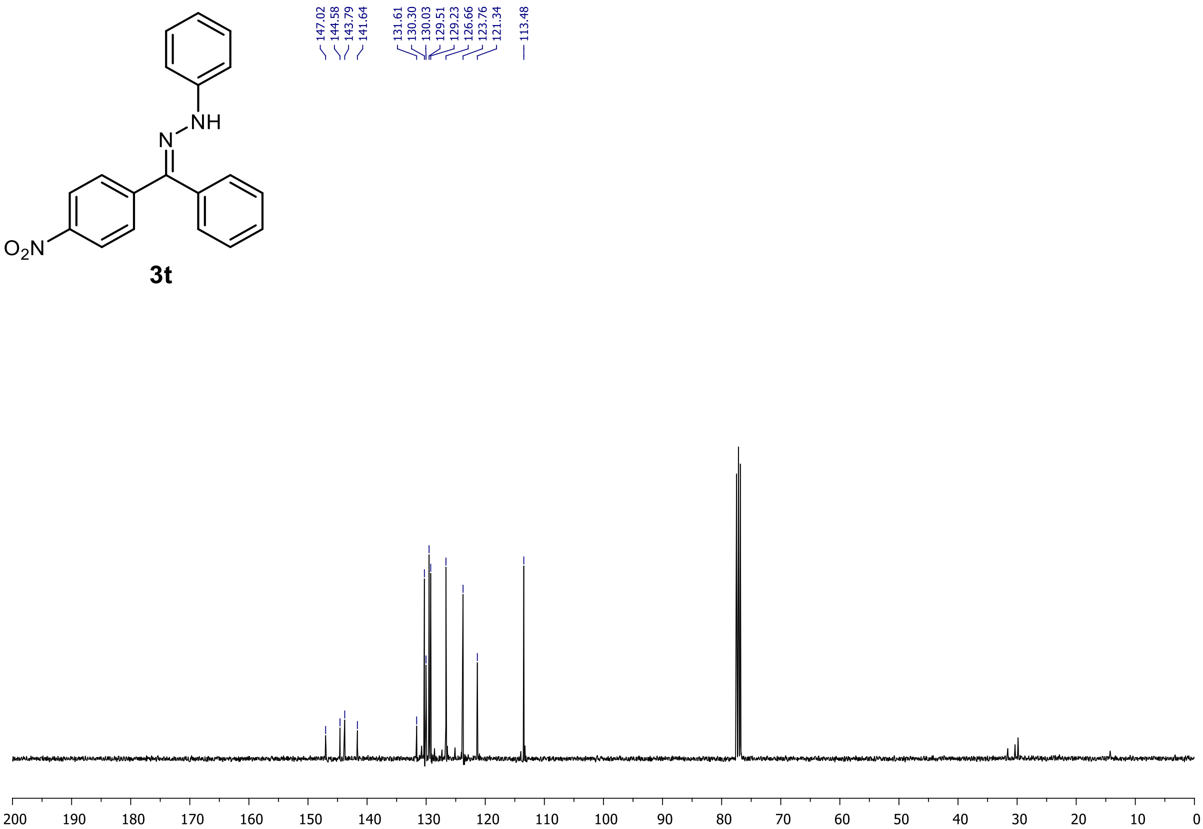

3t

^{1}H NMR (500 MHz, $CDCl_3$)

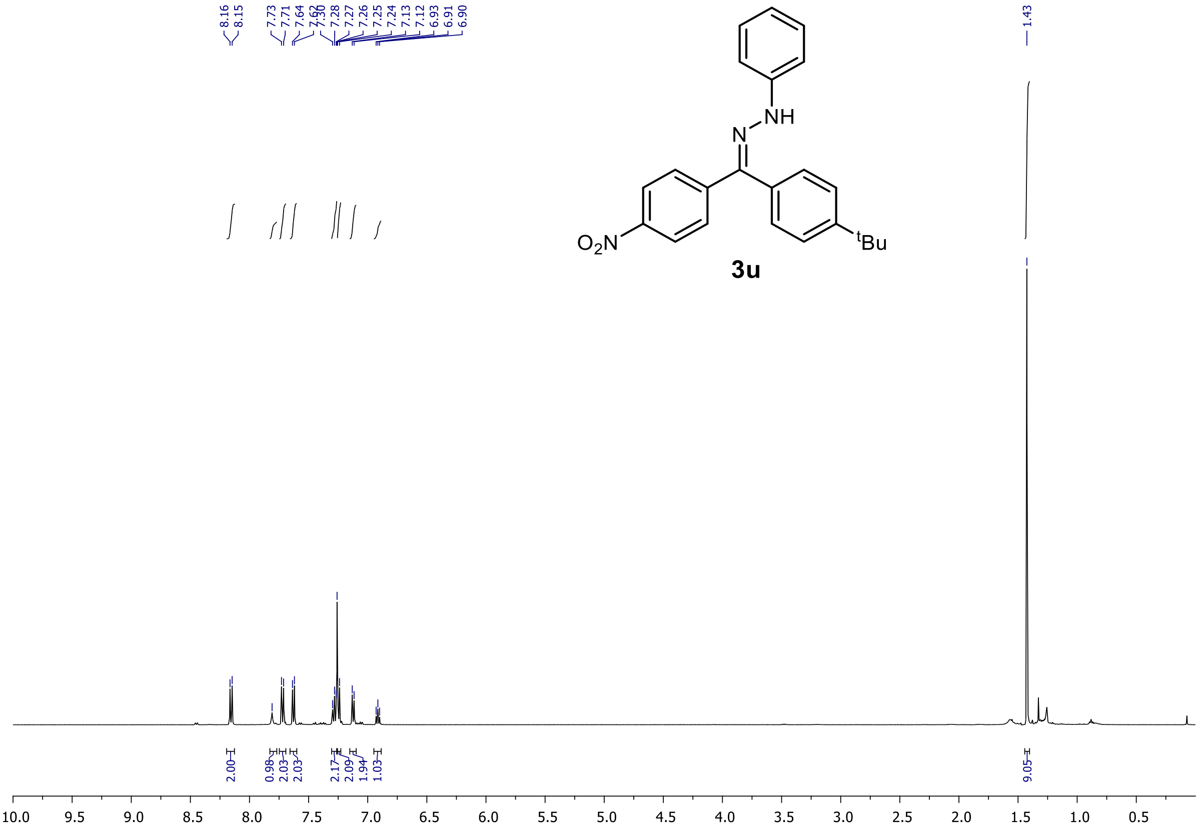

3u

^{13}C NMR (101 MHz, $CDCl_3$)

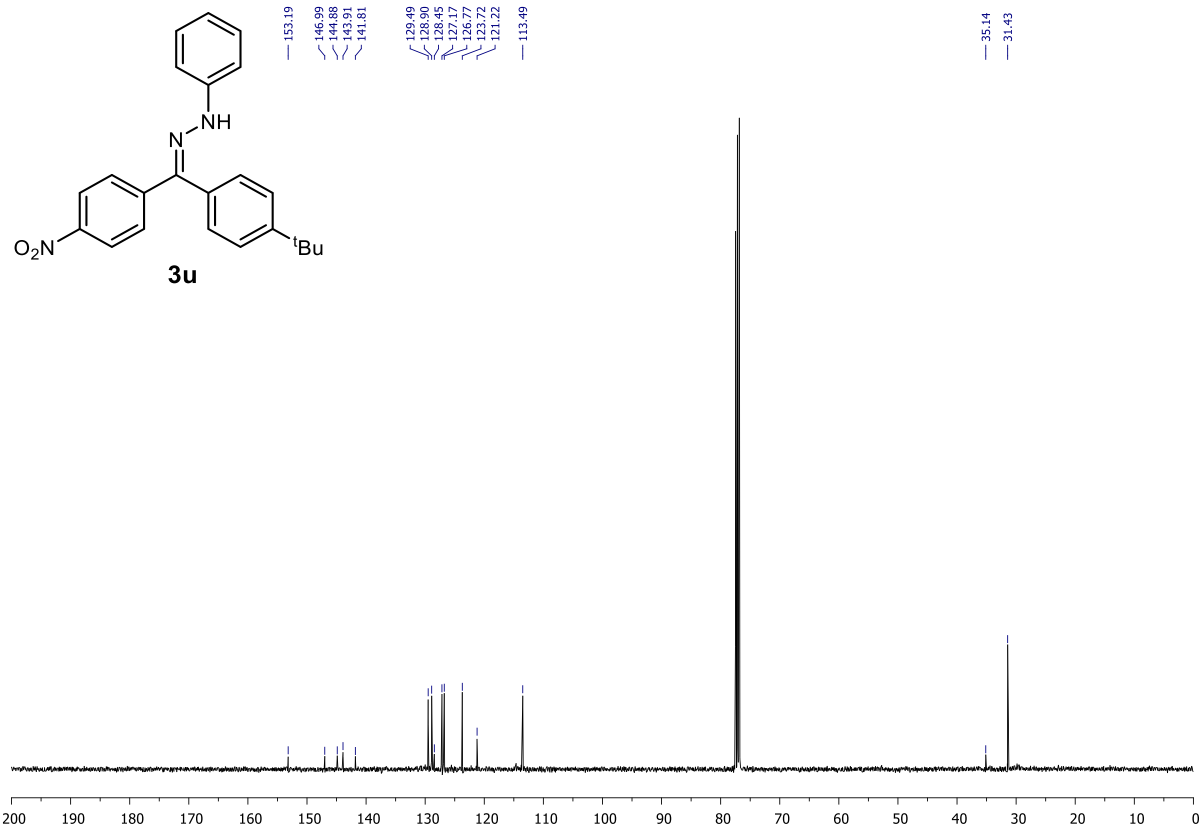

^{1}H NMR (500 MHz, $CDCl_3$)

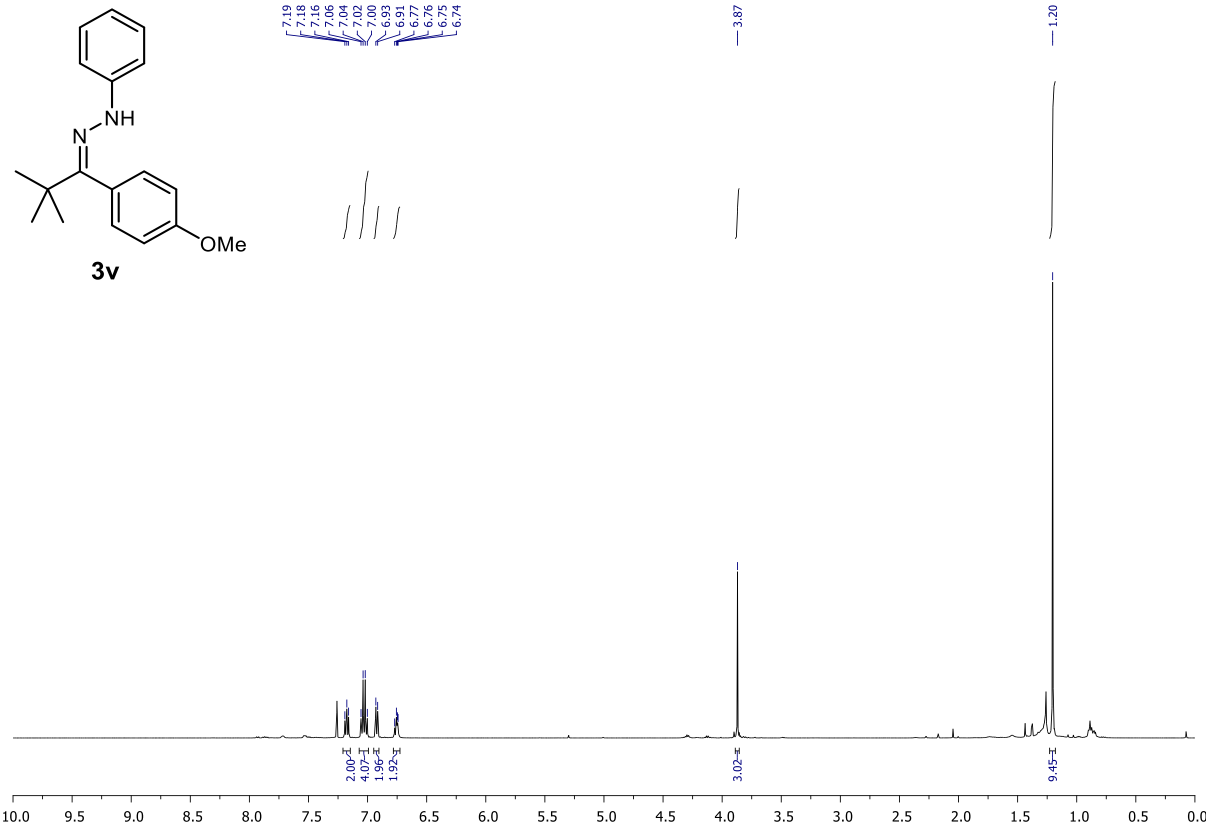

^{13}C NMR (126 MHz, $CDCl_3$)

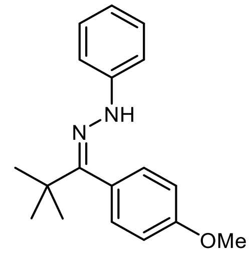

3v

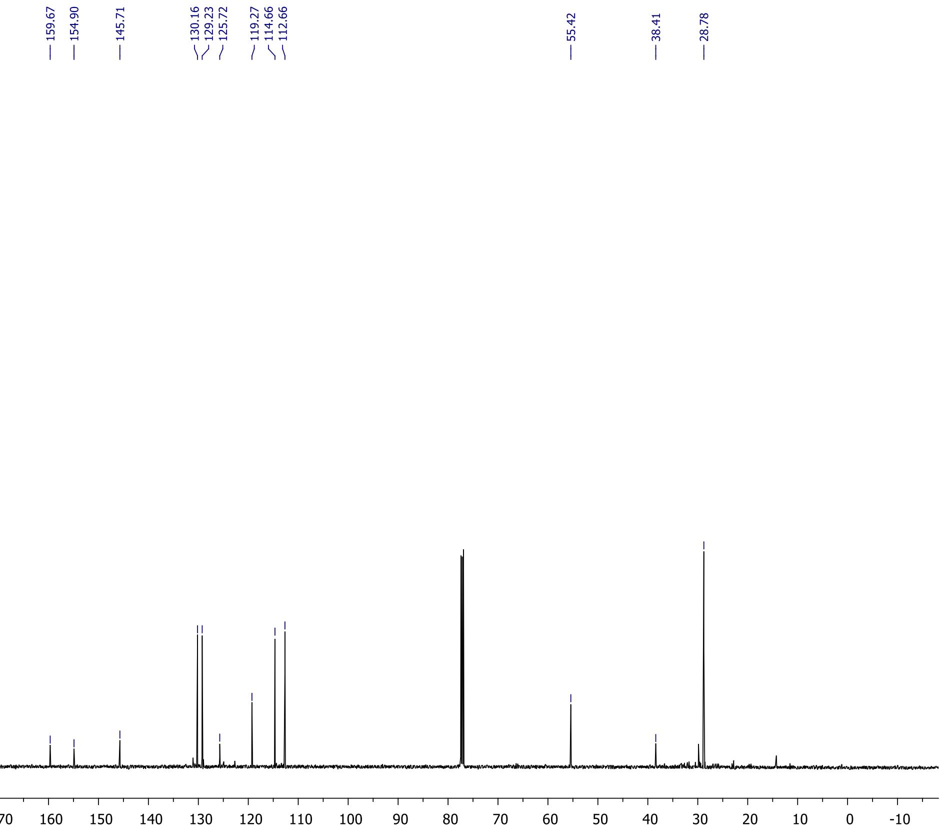

^{1}H NMR (500 MHz, $CDCl_3$)

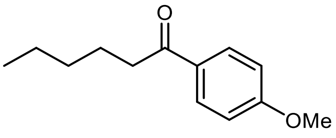

3w

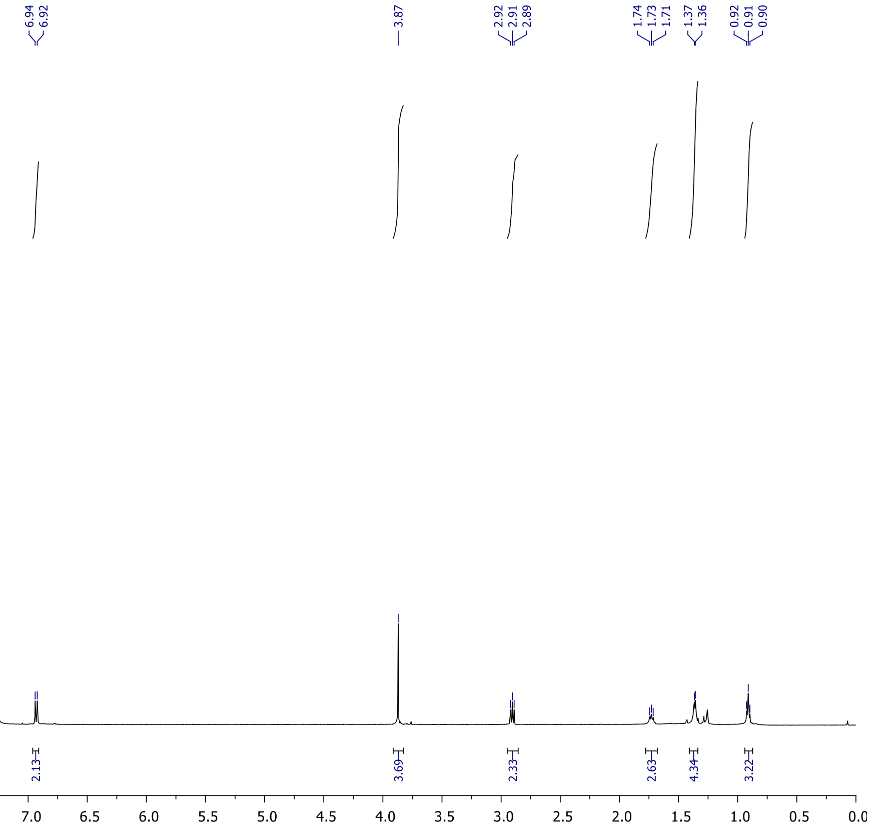

^{13}C NMR (126 MHz, $CDCl_3$)

3w

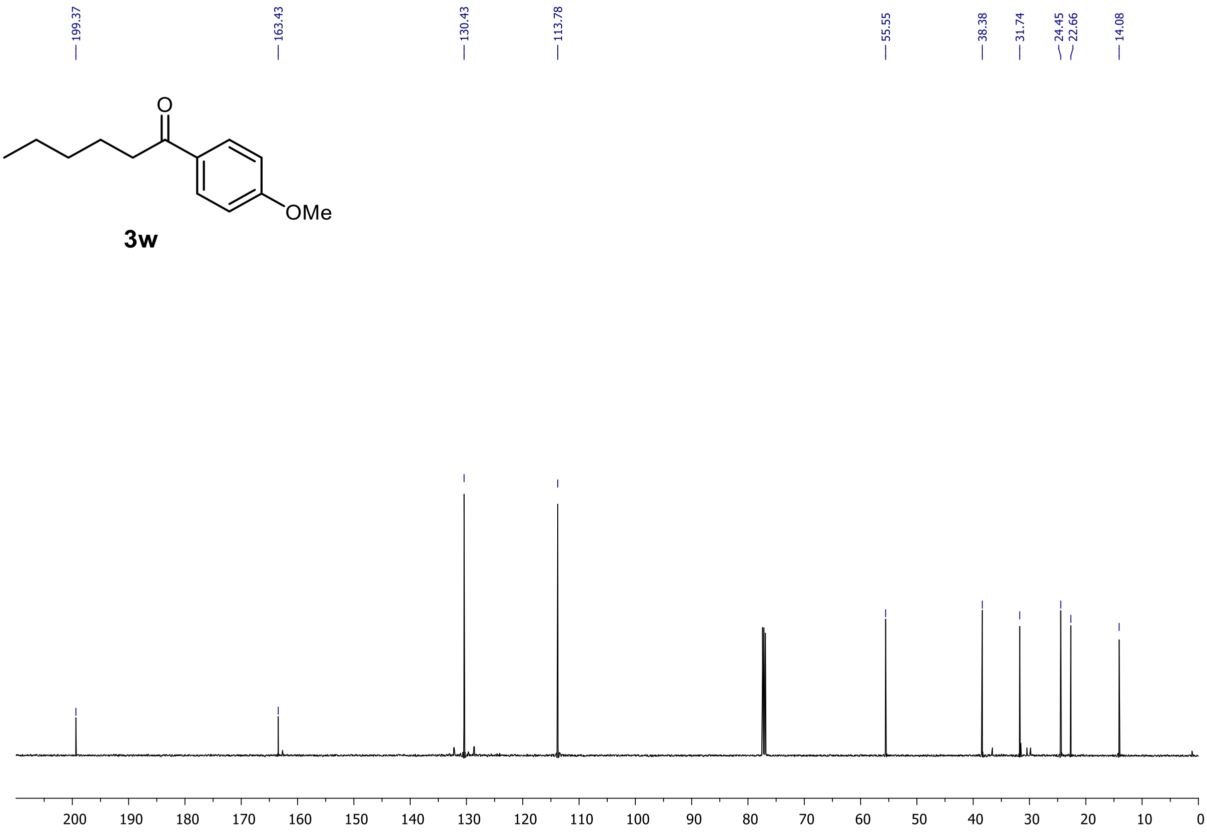

^{1}H NMR (500 MHz, $CDCl_3$)

4

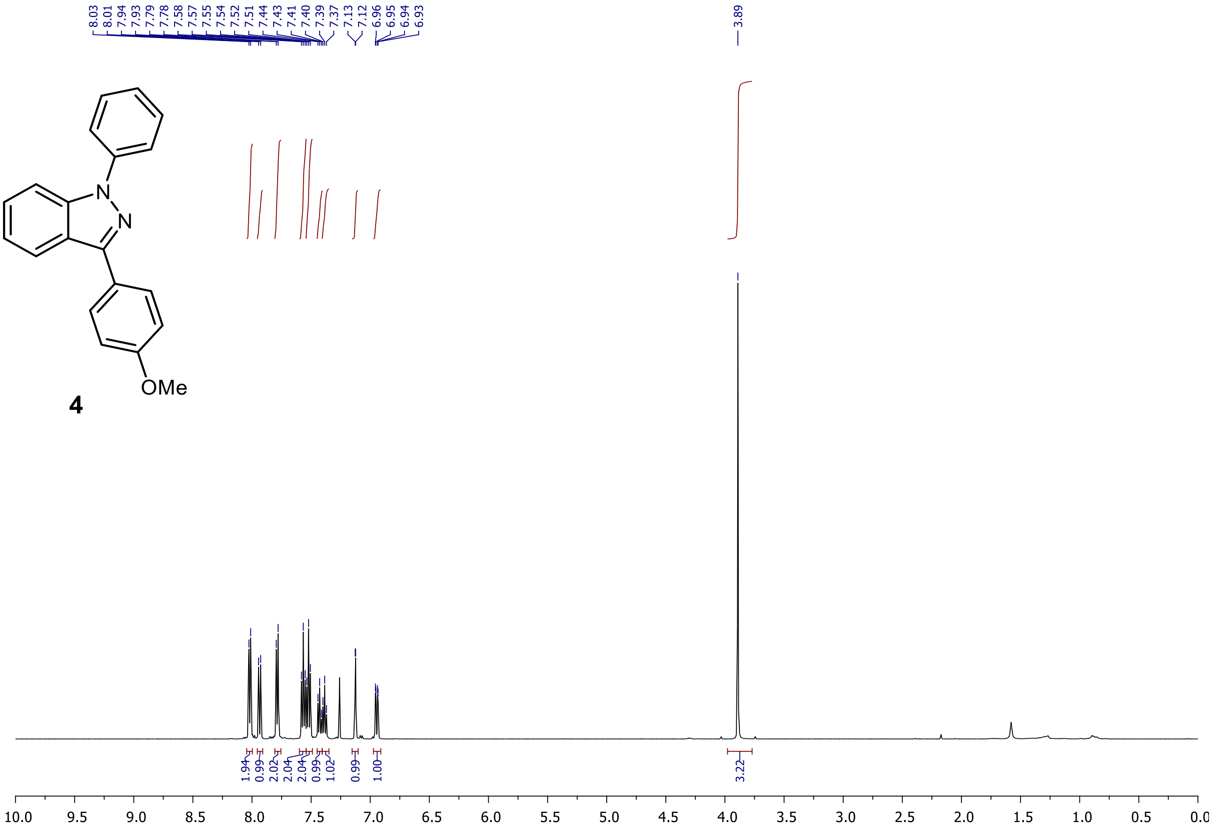

^{13}C NMR (126 MHz, $CDCl_3$)

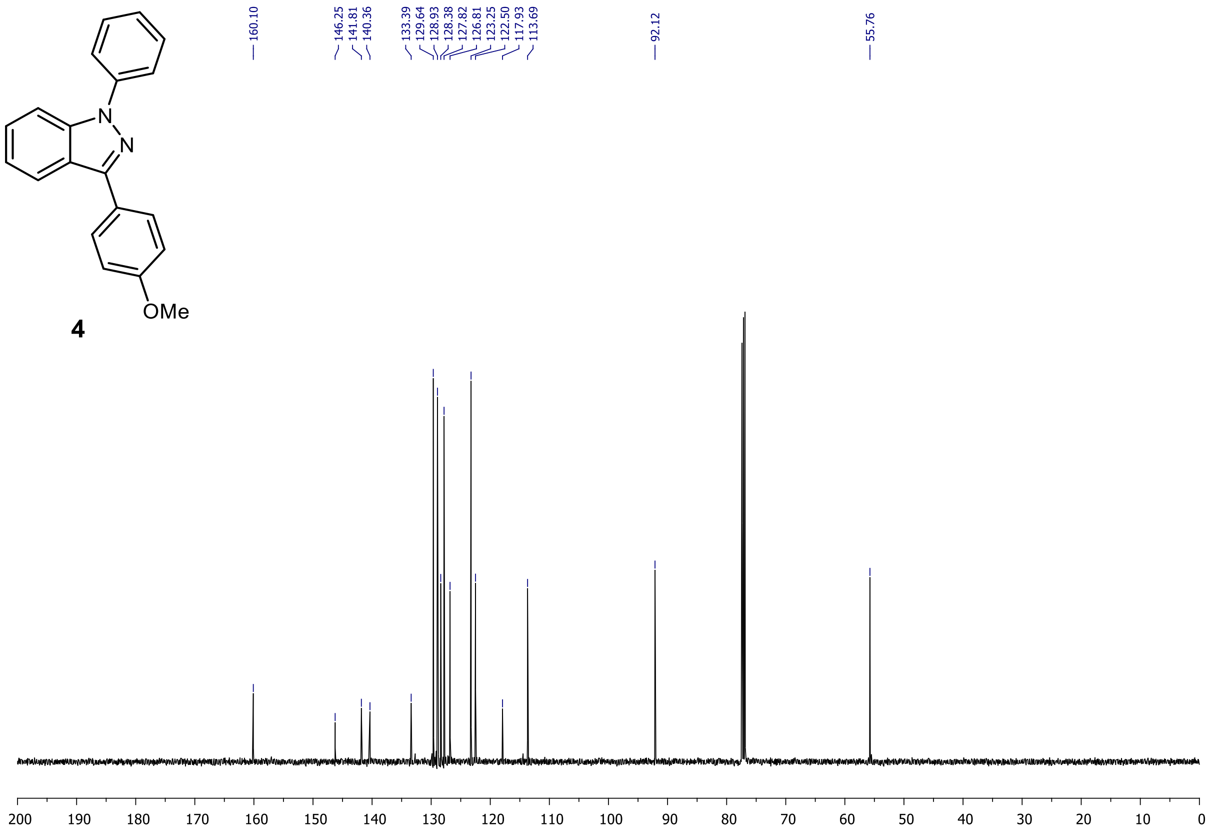

^{1}H NMR (500 MHz, acetone-d_6)

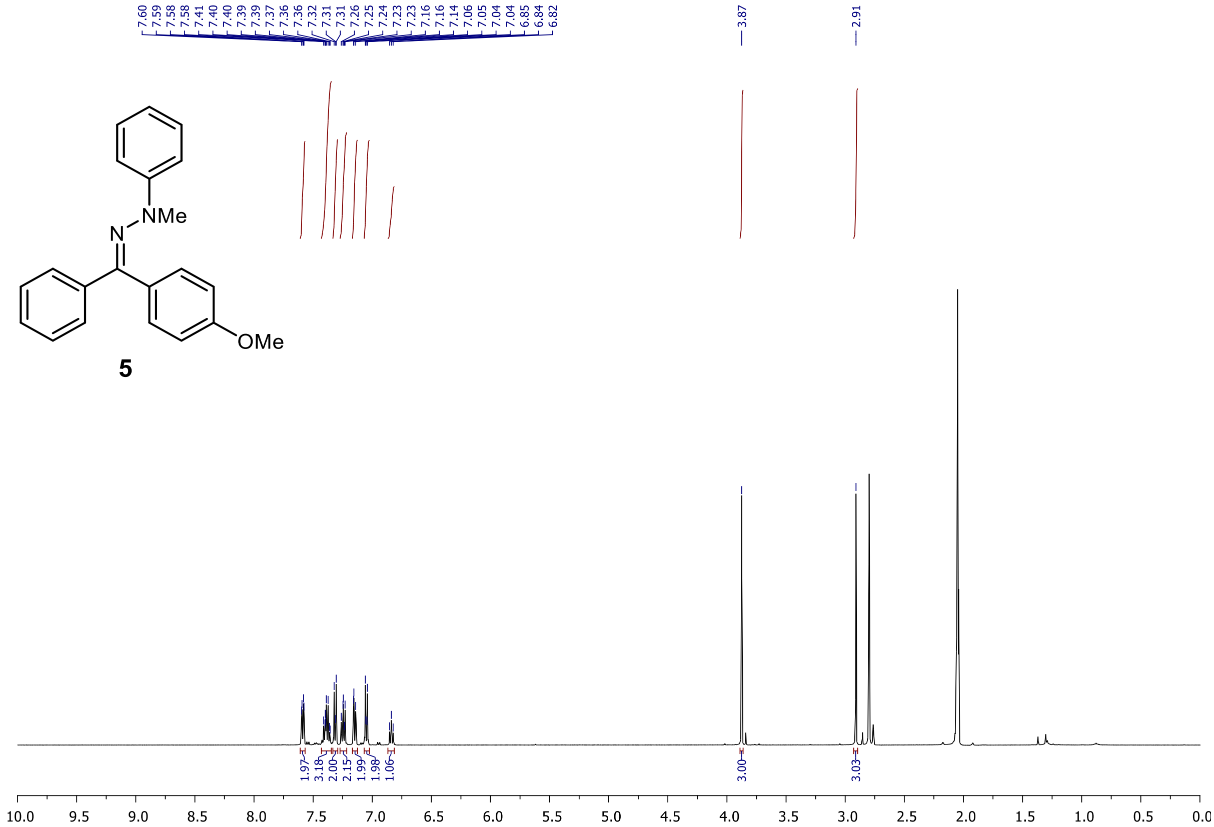

^{13}C NMR (101 MHz, acetone-d_6)

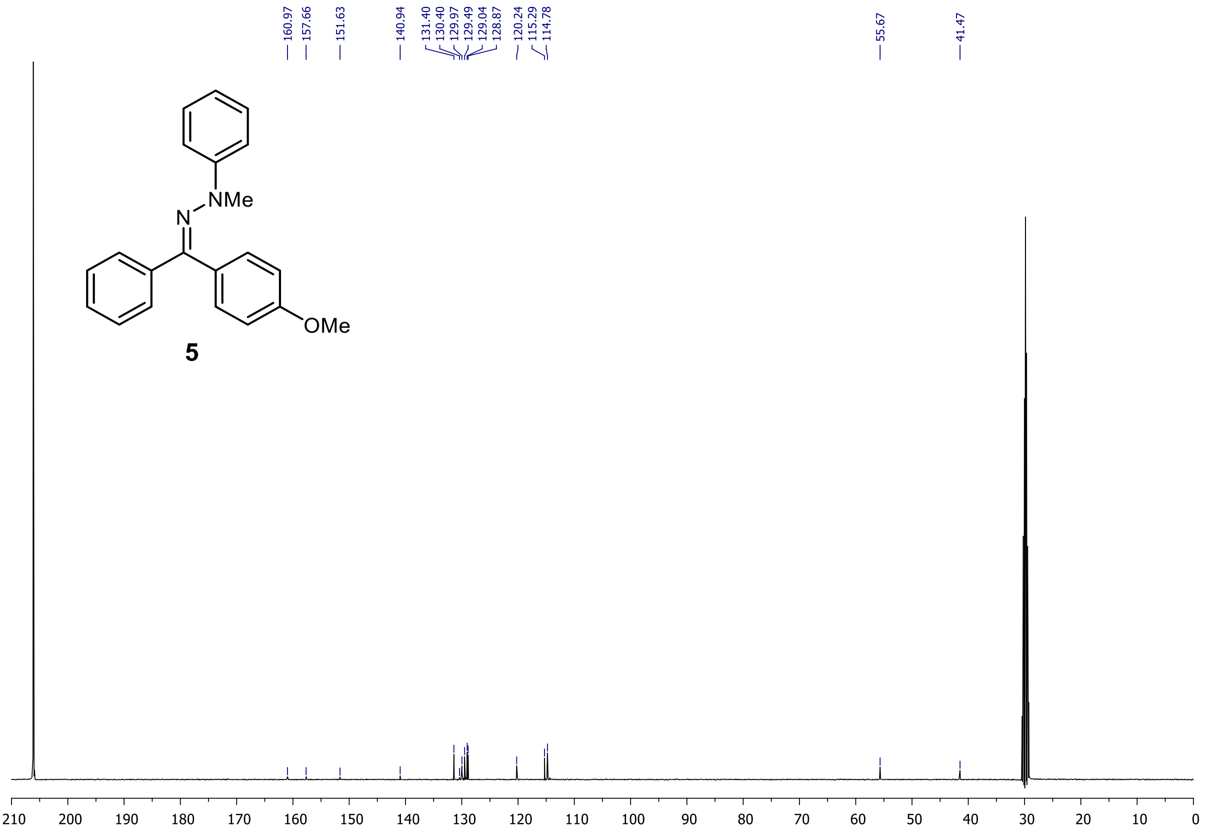

^{1}H NMR (500 MHz, $CDCl_3$)

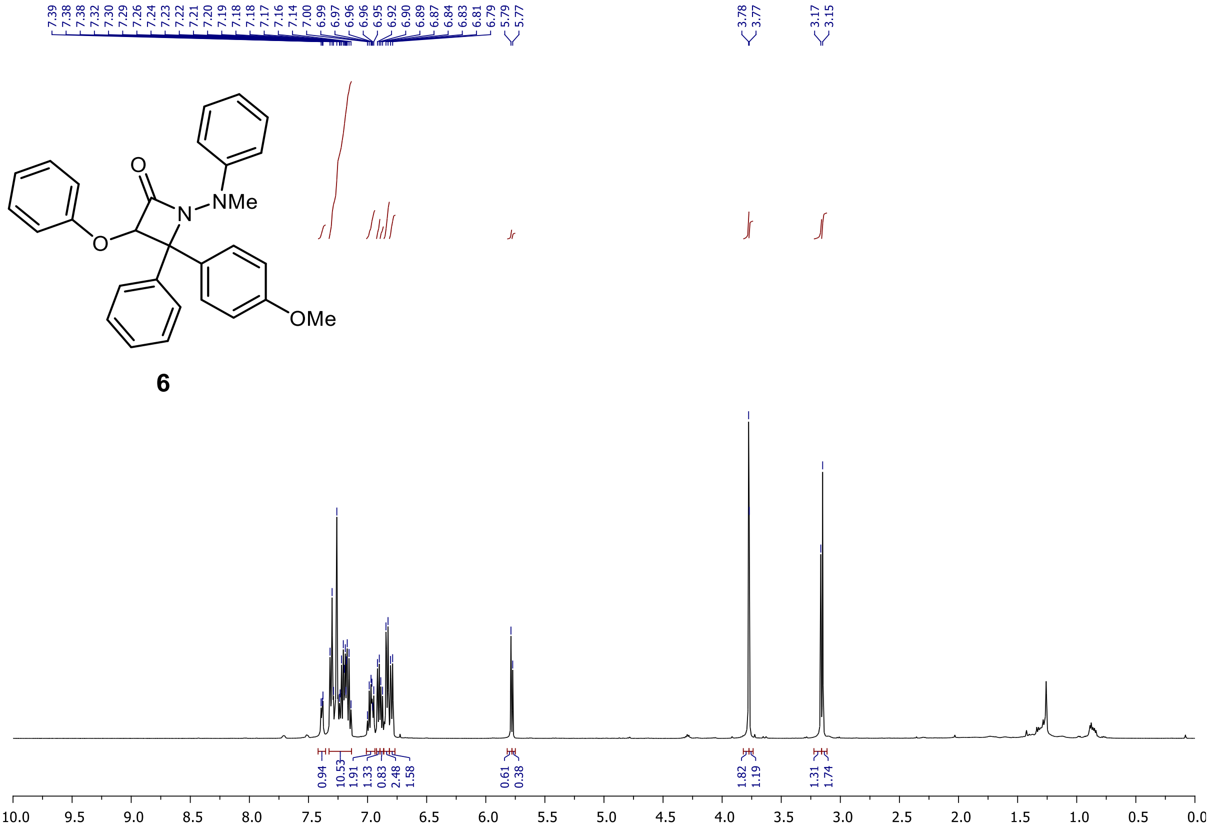

^{13}C NMR (126 MHz, $CDCl_3$)

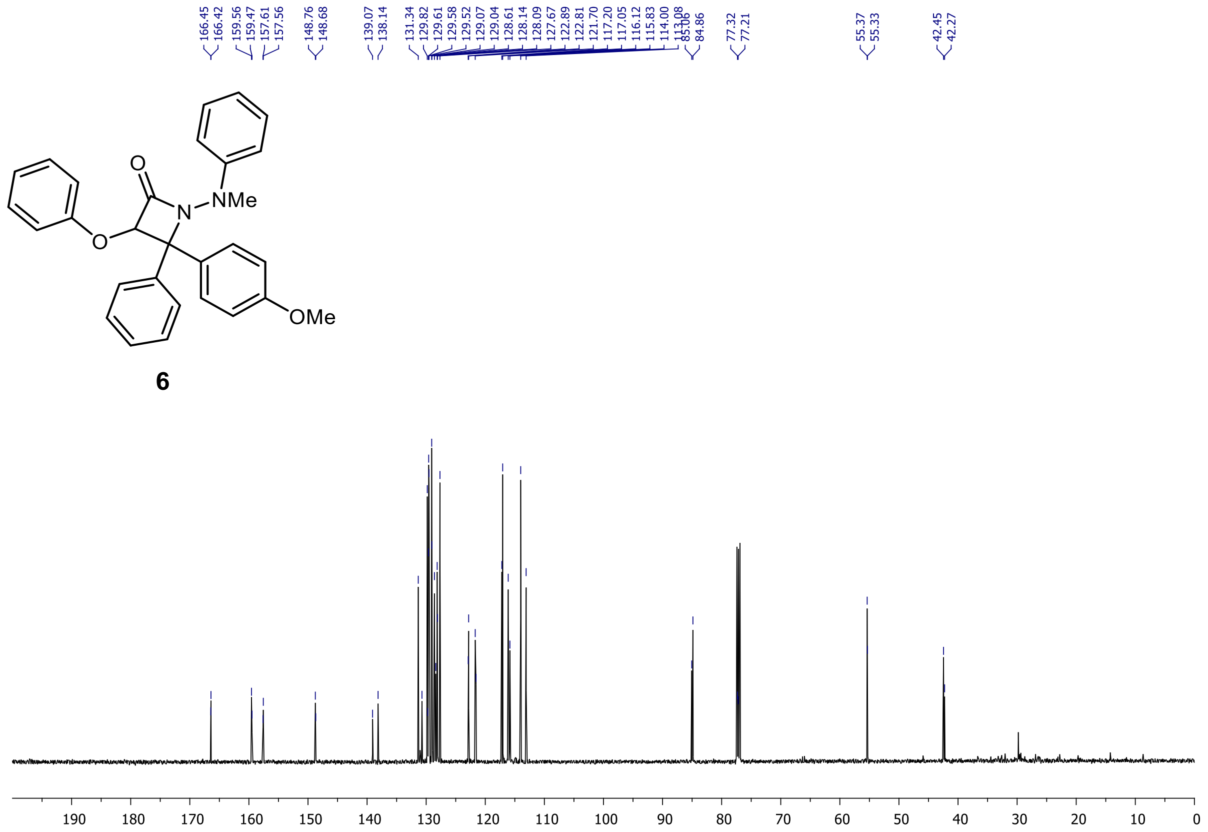

6

^{1}H NMR (500 MHz, acetone-d_6)

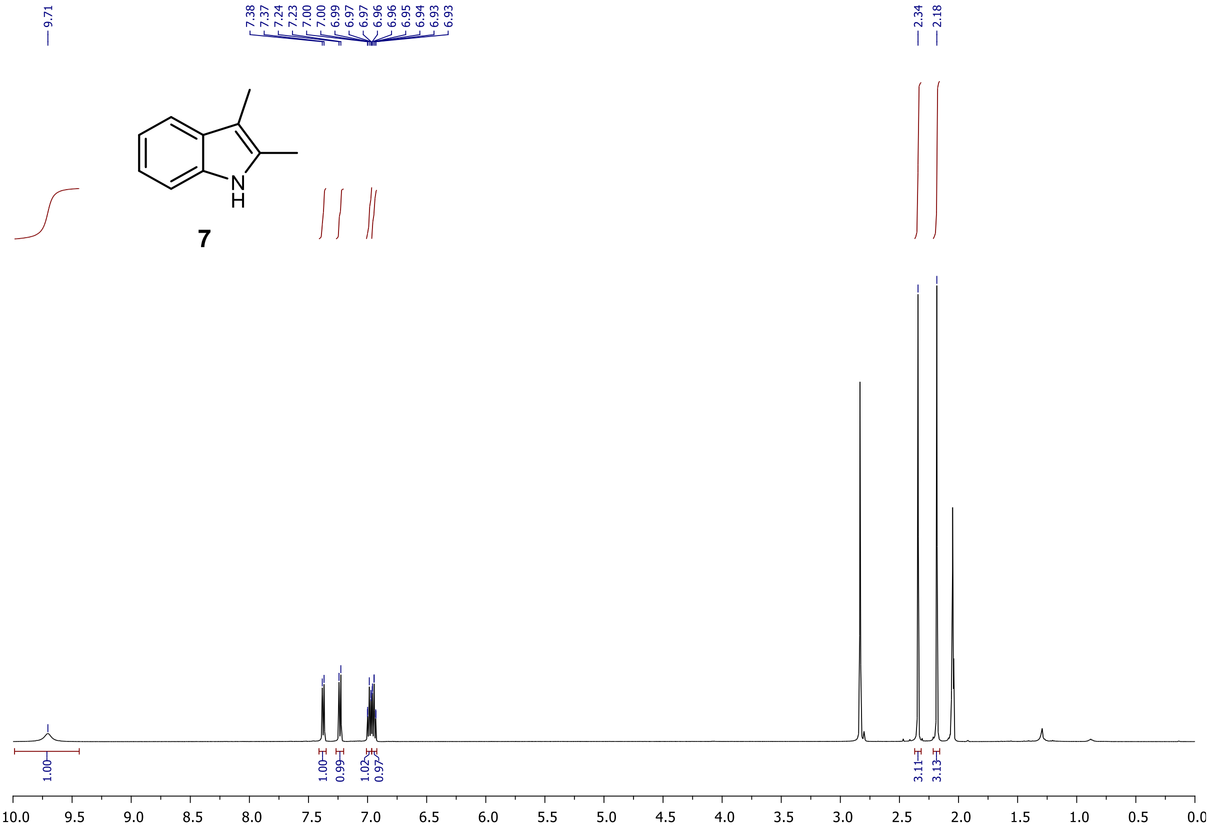

7

^{13}C NMR (101 MHz, acetone-d_6)

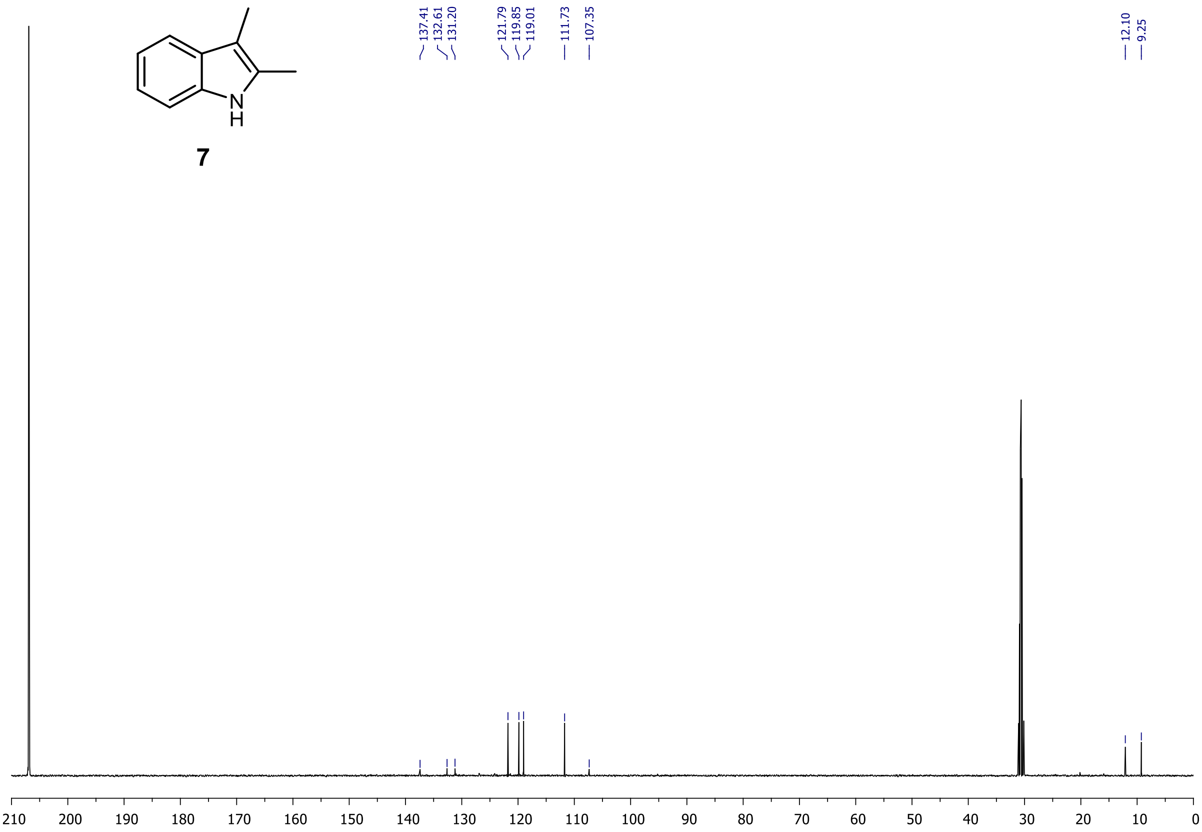

^{1}H NMR (400 MHz, $CDCl_3$)

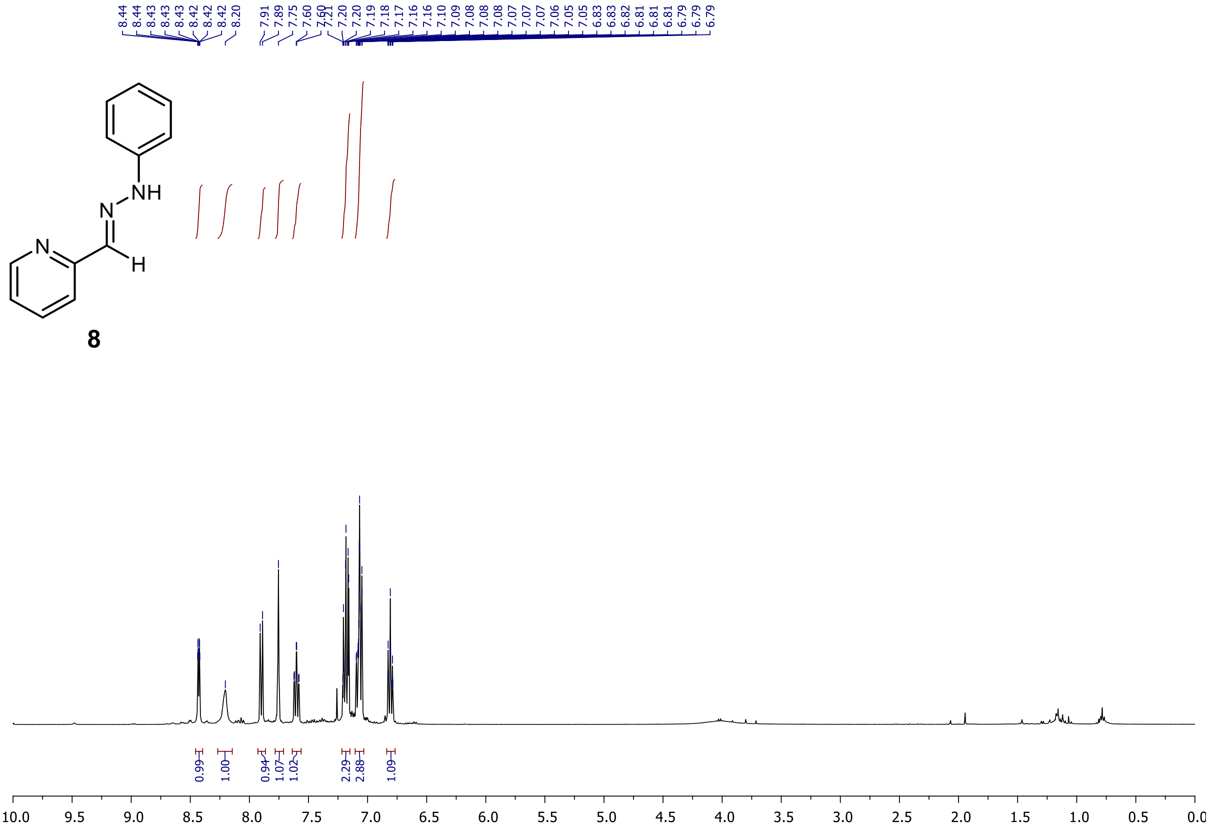

^{13}C NMR (101 MHz, $CDCl_3$)

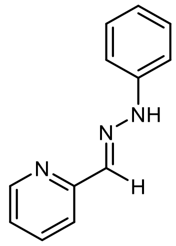

8

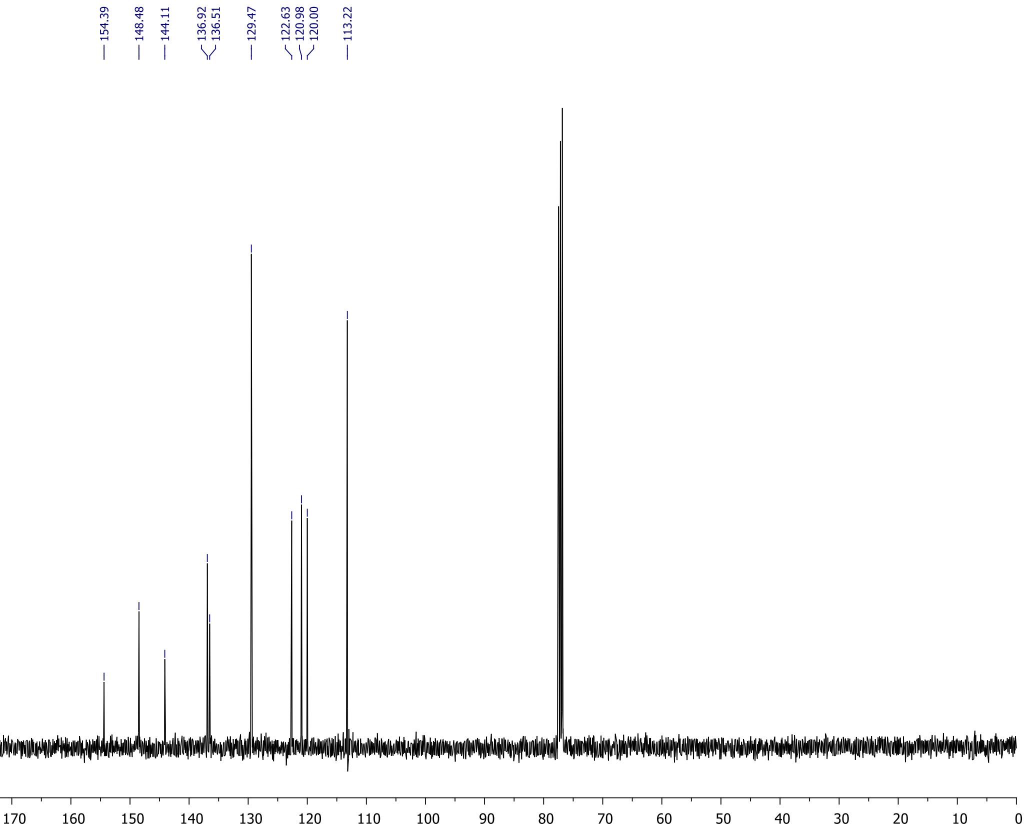

^{1}H NMR (500 MHz, $CDCl_3$)

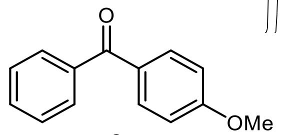

9

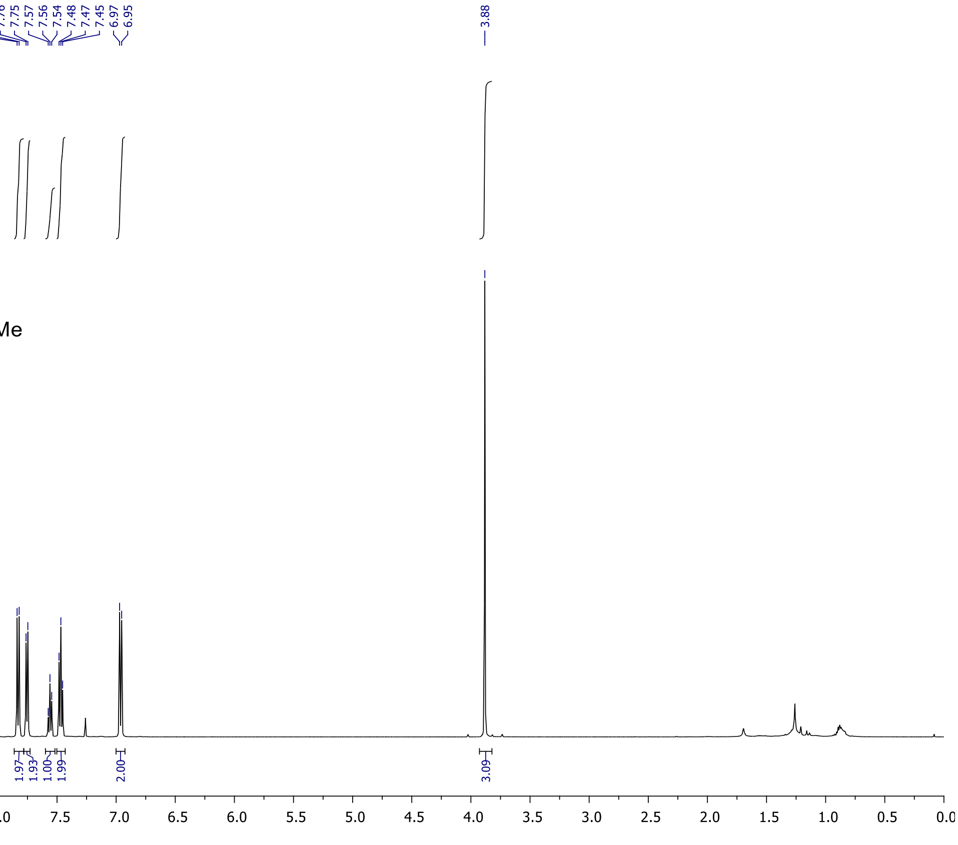

^{13}C NMR (126 MHz, $CDCl_3$)

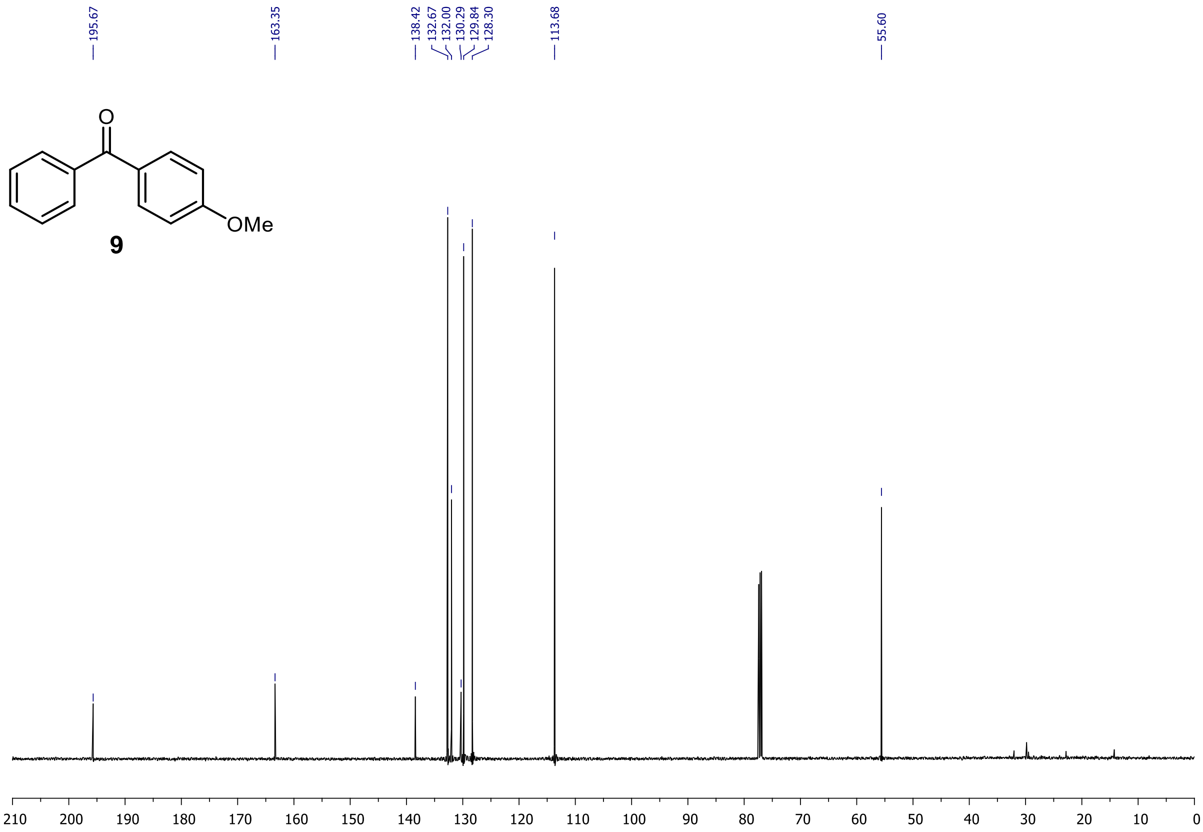

^{1}H NMR (500 MHz, acetone-d_6)

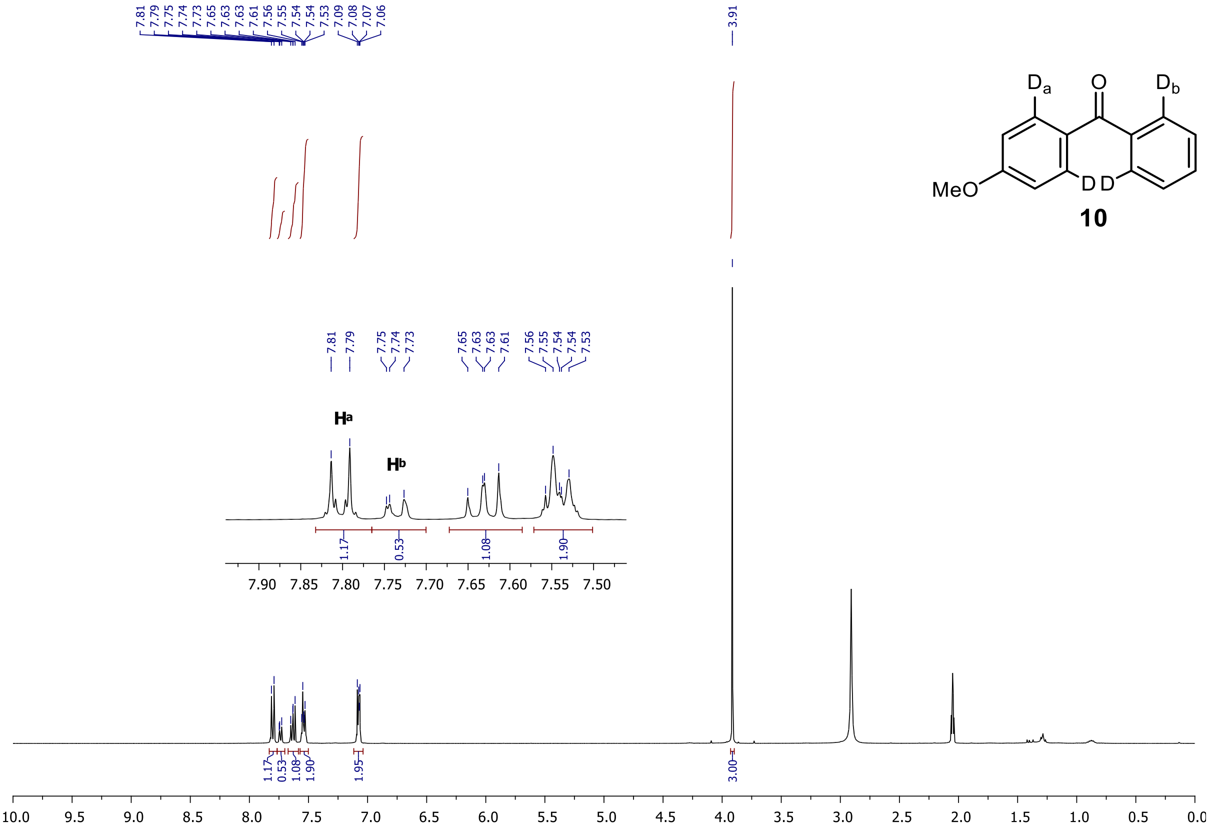

^{13}C NMR (126 MHz, acetone-d_6)

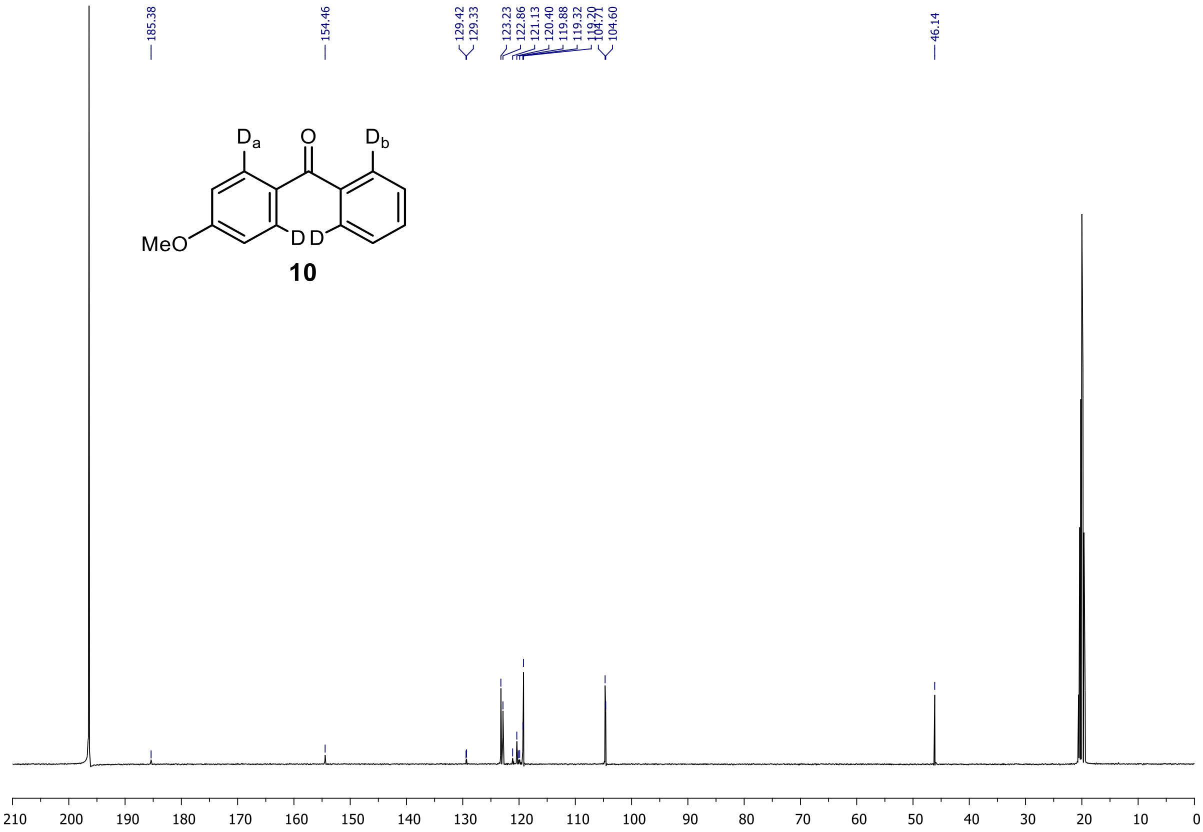

^{1}H NMR (500 MHz, $CDCl_3$)

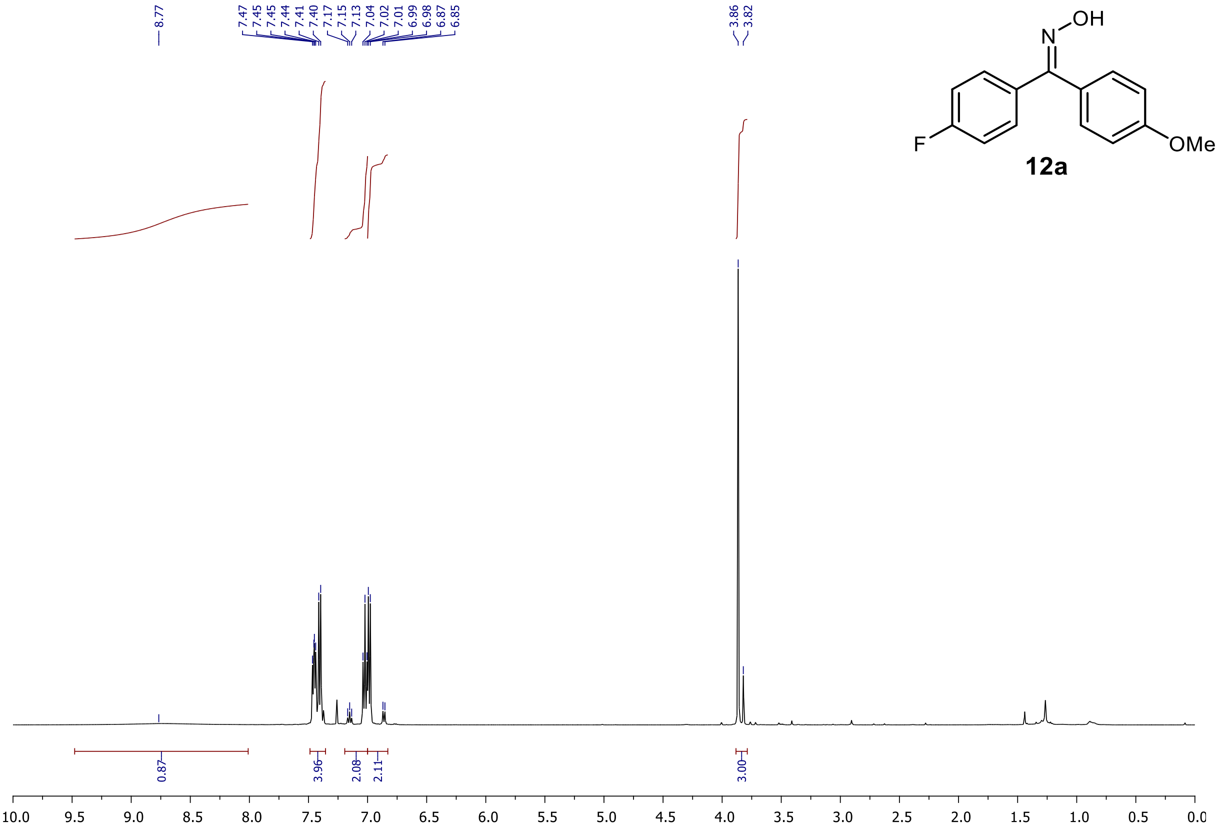

^{19}F NMR (471 MHz, $CDCl_3$)

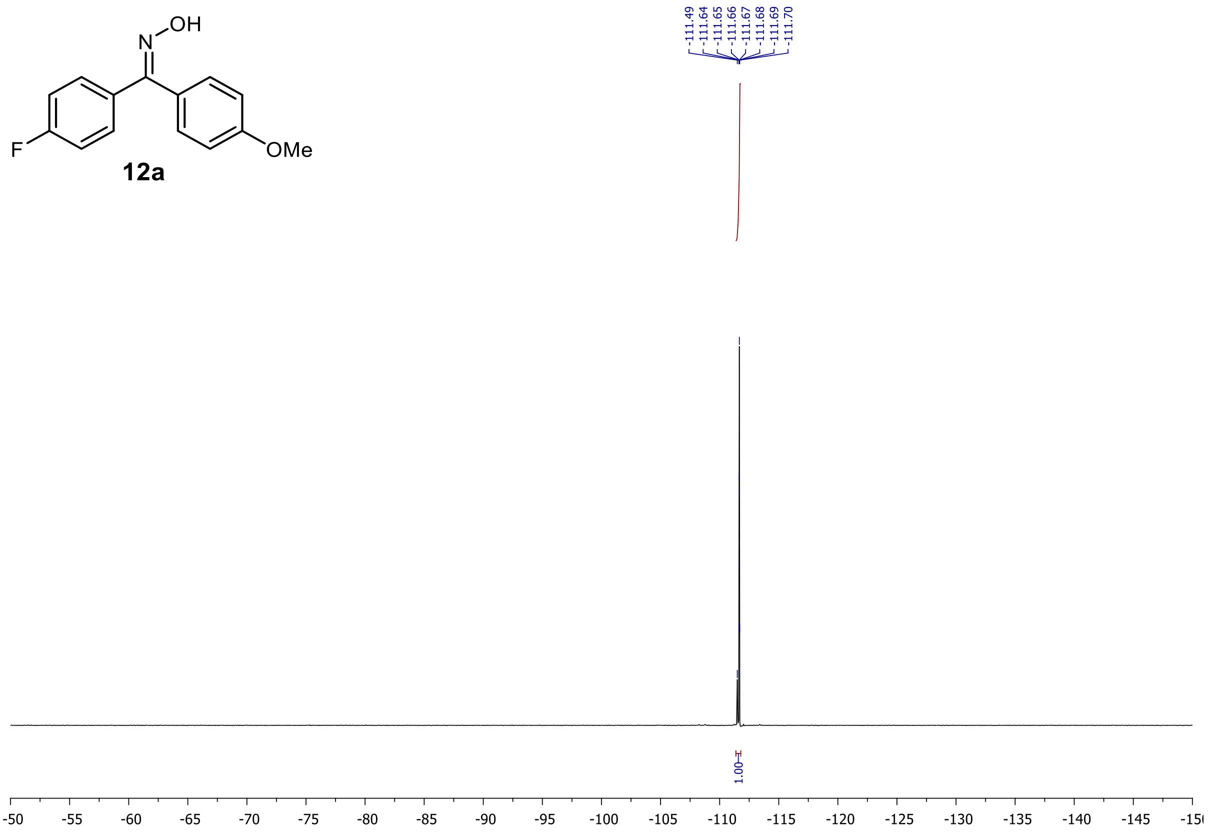

^{13}C NMR (126 MHz, $CDCl_3$)

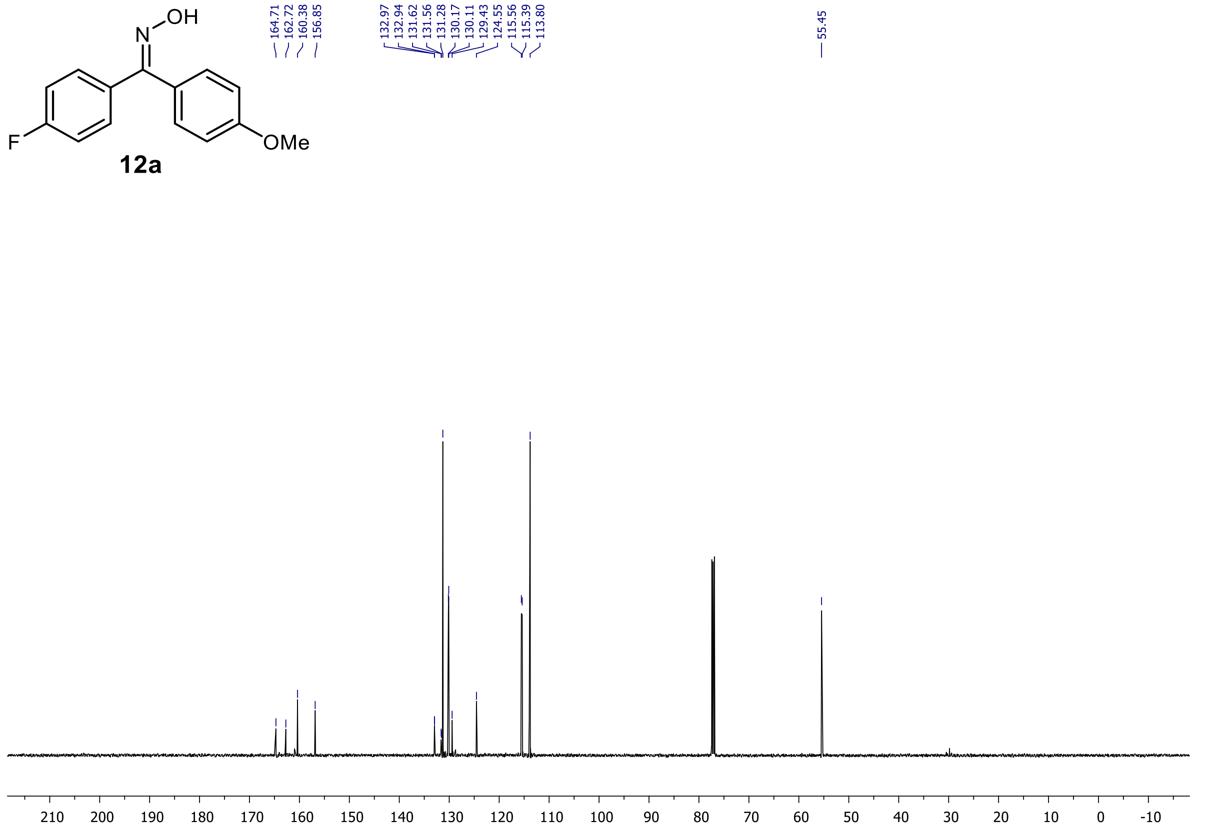

^{1}H NMR (400 MHz, $CDCl_3$)

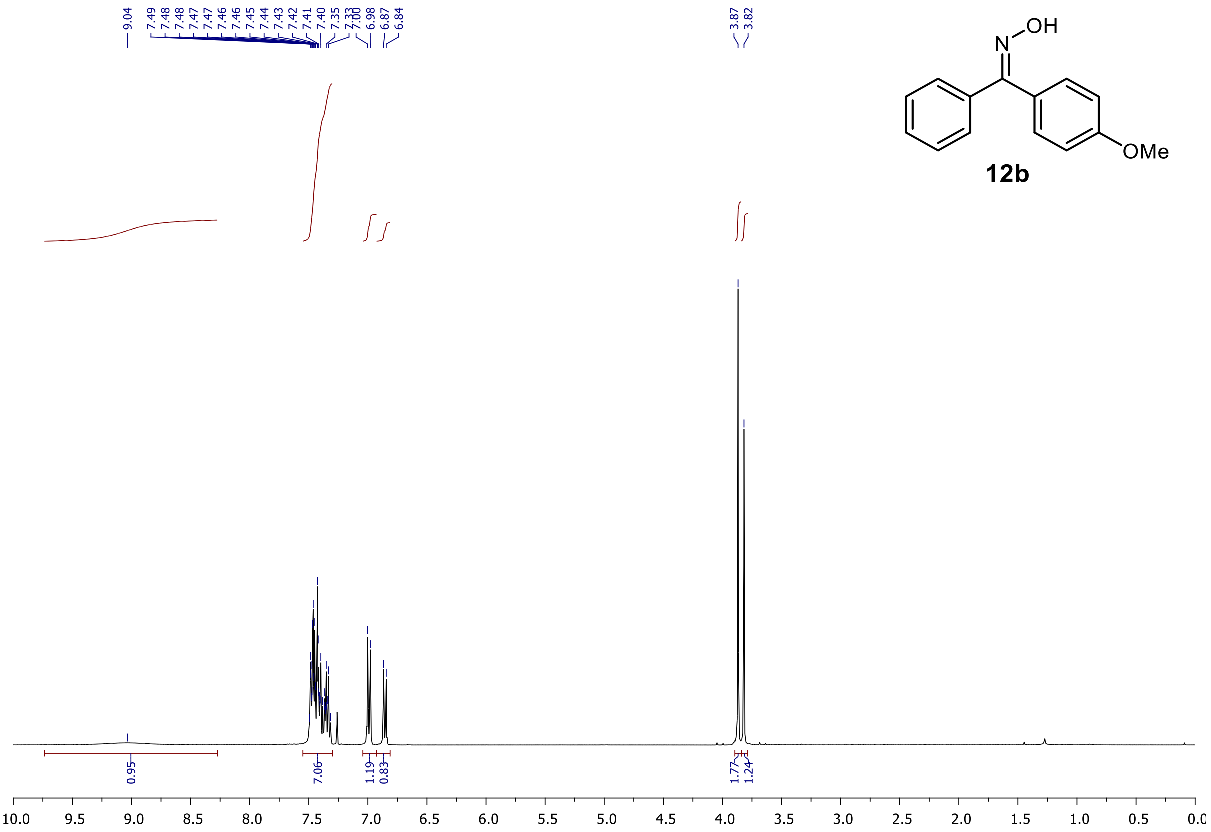

^{13}C NMR (101 MHz, $CDCl_3$)

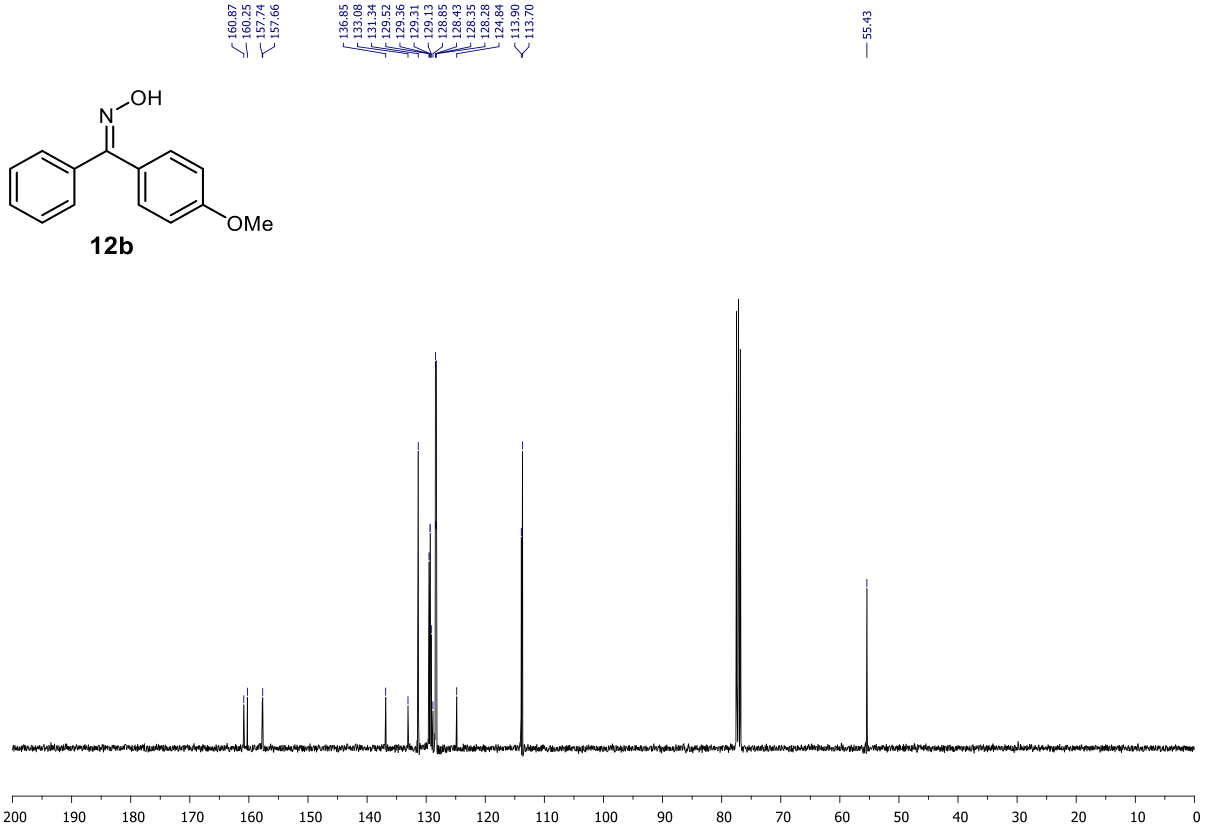

^{1}H NMR (500 MHz, $CDCl_3$)

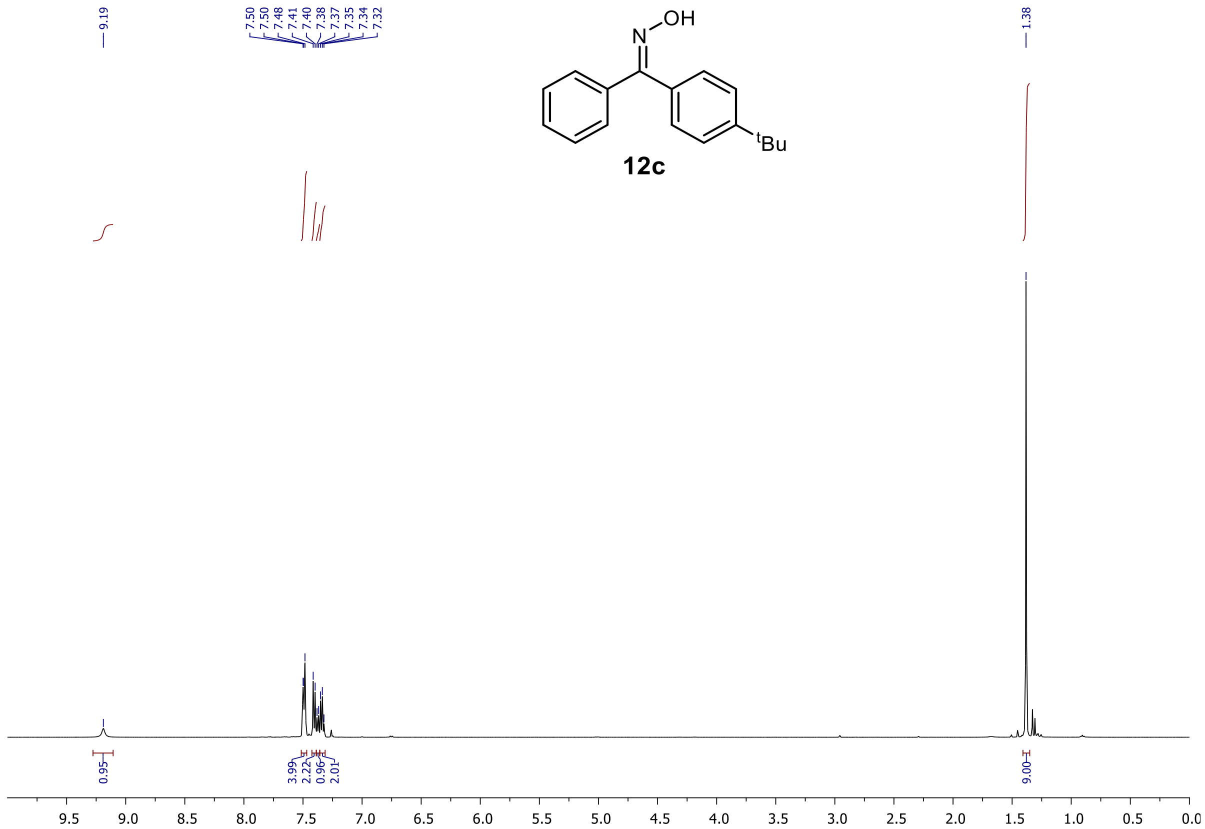

^{13}C NMR (126 MHz, $CDCl_3$)

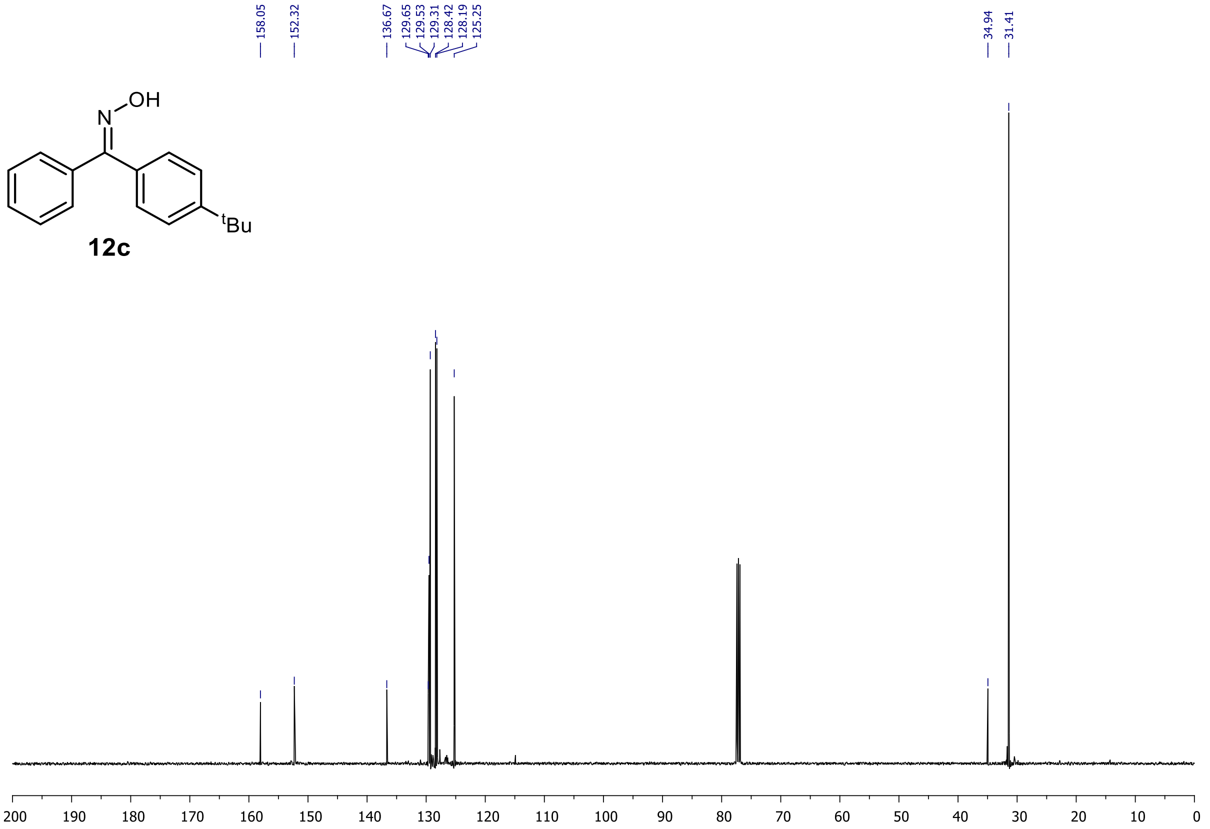

^{1}H NMR (500 MHz, $CDCl_3$)

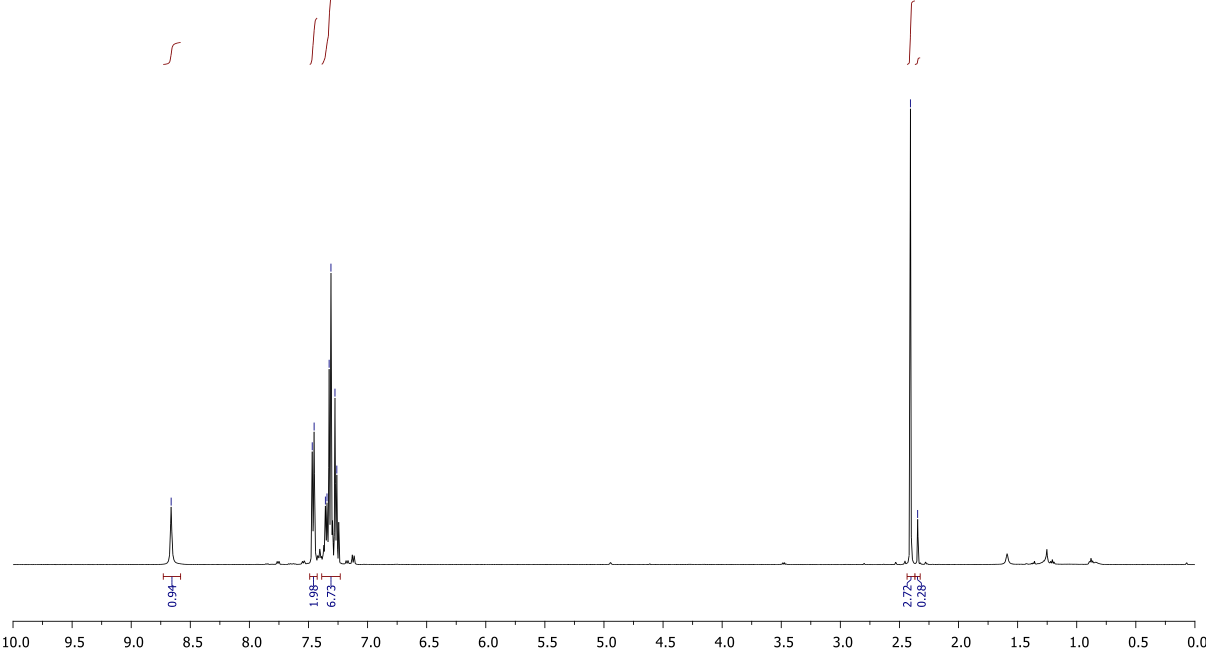

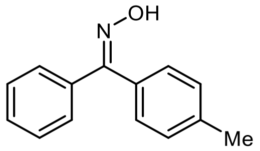

12d

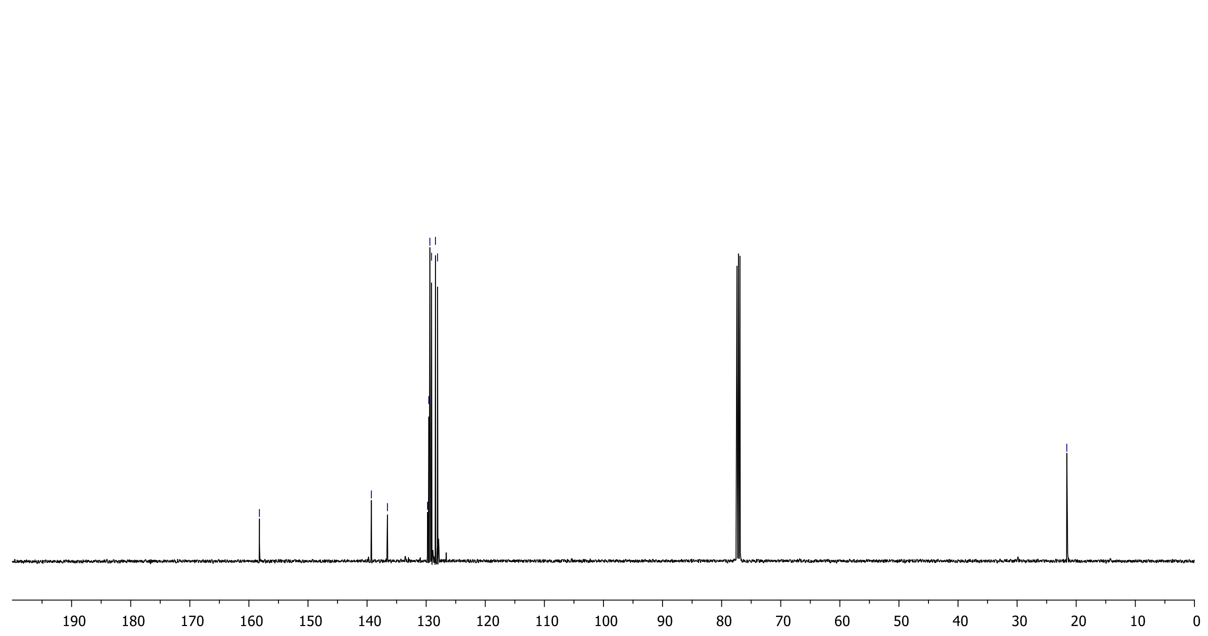

^{13}C NMR (126 MHz, $CDCl_3$)

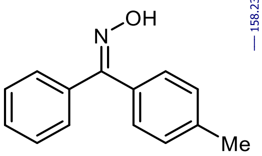

12d

^{1}H NMR (400 MHz, $CDCl_3$)

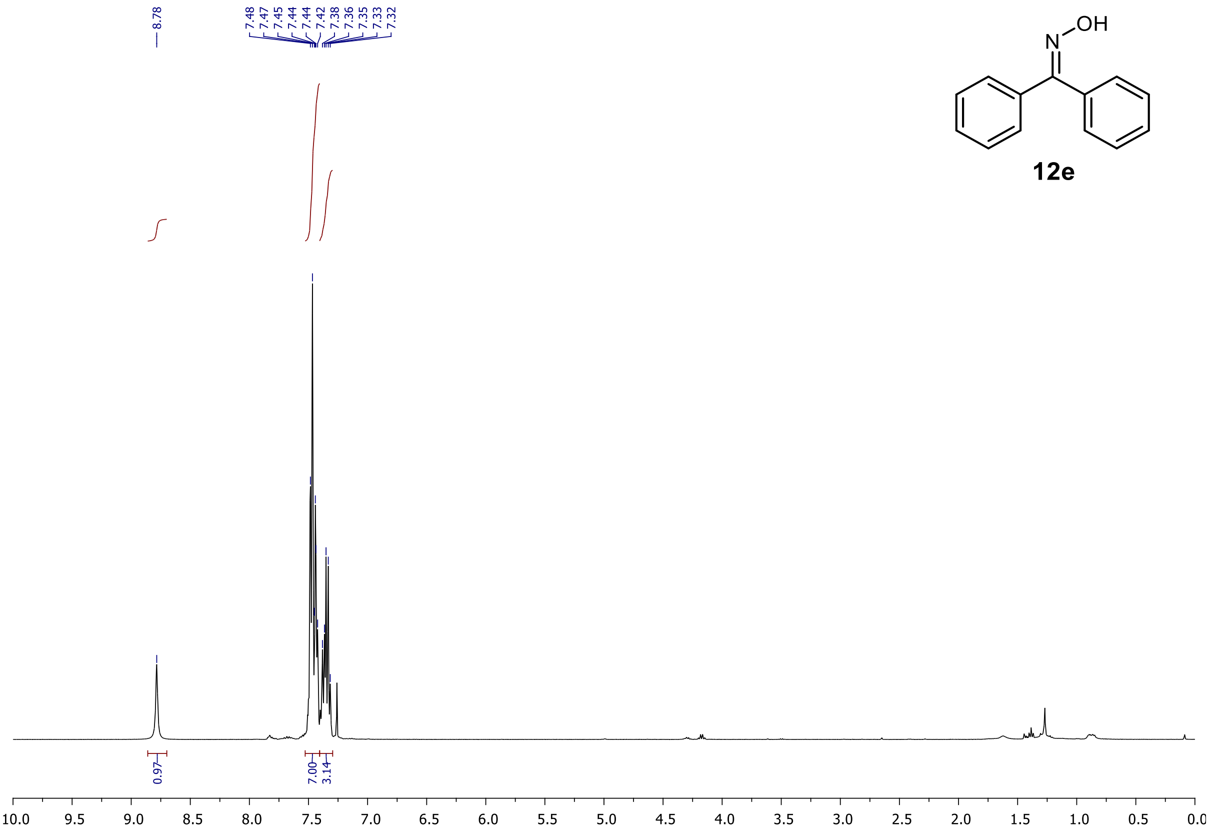

^{13}C NMR (126 MHz, $CDCl_3$)

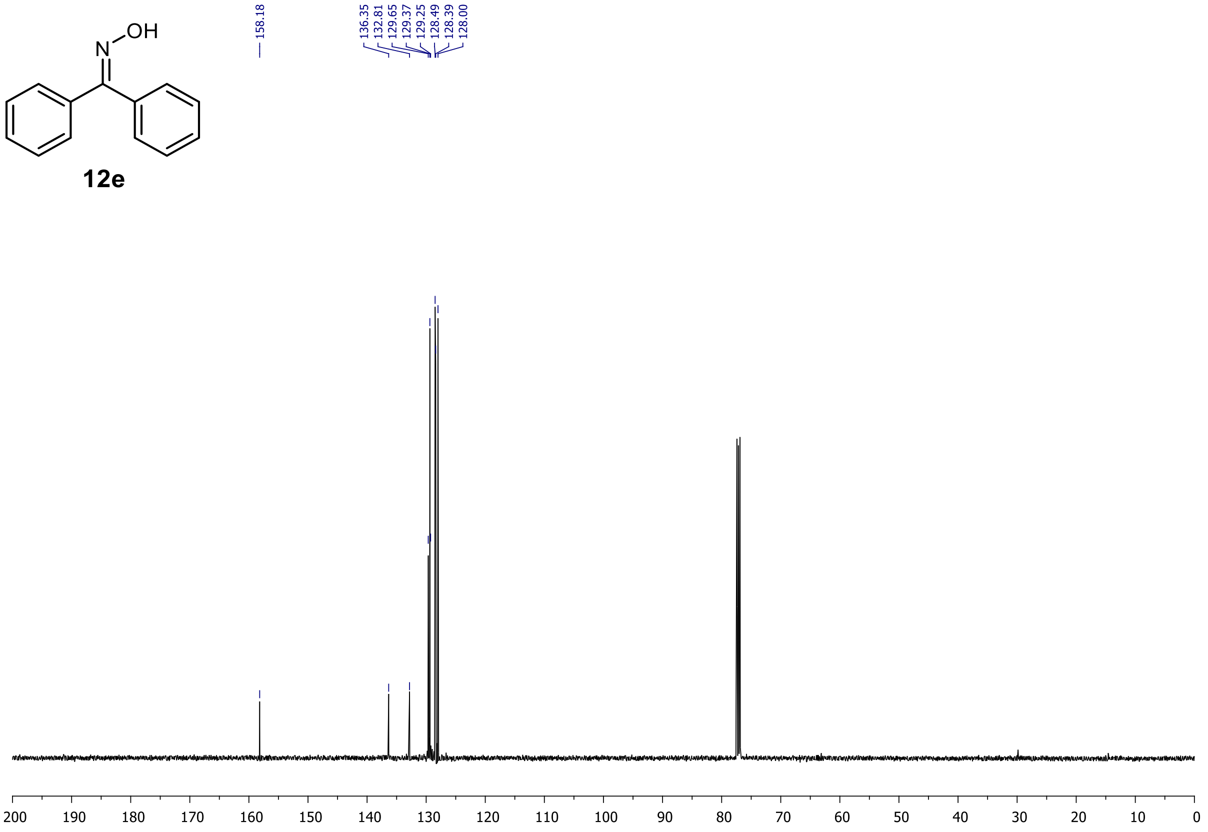

^{1}H NMR (500 MHz, $CDCl_3$)

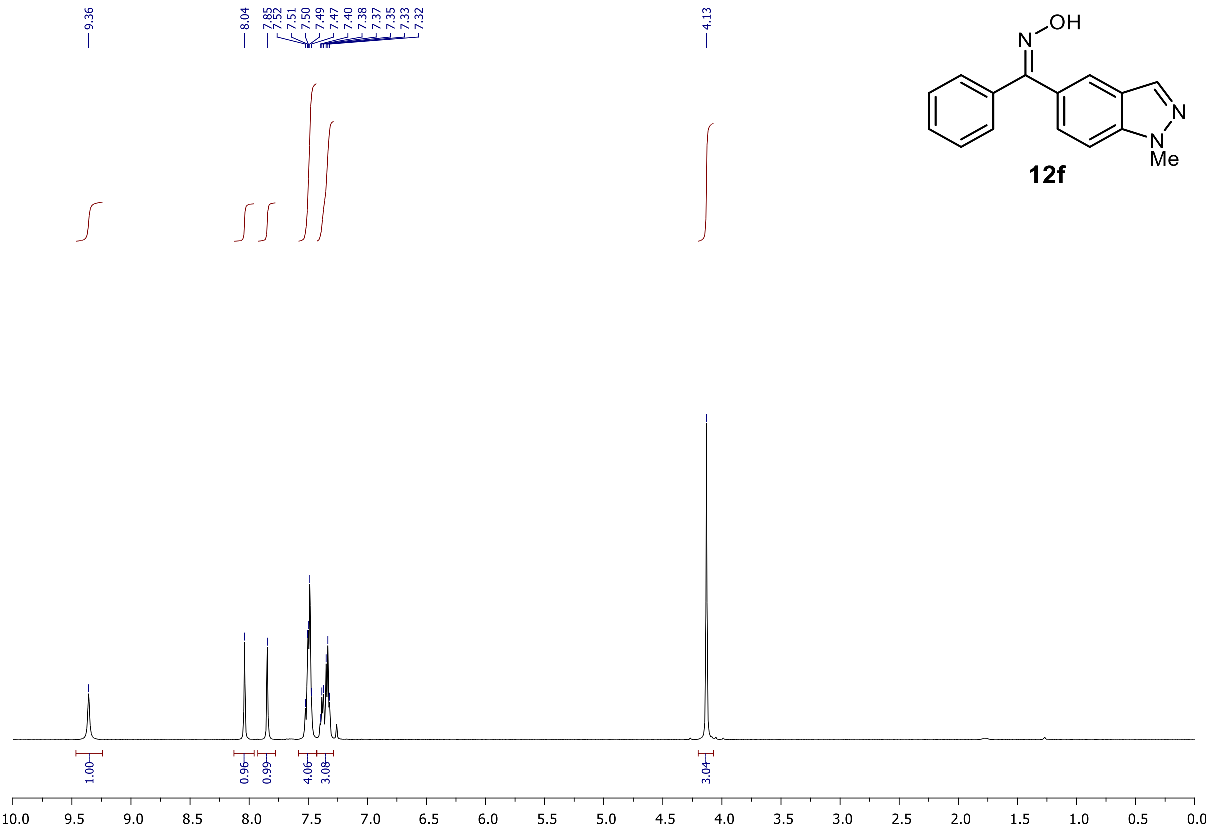

^{13}C NMR (126 MHz, $CDCl_3$)

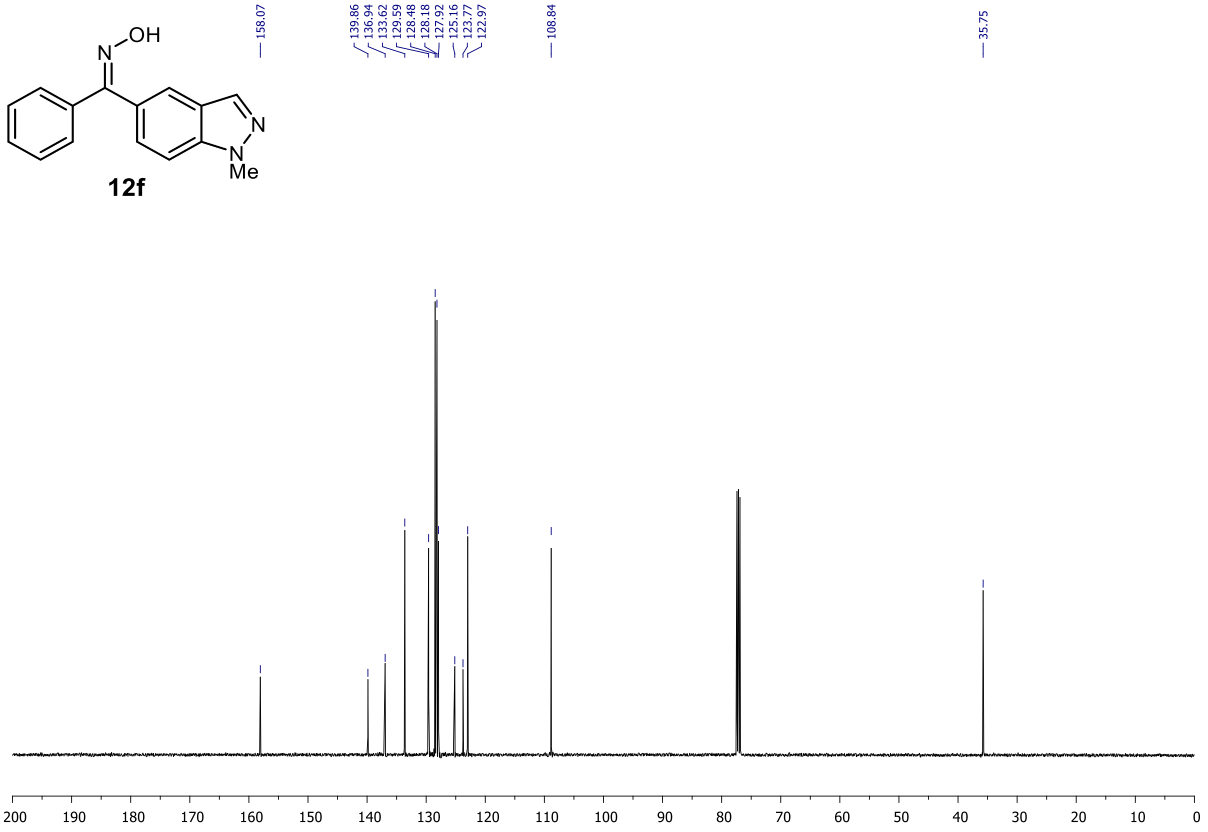

^{1}H NMR (500 MHz, $CDCl_3$)

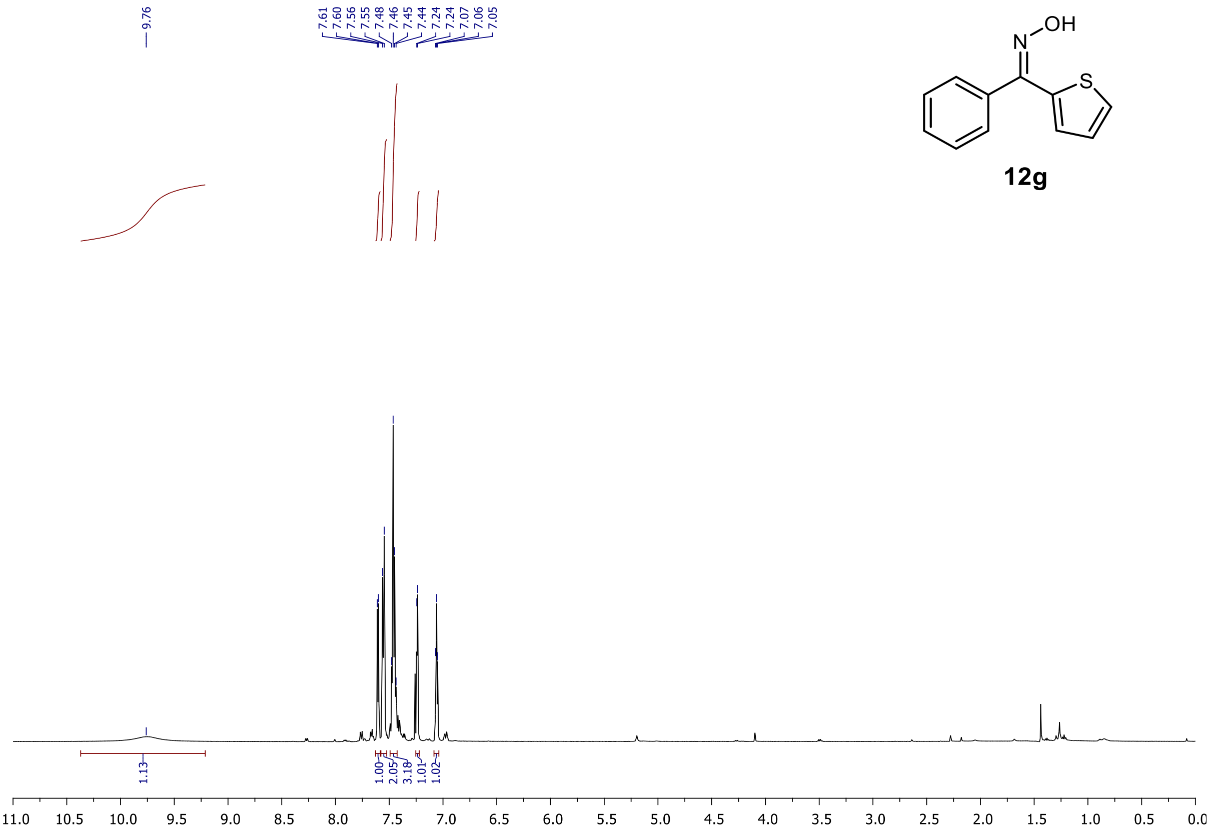

^{13}C NMR (126 MHz, $CDCl_3$)

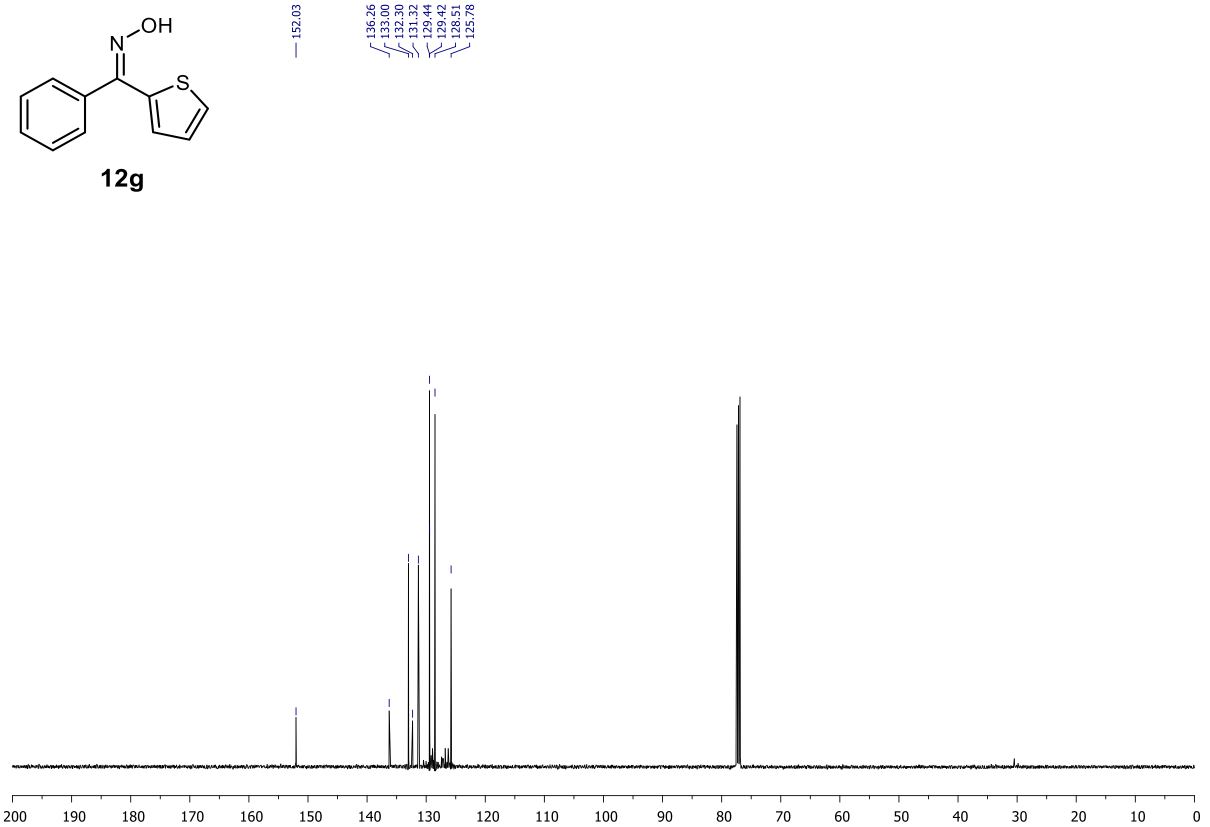

^{1}H NMR (500 MHz, $CDCl_3$)

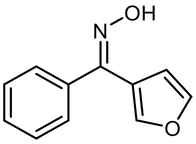

12h

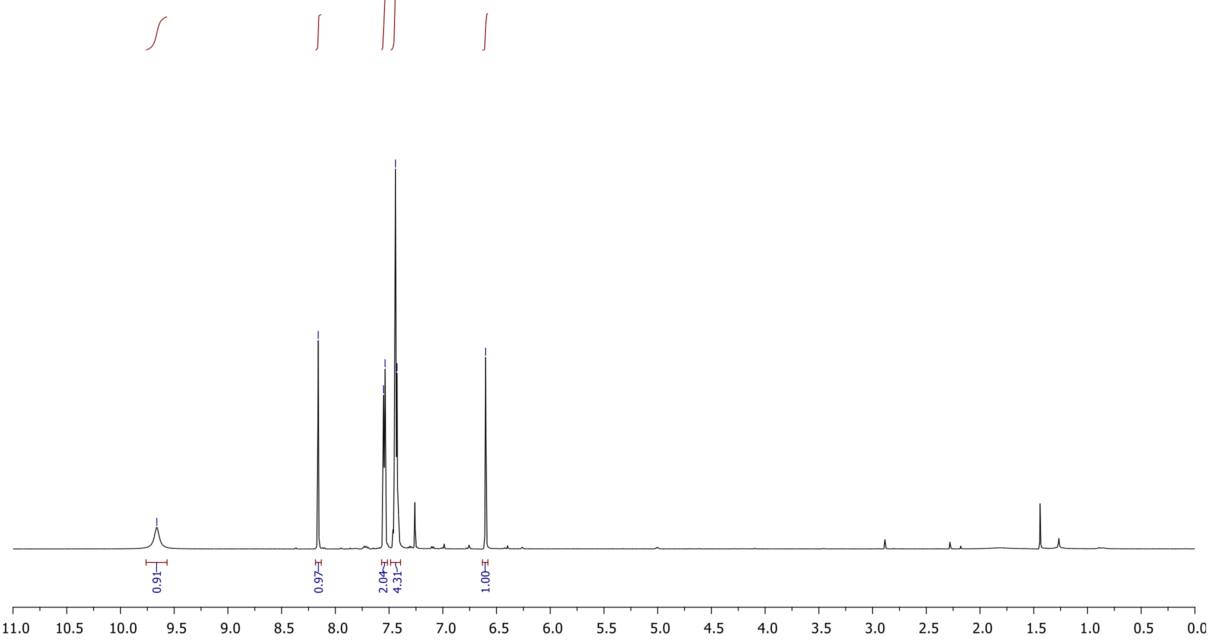

^{13}C NMR (126 MHz, $CDCl_3$)

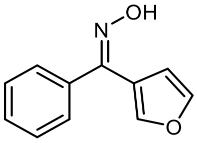

12h

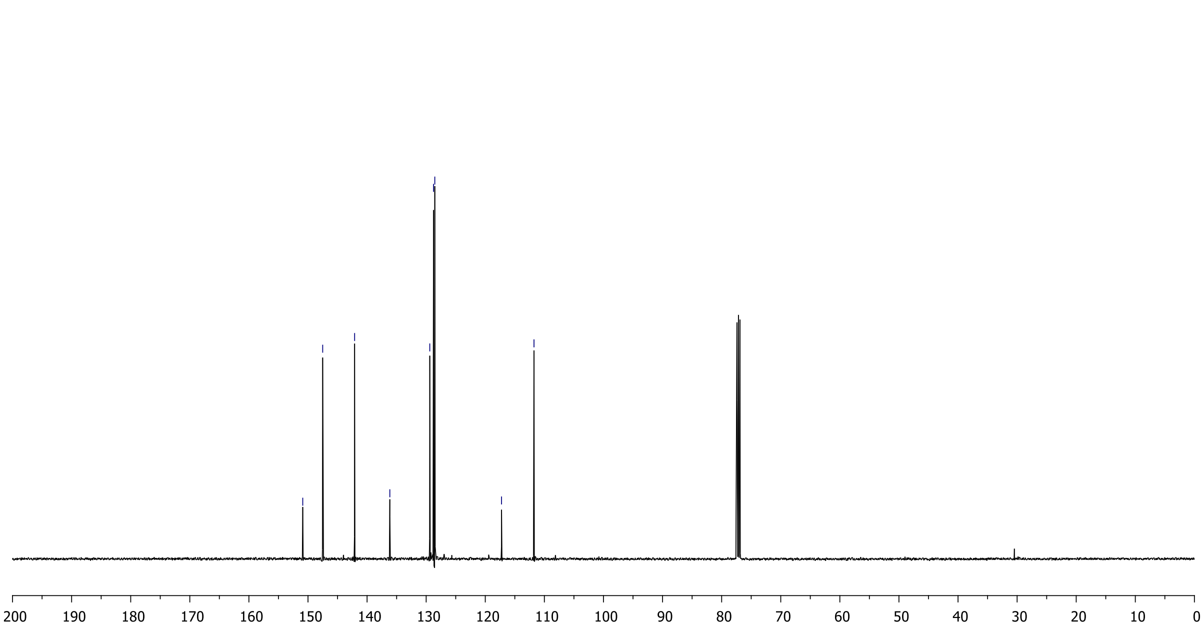

^{1}H NMR (500 MHz, $CDCl_3$)

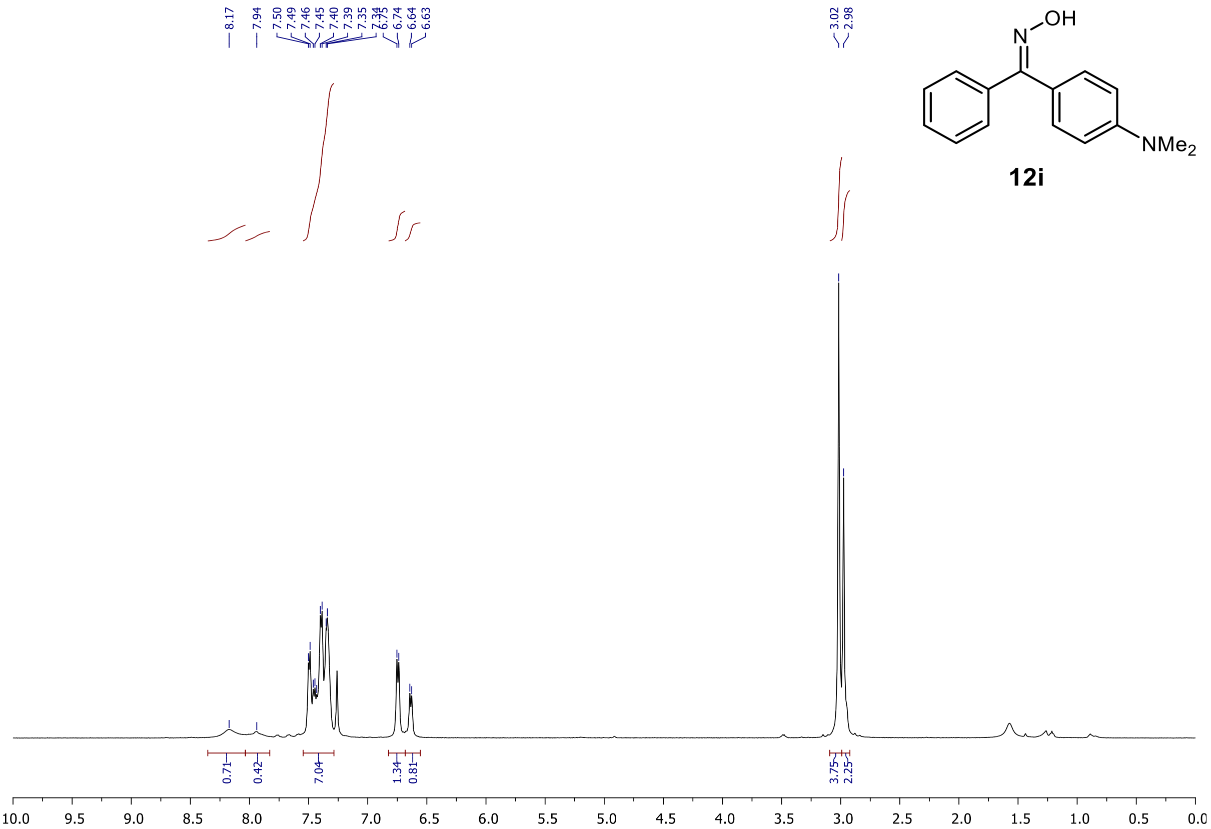

^{13}C NMR (101 MHz, $CDCl_3$)

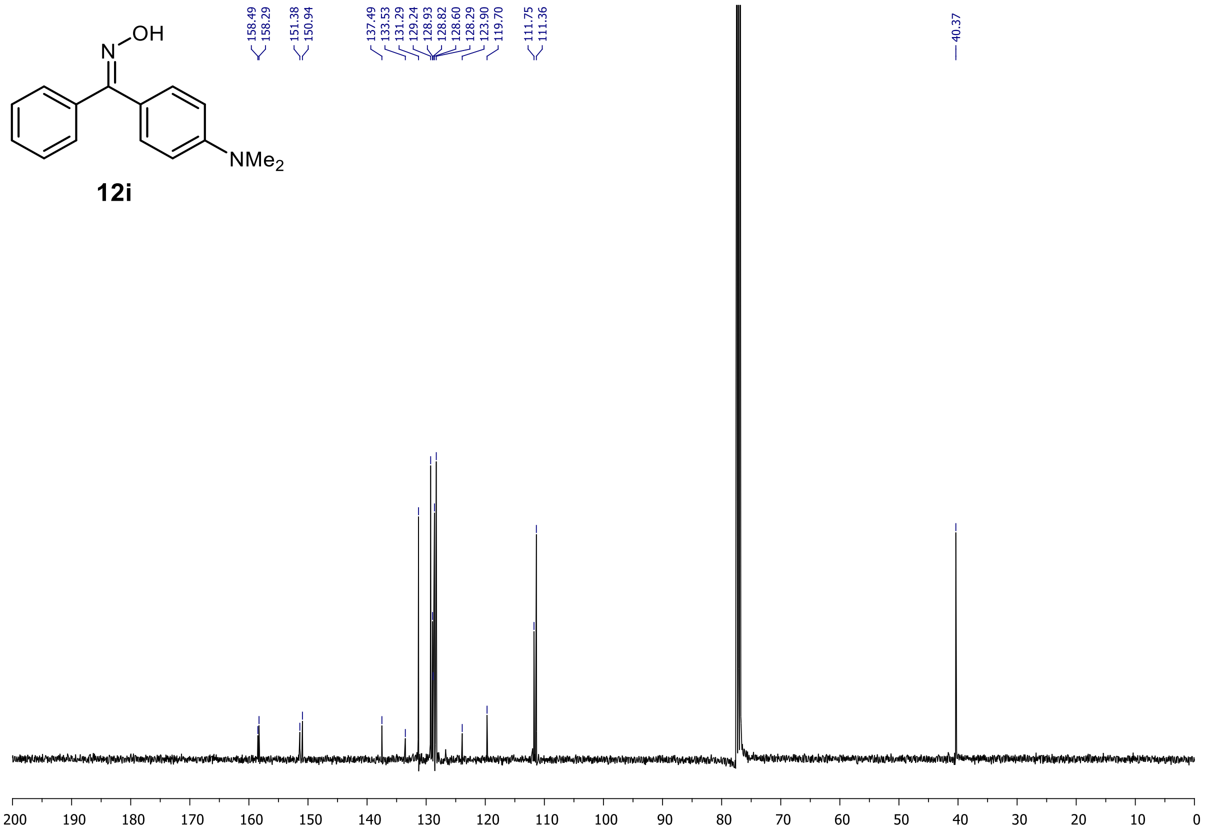

Supplement: Supplementary file 1 — Supplementary [file CHEM-26-10591-s001.pdf]
